# A photochemical method to evidence directional molecular motions

**DOI:** 10.1038/s41467-023-40190-4

**Published:** 2023-07-31

**Authors:** Benjamin Lukas Regen-Pregizer, Ani Ozcelik, Peter Mayer, Frank Hampel, Henry Dube

**Affiliations:** 1 Friedrich-Alexander Universität Erlangen-Nürnberg, Department of Chemistry and Pharmacy, Nikolaus-Fiebiger-Str. 10, 91058 Erlangen, Germany; 2 Ludwig-Maximilians Universität München, Department of Chemistry and Center for Integrated Protein Science CIPSM, Butenandtstr. 5–13, 81377 Munich, Germany

**Keywords:** Reaction mechanisms, Molecular machines and motors, Organic molecules in materials science

## Abstract

Light driven synthetic molecular motors represent crucial building blocks for advanced molecular machines and their applications. A standing challenge is the development of very fast molecular motors able to perform rotations with kHz, MHz or even faster frequencies. Central to this challenge is the direct experimental evidence of directionality because analytical methods able to follow very fast motions rarely deliver precise geometrical insights. Here, a general photochemical method for elucidation of directional motions is presented. In a macrocyclization approach the molecular motor rotations are restricted and forced to proceed in two separate ~180° rotation-photoequilibria. Therefore, all four possible photoinduced rotation steps (clockwise and counterclockwise directions) can be quantified. Comparison of the corresponding quantum yields to the unrestricted motor delivers direct evidence for unidirectionality. This method can be used for any ultrafast molecular motor even in cases where no high energy intermediates are present during the rotation cycle.

## Abstract (web summary)

Light driven synthetic molecular motors represent crucial building blocks for advanced molecular machines and their applications but the development of very fast motors remains challenging. Here the authors restrict fast molecular motor rotations and force them to proceed in two separate rotation-photoequilibria enabling them to elucidate the directionality of the motion.

## Introduction

The control of molecular motions represents a fundamental issue for establishing responsiveness at the nano-scale. It plays a role in virtually any chemical system but has risen to prominence particularly in the fields of molecular switching and molecular machine building^1–8^. Many kinds of molecular motions exist e.g. translations, vibrations, or rotations and such motions are ever-present as inherent (thermal) activity at the molecular scale e.g. the translational Brownian motions or ground state vibrations. To control particular motions at the molecular scale typically requires additional energy input to induce specific ones that are not permanently active in the thermal bath. Such control has many facets and, in most cases, results in reversible switching in which forwards and backwards movements cancel each other out over time. Molecular motors are different as their action result in a net motion that is not canceled over time and thus they represent arguably the most sophisticated control over molecular motions achieved by man today^9–12^. The potential of molecular motors as central powering units of molecular machinery is immense and only starting to be tapped by the imagination of molecular engineers^13–26^.

**Fig. 1:**
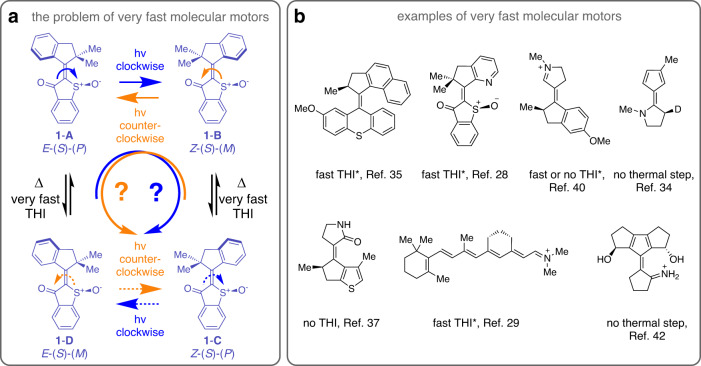
Challenges associated with proving directionality of fast molecular motors. Direct experimental evidence for very fast light-powered molecular motors is difficult to obtain because of the fleeting character of intermediates (e.g. because of very fast thermal helix inversion (THI)) or their complete absence. **a** Fast THI (Δ) in motor **1** (purple) and the resulting structural lability allows for different feasible rotation directions (clockwise depicted by blue arrows and counterclockwise depicted by orange arrows). A preferred potoisomerization (hv) direction following the initial helical twist of the respective starting isomer is assumed. Stereo assignments are given for all structures. **b** A variety of very fast molecular motors with highly flexible helicities or missing thermal steps in the ground state. In the latter case elucidation of directionality is severely hampered because of non-existing intermediates. Examples marked with * were examined experimentally and theoretically and all other examples were scrutinized solely by theory.

At the heart of molecular motor building lies evidencing their unidirectional motion in the first place. This typically requires a step-by-step following of the different molecular changes leading to the overall motion in the end^27^. Depending on the inherent speed, mechanism, and molecular setup of a molecular motor such elucidation can however be very tedious if not impossible by experiment alone. The crucial problem for very fast molecular motors is the fleeting character of intermediates or even complete lack of them, which obscures an unambiguous evidencing of the trajectory for each motion step (Fig. 1a). One such example is a recently disseminated potentially ultrafast hemithioindigo (HTI) molecular motor from our group (Fig. 1b)^28^ and another one is the here presented molecular motor **1** (Fig. 1a), both of which undergo very fast thermal helix inversion (THI) steps. Further examples of (prospective) very fast molecular motors by the groups of Andersen and Bochenkova^29^, Durbeej^30–34^, Feringa^35,36^, Filatov^37–39^, Olivucci^40,41^, or Sampedro^42^ are shown in Fig. 1b. Since individual steps in these motor mechanisms are very fast, spectroscopic methods able to follow the processes (i.e. ultrafast spectroscopy as used in^41^ and^43–46^) are typically not delivering detailed enough information about particular configurations, conformations, and geometry changes. In cases where no intermediate thermal steps are present, directionality cannot be evidenced by following a step-by-step sequence of state populations at all.

**Fig. 2:**
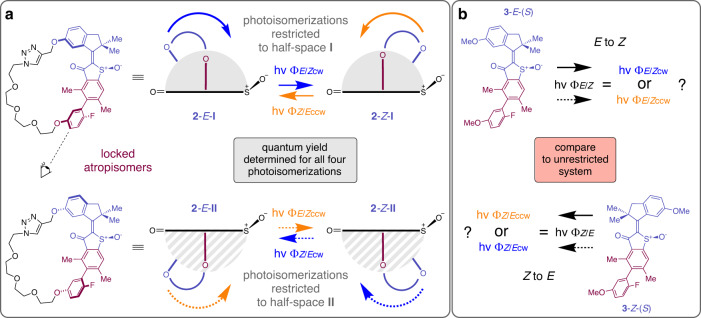
Concept for photochemical elucidation of very fast molecular motor rotations. Indanone rotor and part of the benzothiophenone stator are colored purple and the biaryl moiety is depicted in dark red. **a** Macrocyclic setup **2** with locked atropisomers of the biaryl moiety (purple) restricting the motor (blue) motions to only one half-space with respect to the sulfoxide oxygen (grey half cycles **I** (solid arrows) and dashed grey half cycles **II** (dotted arrows)). Quantum yields *Φ* for all four photoisomerization (hv) directions can thus be measured individually (expected clockwise (cw) direction indicated by blue arrows, opposite counterclockwise (ccw) direction by orange arrows). **b** Comparison of quantum yields with the same non-macrocyclic molecular motor **3** (black arrows) allows evidencing its unidirectional motion.

For this reason, we have devised a different method in this work, enabling to elucidate the directionality of a prospective very fast molecular motor in a photochemical way. To this end, the molecular motor is embedded inside a macrocyclic structure **2** and covalently connected to a sterically strongly hindered biaryl moiety via a flexible linker chain as shown in Fig. 2a (for other molecular motors embedded into macrocycles see^13,19–22,24,25,47–49^). Because of severe sterical hindrance of the biaryl and the restraining length of the linker chain, the motor motion is restricted to one half-space of the macrocycle as defined by the particular axial chirality of the biaryl unit. This molecular setup allows separating the two **~**180° half-rotations (termed **I** and **II** in the following) of the motor and force it to execute both clockwise (cw, as depicted in blue arrows and numbers in the following) and counterclockwise (ccw, as depicted in orange arrows and numbers in the following) rotations in each half-space instead of undergoing a full cyclic motion. When comparing particularly the quantum yields of both rotation directions in macrocyclic **2** with the measured quantum yields of the unrestricted molecular motor **3** (Fig. 2b), the directionality of the latter can then be determined straight forwardly despite its very rapid motions. For this purpose, the quantum yields for the clockwise (blue arrows in Fig. 2a) and counterclockwise rotations (orange arrows in Fig. 2a) within macrocycle **2** need to be significantly different. Then the quantum yield of e.g. the *E* to *Z* photoisomerization of **3** can be matched to only one particular quantum yield measured for the corresponding *E* to *Z* photoisomerization in **2**, evidencing the rotation direction for the former. Implicitly, the particular quantum yield measured for **3** (and matched to **2**) thus reports on the exact helicity of the starting and the final structure in the particular photoisomerization step. Similarly, this can be done for the opposite *Z* to *E* photoisomerization. For an animated visualization of the presented concept, see Supplementary Movie 1.

## Results

**Fig. 3:**
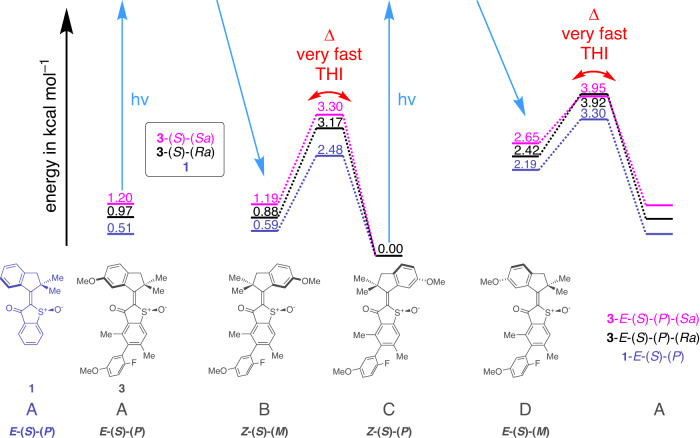
Theoretical prediction of directionalities. Theoretically predicted (B3LYP/6-311 G(d,p) PCM (CH_2_Cl_2_) level of theory) potential energy landscape of HTI motors **1** (purple) and the two atropisomers of **3** ((*S*)-(*R*_a_) shown in black and (*S*)-(*S*_a_) shown in pink). Very fast thermal helix inversion (THI) steps (Δ) are indicated with red arrows. Stereo assignments are given for all structures.

Molecular motors of type **1** are based on the HTI chromophore^50,51^ and bear strong resemblance to the first HTI motor published by our group in 2015^52^, the mechanism of which was fully elucidated in a later study^45^. However, different to the earlier design, we have removed the methoxy substituent in close proximity to the central isomerizable double bond and thus significantly accelerated the speed of the thermal steps in the motion cycle. Based on a theoretical description on the B3LYP/6-311 G(d,p) PCM (CH_2_Cl_2_) level of theory HTI **1** is predicted to behave as an unidirectional molecular motor populating four different states **A** to **D** in sequence during one 360° rotation (Fig. 3). Photoisomerization steps are intrinsically ultrafast for HTI-molecular motors and for motor **1** also the intersecting thermal helix inversion (THI) steps (leading from **B** to **C** and from **D** to **A**) are now predicted to be very fast in the ns time regime at ambient temperatures. Because of these fast thermal isomerizations it is theoretically possible that helicity of **1** is equilibrized quickly and thus photoisomerization steps could in principle start from either helix. The theoretical treatment predicts a reduced energy difference Δ*G* between the different helical states in comparison to the original HTI motor, especially for *Z*-configured isomers **B** and **C**. For the latter two isomers, population of the unfavorable helical state **B** is thus predicted to be non-zero and the backwards motion (corresponding to a counterclockwise rotation in the angle of view of Fig. 1) could be possible leading from **B** to **A** under continuous irradiation. Such behavior would not necessarily compromise overall directionality of **1** but diminish quantitative unidirectionality.

A very similar behavior is observed for the substituted motor derivative **3**, however, the minimum energies predict a more favorable degree of overall unidirectionality because of the higher lying metastable states (Fig. 3). In this case two different atropisomers of **3** need to be distinguished with either (*R*_a_) or (*S*_a_) configuration of the biaryl axis. These diastereomers, termed **3**-(*S*)-(*S*_a_) and **3**-(*S*)-(*R*_a_) in the following, resemble each other in their energy profile closely with the **3**-(*S*)-(*S*_a_) isomers generally possessing slightly higher energy states and higher degree of directionality overall.

Despite the theoretical analysis experimental evidence is needed to establish the actual motion directionality and this task is non-trivial for HTIs **1** and **3** since thermal steps are very fast. Consequentially, only two states with either *E* or *Z* configuration of the central double bond are directly observable for **1** and **3** with common analytic methods, which could represent states of pure *M* or *P* helicity or a mixture of both. The interconversion between just two states precludes a tracing of the associated motions directionalities via intermediate states and thus renders the rotation ambiguous.

To solve this general problem for any very fast molecular motor, we devised a photochemical method to elucidate directionality. Motor core structure **1** was embedded into a macrocyclic structure (**2**), which contains a highly sterically hindered biaryl moiety. Because of the strong sterical hindrance, imposed by two methyl substituents and one fluorine substituent in *ortho*-position to the biaryl axis, four isomers can now be distinguished and isolated as shown in Fig. 2. Two thermally stable atropisomers are possible in macrocylic structure **2**, either connecting the biaryl to the motor at the same half-space as the sulfoxide oxygen atom (termed **I**) or at the opposite half-space (termed **II**). For each of the atropisomers, the central double bond can then adopt either *E* or *Z* configuration. Since no atropisomerization of the macrocyclic system **2** takes place during photoirradiation or heating below 80 °C, isomerization steps of the embedded motor moiety are now restricted and split to individual **~**180° movements in either half-space separately.

**Fig. 4:**
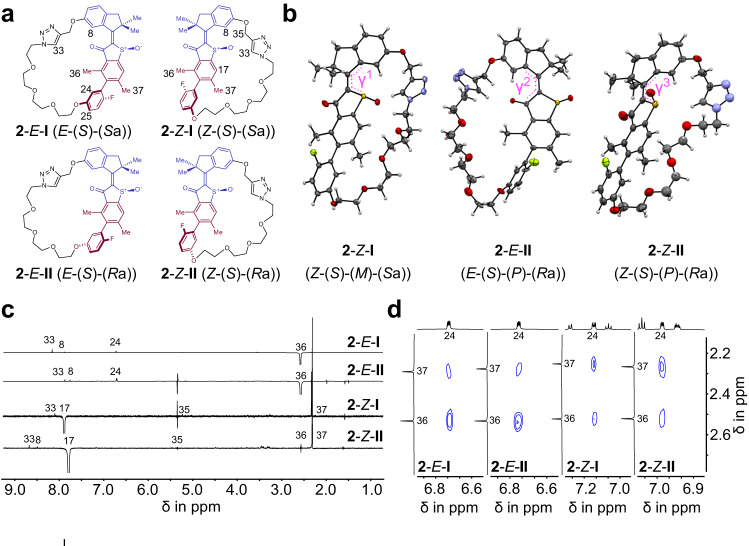
Structural and conformational description of macrocycle 2 in solution and in the crystalline state. Isomeric structures of macrocycle **2** with stereo assignments given for all structures. **a** Schematic representations of the different isolated diastereomeric structures. The indanone rotor and part of the benzothiophenone stator are colored purple and the biaryl moiety is colored in dark red. Helicity assignments were omitted because of the highly dynamic helix inversion. **b** Structure of **2**-*Z*-**I** (*Z*-(*S*)-(*M*)-(*S*_a_) configuration shown), **2**-*E*-**II** (*E*-(*S*)-(*P*)-(*R*_a_) configuration shown), and **2**-*Z*-**II** (*Z*-(*S*)-(*P*)-(*R*_a_) configuration shown) in the racemic crystalline state with 50% probability ellipsoids. Hydrogen atoms are colored white, carbon atoms grey, oxygen atoms red, nitrogen atoms blue and fluorine atoms yellow. Dihedral torsion angles γ^1^ − γ^3^ around the central double bond are depicted in pink. **c** Indicative segments of NOE NMR (600 MHz, 25 °C, CD_2_Cl_2_) spectra of **2**-*E*-**I** to **2**-*Z*-**II** evidencing the configuration of the central double bond. **d** Indicative segments of 2D NOESY NMR (800 MHz, 25 °C, CD_2_Cl_2_) spectra with cross signals of **2**-*E*-**I** to **2**-*Z*-**II** colored blue evidencing the tilt of the biaryl axis in solution.

Four diastereomers of macrocycle **2** could be separated, which represent the two different atropisomers with either *E* or *Z* configured double bond (Fig. 4a). The molecular structures of three racemic isomers **2**-*Z*-**I,** **2**-*E*-**II**, and **2**-*Z*-**II** could be elucidated directly by X-ray diffraction in the crystalline state (see Fig. 4 wherein stereochemical assignments are also given). For **2**-*Z*-**I** and **2**-*E*-**II** the expected more stable (*P*)-helicity of the motor is observed in the crystal for diastereomers with (*S*)-configured sulfoxide stereocenter. For **2**-*Z*-**II** (*M*)-helicity is observed instead. The latter observation lends further support to the theoretical description predicting a smaller energy difference between the two *Z* isomer helicities.

A corresponding analysis of the different structures in solution by NMR spectroscopy revealed close similarities to the crystalline state and allowed to identify and assign important features of the isomeric structures (for details see the Supplementary Information). Confirming the configurations of the double bond in the different isomeric states in solution was possible using NOE NMR spectroscopy (Fig. 4b). The assignments are further supported by the presence of a noticeable tilt of the biaryl axis as revealed by NOESY NMR (Fig. 4c). In every case a preferred tilt following the *E* or *Z* configuration of the double bond through the macrocyclic connection is observed. Assignment of the relative configuration of the biaryl chiral axis with respect to the sulfoxide stereocenter – and thus determination of the half-space **I** or **II** in which the photoisomerization is forced to take place – was also straight forward for all four isomers in solution, since the biaryl axis is thermally highly stable. Therefore, the relative configurations could be inferred from the three structures in the crystalline state leaving only one option for the fourth isomer. Because of the very fast THI between structures in solution an unambiguous assignment of (*P*) or (*M*) helicity was however not possible. Despite the intrinsically highly dynamic helicity a substantial helical twist was observed in the crystalline state for structures **2**-*Z*-**I,** **2**-*E*-**II**, and **2**-*Z*-**II**, as measured by the torsion angles γ^1^ = 13.6°, γ^2^ = 15.5°, and γ^3^ = 11.5° shown in Fig. 4b. Compared to the original HTI motor the initial asymmetric helicity, which is believed to dictate a favorable rotation direction, thus turns out to be similarly pronounced in the motors of structure **1**-**3**. Previous theoretical analyses suggest that even very small initial asymmetry biases can lead to full directionality of a molecular motor rotation^33,53^.

**Fig. 5:**
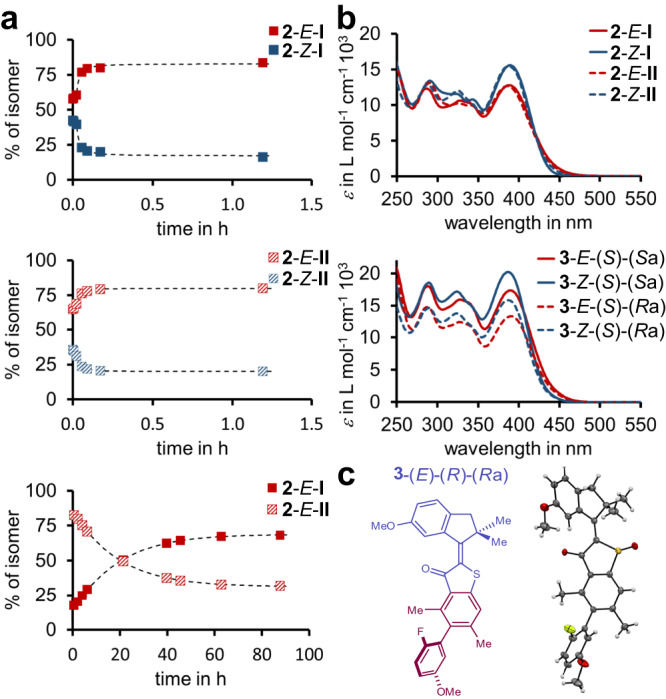
Absorption, thermal behavior, and structure in the crystalline state of macrocyle 2 and motor 3. *E*-configurations are depicted in red and *Z*-configurations in blue. Atropisomers with *R*_a_-configurations are shown with dashed and *S*_a_-configurations with solid markers and lines. **a** Thermal reactions of **2** measured in (CD_2_Cl_2_)_2_ solution at 40 °C (top and middle graph) or 80 °C (bottom graph). Thermal *Z* to *E* isomerizations (top and middle graph) are significantly faster than thermal atropisomerization (bottom graph). **b** Molar absorption coefficients of the four different isomers of macrocycle **2** (top) and of the four different isomers of motor **3** (bottom). **c** Structure of enantiomerically pure **3**-*E*-(*R*)-(*R*_a_) in the crystalline state with 50% probability ellipsoids and corresponding schematic depiction with the indanone rotor and part of the benzothiophenone stator colored in purple and the biaryl axis in dark red. Hydrogen atoms are colored white, carbon atoms grey, oxygen atoms red, nitrogen atoms blue, and fluorine atoms yellow.

After clarifying double bond configurations and relative atropisomerims (biaryl axial chirality with respect to the sulfoxide oxygen, i.e. structures **I** versus **II**) of the different states of macrocycle **2**, the thermal as well as photochemical behavior could be studied in detail (Fig. 5a). Heating experiments revealed that each of the four isolated isomers undergoes thermal double bond isomerization at slightly elevated temperatures (40 °C) while no atropisomerization of the biaryl axis takes place. The latter is only observed at significantly higher temperatures (80 °C). Thermal double bond isomerization within the macrocycle **2** proceeds from the thermodynamically less stable *Z* isomers to the more stable *E* isomers and follow first order kinetics. This behavior is different to the parent motor structures **1** and **3** for which the *Z* isomers are thermodynamically most stable. Similar effects of macrocyclization on the relative isomer stabilities of HTI-based molecular motors have been observed earlier as well^21,22^. At ambient temperatures the photochemistry of **2** is thus not perturbed by competing thermal double bond isomerization or atropisomerization reactions.

The photochemistry of macrocycle **2** was determined next (Fig. 5b). All four individual isomers **2**-*E*-**I,** **2**-*Z*-**I,** **2**-*E*-**II**, and **2**-*Z*-**II** were used in pure form as starting points for irradiation experiments. First the selectivities of individual isomer interconversions were clarified. Irradiation with blue light at ambient temperatures revealed that only *E*/*Z* or *Z*/*E* photoisomerization took place but no light-induced atropisomerization of the biaryl axis. Thus, isomer **2**-*E*-**I** only photoconverts into isomer **2**-*Z*-**I** and vice versa as well as isomer **2**-*E*-**II** only photoconverts into isomer **2**-*Z*-**II** and vice versa. This behavior reveals that the photoisomerization reactions are restricted to only one half-space (**I** or **II**) of the molecular plane as distinguished by the sulfoxide oxygen atom. It is thus feasible to measure quantum yields of all four possible photoisomerization directions of the motor individually in the macrocyclic derivative **2** (Fig. 2a). Such a quantification of the complete photochemistry, including backwards-photoisomerization reactions, has only been achieved so far for a considerably slowed down HTI molecular motor at very low temperatures by using a special in situ irradiation and photocounting approach^54^.

For the motor setups **1** and **3** there is an unifying expected directionality, similar to well characterized slower HTI motors, as predicted by the theoretically obtained energy profiles shown in Fig. 3. As it turns out the photoreactions associated with this expected directionality – i.e. from isomer **2**-*E*-**I** to isomer **2**-*Z*-**I** (*Φ* = 3.5%) and from isomer **2**-*Z*-**II** to isomer **2**-*E*-**II** (*Φ* = 9.3%) – do not necessarily possess the highest quantum yields. In fact, the quantum yield for the backwards (counterclockwise rotation marked in orange in the Figures) photoisomerization from isomer **2**-*Z*-**I** to isomer **2**-*E*-**I** (*Φ* = 16.1%) is standing out as the largest quantum yield overall. This finding is however in very good agreement with the corresponding previously measured quantum yield of a much slower HTI molecular motor^55^ at low temperatures^54^ and thus corroborates on a general trend in this class of compounds. Most importantly the quantum yields for all rotation directions of the motor unit in macrocycle **2** are significantly different to each other to be used for a meaningful comparison. In detail, for the *E* to *Z* photoisomerization the expected clockwise rotation proceeds with *Φ*_*E*/*Z*cw_ = 3.5% and the counterclockwise rotation with *Φ*_*E*/*Z*ccw_ = 4.4%. For the *Z* to *E* photoisomerization the expected clockwise rotation proceeds with *Φ*_*Z*/*E*cw_ = 9.3% and the counterclockwise rotation with *Φ*_*Z*/*E*ccw_ = 16.1%.

**Fig. 6:**
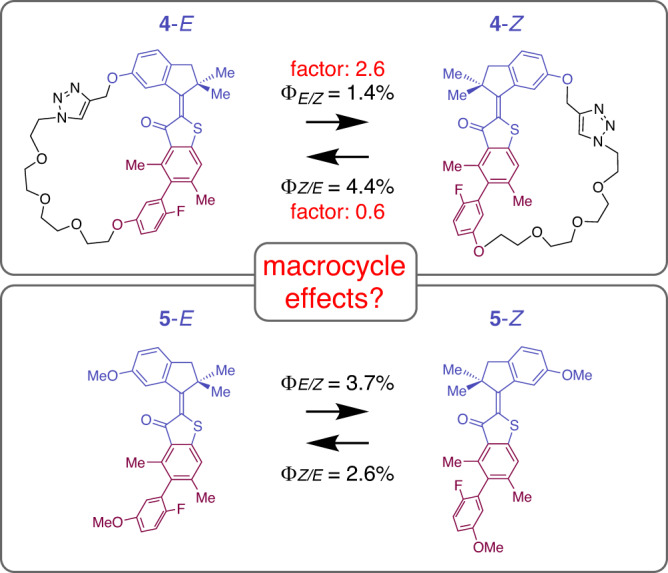
Effect of the macrocyclization on quantum yields. Measurement of the macrocyclization effect on the photoisomerization quantum yields using unoxidized macrocycle **4** and unoxidized unrestricted HTI **5**. The indanone rotor and part of the benzothiophenone stator are colored purple and the biaryl moiety dark red. Macrocyclization effects are given as red colored factors. A significant macrocyclization effect is observed for the *E* to *Z* photoisomerization.

At this point, the effect of macrocyclization on the quantum yields needs to be taken into consideration in order to make correct assignments. To measure the influence of macrocyclization on the quantum yields we compared the unoxidized macrocycle **4** with the unoxidized unrestricted HTI **5** for both *Z* to *E* and *E* to *Z* photoisomerization reactions. In macrocycle **4** clockwise and counterclockwise rotations in the two half spaces **I** and **II** are enantiomeric to each other and thus will give the same quantum yield. Likewise, for HTI **5** the two rotation directions are enantiomeric to each other and give the same quantum yield. The measured macrocyclization effects on the quantum yield are depicted in Fig. 6. As it turns out macrocyclization has a smaller effect on the *Z* to *E* photoisomerization but a significant effect on the *E* to *Z* photoisomerization. For the latter, macrocyclization leads to almost a doubling of the quantum yield, resulting in a considerable underestimation of this value. Therefore, the quantum yield measurements for *Z* to *E* and especially for *E* to *Z* photoisomerizations in the macrocyclic system **2** need to be corrected by the respective factors of 0.6 and 2.6. All measured and corrected quantum yield values are summarized in Table 1.

**Table 1:** Comparison of measured quantum yields

Isomer	*Φ*_aver._ (%)	Confidence Interval (CI)
**C 2**-*E*-**I**	3.45	3.45 ± 0.87
**D 2**-Z-**I**	16.13	16.13 ± 0.87
**A 2**-*E*-**II**	4.37	4.37 ± 0.87
**B 2**-*Z*-**II**	9.26	9.26 ± 0.87
**3**-*E*-(*S*)-(*S*_a_)	3.99	3.99 ± 0.87
**3**-*Z*-(*S*)-(*S*_a_)	6.99	6.99 ± 0.87
**3**-*E*-(*S*)-(*R*_a_)	4.86	4.86 ± 0.87
**3**-*Z*-(*S*)-(*R*_a_)	7.81	7.81 ± 0.87
**4**-*E-(Ra/Sa)*	1.41	1.41 ± 0.87
**4**-*Z-(Ra/Sa)*	4.37	4.37 ± 0.87
**5**-*E-(Ra/Sa)*	3.73	3.73 ± 0.87
**5**-*Z-(Ra/Sa)*	2.60	2.60 ± 0.87
**macrocycle effect** ***E***	2.64	
**macrocycle effect** ***Z***	0.59	
**macrocyc. corr. C 2**-*E*-**I**	8.97	
**macrocyc. corr. D 2**-Z-**I**	9.68	
**macrocyc. corr. A 2**-*E*-**II**	11.36	
**macrocyc. corr. B 2**-*Z*-**II**	5.56	

Quantum yields *Φ* of macrocycle **2**, non-macrocyclic **3**, unoxidized macrocycle **4** and unoxidized non-macrocyclic **5** were measured at 25 °C in CH_2_Cl_2_ solution. Derivatives **2** and **3** were irradiated with a 400 nm LED whereas for **4** and **5** a 450 nm LED was used. Given errors include the maximum errors of molar absorption and quantum yield measurements (see Supplementary Information for individually determined errors). The data set of each measured isomer was considered normally distributed to calculate the confidence intervals (CI), where mean quantum yields are presented with a 95% confidence interval. Stereo assignments are given for all structures.

**Fig. 7:**
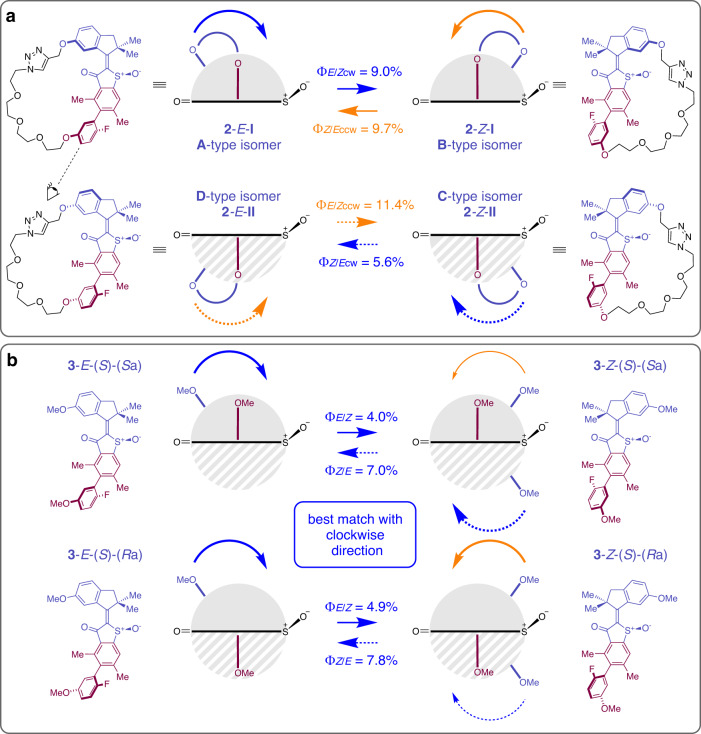
Quantitative comparison of photoisomerization quantum yields. Comparison of the photochemical behavior of restricted macrocycle **2** and molecular motor **3**. The indanone rotor and part of the benzothiophenone stator are colored purple and the biaryl moiety is colored dark red. Clockwise rotations and corresponding quantum yields are depicted by blue arrows and values and counterclockwise rotations are depicted in orange. **a** Because of the macrocyclization light-induced motions are restricted to half space **I** (grey half circle and solid arrows) or **II** (dashed grey half circle and dashed arrows) only in macrocycle **2**. Quantum yield values are corrected for the macrocyclization effect as shown in Fig. 6. **b** Quantum yields for *E* to *Z* and *Z* to *E* photoisomerization of **3**-(*S*)-(*S*_a_) (top) and **3**-(*S*)-(*R*_a_) (bottom). Comparison with macrocycle **2** reveal the theoretically predicted overall unidirectionality (blue arrows) for **3**-(*S*)-(*S*_a_) and **3**-(*S*)-(*R*_a_). A higher degree of unidirectionality is observed for **3**-(*S*)-(*S*_a_) as opposed to **3**-(*S*)-(*R*_a_), for which an increased backwards rotation takes place.

Thus, accounting for the macrocyclization effects, the final quantum yields for each rotation direction in macrocycle **2** are *Φ*_*E*/*Z*cw_ = 9.0% for **2**-*E*-**I** to **2**-*Z*-**I,** *Φ*_*E*/*Z*ccw_ = 11.4% for **2**-*E*-**II** to **2**-*Z*-**II,** *Φ*_*Z*/*E*ccw_ = 9.7% for **2**-*Z*-**I** to **2**-*E*-**I**, and *Φ*_*Z*/*E*cw_ = 5.6% for **2**-*Z*-**II** to **2**-*E*-**II**, respectively as shown in Fig. 7a.

For elucidation of directionality in the fast molecular motor setup **1** a molecular motor structure with closest similarity to the macrocyclic version **2** was needed. For this reason, the unrestricted molecular motor **3** was synthesized and investigated as representative of the motor class with core structure **1** (see Fig. 5c and the Supplementary Information for more details). Two different stable atropisomers of motor **3** can be isolated, each of which is predicted by theory to undergo a full four-step rotation with the same sense of directionality (see Fig. 3). However, a lesser degree of overall directionality is predicted for the (*R*_a_) configured derivative because of its smaller energy difference between isomers **B** and **C** and the resulting non-zero population of **B**. After synthesis the four diastereomers **3**-*E*-(*S*)-(*S*_a_), **3**-*Z*-(*S*)-(*S*_a_), **3**-*E*-(*S*)-(*R*_a_), and **3**-*Z*-(*S*)-(*R*_a_) could be separated. To elucidate the relative atropisomerism with respect to the sulfoxide stereocenter the structure of enantiomerically pure **3**-*E*-(*R*)-(*R*_a_) could be obtained in the crystalline state (Fig. 5c). With this structure all measured spectra and quantum yields could then be assigned unambiguously to one of the four isomers (see for example Fig. 5b for the corresponding UV-Vis spectra). Since helicity is also highly dynamic in the derivatives of **3**, only isomers of *E* or *Z* configuration can be distinguished in their behavior. Comparison of the photoisomerization quantum yields of **3** with the corresponding ones of macrocycle **2** would therefore directly report on the molecular half-space in which this photoisomerization occurs. If *E* to *Z* and *Z* to *E* photoisomerizations indeed occur in opposite half-spaces a full directional cycle must be taking place in the unbiased structures of **3**.

Quantum yields for the *E* to *Z* and for the *Z* to *E* photoisomerization of motor **3** were measured separately for each atropisomer **3**-(*S*)-(*S*_a_) and **3**-(*S*)-(*R*_a_) (Fig. 7b). Quantum yields for the *E* to *Z* direction were measured to be *Φ*_*E*/*Z*_ = 4.0% for **3**-(*S*)-(*S*_a_) and *Φ*_*E*/*Z*_ = 4.9% for **3**-(*S*)-(*R*_a_). Quantum yields for the *Z* to *E* direction were measured to be *Φ*_*Z*/*E*_ = 7.0% for **3**-(*S*)-(*S*_a_) and *Φ*_*Z*/*E*_ = 7.8% for **3**-(*S*)-(*R*_a_). There are similar yet small differences in the quantum yields for photoisomerization between the two atropisomers of **3**, which are somewhat larger for **3**-(*S*)-(*R*_a_) as compared to **3**-(*S*)-(*S*_a_).

When comparing the individual values with the corresponding corrected quantum yields of the macrocyclic system **2** clear assignments can be made. The measured and corrected quantum yield values for **2** and **3** are summarized in Table 1.

The measured quantum yields of the *E* to *Z* photoisomerizations *Φ*_*E*/*Z*_ = 4.0% for **3**-(*S*)-(*S*_a_) and *Φ*_*E*/*Z*_ = 4.9% for **3**-(*S*)-(*R*_a_) are 1. smaller than both *Φ*_*E*/*Z*_ quantum yields measured for macrocycle **2**, and 2. are closest to the *Φ*_*E*/*Z*cw_ = 9.0% quantum yield of **2**, which is associated with the expected clockwise rotation. They thus match far less with the significantly larger value of *Φ*_*E*/*Z*ccw_ = 11.4% associated with the corresponding counterclockwise rotation in **2**. For the *Z* to *E* photoisomerizations the measured values of *Φ*_*Z*/*E*_ = 7.0% for **3**-(*S*)-(*S*_a_) and *Φ*_*Z*/*E*_ = 7.8% for **3**-(*S*)-(*R*_a_) are falling in the middle between the two values measured for the macrocyclic system, which already hints at a reduced degree of directionality for the *Z* to *E* isomerization. For **3**-(*S*)-(*S*_a_) a better match is observed with the *Φ*_*Z*/*E*cw_ = 5.6% value associated with the expected clockwise rotation direction in macrocyclic **2**. For **3**-(*S*)-(*R*_a_) the 7.8% value is closer to the *Φ*_*Z*/*E*ccw_ = 9.7% value associated with the counterclockwise rotation in macrocyclic **2**. Taken together, the measured quantum yields for **3** show a clear directionality for the *E* to *Z* isomerization corresponding to the theoretically suggested clockwise direction because of the unambiguous quantum yield matches with the smaller corresponding macrocycle quantum yield. For the *Z* to *E* isomerization the situation is less clear-cut because the quantum yields of **3** are lying in the middle of the two corresponding macrocycle **2** values. This shows that there is no strongly preferred directionality for the *Z* to *E* isomerization and both rotation directions are present to a significant degree. However, two important points need to be made. First, overall directionality will be defined by the large directionality preference for the *E* to *Z* isomerization even in the case that *Z* to *E* isomerization possesses equal probability in either direction (e.g. consider 100% directionality for *E* to *Z* isomerization and no, i.e. 50%, directionality for the following *Z* to *E* isomerization. From the 100% molecules rotating in one direction in the first 180° rotation, 50% would go on to continue rotation in the same direction for the next 180°, leading to an overall 50% directionality of the motor). Second, for **3**-(*S*)-(*S*_a_) the preference for the *Z* to *E* isomerization is also clockwise like the corresponding *E* to *Z* isomerization, which makes this a fully directional motor for both 180° rotations as predicted by theory. Derivative **3**-(*S*)-(*R*_a_) represents a motor as well, however with diminished directionality overall and slightly preferred backwards rotation for the *Z* to *E* isomerization. These two overall behaviors are well predicted by the theoretical description of **3** (Fig. 3). In particular, theory predicts not only a less complete ratcheting THI of the *Z* isomers as compared to the *E* isomers. It also predicts that THI is less complete for the **3-**(*S*)-(*R*_a_) isomer as opposed to the **3**-(*S*)-(*S*_a_) isomer, leading to a greater absolute degree of directionality in the latter. The quantum yield comparison thus straight-forwardly establishes unidirectional rotation in the very fast derivatives **3**-(*S*)-(*S*_a_) and **3**-(*S*)-(*R*_a_) as representatives for very fast molecular motors with the core structure of **1**. The method even distinguishes relative differences in degree of directionality in both derivatives **3**-(*S*)-(*S*_a_) and **3**-(*S*)-(*R*_a_).

In conclusion, we present in this work a photochemical approach to experimentally evidence the directionality of very fast molecular motors. A prospective ultrafast version of HTI-based molecular motor is restricted in its motions inside a macrocyclic ring structure. Because of strong steric hindrance within the macrocycle, the light-induced motor rotations are now constrained to two separate **~**180° rotations. The quantum yields for the individual clockwise and counterclockwise motions of each **~**180° rotation can then be measured allowing a full quantification of all possible light-induced rotation directions. Comparison with the photoisomerization quantum yields measured for the corresponding unrestricted motor and accounting for the macrocyclization effect then allows to clarify its directionality. In cases where quantum yield differences for clockwise and counterclockwise rotations are large a semi-quantitative high or low degree of unidirectionality can be discerned from the extent of matching numbers in the comparison with the untethered system by this method. Otherwise, as a minimum the preferred directionality can be evidenced and thus the distinction between a true motor and a switch can be made. This method can be used for any very fast molecular motor type to directly prove its unidirectionality even in cases where no metastable intermediates are formed during rotation.

## Methods

### Synthesis

Macrocyclic HTI **2** was synthesized from commercially available 6-hydroxy-1-indanone, tetraethylene glycol, 3-hydroxyphenylboronic acid pinacol ester and 4-bromo-3,5-dimethylphenol in 14 steps. Non-macrocyclic HTI **3** was synthesized from commercially available 6-hydroxy-1-indanone, 3-hydroxyphenylboronic acid pinacol ester and 4-bromo-3,5-dimethylphenol in five steps. All synthetic procedures and characterizations are described in detail in the Supplementary Information. Reagents and solvents were obtained from abcr, Acros Organics, Fluka, Merck, Sigma-Aldrich or TCI in the qualities puriss., p.a., or purum and used as received. Technical solvents were distilled prior to use for column chromatography and extraction on a rotary evaporator (Heidolph Laborota 4000, 4001 and Vacuubrand CVC 3000). Reactions were monitored on Merck Silica 60 F-254 TLC plates and detection was done by irradiation with UV light (254 nm or 366 nm) to determine retardation factors (R_f_). Flash column chromatography was performed on silica gel (Merck, particle size 0.040–0.200 mm, ACROS, 0.035–0.070 mm or Machery-Nagel, particle size 0.040–0.063 mm) using distilled technical solvents. Automated medium pressure liquid chromatography (MPLC) was performed on Biotage Isolera One or Biotage Selekt machines using pre-packed silica columns from Biotage or Macherey-Nagel. High performance liquid chromatography (HPLC) was performed on a Shimadzu HPLC system consisting of a LC-20AP solvent delivery module, a CTO-20A column oven, a SPD-M20A photodiode array UV/Vis detector, and a CBM-20A system controller using a semi preparative CHIRALPAK® IC column (particle size 5 µm) from Daicel and HPLC grade solvents (EtOAc, *n*-hexane, and *i*-PrOH) from Sigma-Aldrich, VWR and ROTH. ^1^H NMR and ^13^C NMR spectra were measured on a JEOL ECX 400 (400 MHz), Bruker AVANCE III HD 400 (400 MHz), Varian VNMRS 400 (400 MHz), Varian VNMRS 600 (600 MHz), or Bruker AVANCE III HD 800 (800 MHz) NMR spectrometer at 298 K unless the temperature is indicated otherwise. Deuterated solvents were obtained from Cambridge Isotope Laboratories, Deutero GmbH, or Eurisotop and used without further purification. Chemical shifts (*δ*) are given relative to tetramethylsilane as an external standard. Residual solvent signals in the ^1^H and ^13^C NMR spectra were used as internal reference. CDCl_3_: *δ*_*H*_ = 7.260 ppm, *δ*_*C*_ = 77.160 ppm; CD_2_Cl_2_: *δ*_*H*_ = 5.320 ppm, *δ*_*C*_ = 54.000 ppm. Resonance multiplicity is indicated as s (singlet), d (doublet), t (triplet), q (quartet), and m (multiplet). Chemical shifts are given in parts per million (ppm) on the delta scale (*δ*) and the coupling constant values (*J*) in Hertz (Hz). Electron impact (EI) and atmospheric pressure photoionization (APPI) mass spectra were measured on a Finnigan MAT95Q or a Finnigan MAT90 and a MicrOTOF II spectrometer respectively. Signals are reported in *m*/*z* units and the molecular ion is assigned as M. UV/Vis absorption spectra were measured on a Varian Cary 5000 or on an Agilent Cary 60 spectrophotometer. The spectra were recorded in quartz cuvettes (1 cm). Spectral grade solvents were obtained from VWR and Merck. Absorption wavelengths (*λ*) are reported in nm and the molar attenuation coefficients (*ε*) in L mol^–1^ cm^–1^. Infrared spectra (ATR) were recorded on a Nicolet iS5, iD7 ATR spectrometer. Transmittance values are qualitatively described by wavenumber (cm^–1^) as strong (s), medium (m), weak (w). Photoisomerization experiments were conducted with continuous irradiation of the solutions in NMR tubes in CD_2_Cl_2_ or C_2_D_2_Cl_2_. Irradiations at 23 °C were conducted using LEDs from Thorlabs GmbH and Roithner Lasertechnik GmbH. X-ray crystallographic analysis of yv042 was performed on a Bruker D8Venture TXS using molybdenum-Kα-radiation or on a SuperNova, Dual, Cu at home/near, Atlas diffractometer. Single crystal structures were solved using Olex2. The X-ray intensity data of xv666, xv772 and xv804 were measured on a Bruker D8 Venture TXS system equipped with a multilayer mirror monochromator and a Mo Kα rotating anode X-ray tube (*λ* = 0.71073 Å). The frames were integrated with the Bruker SAINT software package^56^. Data were corrected for absorption effects using the Multi-Scan method (SADABS)^57^. The structures were solved and refined using the Bruker SHELXTL Software Package^58^.

### Thermal isomerizations

Thermal first-order isomerization processes were observed between **2**-*E*-**I** and **2**-*E*-**II,** **2**-*E*-**I** and **2**-*Z*-**I**, as well as **2**-*E*-**II** and **2**-*Z*-**II**, which proceed until a dynamic equilibrium is reached. This process is exemplarily described for the thermal isomerization between **2**-*E*-**I** and **2**-*Z*-**I** in the following. The observed decay is a combination of the rate constant for the forward process $k\left({{{{{\bf{2}}}}}}-E-{{{{{\bf{I}}}}}}\to {{{{{\bf{2}}}}}}-Z-{{{{{\bf{I}}}}}}\right)$ and the rate constant for the backwards isomerization $k\left({{{{{\bf{2}}}}}}-Z-{{{{{\bf{I}}}}}}\to {{{{{\bf{2}}}}}}-E-{{{{{\bf{I}}}}}}\right)$. This relationship can be fitted with a linear regression analysis using values for the initial concentration of ${{{{{\bf{2}}}}}}-E-{{{{{\bf{I}}}}}}$ $c\left({{{{{{\bf{2}}}}}}-E-{{{{{\bf{I}}}}}}}_{0}\right)$, the concentration of ${{{{{\bf{2}}}}}}-E-{{{{{\bf{I}}}}}}$ at equilibrium $c\left({{{{{{\bf{2}}}}}}-E-{{{{{\bf{I}}}}}}}_{{eq}}\right)$ and the concentration of ${{{{{\bf{2}}}}}}-E-{{{{{\bf{I}}}}}}$ at the elapsed time *t* $c\left({{{{{{\bf{2}}}}}}-E-{{{{{\bf{I}}}}}}}_{t}\right)$. The slope from the linear fit of the logarithmic plot *m* can then be used to determine the rate constant $k\left({{{{{\bf{2}}}}}}-E-{{{{{\bf{I}}}}}}\to {{{{{\bf{2}}}}}}-Z-{{{{{\bf{I}}}}}}\right)$ taking the law of mass action into account. With the rate constant $k\left({{{{{\bf{2}}}}}}-E-{{{{{\bf{I}}}}}}\to {{{{{\bf{2}}}}}}-Z-{{{{{\bf{I}}}}}}\right)$ at hand, the Gibbs free energy of activation Δ*G*^‡^ can be calculated for the thermal ${{{{{\bf{2}}}}}}-$*E-***I** to ${{{{{\bf{2}}}}}}-$*Z*-**I** isomerization, using the Eyring equation. From the stationary equilibrium composition, the relative energy difference between the two isomers ${{{{{\bf{2}}}}}}-E-{{{{{\bf{I}}}}}}$ and ${{{{{\bf{2}}}}}}-Z-{{{{{\bf{I}}}}}}$ can then be determined. The required equations, obtained experimental data, resulting Gibbs free energy differences and, Gibbs free energies of activation can all be found in the Supplementary Information, more specifically in Supplementary Eqs. 1–6, Supplementary Figs. 46–57, and Supplementary Tables 1–2.

### Quantum yield measurements

All quantum yield measurements were carried out using an instrumental setup from the Riedle group^59^. The procedure involved filling a quartz cuvette of 1 cm path length with 2 mL of CH_2_Cl_2_ for which the illumination power ${P}_{{{{{{\rm{ill}}}}}}}$ was measured. The employed setup examines entire photoisomerization kinetics of a particular system until reaching the photostationary state to obtain the forwards and backwards quantum yields of a two-component system in one measurement. Therefore, pure and/or enriched macrocycles **2**-*E*-**I**, **2**-*E*-**II**, **3**-*E*-(*S*)-(*R*_a_), **3**-*E*-(*S*)-(*S*_a_), and **3**-*Z*-(*S*)-(*R*_a_) motors, cyclic **4**-*Z*-(*R*_a_)/(*S*_a_), HTI **5**-*E*-(*R*_a_)/(*S*_a_), and **5**-*Z*-(*R*_a_)/(*S*_a_) were measured to deliver quantum yields for all isomers. A total volume of 2 mL of the corresponding CH_2_Cl_2_ solutions of each compound was filled into the cuvette and the absorption was adjusted to 0.6 – 1.7 a.u. at the particular irradiation wavelength. Whereas the solution of macrocycle **2** and motor **3** were irradiated with a 400 nm LED, a 450 nm LED was used for the irradiation of cyclic **4** and HTI **5** in defined time intervals. After each irradiation step, the power of the solar cell detector was recorded and an UV-Vis absorption spectrum was measured. The change in concentration, and hence the number of photoisomerized molecules was calculated from the predetermined molar extinction coefficients. The required equations, obtained experimental data, resulting quantum yield values, and error analysis can be found in the Supplementary Information, more specifically in Supplementary Eqs. 7–10, Supplementary Figs. 58–92, and Supplementary Tables 3–7.

### Theoretical description

All DFT calculations were performed using the Gaussian program package^60^. Ground state and transition state geometries were optimized at 25 °C on the B3LYP/6-311 G(d,p) IEFPCM (CH_2_Cl_2_) level of theory. The details of the theoretical description are given in the Supplementary Information, more specifically in Supplementary Tables 8–15.

## Supplementary information

Supplementary Information

Description of Additional Supplementary Files

Supplementary Movie 1

## Data Availability

The data generated in this study are provided in the Supplementary Information file. Additional data generated during this study are available from the corresponding author H. D. upon request. The X-ray crystallographic coordinates for the structures **2**-*Z*-**I**, **2**-*E*-**II**, **2**-*Z*-**II** and **3**-*E*-**I**-(*R*_a_) reported in this study have been deposited at the Cambridge Crystallographic Data Centre (CCDC), under CCDC numbers 2154099 (**2**-*Z*-**I**), 2154098 (**2**-*E*-**II**), 2154100 (**2**-*Z*-**II**), 2163097 (**3**-*E*-(*R*)-(*R*_a_)). These data can be obtained free of charge from the Cambridge Crystallographic Data Centre via www.ccdc.cam.ac.uk/data_request/cif. Source data are provided with this paper.

## Source data

Source Data
