# Supplementary material for: A photochemical method to evidence directional molecular motions
Source: Nat Commun. 2023 Jul 31;14:4595. doi: 10.1038/s41467-023-40190-4 (PMC10390485; doi:10.1038/s41467-023-40190-4)
Supplement: Supplementary file 1 — Supplementary Information [file 41467_2023_40190_MOESM1_ESM.pdf]

Supplementary Information

A Photochemical Method to Evidence Directional Molecular Motions

*Benjamin Lukas Regen-Pregizer, Ani Ozcelik, Peter Mayer, Frank Hampel, Henry Dube**

Table of Contents

SUPPLEMENTARY NOTE 1: MATERIALS AND METHODS 4

SUPPLEMENTARY NOTE 2: SYNTHESIS OF COMPOUNDS 6

SYNTHESIS OF MACROCYCLIC HTI 2 6

SYNTHESIS OF HTI 3 24

SUPPLEMENTARY NOTE 3: STRUCTURAL AND CONFORMATIONAL DESCRIPTION .. 35

OVERVIEW OF CONFORMATIONAL STATES FOR MACROCYCLIC HTI 2 35

OVERVIEW OF CONFORMATIONAL STATES FOR HTI 3 36

STRUCTURE OF MACROCYCLIC HTI 2 IN SOLUTION 37

Isomer 2-*E*-I 39

Isomer 2-*Z*-I 44

Isomer 2-*E*-II 49

Isomer 2-*Z*-II 55

STRUCTURE OF HTI 3 IN SOLUTION 61

Isomer 3-(*E*)-(*S*)-(S_a) 63

Isomer 3-(*Z*)-(*S*)-(S_a) 66

Isomer 3-(*E*)-(*S*)-(R_a) 69

Isomer 3-(*Z*)-(*S*)-(R_a) 72

STRUCTURES IN THE CRYSTALLINE STATE 75

Isomer 2-*Z*-I 75

Isomer 2-*E*-II 76

Isomer 2-*Z*-II 77

Isomer 3-*E*-(*R*)-(R_a) 78

SUPPLEMENTARY NOTE 4: BEHAVIOR AT ELEVATED TEMPERATURES 79

Isomer 2-*E*-I and 2-*E*-II 81

Isomer 2-*E*-I and 2-*Z*-I 85

Isomer 2-*E*-II and 2-*Z*-II 87

SUPPLEMENTARY NOTE 5: UV-VIS ABSORPTION SPECTRA 90

ISOMERS 2-*E*-I AND 2-*Z*-I 90

Isomers 2-*E*-II and 2-*Z*-II ... 91
Isomers 3-*E*-(*S*)-(S_A) and 3-*Z*-(*S*)-(S_A) ... 92
Isomers 3-*E*-(*S*)-(R_A) and 3-*Z*-(*S*)-(R_A) ... 93
Isomers 4-*E*-(R_A/S_A) and 4-*Z*-(R_A/S_A) ... 94
Isomers 5-*E*-(R_A/S_A) and 5-*Z*-(R_A/S_A) ... 95

SUPPLEMENTARY NOTE 6: QUANTUM YIELD DETERMINATION ... 96

Quantum Yield of 2-*E*-I and 2-*Z*-I ... 109
Quantum Yield of 2-*E*-II and 2-*Z*-II ... 110
Quantum Yield of 3-*E*-(*S*)-(S_A) and 3-*Z*-(*S*)-(S_A) ... 111
Quantum Yield of 3-*E*-(*S*)-(R_A) and 3-*Z*-(*S*)-(R_A) ... 112
Quantum Yield of 4-*Z*-(R_A/S_A) and 4-*E*-(R_A/S_A) ... 114
Quantum Yield of 5-*E*-(R_A/S_A) and 5-*Z*-(R_A/S_A) ... 115

SUPPLEMENTARY NOTE 7: THEORETICAL DESCRIPTION ... 117

Theoretical obtained Energies ... 117
Ground State Geometries ... 119

SUPPLEMENTARY NOTE 8: CRYSTAL STRUCTURAL DATA ... 131

SUPPLEMENTARY NOTE 9: NMR SPECTRA OF SYNTHESIZED COMPOUNDS ... 134

SUPPLEMENTARY REFERENCES ... 144

Supplementary Note 1: Materials and Methods

Reagents and solvents were obtained from abcr, Acros Organics, Fluka, Merck, Sigma-Aldrich or TCI in the qualities puriss., p.a., or purum and used as received. Technical solvents were distilled prior to use for column chromatography and extraction on a rotary evaporator (Heidolph Laborota 4000, 4001 and Vacuubrand CVC 3000). Reactions were monitored on Merck Silica 60 F-254 TLC plates and detection was done by irradiation with UV light (254 nm or 366 nm) to determine retardation factors (R_f).

Flash column chromatography was performed on silica gel (Merck, particle size 0.040–0.200 mm, ACROS, 0.035–0.070 mm or Machery-Nagel, particle size 0.040–0.063 mm) using distilled technical solvents. Automated medium pressure liquid chromatography (MPLC) was performed on Biotage Isolera One or Biotage Selekt machines using pre-packed silica columns from Biotage or Macherey-Nagel.

High performance liquid chromatography (HPLC) was performed on a Shimadzu HPLC system consisting of a LC-20AP solvent delivery module, a CTO-20A column oven, a SPD-M20A photodiode array UV/Vis detector, and a CBM-20A system controller using a semi preparative CHIRALPAK® IC column (particle size 5 µm) from Daicel and HPLC grade solvents (EtOAc and *n*-hexane) from Sigma-Aldrich, VWR and ROTH.

^{1}H NMR and ^{13}C NMR spectra were measured on a JEOL ECX 400 (400 MHz), Bruker AVANCE III HD 400 (400 MHz), Varian VNMRS 400 (400 MHz), Varian VNMRS 600 (600 MHz), or Bruker AVANCE III HD 800 (800 MHz) NMR spectrometer at 298 K unless the temperature is indicated otherwise. Deuterated solvents were obtained from Cambridge Isotope Laboratories, Deutero GmbH, or Eurisotop and used without further purification. Chemical shifts (δ) are given relative to tetramethylsilane as an external standard. Residual solvent signals in the ^{1}H and ^{13}C NMR spectra were used as internal reference. $CDCl_3$: δ_H = 7.260 ppm, δ_C = 77.160 ppm; CD_2Cl_2: δ_H = 5.320 ppm, δ_C = 54.000 ppm. Resonance multiplicity is indicated as s (singlet), d (doublet), t (triplet), q (quartet), and m (multiplet). Chemical shifts are given in parts per million (ppm) on the delta scale (δ) and the coupling constant values (*J*) in Hertz (Hz).

Electron impact (EI) and atmosperic pressure photoionization (APPI) mass spectra were measured on a Finnigan MAT95Q or a Finnigan MAT90 and a MicrOTOF II spectrometer respectively. Signals are reported in *m*/*z* units and the molecular ion is signed as M.

UV/Vis absorption spectra were measured on a Varian Cary 5000 or on an Agilent Cary 60 spectrophotometer. The spectra were recorded in quartz cuvettes (1 cm). Spectral grade solvents were obtained from VWR and Merck. Absorption wavelengths (λ) are reported in nm and the molar attenuation coefficients (ε) in L mol^{-1} cm^{-1}.

Infrared spectra (ATR) were recorded on a Nicolet iS5, iD7 ATR spectrometer. Transmittance values are qualitatively described by wavenumber (cm^{-1}) as strong (s), medium (m), weak (w).

Photoisomerization experiments were conducted with continuous irradiation of the solutions in NMR tubes in CD_2Cl_2 or $(CDCl_2)_2$. Irradiations at 23 °C were conducted using LEDs (505 nm or 565 nm) from Thorlabs GmbH and Roithner Lasertechnik GmbH.

Quantum yield measurements were performed in CH_2Cl_2 using an instrumental setup from the Riedle group[1] and samples were irradiated with a Luminus 400 nm or an Osram 450 nm LED.

X-ray crystallographic analysis was performed on a Bruker D8Venture TXS using molybdenum-Kα-radiation or on a SuperNova, Dual, Cu at home/near, Atlas diffractometer.

Supplementary Note 2: Synthesis of Compounds

Synthesis of Macrocyclic HTI 2

Macrocyclic HTI **2** was constructed from indanone **8**, tetraethylene glycol chain **13** and benzothiophenone **14**. These building blocks were combined, forming the macrocycle **2** as depicted in Supplementary Figure 1 starting from commercially available **6** and **9**. Building blocks **8** and **14** were synthesized according to a literature procedure[2] and building block **13** was accessed through a modified procedure thereof.

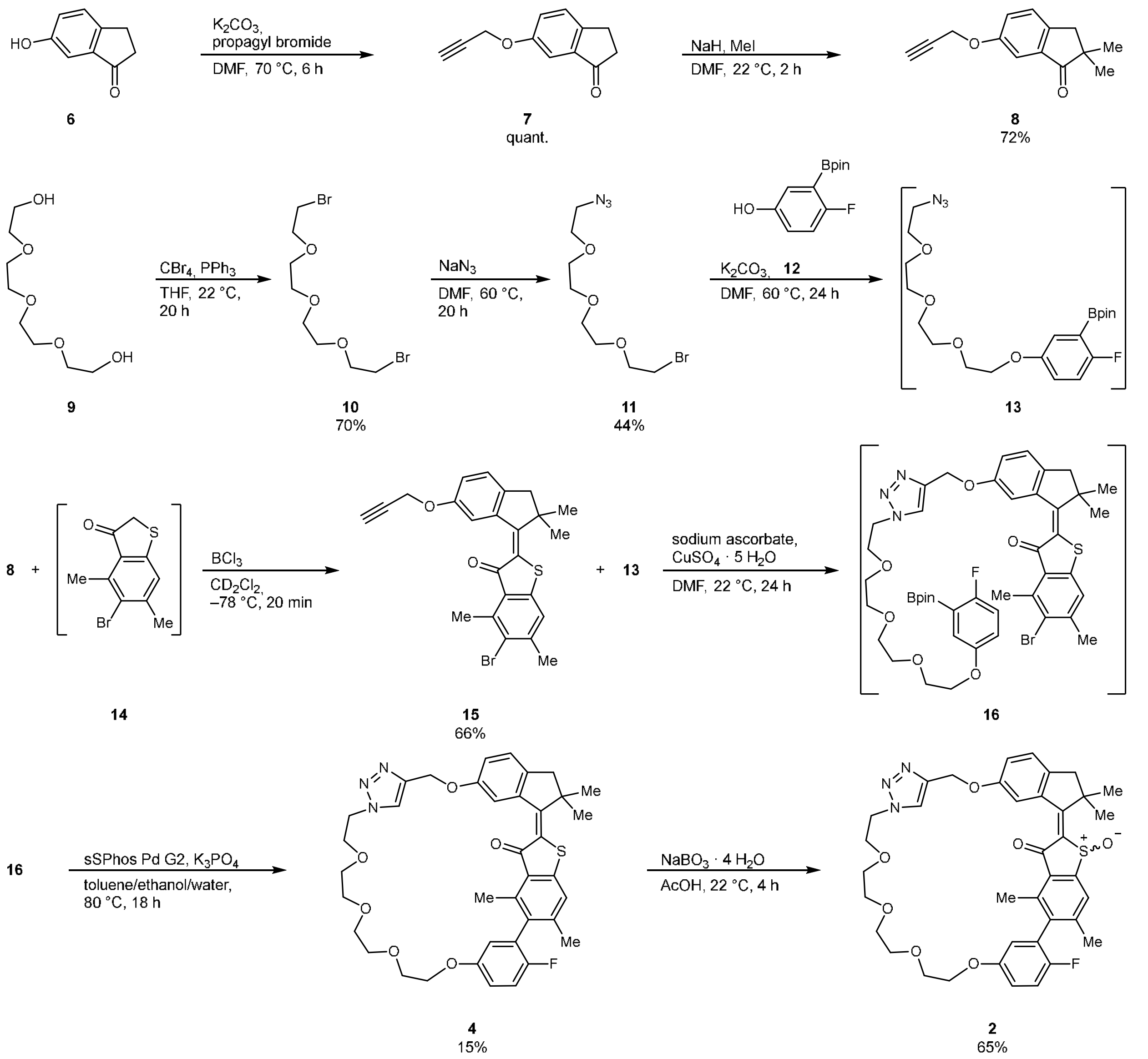

Supplementary Figure 1 Synthetic route for macrocyclic HTI 2. Building blocks **8**, **13** and **14** used for construction of macrocyclic HTI **2**.

6-(prop-2-yn-1-yloxy)-2,3-dihydro-1H-inden-1-one (7)

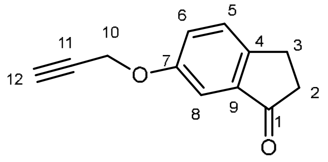

Commercially available 6-hydroxy-1-indanone **6** (909 mg, 6.13 mmol, 1.0 equiv.) was dissolved in DMF (41 mL, 0.15 M) together with potassium carbonate (3.39 g, 24.5 mmol, 4.0 equiv.) and propargyl bromide (0.7 mL, 80% in toluene, 6.13 mmol, 1.0 equiv.). After stirring for 6 h at 90 °C the reaction was stopped by pouring the mixture into a water (100 mL) filled separatory funnel. The aqueous phase was extracted three times with EtOAc (100 mL each) and the combined organic phases were washed four times with water (30 mL each to remove residual DMF) and brine (100 mL) and subsequently dried over anhydrous sodium sulfate. Removal of the solvent *in vacuo* gave crude compound **7** as a colorless liquid (1.12 g, 6.00 mmol, quantitative conversion), which was used for the next synthetic step without further purification.

^{1}H NMR (400 MHz, CD_2Cl_2): δ = 7.45 – 7.39 (m, 1H, H-C8), 7.28 – 7.20 (m, 2H, H-C5, H-C6), 4.74 (d, ^4J(H,H) = 2.4 Hz, 2H, H_2-C10), 3.12 – 3.03 (m, 2H, H_2-C3), 2.71 – 2.64 (m, 2H, H_2-C2), 2.58 (t, ^4J(H,H) = 2.4 Hz, 1H, H-C12) ppm.

^{13}C NMR (101 MHz, CD_2Cl_2): δ = 206.65 (C1), 157.61 (C7), 149.18 (C9), 138.70 (C4), 128.06 (C8), 124.36 (C6), 106.78 (C5), 78.58 (C11), 75.99 (C12), 56.55 (10), 37.36 (C2), 25.51 (C3) ppm.

HR-MS (EI^+, *m*/*z*) $[M]^+$ calc. for $[C_{12}H_{10}O_2]^+$: 186.0675, found: 186.0674.

IR: $\tilde{\nu}$ = 3922 (w), 3908 (w), 3259 (s), 3245 (s), 3060 (m), 2958 (w), 2924 (s), 2884 (w) 2860 (w), 2114 (s), 2040 (w), 1888 (m), 1796 (w), 1690 (s), 1613 (m), 1583 (w), 1485 (s), 1442 (s), 1397 (m), 1374 (m), 1325 (m), 1275 (s), 1242 (s), 1211 (s), 1167 (s), 1118 (w), 1018 (s), 925 (m), 903 (m), 882 (m), 829 (s), 769 (m), 710 (m), 696 (s), 668 (s), 629 (m), 590 (w), 556 (s), 498 (s), 462 (w) cm^{-1}.

TLC (SiO_2, *i*-Hex:EtOAc, 12:1 v/v): R_f = 0.10.

2,2-dimethyl-6-(prop-2-yn-1-yloxy)-2,3-dihydro-1H-inden-1-one (8)

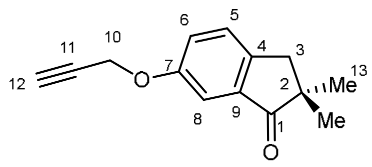

Sodium hydride (344 mg (60% in mineral oil), 8.41 mmol, 2.5 equiv.) was added to a solution of compound **7** (627 mg, 3.37 mmol, 1.0 equiv.) in DMF (6.0 mL, 0.6 M) at 0 °C and the resulting suspension was stirred for 20 min at 0 °C. Then methyl iodide (0.52 mL, 8.41 mmol, 2.5 equiv.) was added dropwise and the mixture was stirred for another 2 h at 22 °C. After addition of a saturated aqueous ammonium chloride solution (25 mL) and extracting the aqueous phase three times with EtOAc (50 mL each), the combined organic phases were washed four times with water (20 mL each to remove residual DMF), twice with brine (30 mL each), dried over anhydrous sodium sulfate, and the solvent was removed *in vacuo*. Purification of the residual crude product was achieved by flash column chromatography (SiO_2, *i*-Hex:EtOAc, 12:1 v/v), yielding compound **8** as a colorless liquid (521 mg, 2.43 mmol, 72%).

^{1}H NMR (400 MHz, CD_2Cl_2): δ = 7.41 – 7.33 (m, 1H, H-C5), 7.28 – 7.20 (m, 2H, H-C6, H-C8), 4.74 (d, ^{4}J(H,H) = 2.4 Hz, 2H, H_2-C10), 2.96 – 2.90 (m, 2H, H_2-C3), 2.59 (t, ^{4}J(H,H) = 2.4 Hz, 1H, H-C12), 1.20 (s, 6H, H_3-C13 and H_3-C13’) ppm.

^{13}C NMR (101 MHz, CD_2Cl_2): δ = 211.18 (C1), 157.68 (C7), 146.22 (C4), 136.90 (C9), 128.05 (C5), 124.61 (C6), 107.32 (C8), 78.59 (C11), 75.98 (C12), 56.50 (C10), 46.59 (C2), 42.46 (C3), 25.44 (C13) ppm.

HR-MS (EI^+, *m/z*) $[M]^+$ calc. for $[C_{14}H_{14}O_2]^+$: 214.0988, found: 214.0986.

IR: $\tilde{\nu}$ = 3367 (w), 3288 (s), 3259 (s), 3061 (w), 3960 (s), 2924 (s), 2866 (m), 2121 (m), 1702 (s), 1614 (m), 1586 (w), 1485 (s), 1444 (m), 1379 (m), 1329 (w), 1304 (s), 1275 (s), 1228 (s), 1182 (m), 1154 (m), 1102 (w), 1019 (s), 924 (m), 883 (w), 863 (w), 824 (m), 770 (m), 673 (s), 626 (s), 544 8m), 468 (m), 430 (m), 403 (m) cm^{-1}.

TLC (SiO_2, *i*-Hex:EtOAc, 12:1 v/v): R_f = 0.29.

(*E*/*Z*)-5-bromo-2-(2,2-dimethyl-6-(prop-2-yn-1-yloxy)-2,3-dihydro-1H-inden-1-ylidene)-4,6-dimethylbenzo[b]thiophen-3(2H)-one (15)

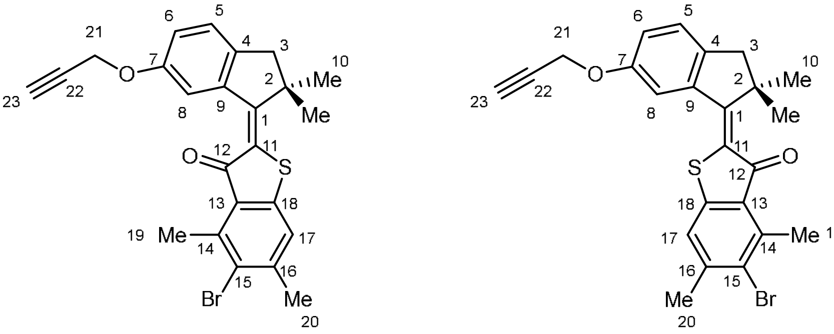

Two separate Schlenk flasks under inert N_2 gas atmosphere were used, one being charged with compound **8** (108 mg, 0.50 mmol, 1.0 equiv.) in anhydrous CH_2Cl_2 (0.3 mL, 1.67 M) at 0 °C and the other one with compound **14** (129 mg, 0.50 mmol, 1.0 equiv.) in anhydrous CH_2Cl_2 (1.5 mL, 0.33 M) at –78 °C. Boron trichloride (0.50 mL, 1 M in anhydrous CH_2Cl_2, 0.50 mmol, 1.0 equiv.) was added to the flask containing compound **14** and the resulting mixture was immediately taken up into the same syringe and transferred to the second flask containing compound **8** at 0 °C. The reaction mixture was vigorously stirred for 40 min while warming up to 22 °C. The reaction was stopped by pouring the mixture onto a saturated aqueous ammonium chloride solution (20 mL). Extraction of the aqueous phase was done four times with CH_2Cl_2 (40 mL each), the combined organic phases were dried over anhydrous sodium sulfate, and the solvent was removed *in vacuo*. The product was purified using flash column chromatography (SiO2, *i*-Hex:EtOAc, 50:1 v/v) yielding compound **15** as a red solid (150 mg, 0.33 mmol, 66%).

^{1}H NMR (600 MHz, CD_2Cl_2): δ = 7.68 (d, ^4J(H,H) = 2.3 Hz, 1H, H-C8), 7.25 (d, ^3J(H,H) = 8.2 Hz, 1H, H-C5), 7.23 (d, ^4J(H,H) = 1.0 Hz, 1H, H-C17), 7.05 (dd, ^3J(H,H) = 8.2 Hz, ^4J(H,H) = 2.3 Hz, 1H, H-C6), 4.78 (d, ^4J(H,H) = 2.4 Hz, 2H, H_2-C21), 3.01 (d, ^4J(H,H) = 1.0 Hz, 2H, H_2-C3), 2.85 (s, 3H, H_3-C19), 2.63 (t, ^4J(H,H) = 2.4 Hz, 1H, H-C23), 2.50 (d, ^4J(H,H) = 0.8 Hz, 3H, H_3-C20), 1.61 (s, 6H, H_3-C10 and H_3-C10') ppm.

^{13}C NMR (151 MHz, CD_2Cl_2): δ = 188.22 (C12), 162.57 (C4), 157.25 (C7), 145.04 (C16), 144.75 (C11), 143.32 (C9), 141.68 (C1), 141.49 (C13), 128.35 (C14), 126.65 (C15), 126.25 (C5), 126.19 (C18), 122.81 (C17), 119.21 (C6), 113.70 (C8), 78.91 (C22), 75.95 (C23), 56.72 (C21), 50.89 (C3) 49.51 (C2), 25.70 (C10), 25.31 (C20), 18.12 (C19) ppm.

HR-MS (EI$^+$, *m*/*z*) [M]$^+$ calc. for $[C_{24}H_{21}{}^{79}BrO_2S]^+$: 452.0440, found: 452.0443.

IR: $\tilde{\nu}$ = 3292 (w), 3280 (s), 3265 (s), 2960 (m), 2923 (m), 2176 (w) 2157 (m), 2034 (w), 1998 (w), 1968 (w), 1761 (w), 1707 (s), 1487 (s), 1445 (m), 1380 (w), 1306 (w), 1261 (s), 1236 (s), 1102 (m), 1026 (s), 803 (m) cm^{-1}.

TLC (SiO_2, *i*-Hex:EtOAc, 50:1 v/v): R_f = 0.36 and 0.47.

1-bromo-2-(2-(2-(2-bromoethoxy)ethoxy)ethoxy)ethane (10)

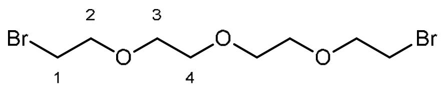

A mixture of commercially available tetraethylene glycol **9** (5.63 mL, 25.7 mmol, 1.0 equiv.) and tetra-bromomethane (18.8 g, 56.6 mmol, 2.2 equiv.) in THF (30 mL, 1.2 M) solution was cooled to 0 °C. Then triphenylphosphine (13.5 g, 54.0 mmol, 2.1 equiv.) was added slowly and the resulting mixture was allowed to warm up to 22 °C and was stirred at this temperature for 21 h. The reaction was stopped by pouring the mixture onto 200 mL of a saturated aqueous ammonium chloride solution. The aqueous phase was extracted three times with EtOAc (200 mL each). The combined organic phases were washed with brine (100 mL), dried over anhydrous sodium sulfate, and the solvent was removed *in vacuo*. Compound **10** was purified by flash column chromatography (SiO_2, *i*-Hex:EtOAc, 3:1 v/v) and was obtained as a colorless oil (5.77 g, 18.1 mmol, 70%).

^{1}H NMR (400 MHz, CD_2Cl_2): δ = 3.79 (t, ^3J(H,H) = 6.1 Hz, 4H, H_2-C2), 3.66 – 3.60 (m, 8H, H_2-C3, H_2-C4), 3.49 (t, ^3J(H,H) = 6.1 Hz, 4H, H_2-C1) ppm.

^{13}C NMR (101 MHz, CD_2Cl_2): δ = 71.56 (C2), 70.97 (C4), 70.91 (C3), 31.27 (C1) ppm.

HR-MS ($APPI^+$, *m/z*) $[M+H]^+$ calc. for $[C_8H_{17}{}^{79}Br_2O_3]^+$: 318.9539, found: 318.9538.

IR: $\tilde{\nu}$ = 2866 (s), 1735 (w), 1455 (m), 1422 (m), 1350 (s), 1325 (w), 1276 (m), 1247 (m), 1226 (m), 1100 (s), 1040 (m), 1020 (m), 998 (m), 953 (m), 932 (m), 878 (m), 822 (m), 800 (s), 662 (s), 570 (s), 467 (s) cm^{-1}.

TLC (SiO_2, *i*-Hex:EtOAc, 3:1 v/v): R_f = 0.33.

1-azido-2-(2-(2-(2-bromoethoxy)ethoxy)ethoxy)ethane (11)

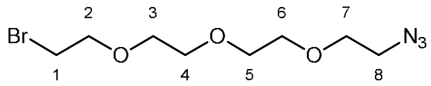

A solution of compound **10** (2.05 g, 6.25 mmol, 1.0 equiv.) and sodium azide (262 mg, 1.87 mmol, 0.6 equiv.) in DMF (4.2 mL,1.49 M) was stirred for 21 h at 60 °C. The reaction mixture was added to a separatory funnel charged with water (100 mL), extracted three times with EtOAc (100 mL each), and the combined organic phases were washed four times with water (50 mL each) to remove residual DMF. After washing the organic phase with brine (100 mL), drying over anhydrous sodium sulfate compound, and solvent removal *in vacuo*, compound **11** was purified by flash column chromatography (SiO_2, *i*Hex:EtOAc, 4:1 v/v) and was obtained as a colorless oil (386 mg, 1.37 mmol, 44%).

^{1}H NMR (400 MHz, CD_2Cl_2): δ = 3.79 (t, ^3J(H,H) = 6.1 Hz, 2H, H_2-C2), 3.67 – 3.60 (m, 10H, H_2-C3, H_2-C4, H_2-C5, H_2-C6, H_2-C7), 3.49 (t, ^3J(H,H) = 6.1 Hz, 2H, H_2-C1), 3.38 (t, ^3J(H,H) = 5.0 Hz, 2H, H_2-C8) ppm.

^{13}C NMR (101 MHz, CD_2Cl_2): δ = 71.52 (C2), 71.02 (C4, C5 or C6), 70.98 (C4, C5 or C6), 70.88 (C4, C5 or C6), 70.31 (C3), 70.31 (C7), 51.23 (C8), 31.28 (C1) ppm.

HR-MS (APPI$^+$, *m*/*z*) $[M+H]^+$ calc. for $[C_8H_{17}BrN_3O_3]^+$: 282.0448, found 282.0449.

IR: $\tilde{\nu}$ = 2953 (s), 2924 (s), 2854 (s), 2362 (w), 2189 (w), 2112 (s), 2098 (s), 1742 (m), 1736 (m), 1458 (m), 1444 (m), 1375 (s), 1359 (s), 1276 (m), 1242 (m), 1122 (s), 821 (w) cm^{-1}.

TLC (SiO_2, *i*-Hex:EtOAc, 4:1 v/v): R_f = 0.18.

2-(5-(2-(2-(2-(2-azidoethoxy)ethoxy)ethoxy)ethoxy)-2-fluorophenyl)-4,4,5,5-tetramethyl-1,3,2-dioxaborolane (13)

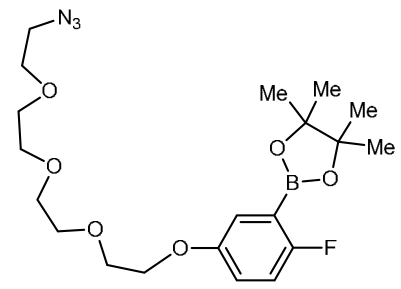

Compound **11** (547 mg, 2.31 mmol, 1.0 equiv.), commercially available 2-fluoro-5-hydroxyphenylboronic acid pinacol ester **12** (543 mg, 2.31 mmol, 1.2 equiv.), and potassium carbonate (585 mg, 8.8 mmol, 4.0 equiv.) were dissolved in DMF (7.5 mL, 0.31 M). The suspension was heated to 60 °C for 23 h and the reaction was stopped by pouring the mixture onto a saturated aqueous sodium bicarbonate solution (100 mL). The aqueous phase was extracted three times with EtOAc (100 mL each). The combined organic phases were washed four times with water (50 mL each to remove remaining DMF) and then with brine (50 mL) and dried over anhydrous sodium sulfate. The volatiles were removed *in vacuo* and compound **13** was directly utilized as crude product without further purification in the following synthetic transformation.

HR-MS (APPI$^+$, *m/z*) $[M+H]^+$ calc. for $[C_{20}H_{32}{}^{79}BFN_3O_6]^+$: 440.2363, found: 440.2364.

(*E*/*Z*)-5-bromo-2-(6-((1-(2-(2-(2-(2-(4-fluoro-3-(4,4,5,5-tetramethyl-1,3,2-dioxaborolan-2-yl)phenoxy)ethoxy)ethoxy)ethoxy)ethyl)-1H-1,2,3-triazol-4-yl)methoxy)-2,2-dimethyl-2,3-dihydro-1H-inden-1-ylidene)-4,6-dimethylbenzo[b]thiophen-3(2H)-one (16)

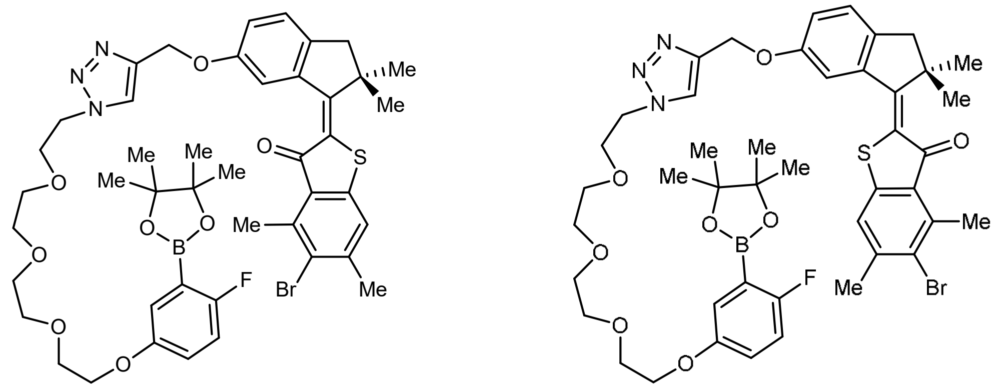

A mixture of HTI **15** (329 mg, 0.73 mmol, 1.0 equiv.), compound **13** (340 mg, 0.77 mmol, 1.05 equiv), sodium ascorbate (19.8 mg, 0.10 mmol, 14 mol%) and copper(II) sulfate pentahydrate (5.1 mg, 0.02 mmol, 3 mol%) were dissolved in DMF (3.0 mL, 1.4 M) and the resulting mixture was stirred for 1 d at 23 °C. After quenching the reaction by pouring the mixture onto a saturated aqueous ammonium chloride solution (75 mL) followed by extraction of the aqueous phase three times with EtOAc (75 mL each), the combined organic phases were then washed four times with water (30 mL to remove residual DMF) and brine (75 mL) and then dried over anhydrous sodium sulfate. The volatiles were removed *in vacuo* and compound **16** was obtained after flash column chromatography (SiO_2, CD_2Cl_2:MeOH, 100:0 to 97:3 v/v) step as a viscous red oil and used without further purification.

HR-MS (APPI$^+$, *m*/*z*) $[M+H]^+$ calc. for $[C_{44}H_{53}B^{79}BrFN_3O_8S]^+$: 892.2808, found: 892.2817.

TLC (SiO_2, CD_2Cl_2:MeOH, 99:1 v/v): R_f = 0.04 and 0.20.

(*E*/*Z*)-16-fluoro-24,26,32,32-tetramethyl-22,23,32,33-tetrahydro-31H,61H-4,9,12,15,18-pentaoxa-6(4,1)-triazola-2(5,2)-benzo[b]thiophena-3(1,6)-indena-1(1,3)-benzenacyclooctadecaphan-23-one (4)

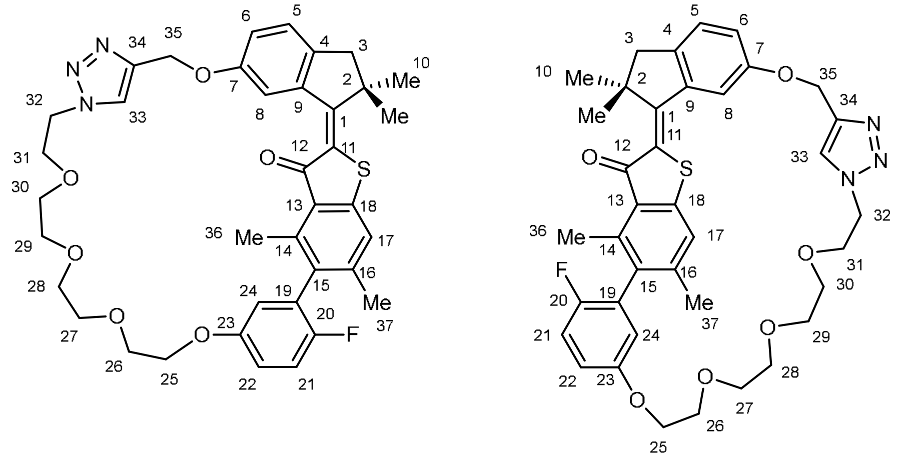

To a dried Schlenk tube compound **16** (424 mg, 0.72 mmol, 1.0 equiv.) and potassium phosphate (465 mg, 2.16 mmol, 3.0 equiv.) were added under inert conditions. After dissolving in a mixture of toluene (13.4 mL), ethanol (13.4 mL), and water (4.5 mL) the reaction mixture (0.023 M) was degassed *via* three freeze-pump-thaw cycles and then sSPhos Pd G2 (48.0 mg, 0.06 mmol, 8 mol%) was added. The reaction mixture was heated at 80 °C for 24 h. Then the reaction was stopped by addition of a saturated aqueous ammonium chloride solution at 22 °C and the aqueous phase was extracted three times with EtOAc (50 mL each). The combined organic phases were dried over anhydrous sodium sulfate and removed *in vacuo* and purified using flash column chromatography (SiO_2, CH_2Cl_2:MeOH, 50:1 v/v) giving compound **4** as an orange solid (74.6 mg, 0.11 mmol, 15%). Four isomers (two atropisomers for each *E*- and *Z*-isomer) were obtained from HPLC separation using a preparative *Daicel* CHIRALPAK® ID column (*n*-hexane:EtOAc, 50:50 to 0:100 v/v, at 40°C).

***E*-Isomer:**

^{1}H NMR (600 MHz, CD_2Cl_2): δ = 8.47 (d, ^3J(H,H) = 2.5 Hz, 1H, H-C8), 7.25 (s, 1H, H-C17), 7.23 (s, 1H, H-C33), 7.18 (dd, ^3J(H,H) = 8.3 Hz, ^5J(H,H) = 0.6 Hz, 1H, H-C5), 7.11 (t, ^3J(H,H) = 8.8 Hz, 1H, H-C21), 6.95 (d, ^3J(H,H) = 2.4 Hz, 1H, H-C6), 6.94 – 6.90 (m, 1H, H-C22), 6.65 (ddd, J(H,H) = 5.8 , 3.2, 1.6 Hz, 1H, H-C24), 5.23 – 5.18 (m, 2H, H-C35), 4.58 – 4.51 (m, 1H, H-C32), 4.46 (m, 1H, H-C32), 4.26

– 4.21 (m, 1H, H-C30, H-C29, H-C28, H-C27, H-C26 or H-C25), 4.05 – 4.01 (m, 1H, H-C30, H-C29, H-C28, H-C27, H-C26 or H-C25), 3.88 – 3.82 (m, 1H, H-C31), 3.78 – 3.72 (m, 1H, H-C31), 3.65 – 3.56 (m, 6H, H_2-C30, H_2-C29, H_2-C28, H_2-C27, H_2-C26 or H_2-C25), 3.54 – 3.49 (m, 2H, H_2-C30, H_2-C29, H_2-C28, H_2-C27, H_2-C26 or H_2-C25), 3.28 – 3.22 (m, 1H, H-C30, H-C29, H-C28, H-C27, H-C26 or H-C25), 3.21 – 3.15 (m, 1H, H-C30, H-C29, H-C28, H-C27, H-C26 or H-C25), 3.00 (d, ^2J(H,H) = 1.4 Hz, 1H, H-C3), 2.93 (d, ^2J(H,H) = 3.1 Hz, 1H, H‘-C), 2.47 (s, 3H, H_3-C36), 2.13 – 2.11 (m, 3H, H_3-C37), 1.52 (s, 3H, H-C10), 1.50 (s, 3H, H_3-C10‘) ppm.

^{13}C NMR (151 MHz, CD_2Cl_2): δ = 189.23 (C12), 161.55 (C1), 158.19 (C9), 156.55 (C7) 155.52 (C23), 154.51 (d, ^1J(C,F) = 233.2 Hz, C20), 143.77 (C34) 140.95 (C4), 140.50 (C14), 134.19 (C16), 133.85 (C15), 127.78 (C13), 127.63 (C11), 127.62 (d, ^2J(C,F) = 13.3 Hz, C19), 127.44 (C18), 125.44 (C5), 121.96 (C17), 121.93 (C33), 118.84 (C6), 116.65 (d, ^3J(C,F) = 19.7 Hz, C21), 116.56 (C24), 114.74 (C22), 112.85 (C8), 71.74 (C30, C29, C28, C27, C26 or C25), 71.38 (C30, C29, C28, C27, C26 or C25), 71.21 (C30, C29, C28, C27, C26 or C25), 71.05 (C32), 70.90 (C30, C29, C28, C27, C26 or C25), 70.82 (C30, C29, C28, C27, C26 or C25), 69.88 (C31), 65.70 (C30, C29, C28, C27, C26 or C25), 61.85 (C35), 50.90 (C3), 49.57 (C2), 26.76 (C10), 26.72 (C10‘), 21.70 (C36), 15.96 (C37) ppm.

HR-MS (APPI$^+$, *m/z*) $[M+H]^+$ calc. for $[C_{38}H_{41}FN_3O_6S]^+$: 686.2695, found: 686.2698.

IR: $\tilde{\nu}$ = 2959 (s), 2932 (s), 2850 (s), 2362 (w), 2327 (w), 2152 (w), 2093 (w), 1673 (m), 1606 (w), 1581 (w), 1495 (w), 1480 (m), 1459 (m), 1343 (m), 1307 (m), 1206 (s), 1007 (w), 1072 (m), 1015 (m), 919 (m), 777 (m), 733 (s), 696 (m) cm^{-1}.

TLC (SiO_2, CD_2Cl_2:MeOH, 50:1 v/v): R_f = 0.30.

***Z*-Isomer:**

^{1}H NMR (600 MHz, CD_2Cl_2): δ = 8.52 (d, ^5J(H,H) = 2.5 Hz, 1H, H-C8), 8.17 (s, 1H, H-C33), 7.25 (s, 1H, H-C17), 7.19 (d, ^3J(H,H) = 8.3 Hz, 1H, H-C6), 7.11 (t, ^3J(H,H) = 8.9 Hz, 1H, H-C21), 7.00 (dd, ^3J(H,H) = 8.3 Hz, ^5J(H,H) = 2.5 Hz, 1H, H-C5), 6.92 (ddd, ^3J(H,H) = 9.0 Hz, ^4J(H,H) = 3.1 Hz, 1H, H-C22), 6.73 (dd, ^4J(H,H) = 3.1 Hz, 1H, H-C24), 5.24 – 5.15 (m, 2H, H_2-C35), 4.55 – 4.44 (m, 2H, H_2-C32), 4.21 – 4.11 (m, 2H, H_2-C25), 3.90 – 3.80 (m, 2H, H_2-C31), 3.79 – 3.75 (m, 2H, H_2-C26), 3.63 – 3.60 (m, 2H, H_2-C27), 3.56 – 3.52 (m, 2H, H_2-C28), 3.52 (m, 4H, H_2-C29, H_2-C30), 3.00 (d, ^2J(H,H) =

15.6 Hz, 1H, H-C3), 2.87 (d, ^{2}J(H,H) = 15.7 Hz, 1H, H'-C3), 2.50 (s, 3H, H_3-C36), 2.17 (s, 3H. H_3-C37), 1.58 (s, 3H, H_3-C10), 1.44 (s, 3H, H_3-C10') ppm.

^{13}C NMR (151 MHz, CD_2Cl_2): δ = 189.53 (C12), 161.98 (C1), 156.54 (C7), 155.50 (d, ^{4}J(C,F) = 1.1 Hz, C23), 154.71 (d, ^{1}J(C,F) = 235.7 Hz, C20), 145.34 (C18), 144.56 (C16), 143.75 (C34), 141.66 (C9), 140.37 (C14), 139.33 (C4), 133.98 (C15), 127.77 (C13), 127.56 (C11), 127.43 (d, ^{2}J(C,F) = 19.8 Hz, C19), 125.90 (C6), 124.95 (C33), 121.92 (C17), 120.80 (C5), 118.50 (d, ^{3}J(C,F) = 3.6 Hz, C24), 116.65 (d, ^{2}J(C,F) = 24.4 Hz, C21), 115.77 (d, ^{3}J(C,F) = 7.7 Hz, C22), 112.59 (C8), 70.89 (C28), 70.61 (C29), 70.19 (C27), 70.07 (C26), 70.03 (C30), 69.86 (C31), 68.91 (C25), 61.83 (C35), 50.56 (C32), 49.63 (C2), 48.86 (C3), 26.74 (C10), 26.49 (C10'), 21.48 (d, ^{5}J(C,F) = 1.4 Hz, C37), 16.59 (C36) ppm.

HR-MS (APPI$^+$, *m/z*) $[M+H]^+$ calc. for $[C_{38}H_{41}FN_3O_6S]^+$: 686.2695, found: 686.2693.

IR: $\tilde{\nu}$ = 3339 (m), 3147 (m), 2922 (s), 2851 (s), 2363 (w), 2331 (w), 2250 (w), 2155 (m), 1705 (w), 1654 (m), 1611 (m), 1496 (w), 1465 (m), 1458 (m), 1405 (m), 1356 (w), 1259 (w), 1205 (s), 1003 (w), 1064 (s), 952 (w), 879 (w), 853 (w) cm^{-1}.

TLC (SiO_2, CD_2Cl_2:MeOH, 50:1 v/v): R_f = 0.26.

(*E*/*Z*)-16-fluoro-24,26,32,32-tetramethyl-22,23,32,33-tetrahydro-31H,61H-4,9,12,15,18-pentaoxa-6(4,1)-triazola-2(5,2)-benzo[b]thiophena-3(1,6)-indena-1(1,3)-benzenacyclooctadecaphan-23-one 21-oxide (2)

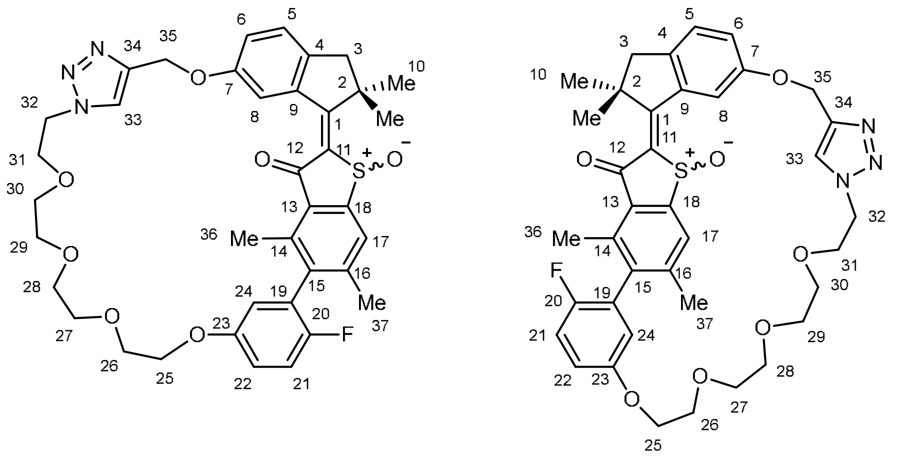

Macrocyclic compound **4** (30.4 mg, 44.3 µmol, 1.0 equiv.) was dissolved in acetic acid (2.5 mL, 0.02 M) and, after addition of sodium perborate tetrahydrate (11.3 mg, 73.4 µmol, 1.66 equiv.), the resulting mixture was stirred at 23 °C for 5 h. The reaction mixture was transferred dropwise to a separatory funnel charged with a saturated aqueous sodium bicarbonate solution (50 mL). The aqueous phase was extracted three times with EtOAc (50 mL each), washed with brine (50 mL), dried over anhydrous sodium sulfate, and the volatiles were removed *in vacuo*. The product was purified via flash column chromatography (SiO_2, CH_2Cl_2:MeOH, 40:1 v/v) giving compound **2** (20.2 mg, 28.7 µmol, 65%) as a yellow solid. Both *E*-configured atropisomers were obtained from the reaction and separated on a *Macherey-Nagel Nucleosil* SiO_2 column (100% EtOAc, at 40 °C). *Z*-configured isomers were obtained after irradiation using 505 nm or 565 nm and HPLC separation using a preparative *Macherey-Nagel Nucleosil* SiO_2 column (100% EtOAc, at 40 °C) followed by a preparative *Daicel* CHIRALPAK® IC column (*n*-hexane:EtOAc, 50:50 to 0:100 v/v, at 40 °C).

Only (*S*) configured diastereomers are shown in the following for clarity.

***E*-isomers:**

Isomer 2-*E*-I – *E*-(*R*)-(*M*)-(*R*$_a$)/*E*-(*S*)-(*P*)-(*S*$_a$)

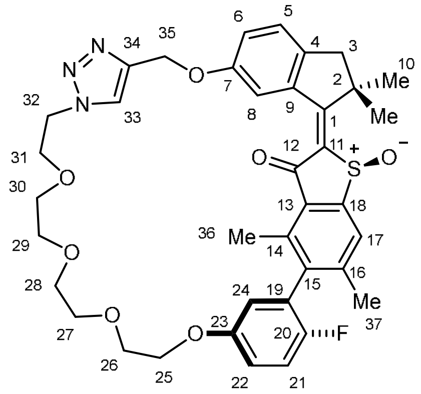

^{1}H NMR (800 MHz, CD_2Cl_2): δ = 7.87 (s, 1H, H-C33), 7.83 (s, 1H, H-C17), 7.75 (d, ^4J(H,H) = 2.5 Hz, 1H, H-C8), 7.29 (d, *J* = 8.2 Hz, 1H, H-C5), 7.15 (t, *J* = 8.8 Hz, 1H, H-C21), 7.10 (dd, ^3J(H,H) = 8.3 Hz, ^4J(H,H) = 2.5 Hz, 1H, H-C6), 6.96 (dt, *J*(H,H) = 9.0, 3.5, 1H, H-C22), 6.70 (dd, J(H,H) = 5.8, 3.1, 1H, H-C24), 5.17 – 5.08 (m, 2H, H_2-C35), 4.53 (qdd, J=14.3, 6.1, 4.0, 2H, H_2-C32), 4.17 – 4.10 (m, 2H, H_2-C25), 3.91 – 3.84 (m, 2H, H_2-C31), 3.78 (dd, J=5.1, 4.1, 2H, H_2-C26), 3.62 (dd, J=4.9, 4.1, 2H, H_2-C27), 3.57 – 3.52 (m, 6H, H_2-C28, H_2-C29 and H_2-C30), 3.20 (d, ^2J(H,H) = 14.8 Hz, 1H, H-C3), 2.81 (d, ^2J(H,H) = 14.8 Hz, 1H, H‘-C3), 2.53 (s, 3H, H_3-C36), 2.30 (s, 3H, H_3-C37), 1.94 (s, 3H, H_3-C10), 1.44 (s, 3H, H_3-C10‘) ppm.

^{13}C NMR (201 MHz, CD_2Cl_2) δ = 185.18 (C12), 174.51 (C11), 156.74(C7), 155.67 (C23), 154.05 (d, ^1J(C,F) = 236.9 Hz, C20), 150.13 (C18), 145.95 (C14), 143.63 (C34), 143.33 (C4), 141.37 (C15), 139.94 (C16), 139.40 (C1), 138.85 (C9), 130.53 (C13), 126.79 (d, ^2J(C,F) = 20,1 Hz, C19), 126.18 (C5), 125.16 (C17), 124.45 (C33), 121.56 (C6), 117.39 (d, ^3J(C,F) = 3.0 Hz, C24), 116.99 (d, ^2J(C,F) = 23.8 Hz, C21), 116.01 (d, ^3J(C,F) = 7.8 Hz, C22), 114.62 (C8), 71.13 (C27), 71.06 (C30), 70.97 (C29), 70.59 (C28), 70.00 (C26), 69.89 (C31), 68.71 (C25), 62.42 (C35), 52.58 (C2), 50.77 (C32), 49.48 (C3), 29.21 (C10), 26.07 (C10‘), 21.66 (C37), 16.79 (C36) ppm.

^{19}F NMR (377 MHz, CD_2Cl_2): δ = –126.53 (F-C20) ppm.

HR-MS (EI$^+$, *m/z*) $[M+H]^+$ calc. for $[C_{38}H_{40}FN_3O_7S]^+$: 701.2566, found: 701.2572.

IR: $\tilde{\nu}$ = 3139 (w), 2926 (s), 2869 (s), 1732 (s), 1673 (s), 1581 (s), 1539 (s), 1494 (s), 1485 (s), 1461 (m), 1373 (w), 1293 (m), 1242 (s), 1228 (s), 1207 (m), 1170 (w), 1143 (s), 1122 (s), 1051 (s), 1035 (s), 1002 (m), 947 (w), 862 (m), 817 (m), 792 (s), 742 (s), 714 (s), 635 (s), 510 (s), 411 (w) cm^{-1}.

TLC (SiO_2, CD_2Cl_2:MeOH, 40:1 v/v): R_f = 0.34.

Isomer 2-*E*-II – *E*-(*R*)-(*M*)-(*S_a*)/*E*-(*S*)-(*P*)-(*R_a*)

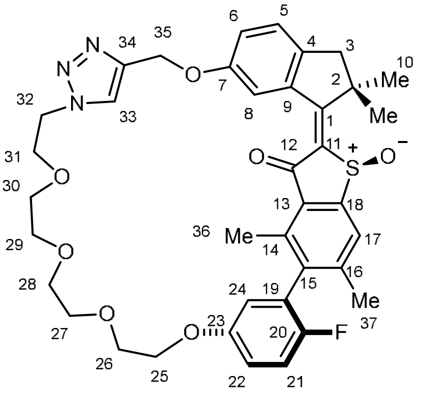

¹H NMR (800 MHz, CD_2Cl_2): δ = 8.16 (s, 1H, H-C33), 7.88 (d, ^4J(H,H) = 2.5 Hz, 1H, H-C8), 7.78 (s, 1H, H-C17), 7.25 (d, ^3J(H,H) = 8.3 Hz, 1H, H-C5), 7.15 (t, J(H,H) = 8.8 Hz, 1H, H-C21), 7.08 (dd, ^3J(H,H) = 8.2 Hz, ^4J(H,H) = 2.5 Hz, 1H, H-C6), 6.97 (dt, J(H,H) = 9.1 Hz, 3.5 Hz, 1H, H-C22), 6.71 (dd, J(H,H) = 5.8 Hz, 3.1 Hz, 1H, H-C24), 5.20 (d, J(H,H) = 2.6 Hz, 2H, H_2-C35), 4.54 – 4.42 (m, 2H, H_2-C32), 4.20 – 4.13 (m, 2H, H_2-C25), 3.89 – 3.80 (m, 2H, H_2-C31), 3.80 – 3.74 (m, 2H, H_2-C26), 3.62 – 3.58 (m, 2H, H_2-C27), 3.54 – 3.48 (m, 6H, H_2-C28, H_2-C29, H_2-C30), 3.02 (d, ^2J(H,H) = 15.3 Hz, 1H, H-C3), 2.95 (d, ^2J(H,H) = 15.3 Hz, 1H, H‘-C3), 2.53 (s, 3H, H_3-C36), 2.29 (s, 3H, H_3-C37), 1.75 (s, 3H, H_3-C10), 1.65 (s, 3H, H_3-C10‘) ppm.

¹³C NMR (201 MHz, CD_2Cl_2): δ = 188.44 (C12), 174.14 (C11), 156.31 (C7), 155.69 (C23), 154.15 (d, ^1J(C,F) = 238.2 Hz, C20), 149.31 (C18), 146.25 (C14), 143.73 (C4), 143.52 (C34), 141.75 (C15), 140.05 (C16), 138.22 (C9), 137.12 (C1), 129.99 (C13), 126.66 (d, ^2J(C,F) = 20.1 Hz, C19), 126.35 (C5), 125.70 (C17), 124.81 (C33), 123.12 (C6), 117.68 (C24), 116.93 (d, ^2J(C,F) = 23.8 Hz, C21), 116.11 (d, ^3J(C,F) = 7.2 Hz, C22), 113.98 (C8), 71.18 (C27), 71.00 (C28), 70.91 (C29), 70.62 (C30), 70.17 (C26), 69.75 (C31), 68.92 (C25), 61.77 (C35), 51.92 (C2), 50.42 (C32), 49.28 (C3), 29.37 (C10), 27.93 (C10’), 21.63 (C37), 16.77 (C36) ppm.

^{19}**F NMR** (377 MHz, CD_2Cl_2): δ = –126.00 (F-C20) ppm.

HR-MS (EI^+, *m/z*) $[M+H]^+$ calc. for $[C_{38}H_{40}FN_3O_7S]^+$: 701.2566, found: 701.2572.

IR: $\tilde{\nu}$ = 3150 (w), 2923 (s), 2867 (s), 1733 (s), 1669 (s), 1580 (s), 1536 (m), 1494 (s), 1484 (s), 1459 (m), 1417 (w), 1375 (w), 1297 (m), 1241 (m), 1229 (m), 1205 (m), 1192 (m), 1142 (m), 1104 (m), 1050 (s), 1034 (s), 938 (w), 892 (w), 861 (w), 816 (m), 793 (m), 741 (s), 634 (m), 536 (w) cm^{-1}.

TLC (SiO_2, CD_2Cl_2:MeOH, 40:1 v/v): R_f = 0.34.

***Z*-isomers:**

Isomer 2-*Z*-I – *Z*-(*R*)-(*M*)-(*R$_a$*)/*Z*-(*S*)-(*P*)-(*S$_a$*)

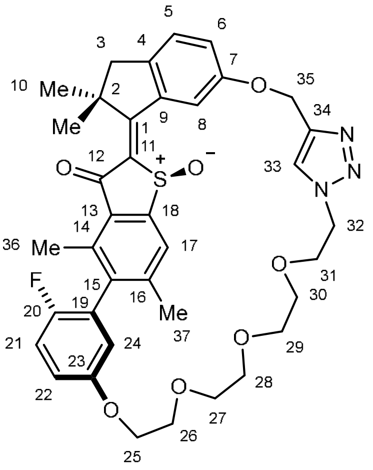

^{1}**H NMR** (800 MHz, CD_2Cl_2) δ = 8.68 (s, 1H, H-C33), 8.50 (d, J=2.4, 1H, H-C8), 7.79 (s, 1H, H-C17), 7.26 (d, *J*(H,H) = 8.3 Hz, 1H, H-C5), 7.18 – 7.13 (m, 2H, H-C6 and H-C24), 7.10 (t, J(H,H) = 8.8 Hz, 1H, H-C21), 6.96 (ddd, *J*(H,H) = 9.0 Hz, 4.1, 3.1, 1H, H-C22), 5.40 (dd, ^1J(H,H) = 88.3 Hz, 14.1, 4H, H_2-C35), 4.46 (dt, ^3J(H,H) = 6.1 Hz, 3.8, 2H, H_2-C32), 4.25 (dt, *J*(H,H) = 5.6, 2.8, 2H, H_2-C25), 3.74 (ddd, ^1J(H,H) = 10.1 Hz, ^3J(H,H) = 5.9 Hz, 3.8, 1H, H-C31), 3.72 (ddd, ^1J(H,H) = 11.3 Hz, 5.2, 3.2, 1H, H-C26), 3.68 (ddd, ^1J(H,H) = 11.3 Hz, 5.2, 3.2, 1H, H-C26), 3.63 (ddd, ^1J(H,H) = 10.4 Hz, ^3J(H,H) = 6.3 Hz, 3.9, 1H, H-C31), 3.53 – 3.46 (m, 2H, H_2-C27), 3.44 – 3.40 (m, 2H, H_2-C30), 3.40 – 3.36 (m, 1H, H-C29), 3.30 – 3.26 (m, 2H, H_2-C28), 3.24 (ddd, J=10.3, 9.0, 4.7, 1H, H-C29), 3.12 (d, ^1J(H,H) = 16.2 Hz, 1H, H-C3) 2.91 (d, ^1J(H,H) = 16.1 Hz, 1H, H'-C3), 2.52 (s, 3H, H_3-C36), 2.26 (s, 3H, H_3-C37), 1.57 (s, 3H, H_3-C10), 1.54 (s, 3H, H_3-C10') ppm.

^{13}**C NMR** (201 MHz, CD_2Cl_2) δ = 189.16 (C12), 179.36 (C11), 157.84 (C7), 156.64 (C23), 154.80 (C19), 153.62 (C20), 149.74 (C18), 146.11 (C14), 143.96 (C4), 143.08 (C34), 143.03 (C15), 140.22 (C16), 139.65 (C9), 134.23 (C1), 129.84 (C13), 126.90 (C5), 126.09 (C33), 125.98 (C17), 124.80 (C6), 119.54 (d, ^3J(C,F) = 7.7 Hz, C22), 118.03 (C24), 116.72 (d, ^2J(C,F) = 23.8 Hz, C21), 112.22 (C8), 72.12 (C26), 70.92 (C25), 70.88 (C27), 70.75 (C30), 70.66 (C29), 70.44 (C28), 69.81 (C31), 61.35 (C35), 50.68 (C2), 50.63 (C3), 50.48 (C32), 27.26 (C10), 25.43 (C10'), 21.96 (C37), 16.05 (C36) ppm.

^{19}**F NMR** (377 MHz, CD_2Cl_2): δ = –126.85 (F-C20) ppm.

HR-MS (EI^+, *m*/*z*) $[M+H]^+$ calc. for $[C_{38}H_{40}FN_3O_7S]^+$: 701.2566, found: 701.2572.

IR: $\tilde{\nu}$ = 3285 (w), 3123 (w), 2078 (w), 2958 (m), 2923 (s), 2853 (m), 1752 (m), 1704 (m), 1673 (m), 1599 (s), 1538 (s), 1486 (s), 1427 (m), 1377 (w), 1300 (w), 1275 (w), 1259 (w), 1230 (s), 1167 (w), 1143 (w), 1100 (m), 1052 (m), 1028 (m), 909 (w), 866 (w), 802 (m), 772 (w), 679 (s), 441 (w), 418 (s) cm^{-1}.

TLC (SiO_2, CD_2Cl_2:MeOH, 40:1 v/v): R_f = 0.34.

Isomer 2-*Z*-II – *Z*-(*R*)-(*M*)-(S_a)/*Z*-(*S*)-(*P*)-(R_a)

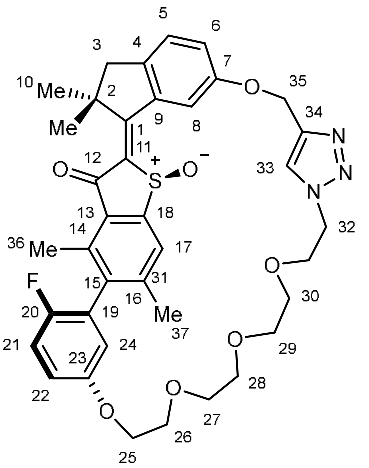

^{1}**H NMR** (800 MHz, CD_2Cl_2) δ = 8.09 (s, 1H, H-C33), 7.98 (d, ^4J(H,H) = 2.3 Hz, 1H, H-C8), 7.88 (s, 1H, H-C17), 7.28 (d, ^3J(H,H) = 8.4 Hz, 1H, H-C5), 7.20 (dd, ^3J(H,H) = 8.4 Hz, ^4J(H,H) = 2.3Hz, 1H, H-C6), 7.11 (t, *J*(H,H) = 8.7 Hz, 1H, H-C21), 7.03 (dd, *J*(H,H) = 5.8 Hz, 3.1 Hz, 1H, H-C24), 6.97 (dt, *J*(H,H) = 9.1 Hz, 3.6 Hz, 1H, H-C22), 5.44 – 5.37 (m, 3H, H_2-C35), 4.59 – 4.49 (m, 2H, H_2-C32), 4.29 – 4.20 (m, 2H, H_2-C25), 3.82 (ddd, *J*(H,H) = 10.5 Hz, 5.9, 4.9, 1H, H-C31), 3.73 (ddd,

J(H,H) = 10.2 Hz, 7.0, 4.8, 1H, H-C31), 3.70 – 3.63 (m, 2H, H_2-C26), 3.50 (dddd, J=28.6, 10.4, 6.0, 2.9, 2H, H_2-C27), 3.40 (dddd, J=40.3, 10.8, 6.1, 2.9, 2H, H_2-C28), 3.34 – 3.26 (m, 2H, H_2-C30 or H_2-C29), 3.25 – 3.19 (m, 2H, H_2-C30 or H_2-C29), 3.09 (d, ^2J(H,H) = 16.1 Hz, 1H, H-C3) , 2.95 (d, ^2J(H,H) = 16.1 Hz, 1H, H'-C3), 2.52 (s, 3H, H_3-C36), 2.28 (s, 3H, H_3-C37), 1.67 (s, 3H, H_3-C10), 1.52 (s, 3H, H_3-C10') ppm.

^{13}C NMR (201 MHz, CD_2Cl_2) δ = 186.87 (C12), 176.84 (C11), 158.05 (C7), 156.63 (C23), 154.22 (d, ^1J(C,F) = 238.3 Hz, C20), 148.40 (C18), 145.77 (C14), 145.15 (C4), 143.69 (C34), 142.58 (C15), 140.07 (C16), 138.47 (C9), 136.21 (C1), 130.53 (C13), 127.02 (C5), 126.03 (C17), 124.91 (C6), 124.70 (C33), 119.64 (C22), 119.60 (C19), 117.72 (C24), 116.86 (d, ^1J(C,F) = 22.8 Hz, C21), 112.92 (C8), 71.82 (C26), 71.28 (C27), 70.94 (C28), 70.74 (C25), 70.38 (C29 or C30), 70.17 (C29 or C30), 69.99 (C31), 62.52 (C35), 50.66 (C2), 50.60 (C3), 50.58 (C32), 26.41 (C10), 26.18 (C10'), 22.09 (C37), 16.10 (C36) ppm.

^{19}F NMR (377 MHz, CD_2Cl_2): δ = –126.10 (F-C20) ppm.

HR-MS (EI^+, *m*/*z*) $[M+H]^+$ calc. for $[C_{38}H_{40}FN_3O_7S]^+$: 701.2566, found: 701.2572.

IR: $\tilde{\nu}$ = 3143 (w), 3079 (w), 2921 (s), 2856 (s), 1732 (m), 1667 (s), 1604 (m), 1579 (s), 1523 (s), 1487 (s), 1456 (m), 1412 (w), 1379 (m), 1299 (w), 1285 (w), 1260 (w), 1221 (s), 1206 (s), 1144 (s), 1104 (s), 1036 (s), 1003 (w), 988 (m), 961 (w), 882 (m), 828 (s), 769 (m), 631 (m) cm^{-1}.

TLC (SiO_2, CD_2Cl_2:MeOH, 40:1 v/v): R_f = 0.34.

Synthesis of HTI 3

HTI motor **3** was constructed from indanone **18**, benzothiophenone **14** and biaryl **20**. These building blocks were combined forming HTI **3**, as depicted in Supplementary Figure 2. Building block **14** was synthesized according to a literature procedure[1] and building blocks **18** and **20** through a modified procedure thereof.

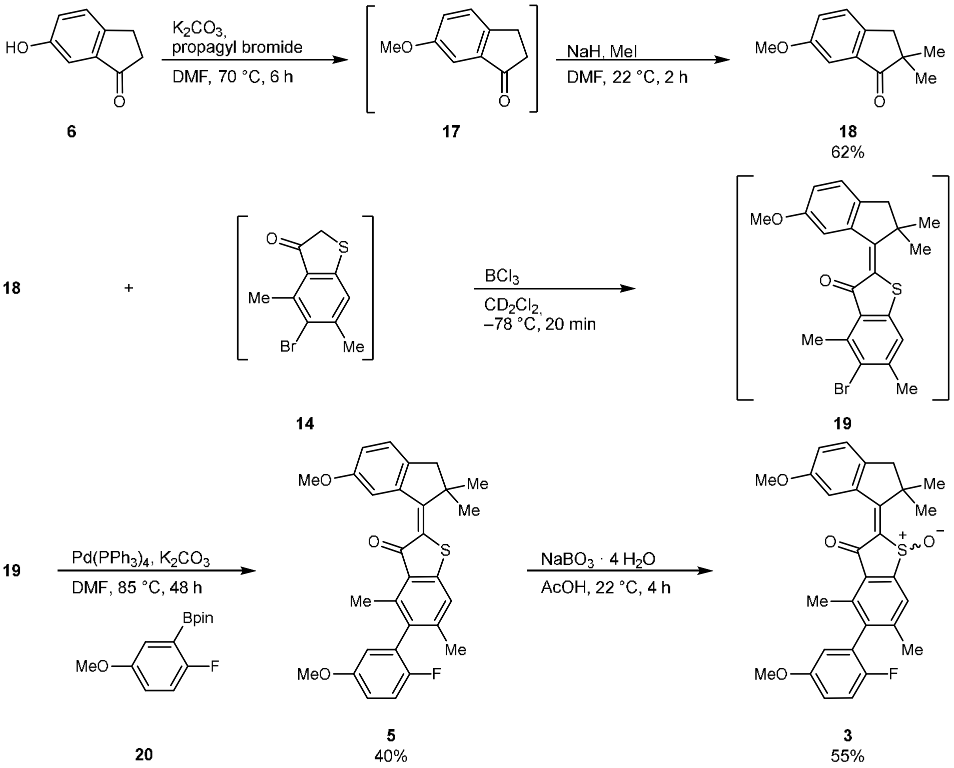

Supplementary Figure 3 Synthetic route for non-macrocyclic HTI 3. Building blocks **14**, **18** and **20** used for construction of HTI motor **3**.

6-methoxy-2,3-dihydro-1H-inden-1-one (17)

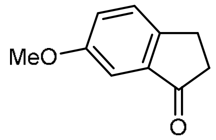

Commercially available 6-hydroxy-1-indanone **6** (1.00 g, 6.75 mmol, 1.0 equiv.) was dissolved in DMF (43 mL, 0.15 M) together with potassium carbonate (3.70 g, 27.0 mmol, 4.0 equiv.) and iodomethane (0.63 mL, 10.13 mmol, 1.5 equiv.). After stirring the mixture for 6 h at 70 °C, the reaction was stopped by pouring the mixture into a water (100 mL) filled separatory funnel. The aqueous phase was extracted three times with EtOAc (100 mL each) and the organic phases were washed four times with water (30 mL each to remove residual DMF) and with brine (100 mL) and dried over anhydrous sodium sulfate. Removal of the solvent *in vacuo* gave compound **17** which was used as the crude product without further purification.

HR-MS (APPI$^+$, *m/z*) $[M+H]^+$ calc. for $[C_{10}H_{11}O_2]^+$: 162.0754, found: 162.0751.

TLC (SiO_2, *i*-Hex:EtOAc, 1:1 v/v): $R_f = 0.60$.

6-methoxy-2,2-dimethyl-2,3-dihydro-1H-inden-1-one (18)

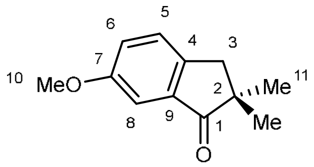

To a solution of compound **17** (775 mg, 4.78 mmol, 1.0 equiv.) in DMF (9.5 mL, 0.5 M) at 0 °C, sodium hydride (290 mg (60% in mineral oil), 7.18 mmol, 1.5 equiv.) was added. The mixture was stirred for 20 min at 0 °C. Then dropwise addition of methyl iodide (0.45 mL, 7.18 mmol, 1.5 equiv.) followed by stirring for another 2 h at 22 °C led to full conversion of starting material. After addition of a saturated aqueous ammonium chloride solution (25 mL), the aqueous phase was extracted three times with EtOAc (50 mL each). The combined organic phases were washed four times with water (20 mL each to remove residual DMF), twice with brine (30 mL each), dried over anhydrous sodium sulfate, and the solvent was removed *in vacuo*. Purification of the product was achieved with flash column chromatography (SiO_2, *i*-Hex:EtOAc, 9:1 v/v) yielding compound **18** as a colorless liquid (563 mg, 2.96 mmol, 62%).

^{1}H NMR (500 MHz, CD_2Cl_2): δ = 7.34 (dq, ^3J(H,H) = 8.3 Hz, ^4J(H,H) = 0.9 Hz, 1H, H-C5), 7.19 (dd, ^3J(H,H) = 8.3 Hz, ^5J(H,H) = 2.6 Hz, 1H, H-C6), 7.16 (d, ^5J(H,H) = 2.5 Hz, 1H, H-C8), 3.83 (s, 3H, H_3-C10), 2.92 (d, ^4J(H,H) = 0.8 Hz, 2H, H_2-C3), 1.20 (s, 6H, H_3-C11 and H_3-C11') ppm.

^{13}C NMR (126 MHz, CD_2Cl_2): δ = 211.39 (C1), 159.91 (C7), 145.39 (C4), 136.92 (C9), 127.81 (C5), 124.16 (C6), 105.85 (C8), 56.00 (C10), 46.57 (C2), 42.45 (C3), 25.46 (C11) ppm.

HR-MS (APPI$^+$, *m/z*) $[M+H]^+$ calc. for $[C_{12}H_{15}O_2]^+$: 191.1067, found: 191.1064.

IR: $\tilde{\nu}$ = 3400 (w), 3058 (w), 3001 (w), 2958 (s), 2924 (s), 2864 (m), 2835 (m), 1778 (w), 1704 (s), 1616 (m), 1585 (w), 1489 (s), 1464 (s), 1429 (s), 1379 (w), 1359 (w), 1334 (w), 1305 (m), 1276 (s), 1241 (s), 1153 (m), 1097 (w), 1027 (s), 1004 (s), 886 (m), 862 (m), 823 (m), 772 (s), 706 (w), 689 (w), 617 (w), 549 (w), 501 (w) cm^{-1}.

TLC (SiO_2, *i*-Hex:EtOAc, 9:1 v/v): R_f = 0.50.

2-(2-fluoro-5-methoxyphenyl)-4,4,5,5-tetramethyl-1,3,2-dioxaborolane (20)

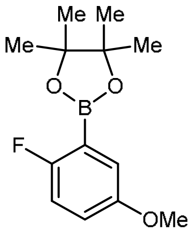

Commercially available 2-fluoro-5-hydroxyphenylboronic acid pinacol ester **12** (503 mg, 2.1 mmol, 1.0 equiv.) was dissolved in DMF (14 mL, 0.15 M) together with potassium carbonate (1.17 g, 8.4 mmol, 4.0 equiv.) and iodomethane (0.20 mL, 3.15 mmol, 1.5 equiv.). After the mixture was stirred for 18 h at 70°C the reaction was stopped by pouring the mixture into a separatory funnel charged with water (100 mL). The aqueous phase was extracted three times with EtOAc (100 mL each) and the combined organic phases were washed four times with water (30 mL each to remove residual DMF) and with brine (100 mL) and dried over anhydrous sodium sulfate. Removal of the solvent *in vacuo* gave compound **20** which was used as crude product without further purification.

HR-MS (APPI$^+$, *m/z*) $[M]^+$ calc. for $[C_{13}H_{18}BFO_3]^+$: 252.1333, found: 252.1328.

TLC (SiO_2, *i*-Hex:EtOAc, 1:1 v/v): $R_f = 0.91$.

(*E*/*Z*)-5-bromo-2-(6-methoxy-2,2-dimethyl-2,3-dihydro-1H-inden-1-ylidene)-4,6-dimethylbenzo[b]thiophen-3(2H)-one (19)

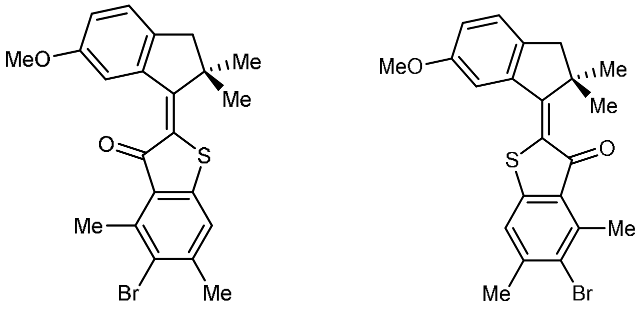

Two separate Schlenk flasks under inert N_2 gas atmosphere were used, one being charged with compound **18** (500 mg, 2.63 mmol, 1.0 equiv.) in anhydrous CH_2Cl_2 (5.1 mL, 0.52 M) at 0 °C and the other one with compound **14** (678.8 mg, 2.63 mmol, 1.0 equiv.) in anhydrous CH_2Cl_2 (1.2 mL, 2.116 M) at –78 °C. Boron trichloride (2.63 mL, 1 M in anhydrous CH_2Cl_2, 2.63 mmol, 1.0 equiv.) was added to the flask containing compound **14** and the resulting mixture was immediately taken up into the same syringe and transferred to the second flask containing compound **18** at 0 °C. The reaction mixture was vigorously stirred for 40 min while warming up to 22 °C. The reaction was stopped by pouring the mixture onto a saturated aqueous ammonium chloride solution (70 mL). Extraction of the aqueous phase was done four times with CH_2Cl_2 (70 mL each), the combined organic phases were dried over anhydrous sodium sulfate, and the solvent was removed *in vacuo*. The product was purified using flash column chromatography (SiO_2, *i*-Hex:EtOAc, 50:1 v/v) yielding compound **19** as a red solid which was used without further purification.

HR-MS (APPI$^+$, *m*/*z*) $[M+H]^+$ calc. for $[C_{22}H_{22}BrO_2S]^+$: 429.0518, found: 429.0517.

TLC (SiO_2, *i*-Hex:EtOAc, 50:1 v/v): $R_f = 0.22$ and 0.28.

(*E*/*Z*)-5-(2-fluoro-5-methoxyphenyl)-2-(6-methoxy-2,2-dimethyl-2,3-dihydro-1H-inden-1-ylidene)-4,6-dimethylbenzo[b]thiophen-3(2H)-one (5)

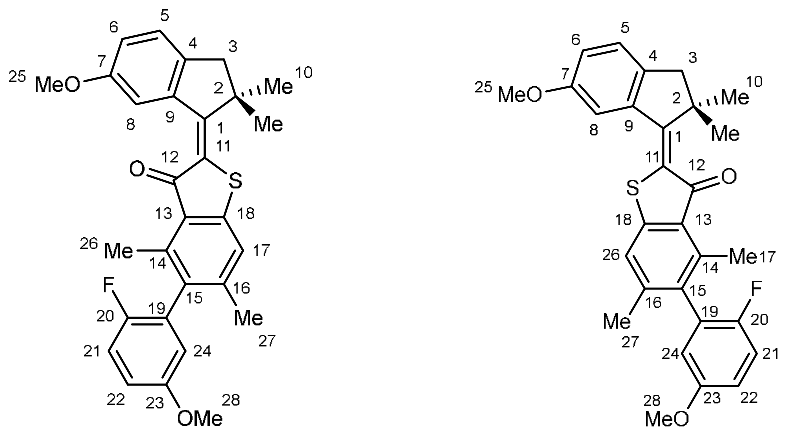

To a dried Schlenk tube under inert N_2 atmosphere compound **19** (318 mg, 0.74 mmol, 1.0 equiv.) and potassium phosphate (309 mg, 2.22 mmol, 3.0 equiv.) were added. After addition of anhydrous DMF (7.4 mL, 0.1 M) and tetrakis(triphenylphosphane)palladium(0) (42.5 mg, 0.037 mmol, 5 mol%), the suspension was heated at 85 °C for 48 h. The reaction was stopped by addition of a saturated aqueous ammonium chloride solution at 22 °C and the aqueous phase was extracted three times with EtOAc (50 mL each). The combined organic phases were dried over anhydrous sodium sulfate and the solvent was removed *in vacuo*. The crude product was purified using flash column chromatography (SiO_2, *i*-Hex:EtOAc, 50:1 v/v) yielding compound **5** as an orange solid (155 mg, 0.33 mmol, 40%). Four isomers (two atropisomers for each *E*- and *Z*-isomer) were obtained from HPLC separation using a preparative *Daicel* CHI-RALPAK® ID column (*n*-hexane:EtOAc, 80:20 to 75:25 v/v, at 40 °C).

***E*-Isomer:**

^{1}**H NMR** (600 MHz, CD_2Cl_2): δ = 8.47 (d, ^4J(H,H) = 2.5 Hz, 1H, H-C8), 7.23 (s, 1H, H-C17), 7.18 (dd, ^3J(H,H) = 8.3 Hz, 1H, H-C5), 7.11 (t, ^3J(H,H) = 8.9 Hz, 1H, H-C21), 6.95 (dd, ^3J(H,H) = 8.3 Hz, ^4J(H,H) = 2.5 Hz, 1H, H-C6), 6.92 (ddd, ^3J(H,H) = 9.0, ^4J(H,F) = 4.0 Hz, ^4J(H,H) = 3.2, 1H, H-C22), 6.65 (dd, ^4J(H,H) = 5.8 Hz, ^4J(H,H) = 3.2 Hz, 1H, H-C24), 3.82 (s, 3H, H_3-C25), 3.79 (s, 3H, H_3-C28), 2.93 (s, 2H, H_2-C3), 2.47 (s, 3H, H_3-C26), 2.12 (s, 3H, H_3-C27), 1.50 (s, 3H, H_3-C10), 1.26 (s, 3H, H_3-C10’).

^{13}**C NMR** (151 MHz, CD_2Cl_2): δ = 189.23 (C12), 161.56 (C1), 158.20 (C7), 156.38 (C23), 155.62 (C20), 145.16 (C18), 144.33 (C16), 140.96 (C4), 140.51 (C14), 139.45 (C9), 133.86 (C15), 127.66 (C13), 127.54 (C11), 127.44 (C19), 125.44 (C5), 121.96 (C17), 118.85 (C6), 116.59 (C21), 116.56 (C24), 114.79 (C22), 112.86 (C8), 56.16 (C28), 55.81 (C25), 48.91 (C3), 30.10 (C10), 26.77 (C10‘), 26.72 (C2), 21.70 (C27), 15.97 (C26).

^{19}**F NMR** (377 MHz, CD_2Cl_2): δ = –126.84 (F-C20) ppm.

HR-MS ($APPI^+$, *m*/*z*) $[M+H]^+$ calc. for $[C_{29}H_{28}FO_3S]^+$: 475.1738, found: 475.1737.

IR: $\tilde{\nu}$ = 3084 (w), 2991 (w), 2945 (s), 2917 (s), 2852 (m), 2831 (m), 1655 (s), 1601 (m), 1577 (s), 1513 (m), 1485 (s), 1448 (s), 1375 (m), 1288 (m), 1261 (m), 1249 (s), 1205 (s), 1174 (s), 1114 (w), 1032 (s), 956 (m), 856 (m), 812 (m), 786 (m), 748 (w), 595 (s) cm^{-1}.

TLC (SiO_2, *i*-Hex:EtOAc, 50:1 v/v): R_f = 0.33.

***Z*-Isomer:**

^{1}**H NMR** (400 MHz, CD_2Cl_2): δ = 7.65 (d, ^4J(H,H) = 2.3 Hz, 1H, H-C8), 7.28 – 7.24 (m, 1H, H-C17), 7.24 – 7.20 (m, 1H, H-C5), 7.12 (t, ^3J(H,H) = 8.9 Hz, 1H, H-C21), 6.99 (dd, ^3J(H,H) = 8.3 Hz, ^4J(H,H) = 2.3 Hz, 1H, H-C6), 6.92 (ddd, ^3J(H,H) = 9.0 Hz, ^4J(H,H) = 3.2 Hz, 1H, H-C22), 6.66 (dd, *J*(H,H) = 5.9 Hz, ^4J(H,H) = 3.1, 1H, H-C24), 3.90 (s, 3H, H_3-C25), 3.80 (s, 3H, H_3-C28), 3.00 (s, 2H, H_2-C3), 2.46 (s, 3H, H_3-C26), 2.13 (s, 3H, H_3-C27), 1.62 (s, 6H, H_3-C10 and H_3-C10’) ppm.

^{13}**C NMR** (126 MHz, CD_2Cl_2): δ = 189.18 (C12), 162.10 (C1), 159.39 (C7), 156.40 (C23), 154.51 (C20), 145.73 (C18), 144.17 (C16), 142.22 (C4), 141.80 (C9), 140.29 (C14), 134.20 (C15), 127.58 (C19), 127.49 (C13), 126.23 (C11), 126.11 (C5), 122.08 (C17), 118.28 (C6), 116.66 (C21), 116.60 (C24), 114.78 (C22), 112.37 (C8), 56.15 (C29), 55.99 (C25), 50.92 (C3), 49.35 (C2), 25.90 (C10), 25.74 (C10’), 21.69 (C27), 15.95 (C26) ppm.

^{19}**F NMR** (377 MHz, CD_2Cl_2): δ = –126.74 (F-C20) ppm.

HR-MS ($APPI^+$, *m*/*z*) $[M+H]^+$ calc. for $[C_{29}H_{28}FO_3S]^+$: 475.1738, found: 475.1737.

IR: $\tilde{\nu}$ = 2945 (s), 2922 (s), 2358 (m), 1656 (s), 1601 (m), 1583 (s), 1522 (m), 1487 (s), 1458 (s), 1376 (m), 1288 (m), 1261 (m), 1249 (s), 1208 (s), 1147 (s), 1120 (w), 1037 (s), 993 (m), 946 (m), 855 (m), 814 (m), 716 (w), 667 (w), 423 (s) cm^{-1}.

TLC (SiO_2, *i*-Hex:EtOAc, 50:1 v/v): R_f = 0.29.

(*E*/*Z*)-5-(2-fluoro-5-methoxyphenyl)-2-(6-methoxy-2,2-dimethyl-2,3-dihydro-1H-inden-1-ylidene)-4,6-dimethylbenzo[b]thiophen-3(2H)-one 1-oxide (3)

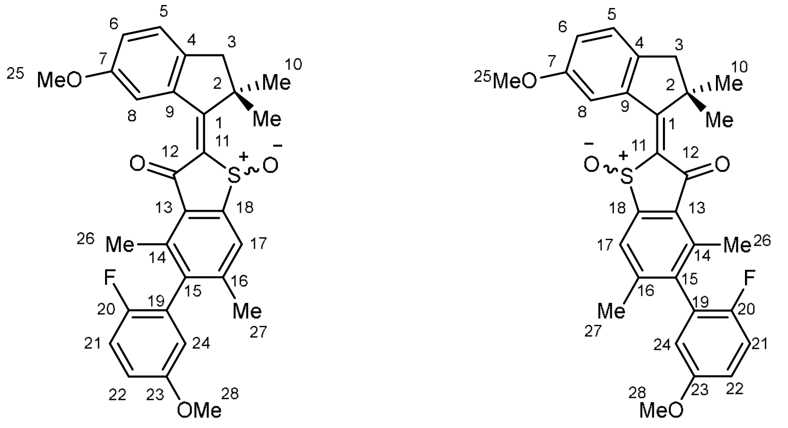

A solution of compound **5** (155 mg, 0.33 mmol, 1.0 equiv.) in acetic acid (15.1 mL, 0.02 M) was stirred after portionwise addition of sodium perborate tetrahydrate (101 mg, 0.66 mmol, 2.0 equiv.) at 23 °C for 3.5 h. The reaction mixture was transferred cautiously to a separatory funnel charged with saturated aqueous sodium bicarbonate solution (100 mL). The aqueous phase was extracted three times with EtOAc (100 mL each) and the combined organic phases were then washed with brine (100 mL), dried over anhydrous sodium sulfate, and the solvent was removed *in vacuo*. The crude product was purified via flash column chromatography (SiO_2, *i*-Hex:EtOAc, 3:2 v/v) yielding compound **3** (88.5 mg, 0.18 mmol, 55%) as a yellow solid. Four isomers (with differently configured sulfoxide stereocenter, biaryl chiral axis, and *E* or *Z* configuration of the double bond) could be separated from the product mixture via HPLC separation using a Diacel CHIRALPAK® ID column (*n*-hexane:EtOAc, 50:50 to 30:70 v/v, at 40 °C).

Only (*S*) configured diastereomers are shown in the following for clarity.

E-isomers:

Isomer 3-(*E*)-(*S*)-(S_a)

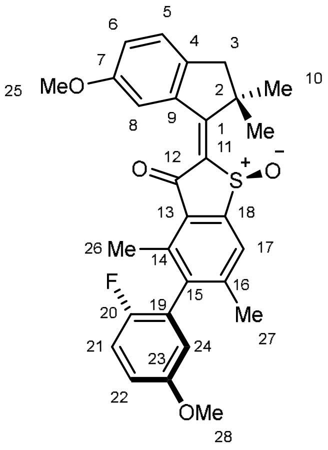

^{1}H NMR (601 MHz, CD_2Cl_2): δ = 7.86 (d, ^4J = 2.5 Hz, 1H, H-C8), 7.82 – 7.78 (m, 1H, H-C17), 7.26 (dp, J = 8.31, 1H, H-C5), 7.15 (t, ^3J = 8.9 Hz, 1H, H-C21), 7.05 (dd, ^3J = 8.3 Hz, ^4J = 2.5 Hz, 1H, H-C6), 6.96 (ddd, ^3J = 9.1 Hz, J = 4.0 Hz, ^4J = 3.2 Hz, 1H, H-C22), 6.65 (dd, J = 5.8 Hz, ^4J = 3.1 Hz, 1H, H-C24), 3.81 (s, 3H, H_3-C25), 3.81 (s, 3H, H_3-C28), 3.14 (d, ^2J = 15.2 Hz, 1H, H-C3), 2.85 (m, ^2J = 15.2 Hz, 1H, H'-C3), 2.52 (s, 3H, H_3-C26), 2.26 (s, 3H, H_3-C27), 1.89 (s, 3H, H_3-C10), 1.52 (s, 3H, H_3-C10').

^{13}C NMR (151 MHz, CD_2Cl_2): δ = 186.17 (C12), 174.94 (C1), 158.03 (C7), 156.59 (C23), 154.01 (d, ^1J(C,F) = 236.7 Hz, C20), 149.19 (C18), 145.92 (C16), 142.94 (C4), 141.53 (C15), 140.15 (C13), 138.69 (C9), 137.70 (C11), 130.11 (C14), 126.83 (d, ^2J(C,F) = 19.7 Hz, C19), 125.83 (C5), 125.70 (C17), 120.92 (C6), 116.92 (d, ^1J(C,F) = 24.0 Hz, C21), 115.81 (d, ^2J(C,F) = 3.4 Hz, C24), 115.30 (d, ^1J(C,F) = 7.8 Hz, C22), 114.45 (C8), 56.22 (C28), 55.86 (C25), 52.37 (C3), 49.41 (C2), 29.49 (C10), 27.06 (C10'), 21.88 (C26), 16.18 (C27).

^{19}F NMR (377 MHz, CD_2Cl_2): δ = –127.11 (F-C20) ppm.

HR-MS (ESI$^+$, *m*/*z*) $[M+H]^+$ calc. for $[C_{29}H_{28}FO_4S]^+$: 491.1687, found: 491.1683.

IR: $\tilde{\nu}$ = 3310 (w), 3153 (w), 2954 (w), 2917 (s), 2849 (s), 2359 (w), 1736 (w), 1662 (s), 1582 (w), 1559 (w), 1527 (w) 1496 (m), 1463 (m), 1405 (w), 1379 (w), 1296 (w), 1250 (w), 1206 (s), 1143 (w), 1123 (w), 1064 (w), 1032 (m), 948 (w), 851 (w), 717 (w), 418 (s) cm^{-1}.

TLC (SiO_2, *i*-Hex:EtOAc, 3:2 v/v): R_f = 0.39.

Isomer 3-(*E*)-(*S*)-(*R*a)

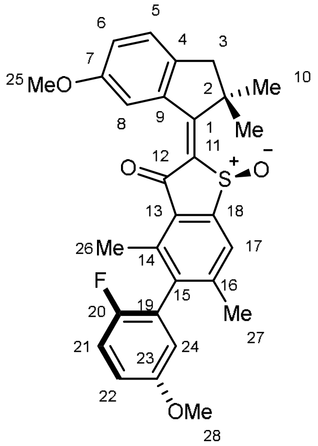

^{1}H NMR (601 MHz, CD_2Cl_2): δ = 7.82 (d, ^4J = 2.5 Hz, 1H, H-C8), 7.81 – 7.78 (m, 1H, H-C17), 7.28 – 7.23 (m, 1H, H-C5), 7.16 (t, ^3J = 8.9 Hz, 1H, H-C21), 7.04 (dd, ^3J = 8.3 Hz, ^4J = 2.5 Hz, 1H, H-C6), 6.96 (ddd, ^3J = 9.1 Hz, J = 4.0 Hz, ^4J = 3.2 Hz, 1H, H-C22), 6.62 (dd, J = 5.8 Hz, ^4J = 3.1 Hz, 1H, H-C24), 3.81 (s, 3H, H_3-C25), 3.80 (s, 3H, H_3-C28), 3.16 (d, ^2J = 15.1 Hz, 1H, H-C3), 2.83 (d, ^2J = 15.1 Hz, 1H, H'-C3), 2.50 (s, 3H, H_3-C26), 2.25 (s, 3H, H_3-C27), 1.91 (s, 3H, H_3-C10), 1.49 (s, 3H, H_3-C10').

^{13}C NMR (151 MHz, CD_2Cl_2): δ = 185.81 (C12), 174.95 (C1), 158.00 (C7), 156.54 (C23), 154.97 (d, ^1J(C,F) = 236.9 Hz, C20), 149.22 (C18), 145.93 (C16), 142.89 (C4), 141.50 (C15), 140.00 (C14), 138.73 (C9), 137.93 (C11), 130.24 (C13), 126.88 (d, ^2J(C,F) = 19.5 Hz, C19), 125.83 (C6), 125.62 (C17), 120.99 (C5), 116.97 (d, ^1J(C,F) = 24.3 Hz, C21), 115.87 (d, ^2J(C,F) = 3.3 Hz, C24), 115.24 (d, ^1J(C,F) = 7.7 Hz, C22), 114.31 (C8), 56.20 (C28), 55.85 (C25), 52.45 (C2), 49.42 (C3), 29.47 (C10), 26.76 (C10'), 21.84 (C26), 16.29 (C27).

^{19}F NMR (377 MHz, CD_2Cl_2): δ = –126.76 (F-C20) ppm.

HR-MS (ESI$^+$, *m/z*) $[M+H]^+$ calc. for $[C_{29}H_{28}FO_4S]^+$: 491.1687, found: 491.1683.

IR: $\tilde{\nu}$ = 3435 (w), 2955 (m), 2926 (m), 2848 (m), 2166 (w), 1673 (s), 1582 (s), 1538 (s), 1492 (s) 1488 (s), 1464 (m), 1419 (w), 1378 (w), 1293 (m), 1251 (m), 1117 (w), 1207 (s), 1152 (m), 1056 (m), 1034 (s), 954 (w), 862 (m), 816 (m), 792 (w), 740 (w), 712 (w) cm^{-1}.

TLC (SiO_2, *i*-Hex:EtOAc, 3:2 v/v): R_f = 0.39.

***Z*-isomers:**

Isomer 3-(*Z*)-(*S*)-(S_a)

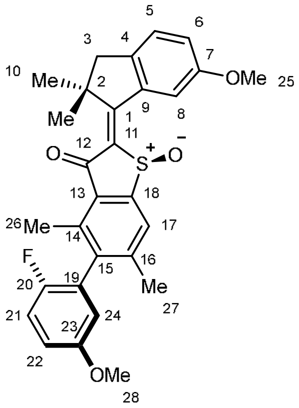

^{1}H NMR (601 MHz, CD_2Cl_2): δ = 8.09 (d, ^4J = 2.3 Hz, 1H, H-C8), 7.80 – 7.77 (m, 1H, H-C17), 7.28 (dq, ^3J = 8.4 Hz, J = 0.9 Hz, 1H, H-C5), 7.15 (t, ^3J = 8.9 Hz, 1H, H-C21), 7.13 (dd, ^3J = 8.4 Hz, ^4J = 2.4 Hz, 1H, H-C6), 6.96 (ddd, ^3J = 9.1 Hz, J = 4.0 Hz, ^4J = 3.2 Hz, 1H, H-C22), 6.65 (dd, J = 5.8 Hz, ^4J = 3.1 Hz, 1H, H-C24), 3.93 (s, 3H, H_3-C25), 3.80 (s, 3H, H_3-C28), 3.03 (d, ^2J = 16.2 Hz, 1H, H-C3), 3.04 (d, ^2J = 16.2 Hz, 1H, H'-C3), 2.50 (s, 3H, H_3-C26), 2.24 (s, 3H, H_3-C27), 1.64 (s, 3H, H_3-C10), 1.55 (s, 3H, H_3-C10').

^{13}C NMR (151 MHz, CD_2Cl_2): δ = 188.07 (C12), 176.45 (C1), 159.70 (C7), 156.61 (C23), 153.96 (d, ^1J(C,F) = 236.5 Hz, C20), 149.68 (C18), 145.83 (C16), 143.67 (C4), 141.72 (C15), 140.10 (C14), 139.33 (C9), 136.76 (C11), 130.41 (C13), 126.78 (d, ^2J(C,F) = 19.6 Hz, C19), 126.46 (C5), 125.96 (C17), 121.47 (C6), 116.91 (d, ^2J(C,F) = 24.0 Hz, C21), 115.74 (d, ^3J(C,F) = 3.3 Hz, C24), 115.40 (d, ^1J(C,F) = 7.8 Hz, C22), 113.31 (C8), 56.21 (C28), 56.12 (C25), 50.51 (C3), 50.46 (C2), 26.63 (C10), 26.31 (C10'), 21.83 (C27), 16.10 (C26).

^{19}F NMR (377 MHz, CD_2Cl_2): δ = –127.17 (F-C20) ppm.

HR-MS (ESI^+, *m*/*z*) $[M+H]^+$ calc. for $[C_{29}H_{28}FO_4S]^+$: 491.1687, found: 491.1680.

IR: $\tilde{\nu}$ = 3359 (s), 3186 (m), 3001 (w), 2956 (w), 2922 (s), 2851 (s), 2360 (w), 2335 (w), 1659 (s) 1632 (s), 1582 (w), 1539 (w), 1496 (m), 1468 (m), 1423 (w), 1411 (w), 1294 (w), 1206 (m), 1142 (w), 1035 (s), 861 (w), 818 (w), 711 (w), 643 (w), 408 (s) cm^{-1}.

TLC (SiO_2, *i*-Hex:EtOAc, 3:2 v/v): R_f = 0.26.

Isomer 3-(*Z*)-(*S*)-(*R*a)

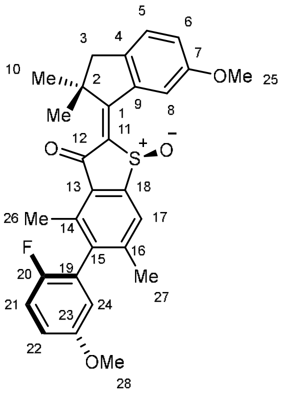

^{1}H NMR (601 MHz, CD_2Cl_2): δ = 8.06 (d, ^4J = 2.3 Hz, 1H, H-C8), 7.79 (p, J = 0.67 Hz, 1H, H-C17), 7.28 (dq, ^3J = 8.4 Hz, J = 0.9 Hz, 1H, H-C5), 7.16 (t, ^3J = 8.9 Hz, 1H, H-C21), 7.13 (dd, ^3J = 8.4 Hz, ^4J = 2.4 Hz, 1H, H-C6), 6.96 (ddd, ^3J = 9.1 Hz, J = 4.0 Hz, ^4J = 3.2 Hz, 1H, H-C22), 6.62 (dd, J = 5.8 Hz, ^4J = 3.2 Hz, 1H, H-C24), 3.93 (s, 3H, H_3-C25), 3.80 (s, 3H, H_3-C28), 3.06 (d, ^2J = 16.1 Hz, 1H, H-C3), 3.00 (d, ^2J = 16.1 Hz, 1H, H'-C3), 2.50 (s, 3H, H_3-C26), 2.24 (s, 3H, H_3-C27), 1.64 (s, 3H, H_3-C10), 1.55 (s, 3H, H_3-C10').

^{13}C NMR (151 MHz, CD_2Cl_2): δ = 188.07 (C12), 176.45 (C1), 159.70 (C7), 156.61 (C23), 153.96 (d, ^1J(C,F) = 236.5 Hz, C20), 149.68 (C18), 145.83 (C16), 143.67 (C4), 141.72 (C15), 140.10 (C14), 139.33 (C9), 136.76 (C11), 130.41 (C13), 126.78 (d, ^2J(C,F) = 19.6 Hz, C19), 126.46 (C5), 125.96 (C17), 121.47 (C6), 116.91 (d, ^2J(C,F) = 24.0 Hz, C21), 115.74 (d, ^3J(C,F) = 3.3 Hz, C24), 115.40 (d, ^1J(C,F) = 7.8 Hz, C22), 113.31 (C8), 56.21 (C28), 56.12 (C25), 50.51 (C3), 50.46 (C2), 26.63 (C10), 26.31 (C10'), 21.83 (C27), 16.10 (C26).

^{19}F NMR (377 MHz, CD_2Cl_2): δ = –126.53 (F-C20) ppm.

HR-MS (ESI^+, *m*/*z*) $[M+H]^+$ calc. for $[C_{29}H_{28}FO_4S]^+$: 491.1687, found: 491.1689.

IR: $\tilde{\nu}$ = 3433 (m), 2961 (m), 2929 (w), 2866 (w), 2836 (w), 1673 (s), 1606 (w), 1582 (w), 1536 (s), 1491 (s), 1463 (w), 1421 (s), 1378 (w), 1292 (s), 1251 (m), 1228 (m), 1203 (s), 1145 (m), 1121 (w), 1067 (w), 1032 (s), 991 (w), 953 (m), 918 (w), 878 (m), 816 (m), 718 (m), 618 (w), 563 (w) cm^{-1}.

TLC (SiO_2, *i*-Hex:EtOAc, 3:2 v/v): R_f = 0.26.

Supplementary Note 3: Structural and Conformational Description

Overview of Conformational States for Macrocyclic HTI 2

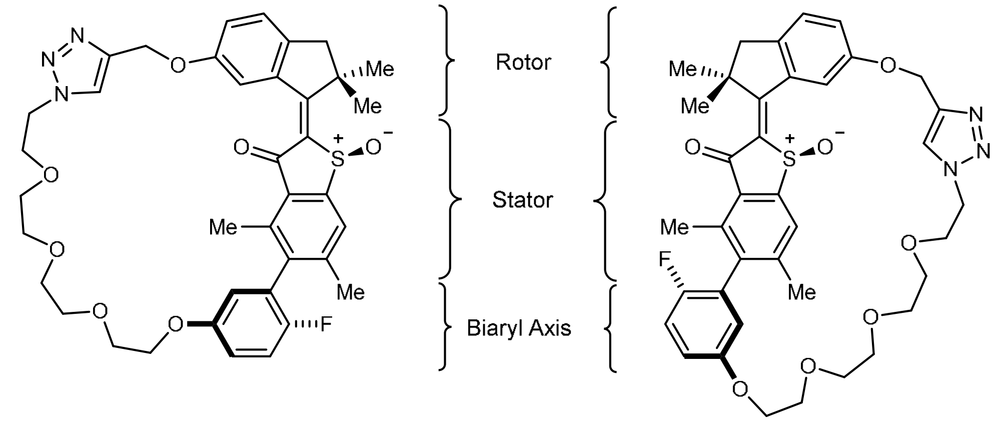

2-*E*-I **2-*Z*-I**

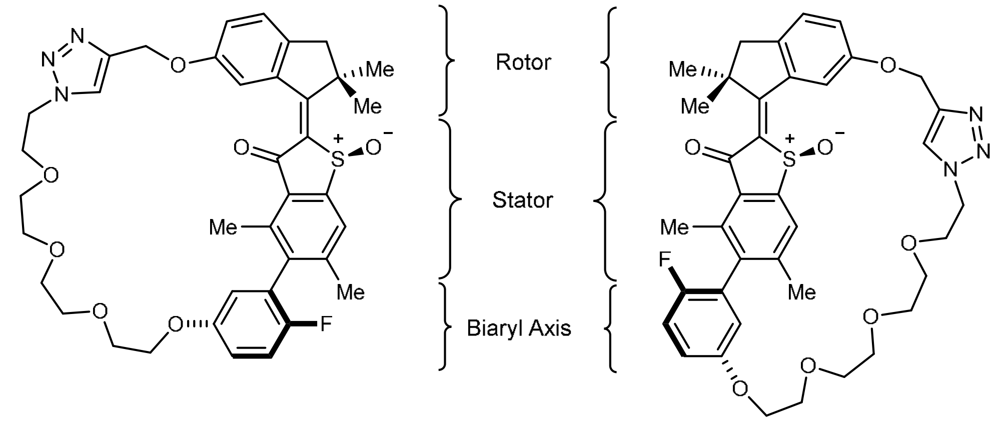

2-*E*-II **2-*Z*-II**

Supplementary Figure 4 Overview of conformational states of HTI 2. Overview of the isomeric states for **2**-*E*-**I**, **2**-*Z*-**I**, **2**-*E*-**II**, and **2**-*Z*-**II**, of macrocyclic compound **2**, which are stable at 22 °C. For clarity only structures with (*S*) configured sulfoxide are depicted. The set of isomers consists of two (*E*) and two (*Z*) configured isomers with either a (R_a) or (S_a) configured biaryl axis. The helicity of the rotor fragment is highly dynamic and can thus not be determined with certainty at ambient temperatures.

Overview of Conformational States for HTI 3

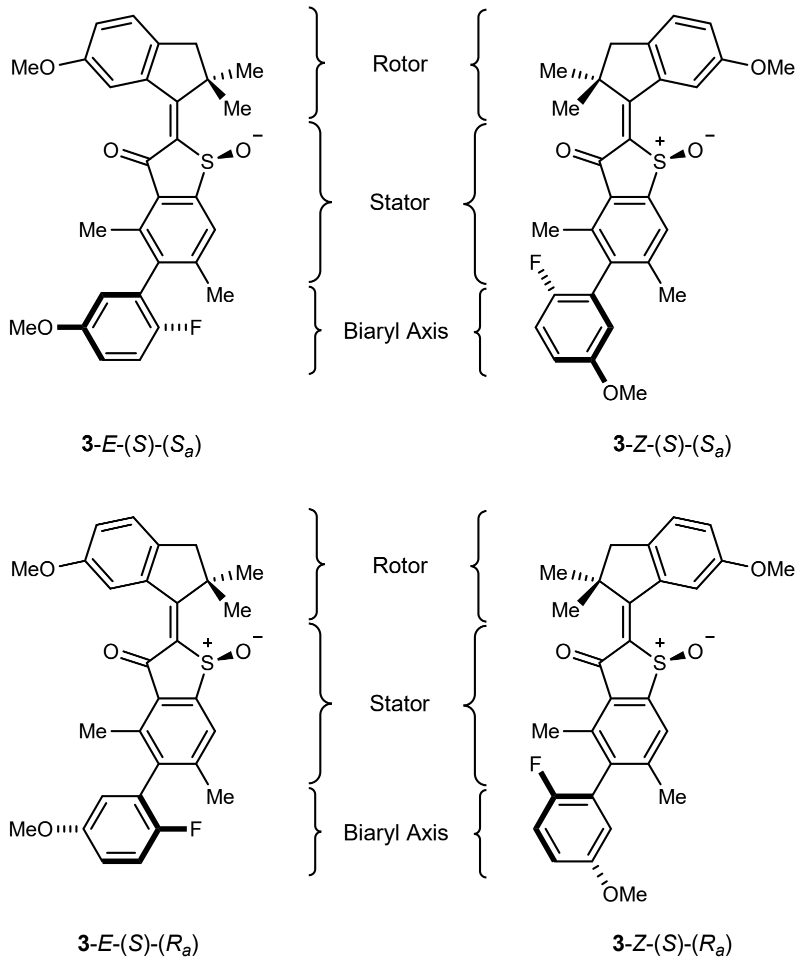

Supplementary Figure 5 Overview of conformational states of HTI 3. Overview of the isomeric states for **3**-*E*-(*S*)-(*R*a), **3**-*Z*-(*S*)-(*R*a), **3**-*E*-(*S*)-(*S*a), and **3**-*Z*-(*S*)-(*S*a), which are stable at 22 °C. For clarity only structures with (*S*) configured sulfoxide are depicted. The set of isomers consists of two (*E*) and two (*Z*) configured isomers with either a (R_a) or (S_a) configured atropisomer respectively. The helicity of the rotor fragment is highly dynamic and can thus not be determined with certainty at ambient temperatures.

Structure of Macrocyclic HTI 2 in Solution

The structures of the different isomers of macrocyclic **2** in solution were analyzed by 1D and 2D NMR spectroscopy. 2D NOESY and 1D NOE NMR spectra were compared for isomer **2**-*E*-**II** and **2**-*Z*-**II** demonstrating equivalent information content regarding double bond configuration. For all other isomers 1D NOE NMR spectra were used for evidencing the double bond configuration to minimize acquisition time.

Aromatic regions of the ^{1}H NMR spectra of all four isomers **2**-*E*-**I**, **2**-*Z*-**I**, **2**-*E*-**II**, and **2**-*Z*-**II** are shown in Figure 7 for comparison, evidencing distinct spectral features facilitating the analysis of thermal and photochemical behavior by NMR spectroscopy. Only structures with (*S*) configured sulfoxides will be considered in the following for reasons of clarity.

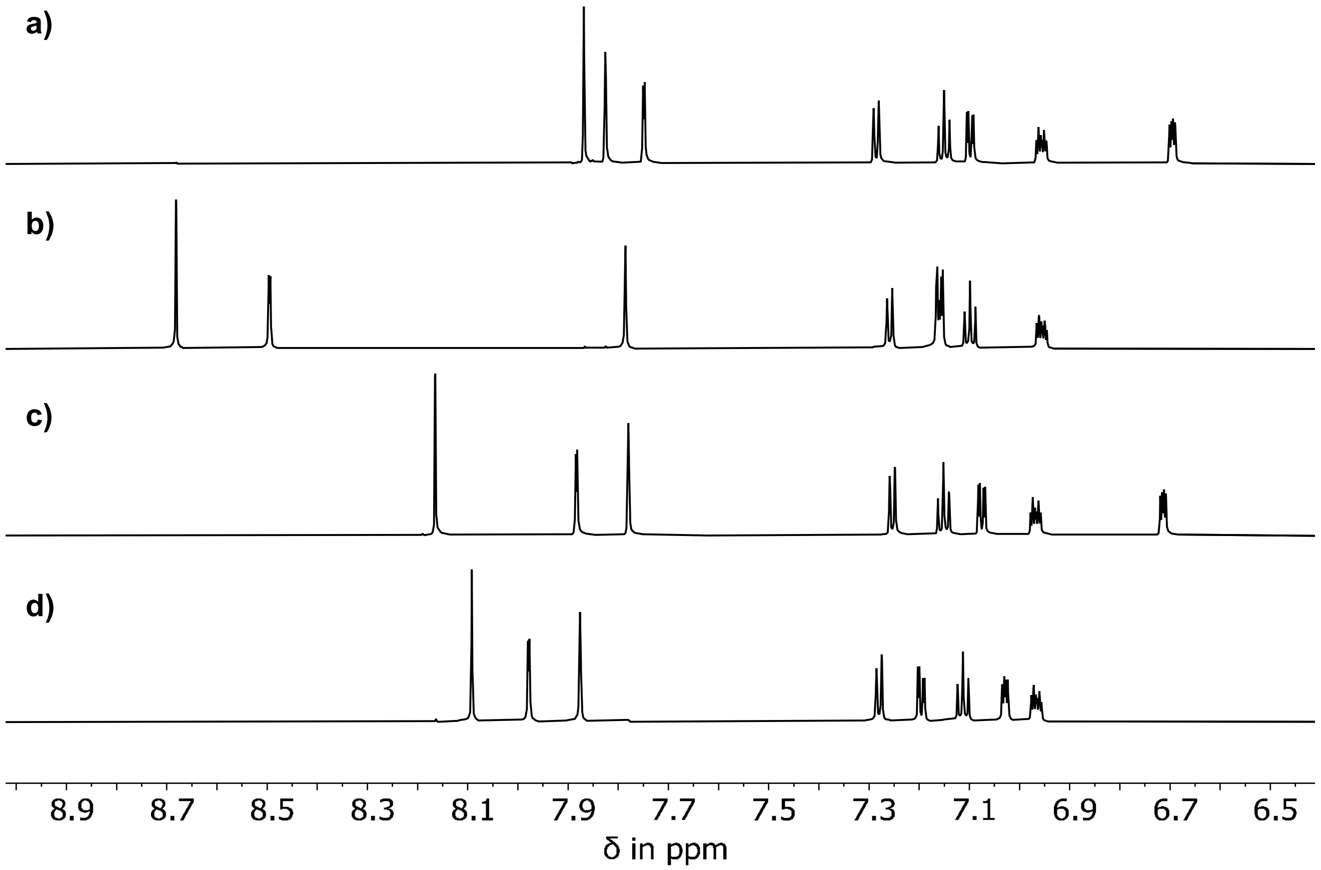

Supplementary Figure 6 Overview of ^{1}H NMR spectra of HTI 2. Aromatic region of ^{1}H NMR spectra (CD_2Cl_2, 400 MHz, 25 °C) of isomers a) **2**-*E*-**I**, b) **2**-*Z*-**I**, c) **2**-*E*-**II** and d) **2**-*Z*-**II** showing distinctly shifted signals.

Isomer 2-*E*-I

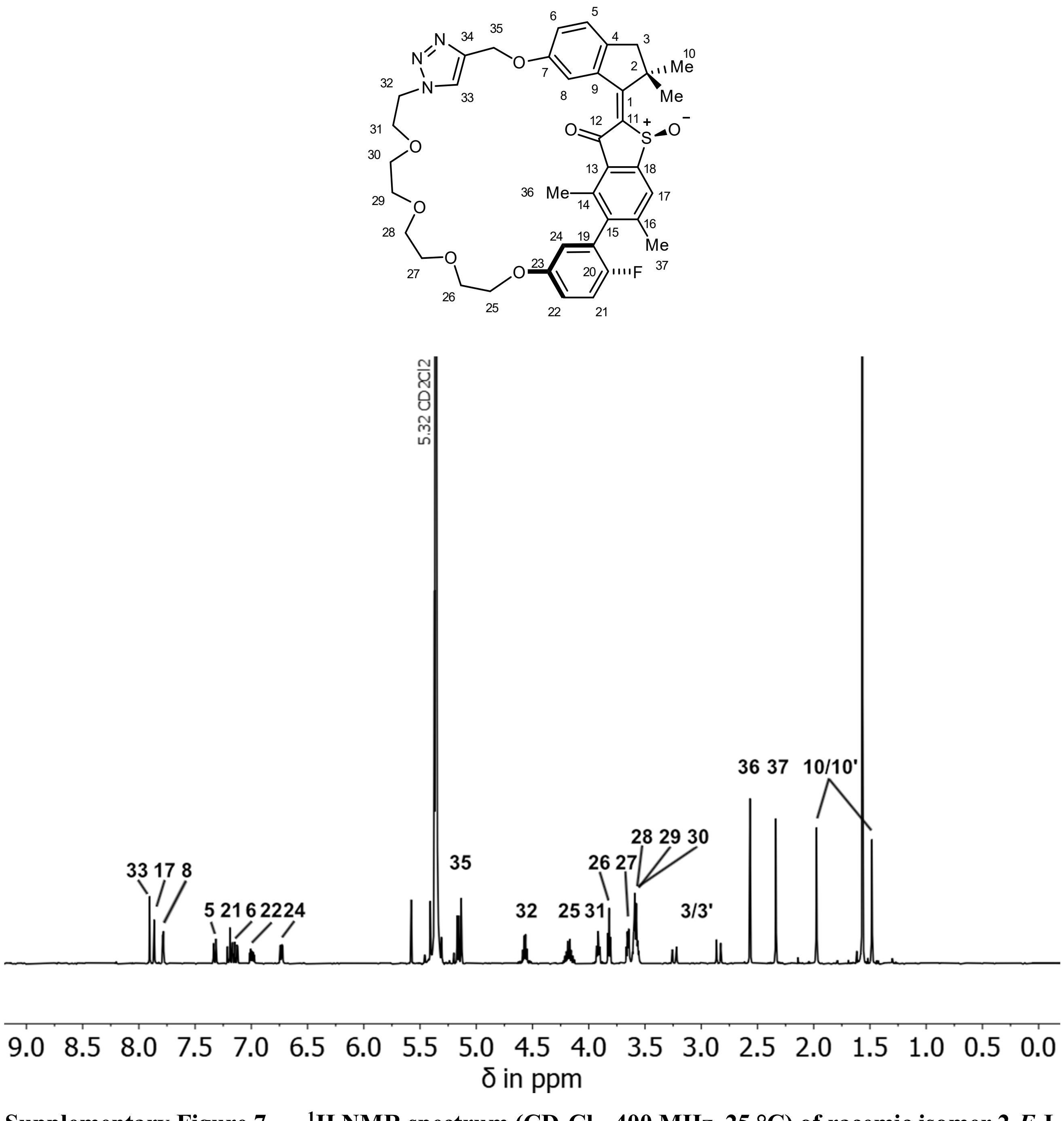

Supplementary Figure 7 **^{1}H NMR spectrum (CD_2Cl_2, 400 MHz, 25 °C) of racemic isomer 2-*E*-I.** Arbitrary given numbers 1-35 were used for assignment of signals.

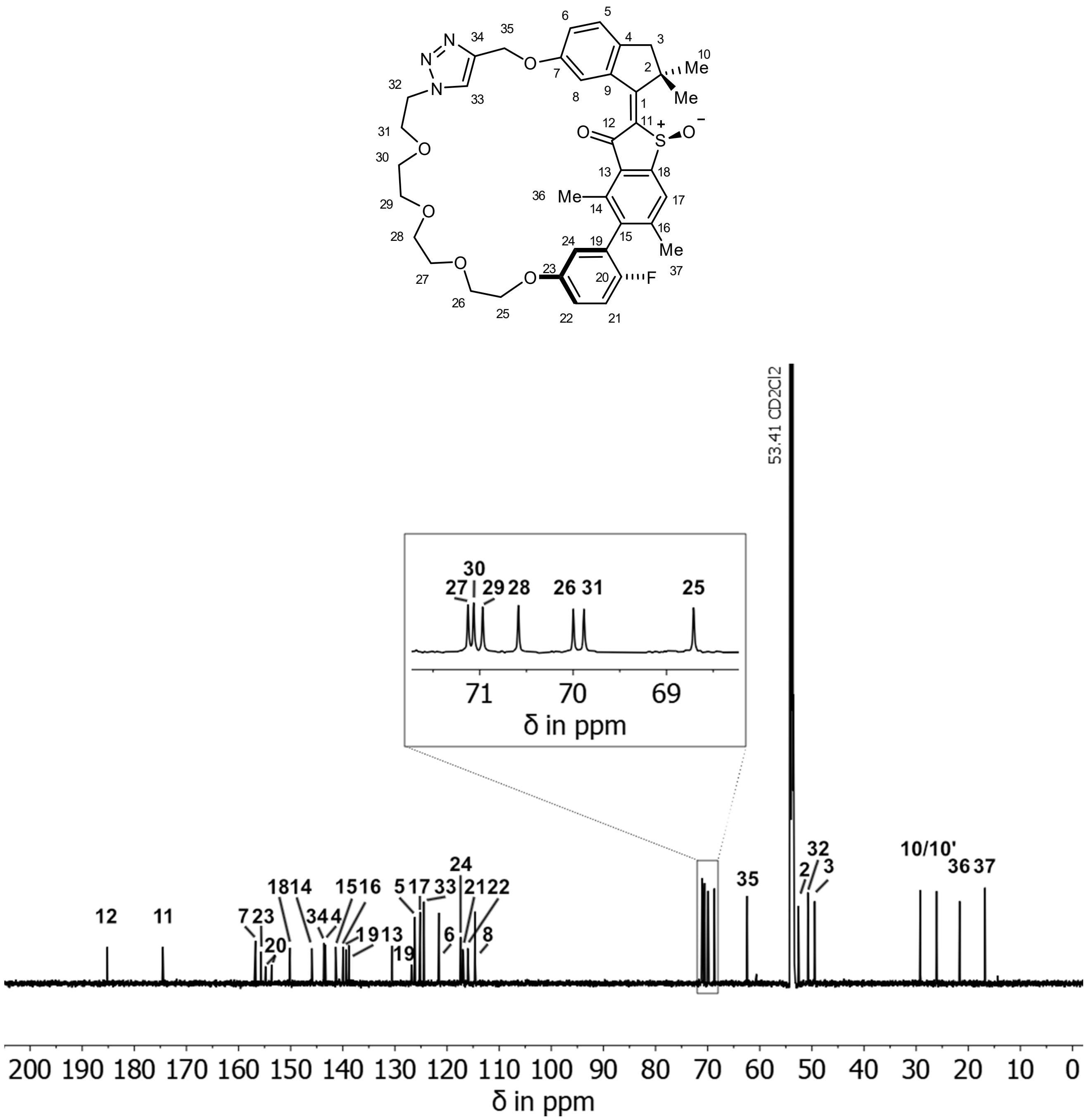

Supplementary Figure 8 **^{13}C NMR spectrum (CD_2Cl_2, 201 MHz, 25 °C) of racemic isomer 2-*E*-I.** Arbitrary given numbers 1-35 were used for assignment of signals.

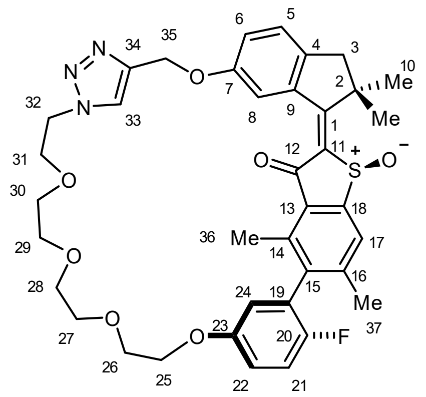

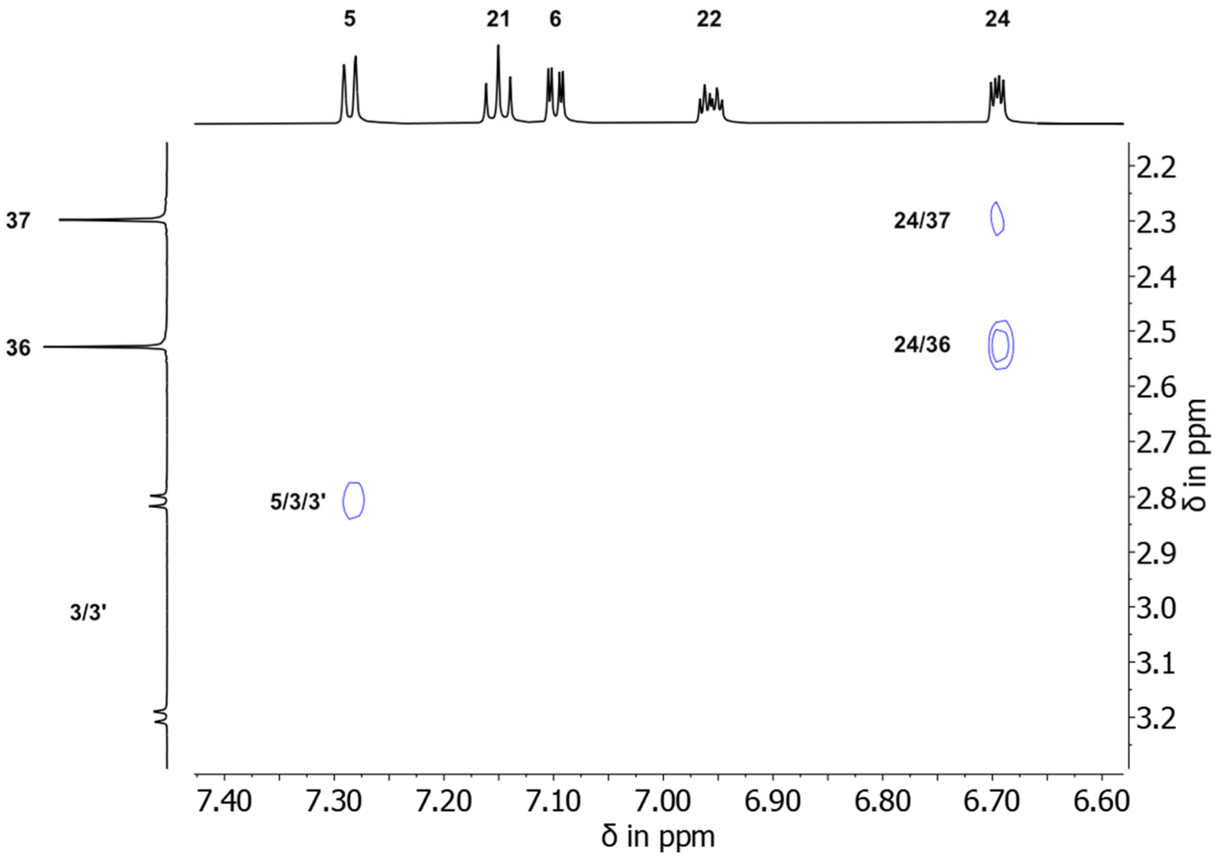

Supplementary Figure 9 NOESY NMR spectrum (CD_2Cl_2, 800 MHz, 25 °C) of isomer 2-*E*-II evidencing biaryl axis tilt. Intensity difference between cross signals (blue) of aromatic methyl groups H_3-C36 and H_3-C37 to the aromatic proton H-C24 indicate the two aromatic planes are non-orthogonal. The stronger cross signal between proton H-C24 and methyl group H_3-C36 suggests a stronger preferred tilt of the biaryl, which brings aromatic proton H-C24 (and thus also anchoring point of the covalent linker C23 towards the rest of the chain) closer to H_3-C36.

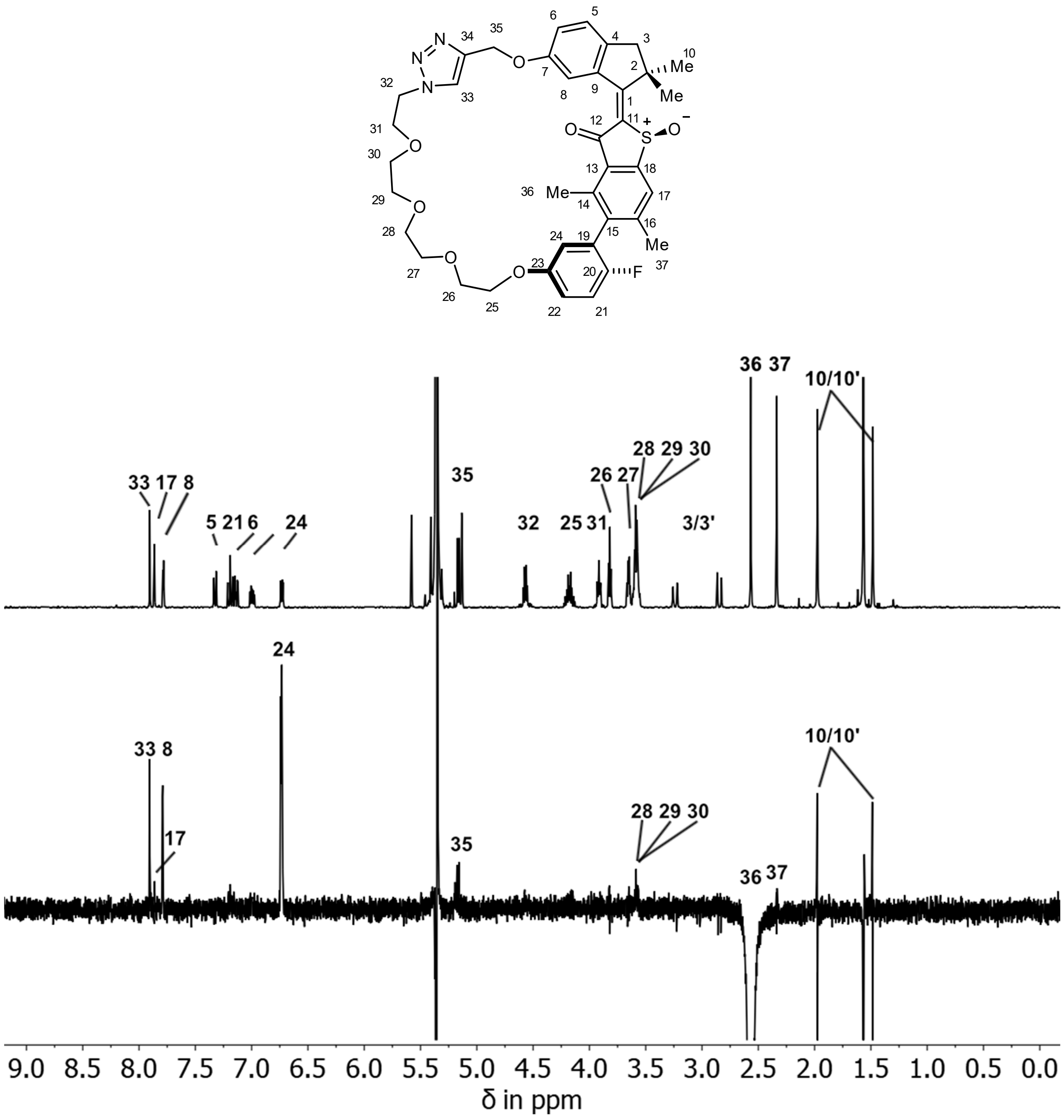

Supplementary Figure 10 NOE NMR spectrum (CD_2Cl_2, 600 MHz, 25 °C) of isomer 2-*E*-I evidencing *E*-configuration. Double bond configuration was assigned by coupling of aromatic proton H-C8 and aromatic triazole proton H-C33 to aromatic methyl group H_3-C36.

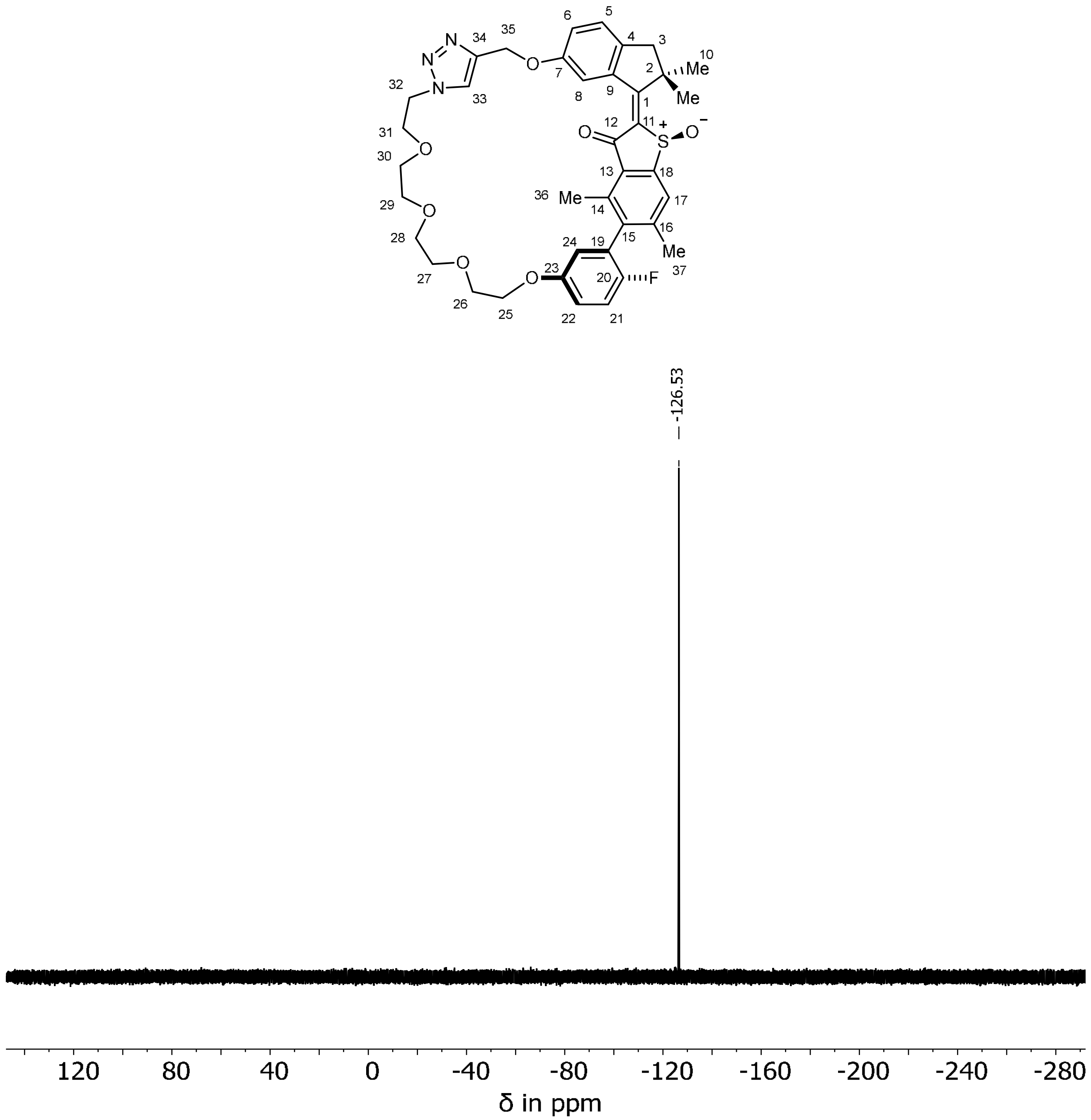

Supplementary Figure 11 **^{19}F NMR spectrum (CD_2Cl_2, 377 MHz, 25 °C) of racemic isomer 2-*E*-I.** One distinct signal at –126.53 ppm is observed.

Isomer 2-*Z*-I

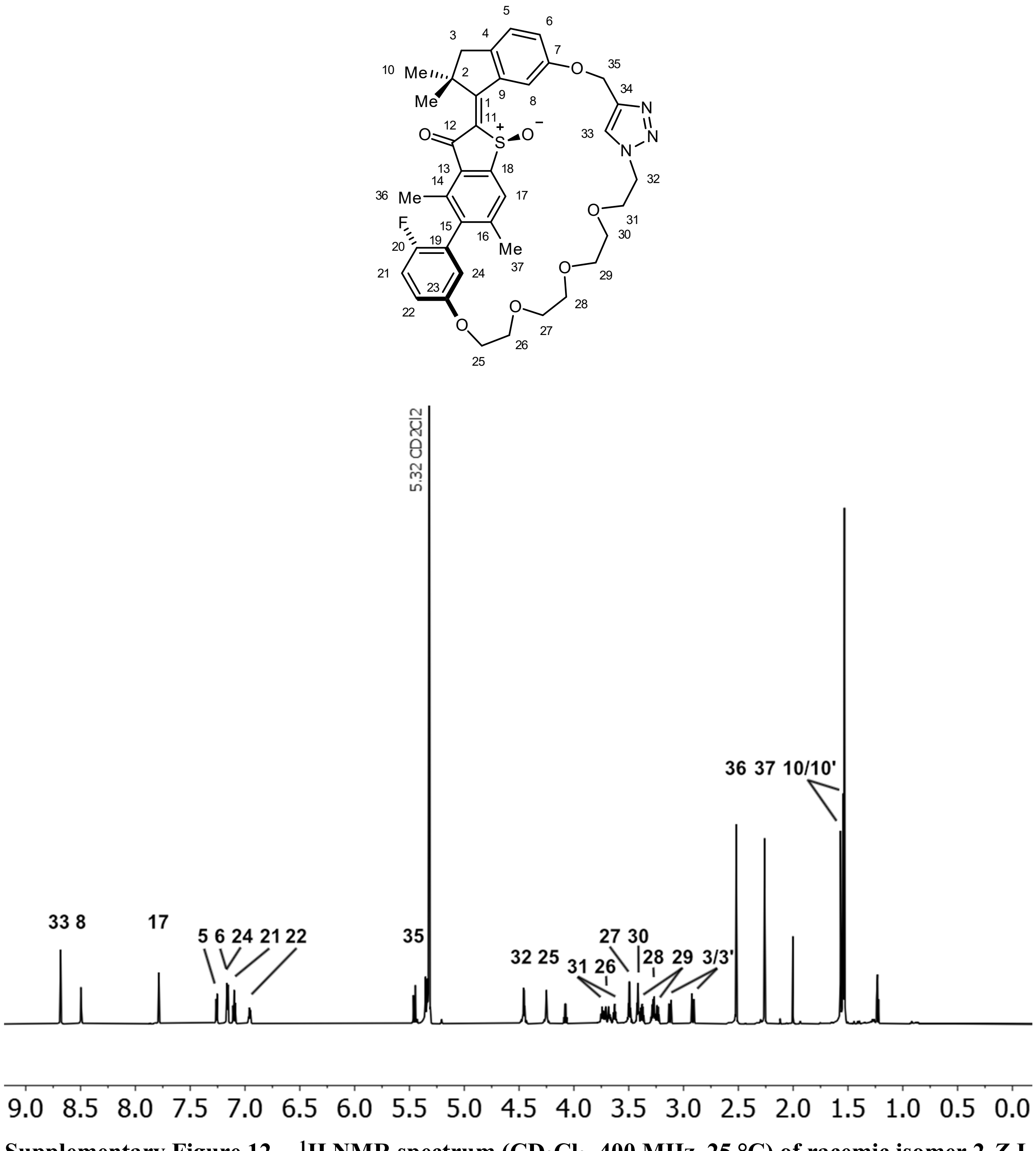

Supplementary Figure 12 **^{1}H NMR spectrum (CD_2Cl_2, 400 MHz, 25 °C) of racemic isomer 2-*Z*-I.** Arbitrary given numbers 1-35 were used for assignment of signals.

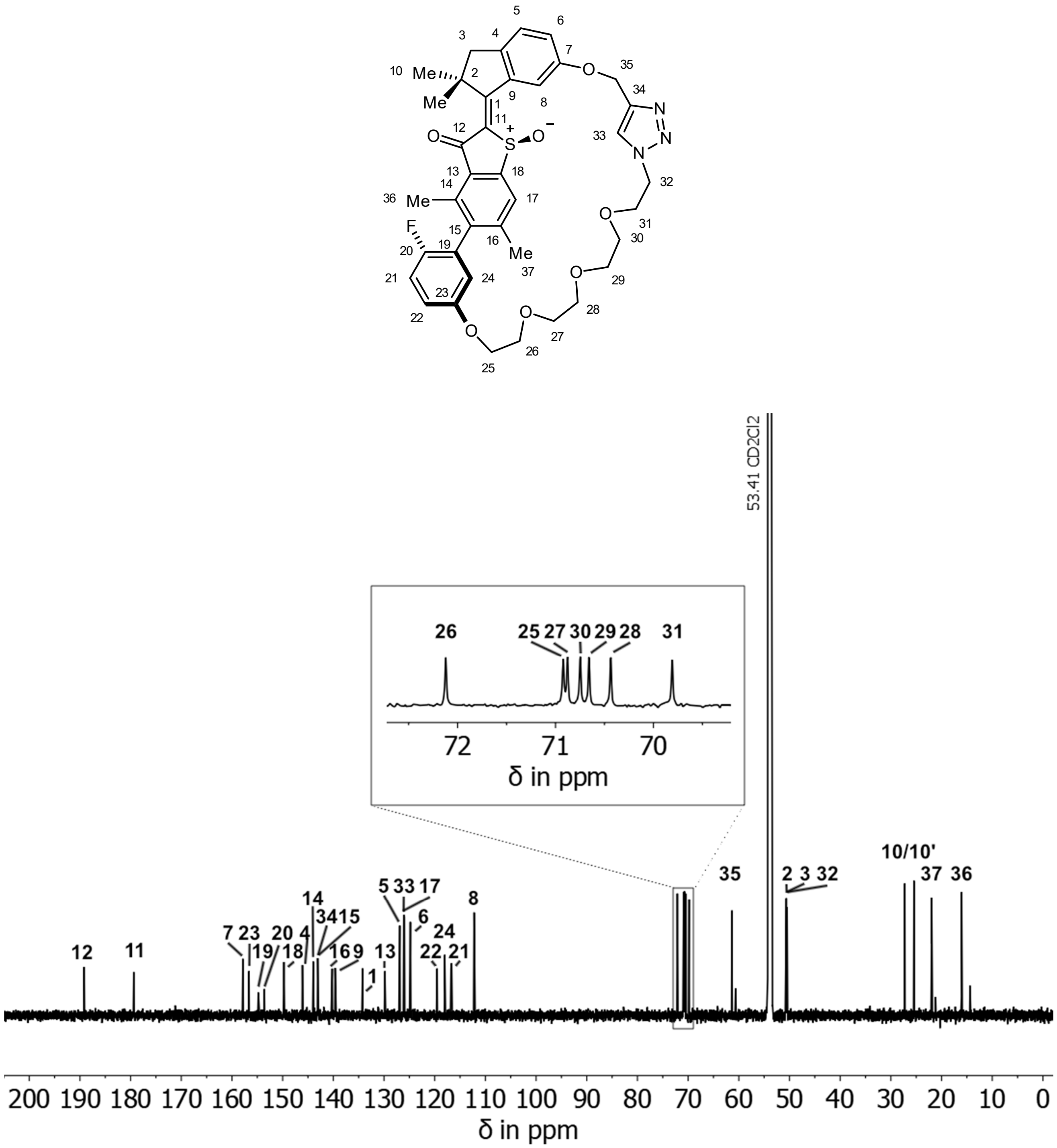

Supplementary Figure 13 **^{13}C NMR spectrum (CD_2Cl_2, 201 MHz, 25 °C) of racemic isomer 2-*Z*-I.** Arbitrary given numbers 1-35 were used for assignment of signals.

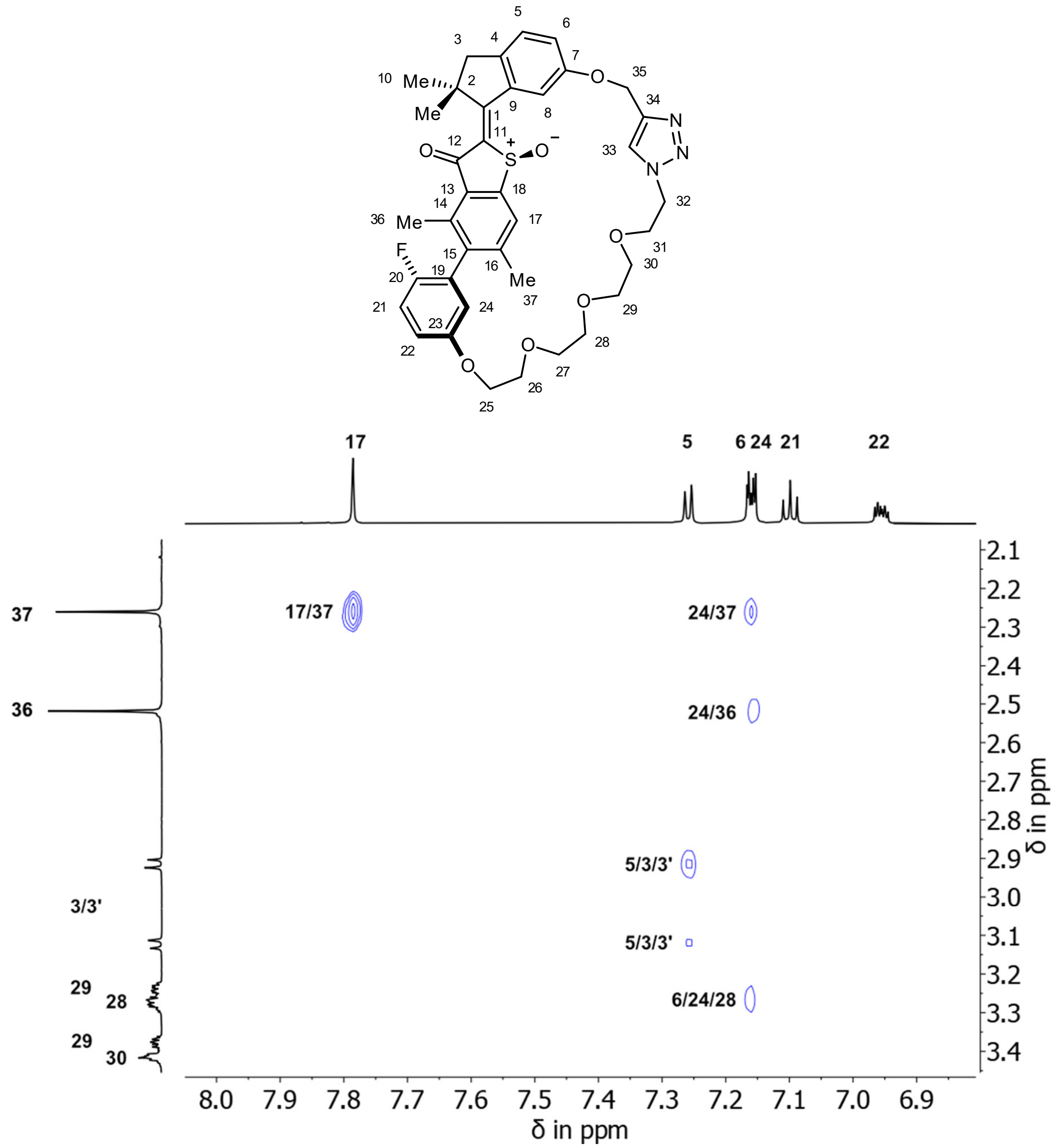

Supplementary Figure 14 NOESY NMR spectrum (CD_2Cl_2, 800 MHz, 25 °C) of isomer 2-*Z*-I evidencing biaryl axis tilt. Intensity difference between cross signals (blue) of aromatic methyl groups H_3-C36 and H_3-C37 to the aromatic proton H-C24 indicate the two aromatic planes are non-orthogonal. The stronger cross signal between proton H-C24 and methyl group H_3-C37 suggests a stronger preferred tilt of the biaryl, which brings aromatic proton H-C24 (and thus also anchoring point of the covalent linker C23 towards the rest of the chain) closer to H_3-C37.

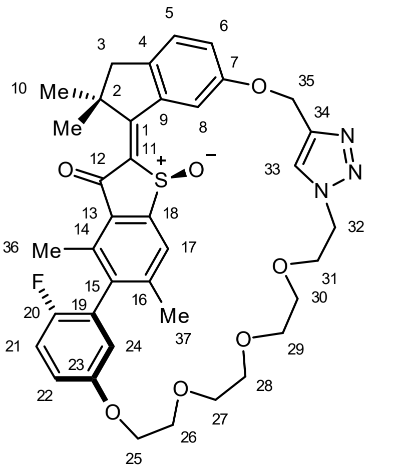

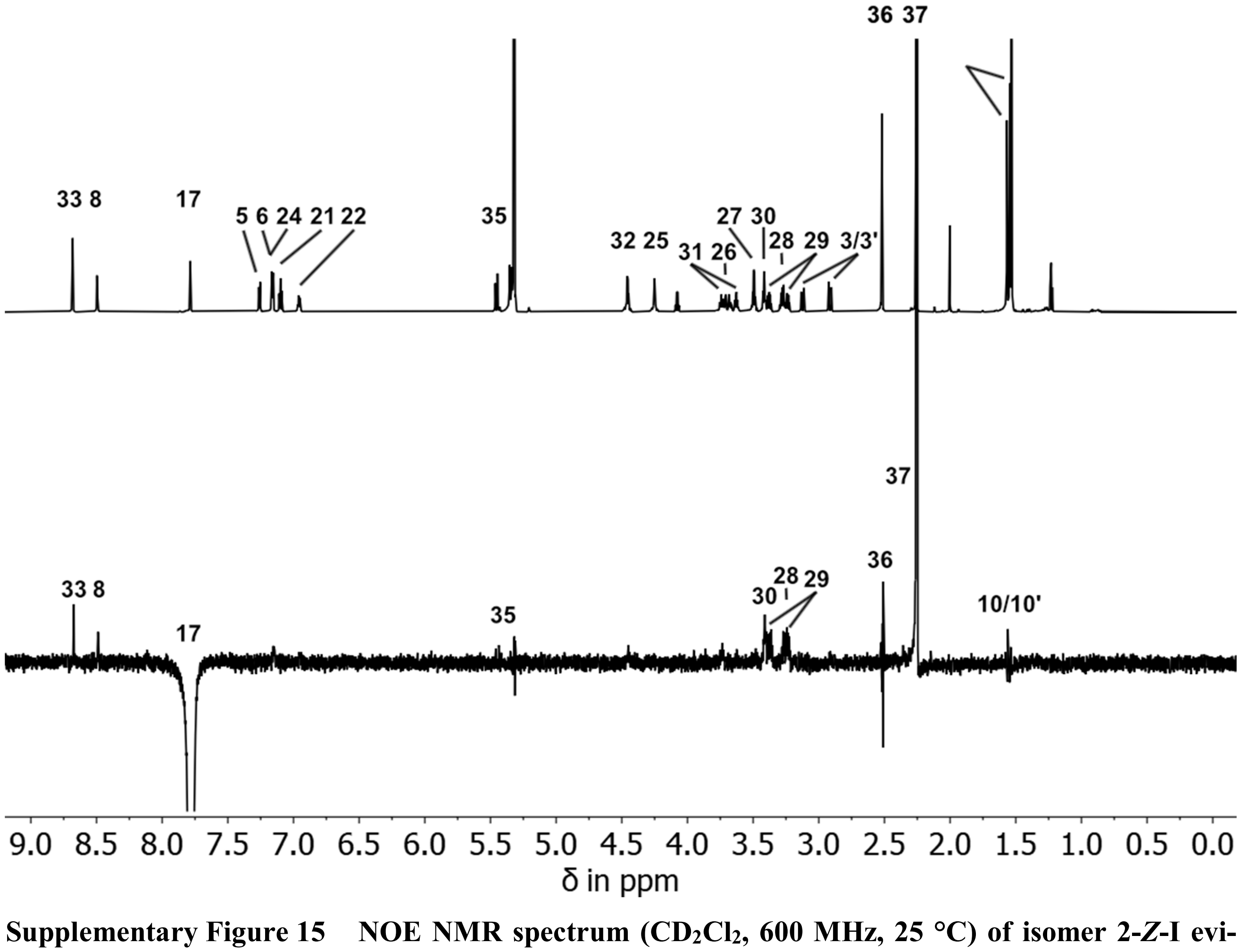

Supplementary Figure 15 NOE NMR spectrum (CD_2Cl_2, 600 MHz, 25 °C) of isomer 2-*Z*-I evidencing *Z*-configuration. Double bond configuration was assigned by coupling of aromatic proton H-C17 to aromatic triazole proton H-C33 and aromatic proton H-C8.

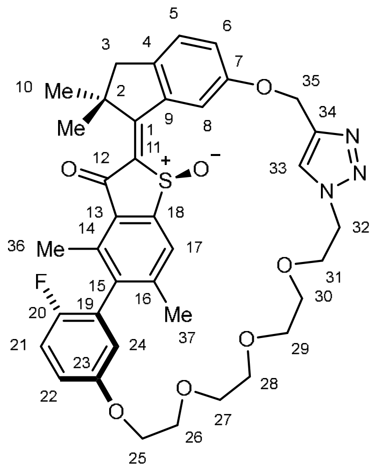

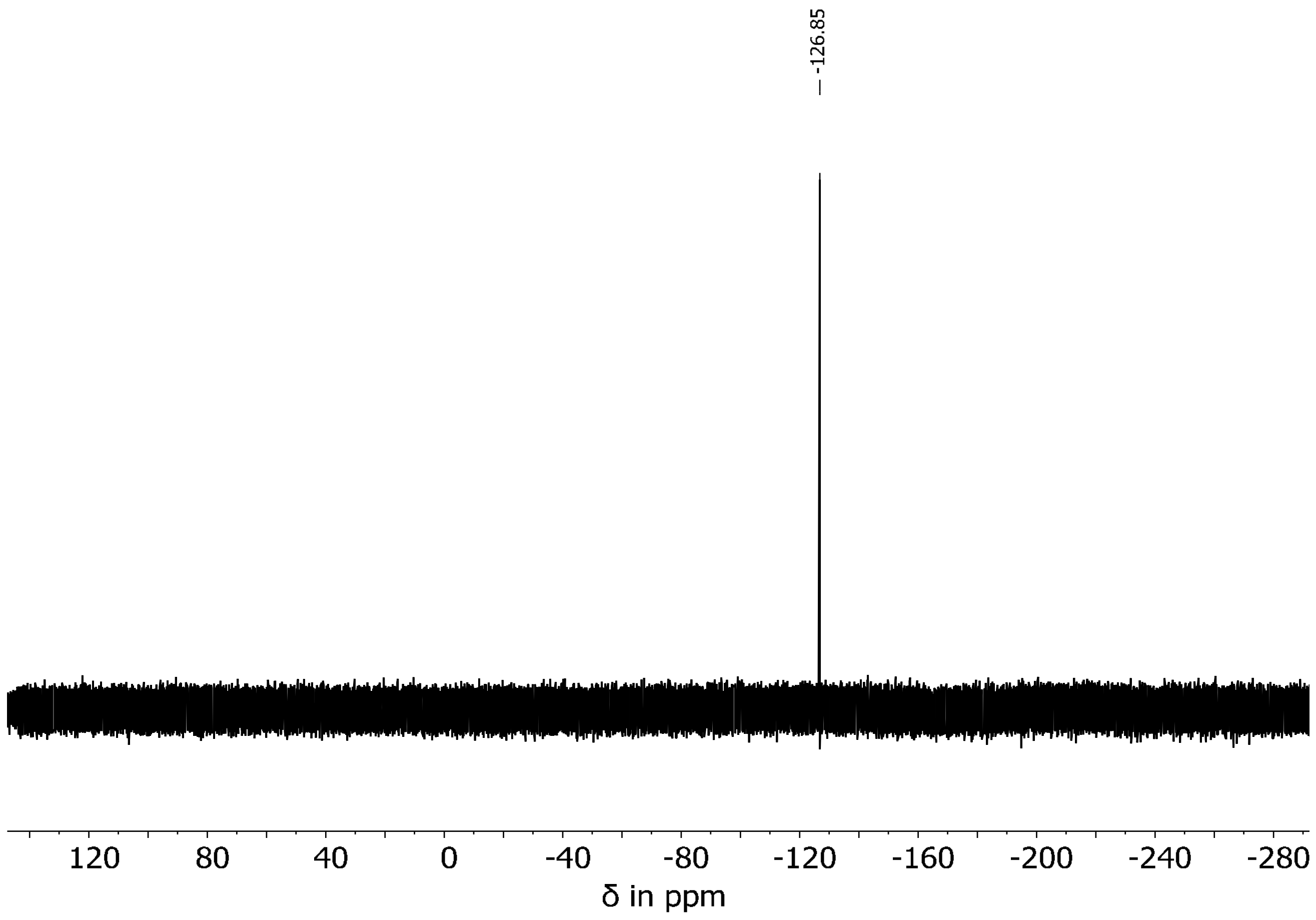

Supplementary Figure 16 **^{19}F NMR spectrum (CD_2Cl_2, 377 MHz, 25 °C) of racemic isomer 2-*Z*-I.** One distinct signal at –126.85 ppm is observed.

Isomer 2-*E*-II

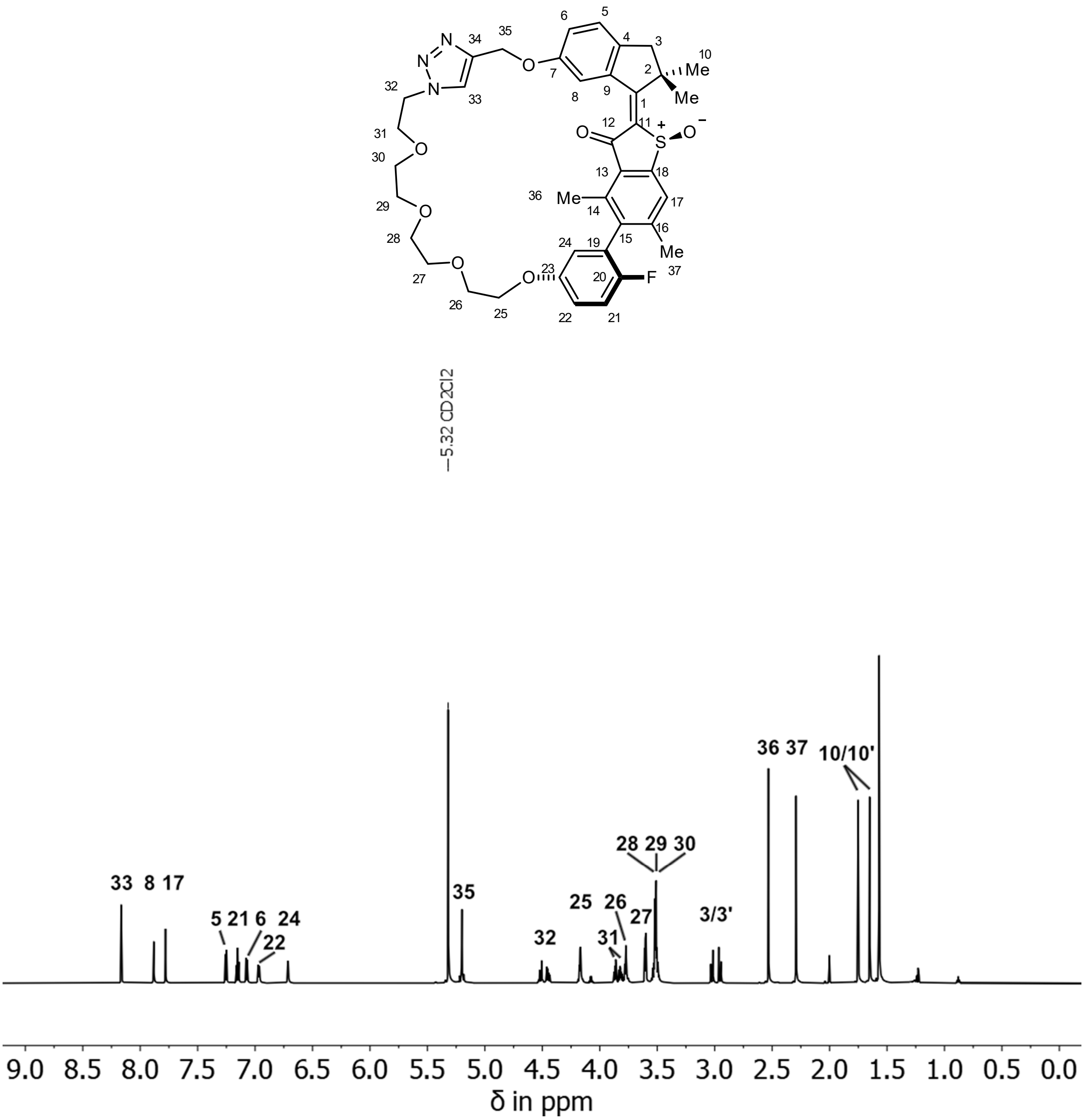

Supplementary Figure 17 **^{1}H NMR spectrum (CD_2Cl_2, 600 MHz, 25 °C) of racemic isomer 2-*E*-II.** Arbitrary given numbers 1-35 were used for assignment of signals.

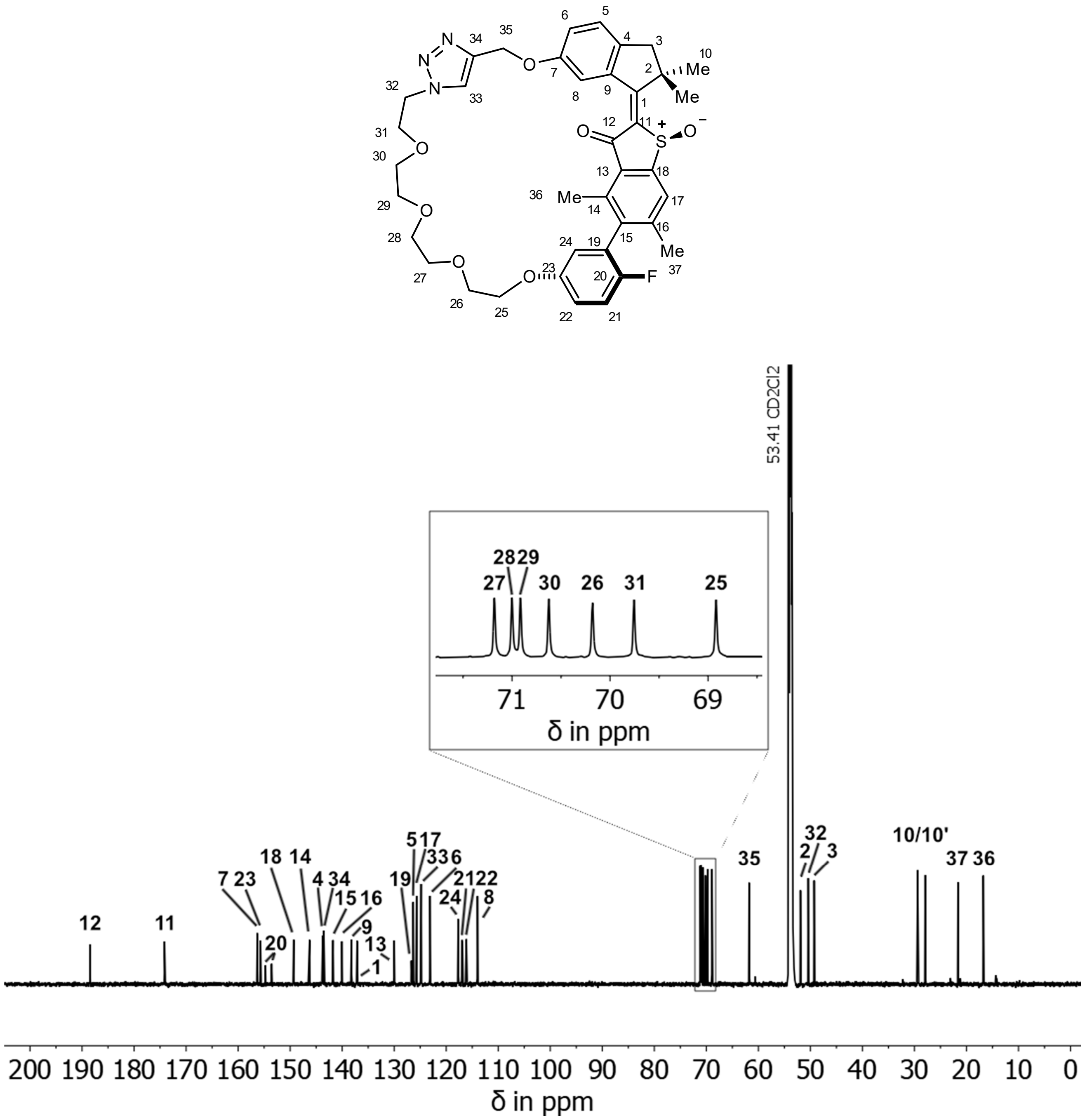

Supplementary Figure 18 **^{13}C NMR spectrum (CD_2Cl_2, 201 MHz, 25 °C) of racemic isomer 2-*E*-II.** Arbitrary given numbers 1-35 were used for assignment of signals.

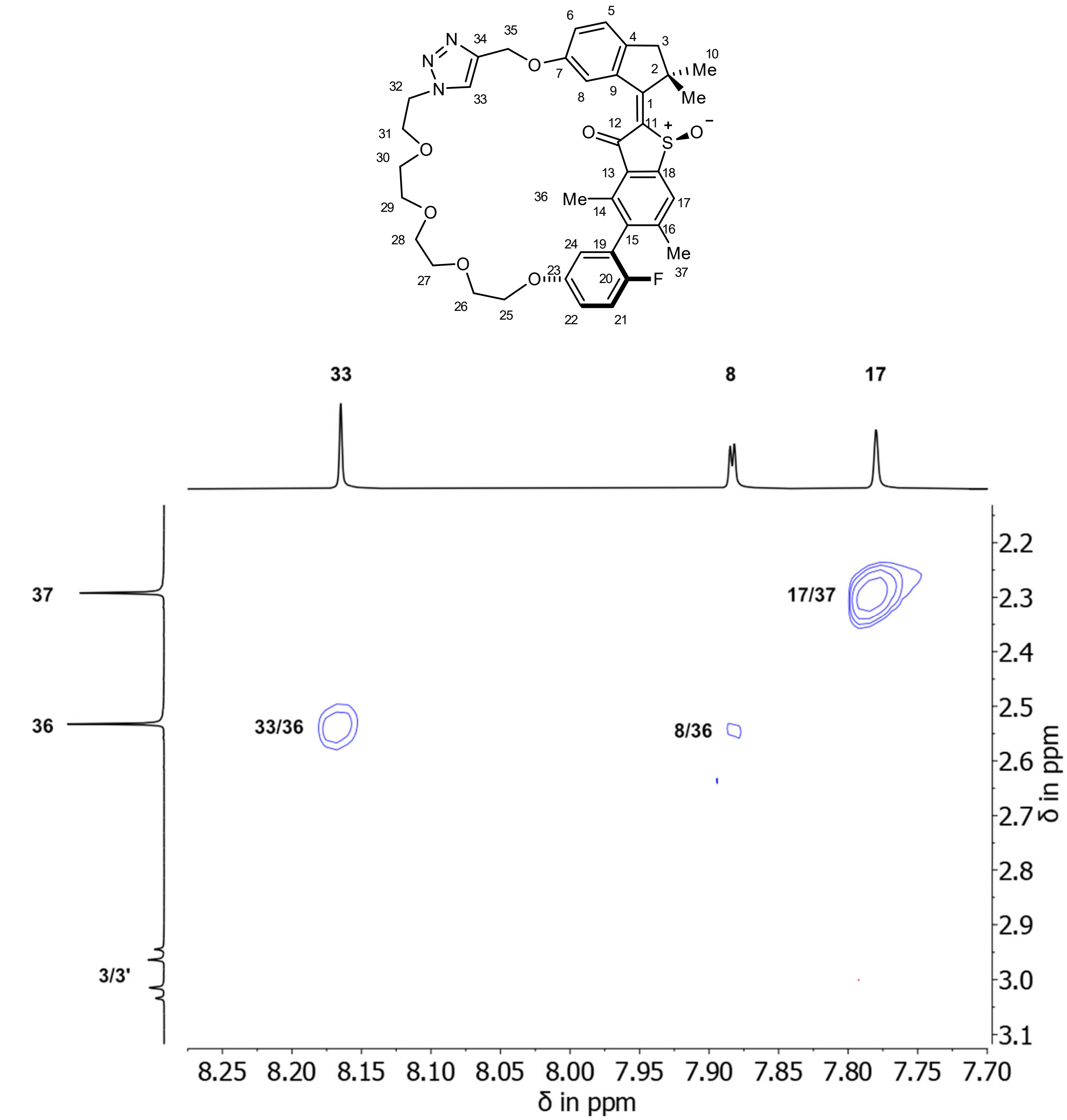

Supplementary Figure 19 NOESY NMR spectrum of 2-*E*-II (CD_2Cl_2, 800 MHz, 25 °C) evidencing *E*-configuration. Double bond configuration was assigned through a strong cross signal (blue) between methyl group H_3-C36 and the aromatic triazole proton H-C33 as well as aromatic proton H-C8 and absence of a cross signal between methyl group H_3-C37 and aromatic triazole proton H-C33.

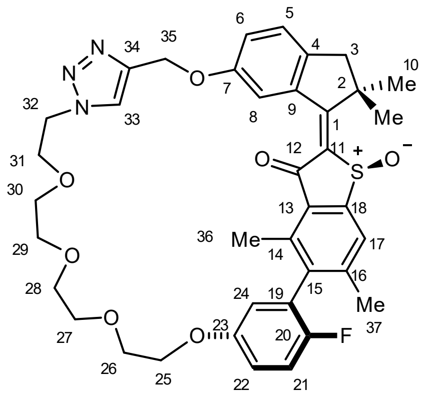

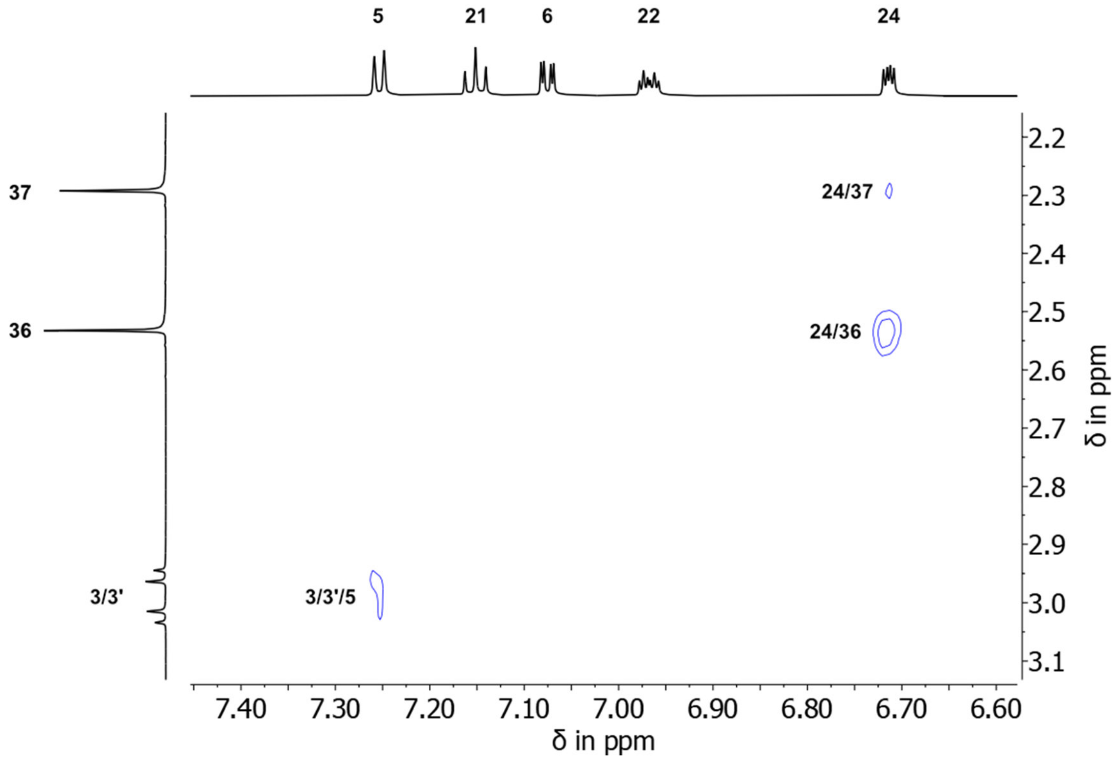

Supplementary Figure 20 NOESY NMR spectrum (CD_2Cl_2, 800 MHz, 25 °C) of isomer 2-*E*-II evidencing biaryl axis tilt. Intensity difference between cross signals (blue) of aromatic methyl groups H_3-C36 and H_3-C37 to the aromatic proton H-C24 indicate the two aromatic planes are non-orthogonal. The stronger cross signal between proton H-C24 and methyl group H_3-C36 suggests a stronger preferred tilt of the biaryl, which brings aromatic proton H-C24 (and thus also anchoring point of the covalent linker C23 towards the rest of the chain) closer to H_3-C36.

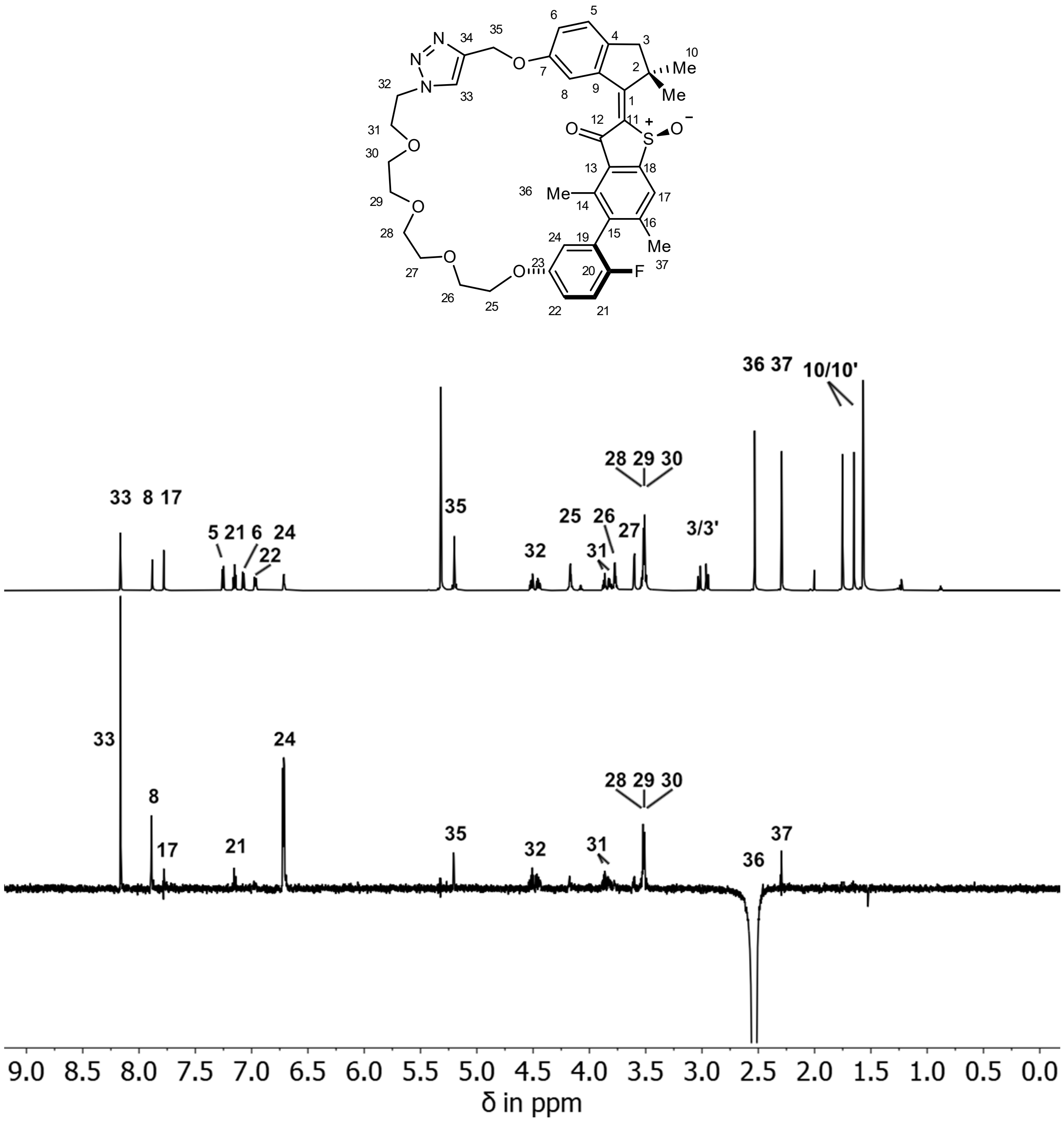

Supplementary Figure 21 NOE NMR spectrum (CD_2Cl_2, 600 MHz, 25 °C) of isomer 2-*E*-II evidencing *E*-configuration. Double bond configuration was assigned by coupling of aromatic proton H-C8 and aromatic triazole proton H-C33 to aromatic methyl group H_3-C36.

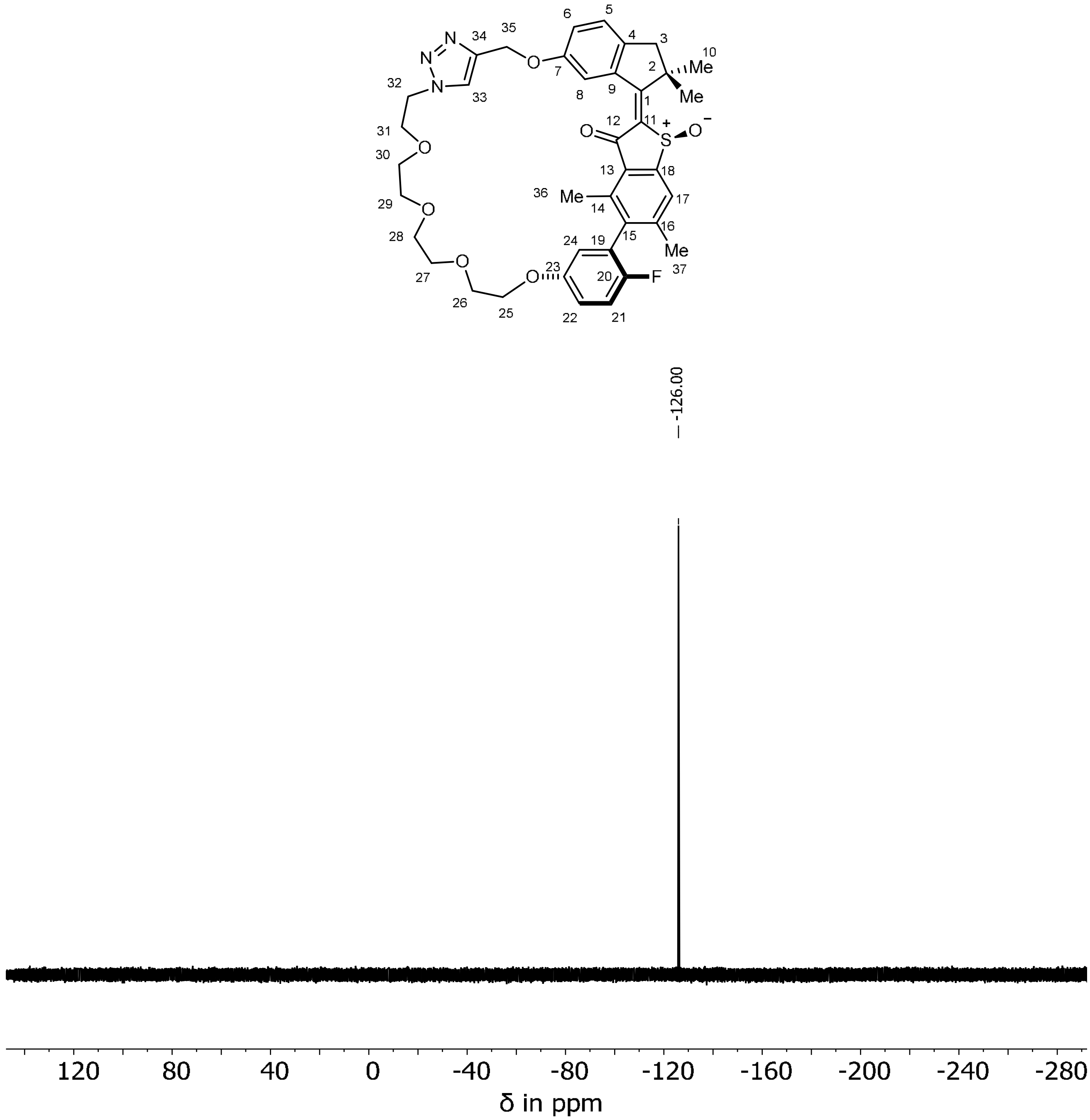

Supplementary Figure 22 **^{19}F NMR spectrum (CD_2Cl_2, 377 MHz, 25 °C) of racemic isomer 2-*E*-II**

One distinct signal at –126.00 ppm is observed.

Isomer 2-*Z*-II

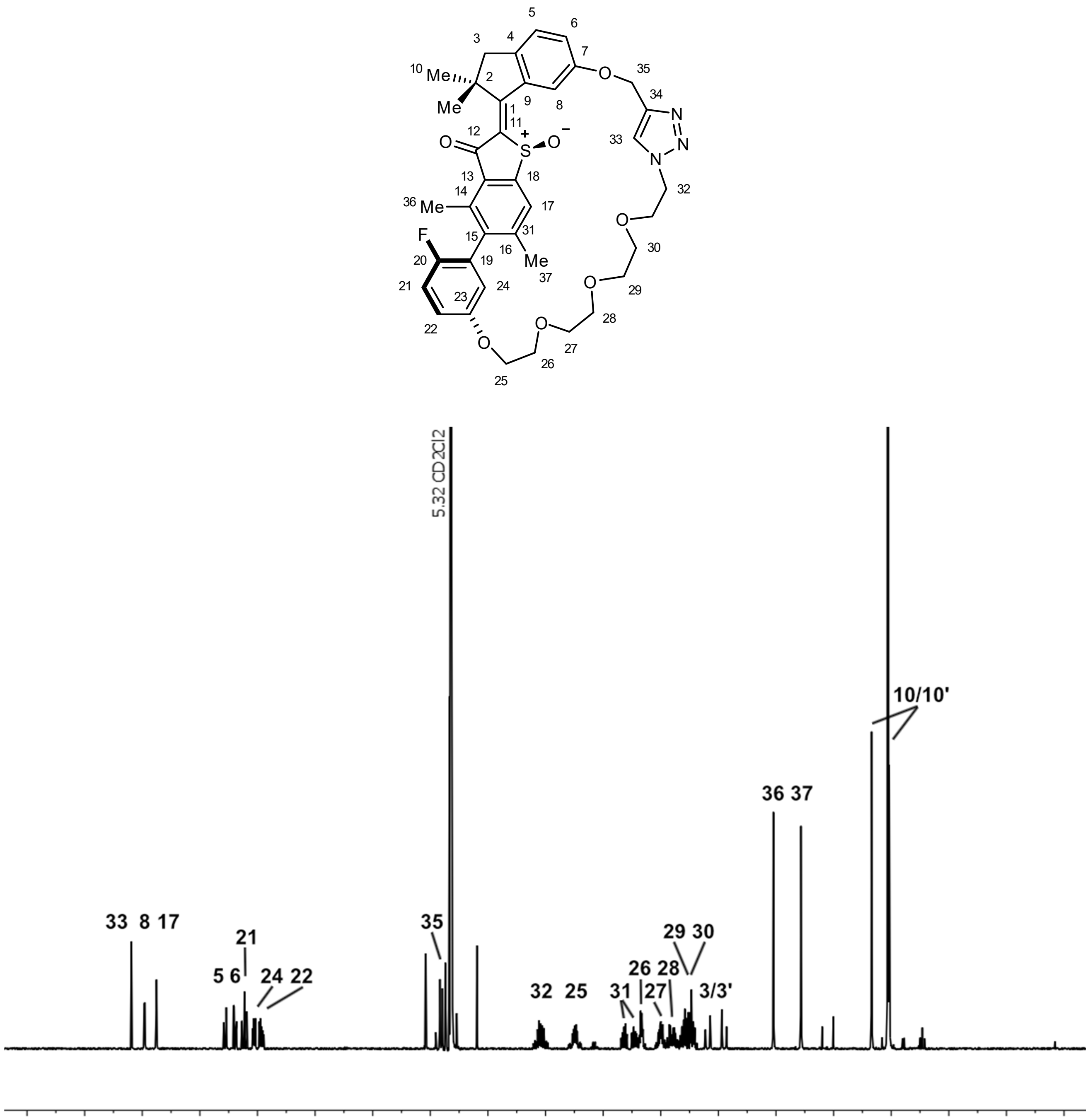

Supplementary Figure 23 **^{1}H NMR spectrum (CD_2Cl_2, 400 MHz, 25 °C) of racemic isomer 2-*Z*-II.** Arbitrary given numbers 1-35 were used for assignment of signals.

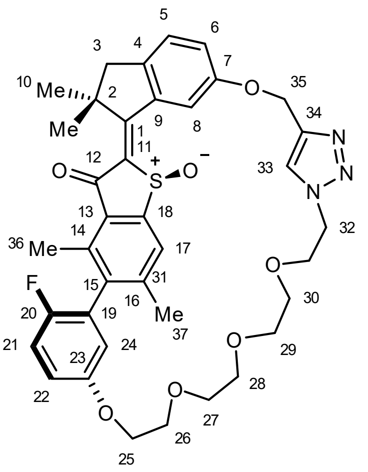

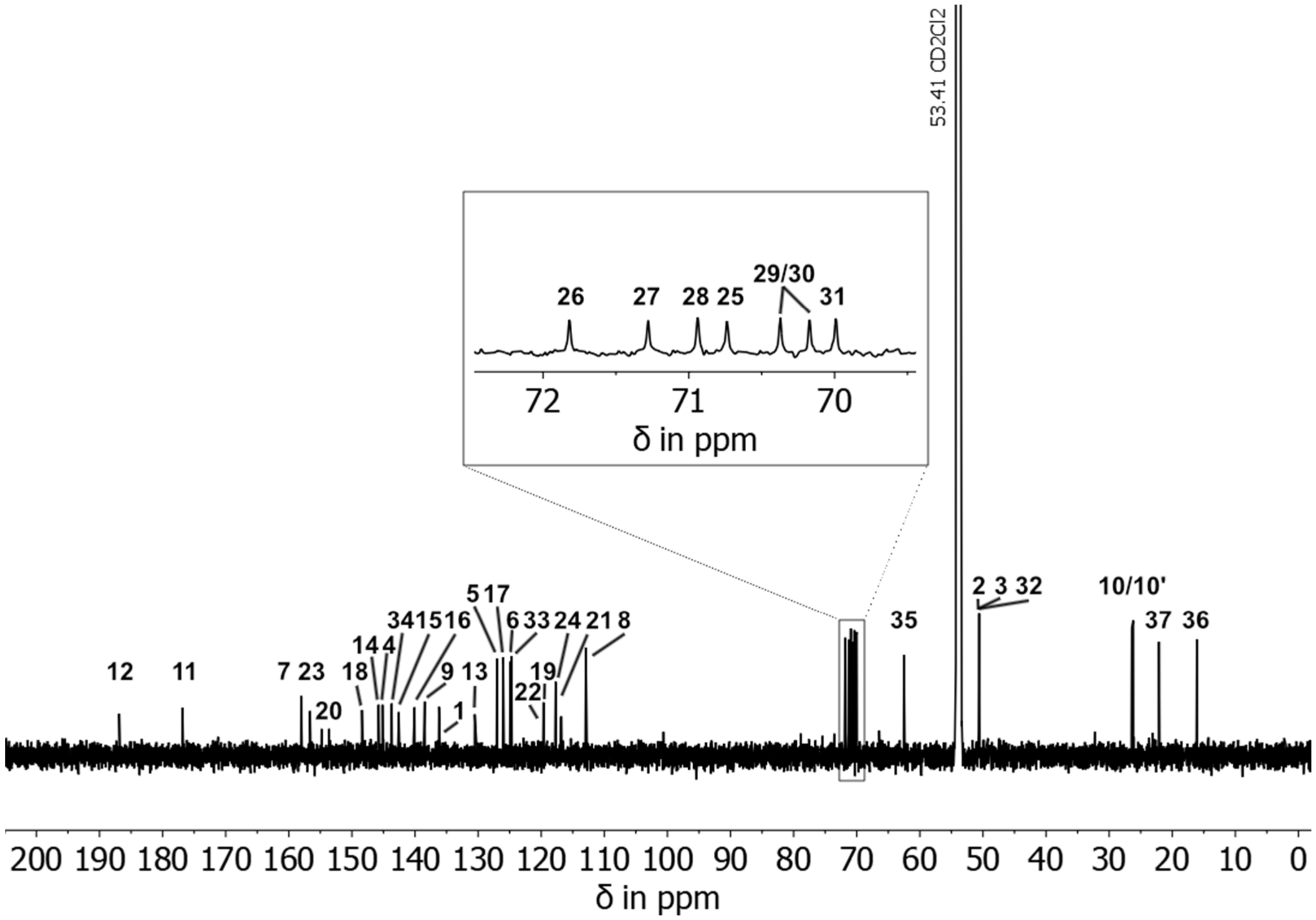

Supplementary Figure 24 **^{13}C NMR spectrum (CD_2Cl_2, 201 MHz, 25 °C) of racemic isomer 2-*Z*-II.** Arbitrary given numbers 1-35 were used for assignment of signals.

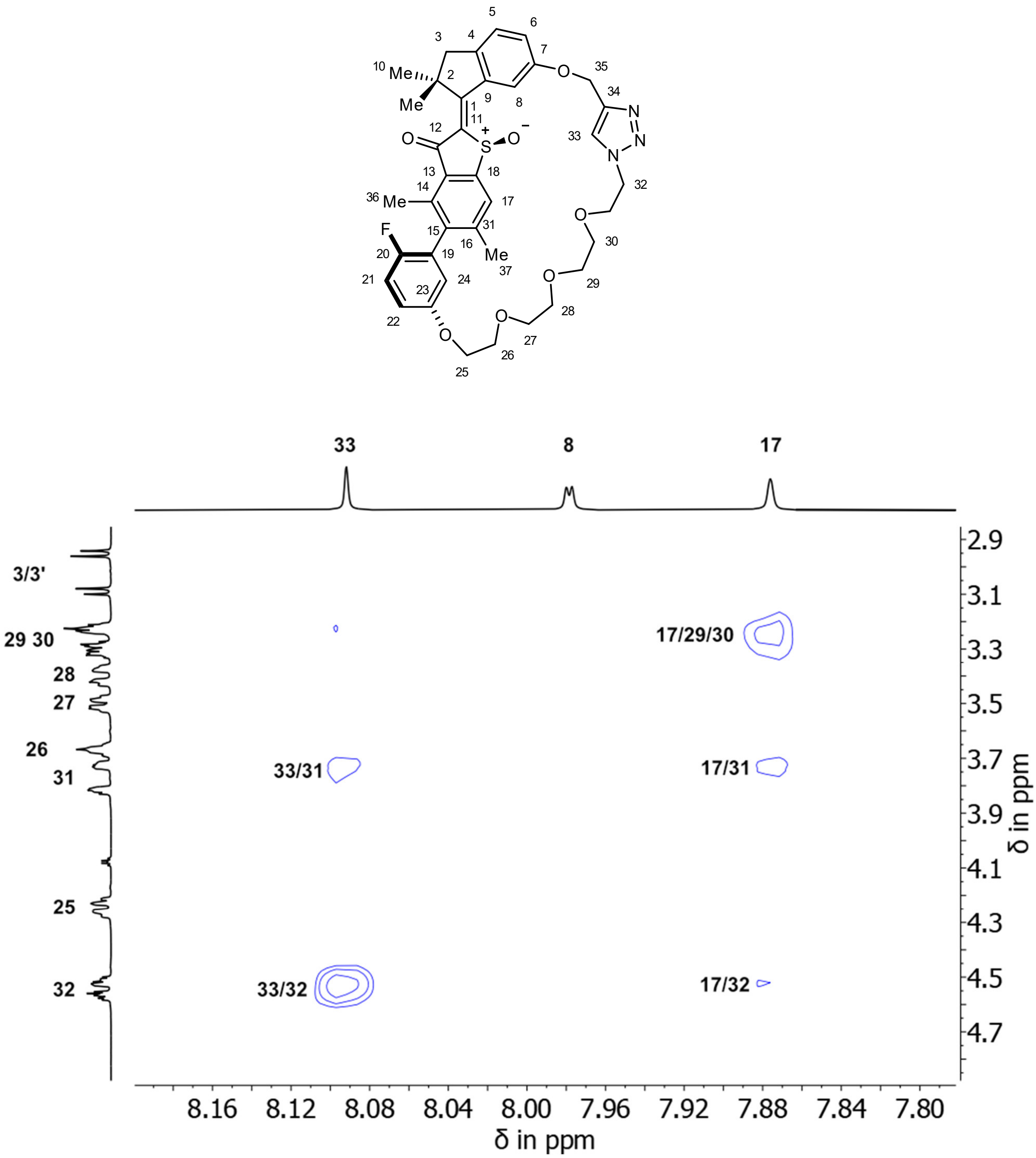

Supplementary Figure 25 NOESY NMR spectrum of 2-*Z*-II (CD_2Cl_2, 800 MHz, 25 °C) evidencing *Z*-configuration. Double bond configuration was assigned through multiple cross signals (blue) between aromatic proton H-C17 and protons H_2-C29, H_2-C30, H_2-C31 and H_2-C32 of the covalent linker.

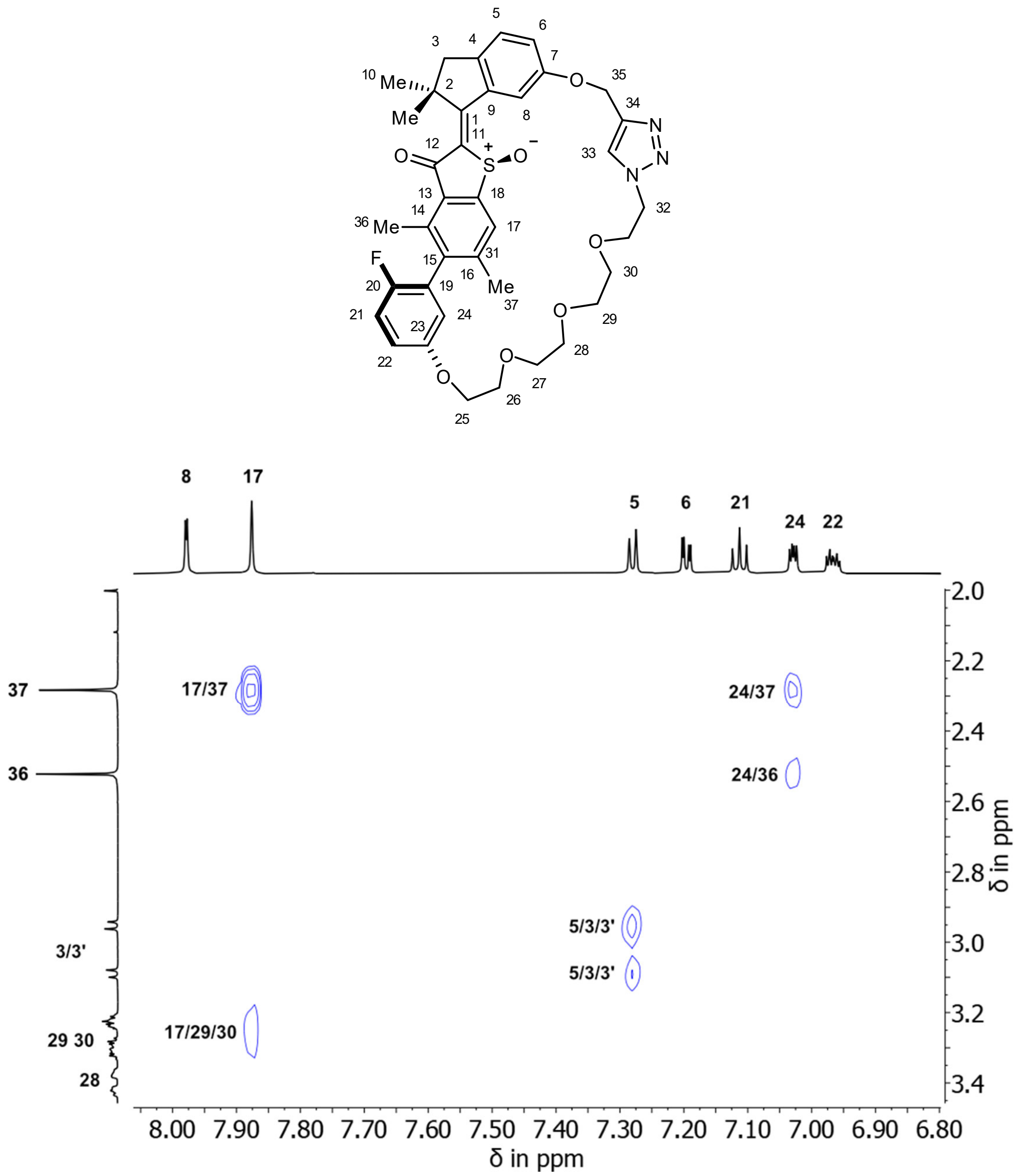

Supplementary Figure 26 NOESY NMR spectrum (CD_2Cl_2, 800 MHz, 25 °C) of isomer 2-*Z*-II evidencing biaryl axis tilt. Intensity difference between cross signals (blue) of aromatic methyl groups H_3-C36 and H_3-C37 to the aromatic proton H-C24 indicate the two aromatic planes are non-orthogonal. The stronger cross signal between proton H-C24 and methyl group H_3-C37 suggests a stronger preferred tilt of the biaryl, which brings aromatic proton H-C24 (and thus also anchoring point of the covalent linker C23 towards the rest of the chain) closer to H_3-C37.

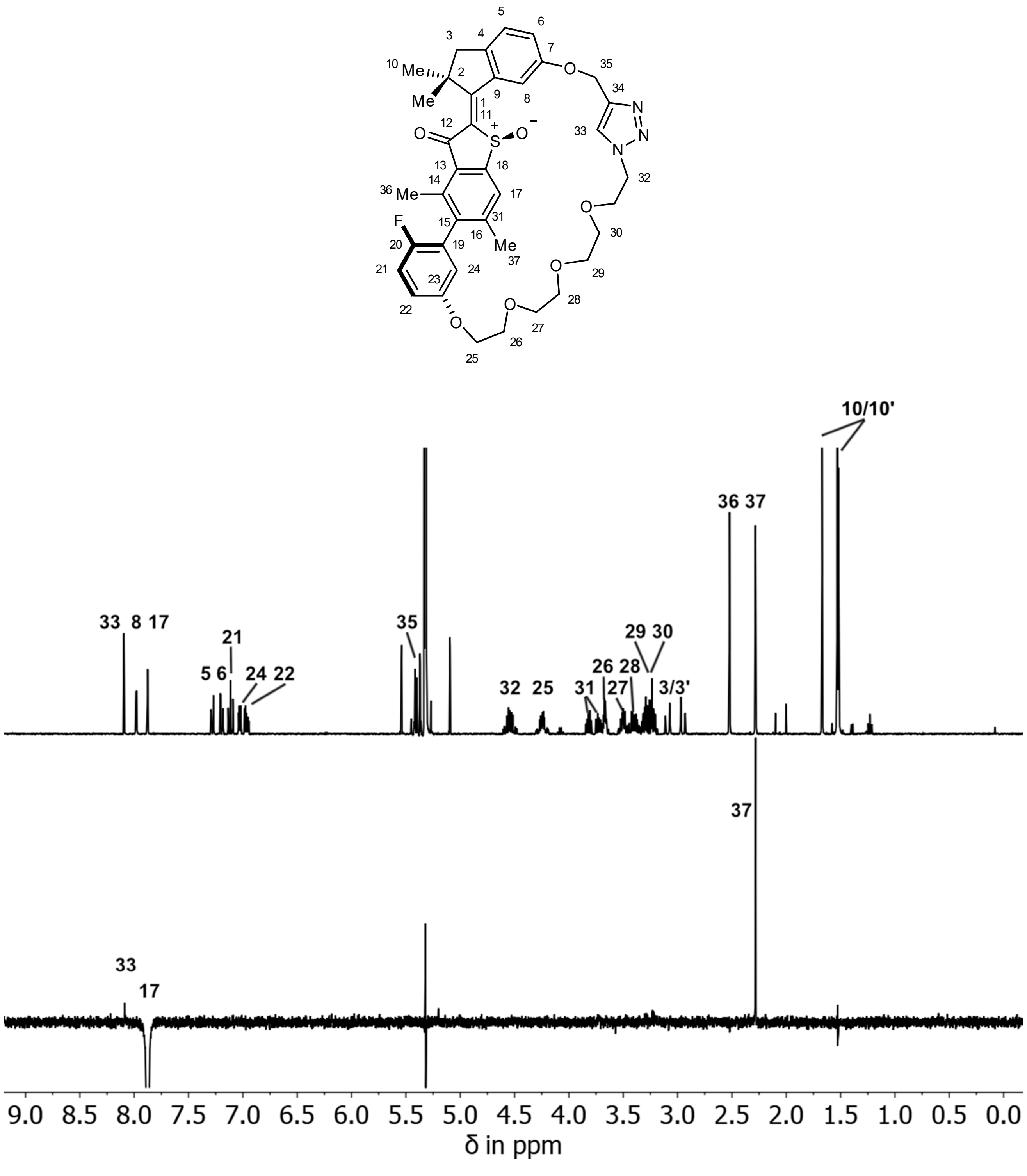

Supplementary Figure 27 NOE NMR spectrum (CD_2Cl_2, 600 MHz, 25 °C) of isomer 2-*Z*-II evidencing *Z*-configuration. Double bond configuration was assigned by coupling of aromatic proton H-C17 to aromatic triazole proton H-C33.

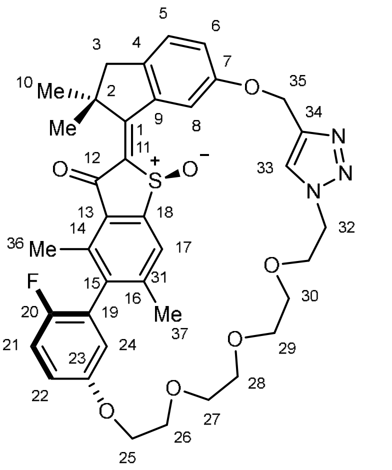

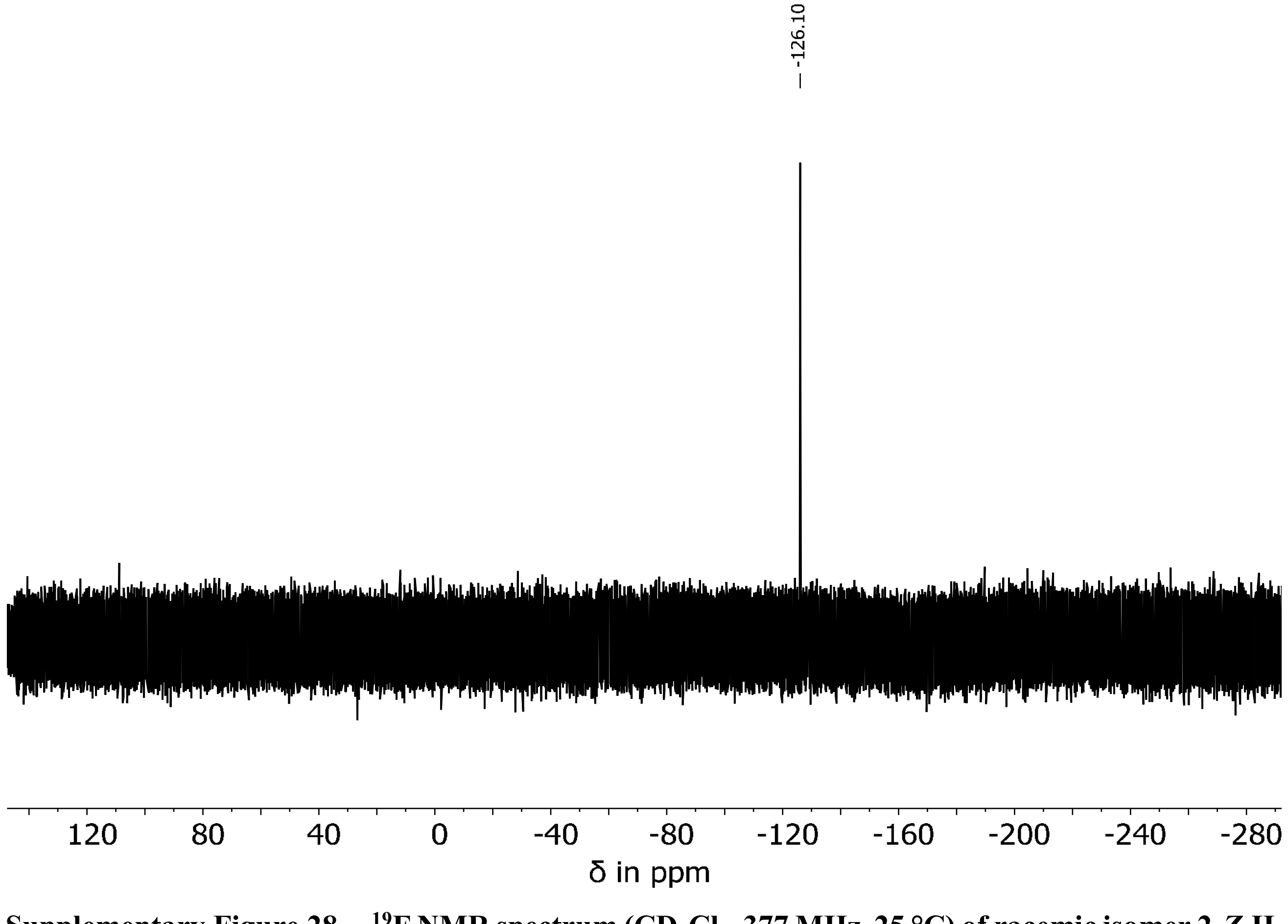

Supplementary Figure 28 **^{19}F NMR spectrum (CD_2Cl_2, 377 MHz, 25 °C) of racemic isomer 2-*Z*-II.** One distinct signal at –126.10 ppm is observed.

Structure of HTI 3 in Solution

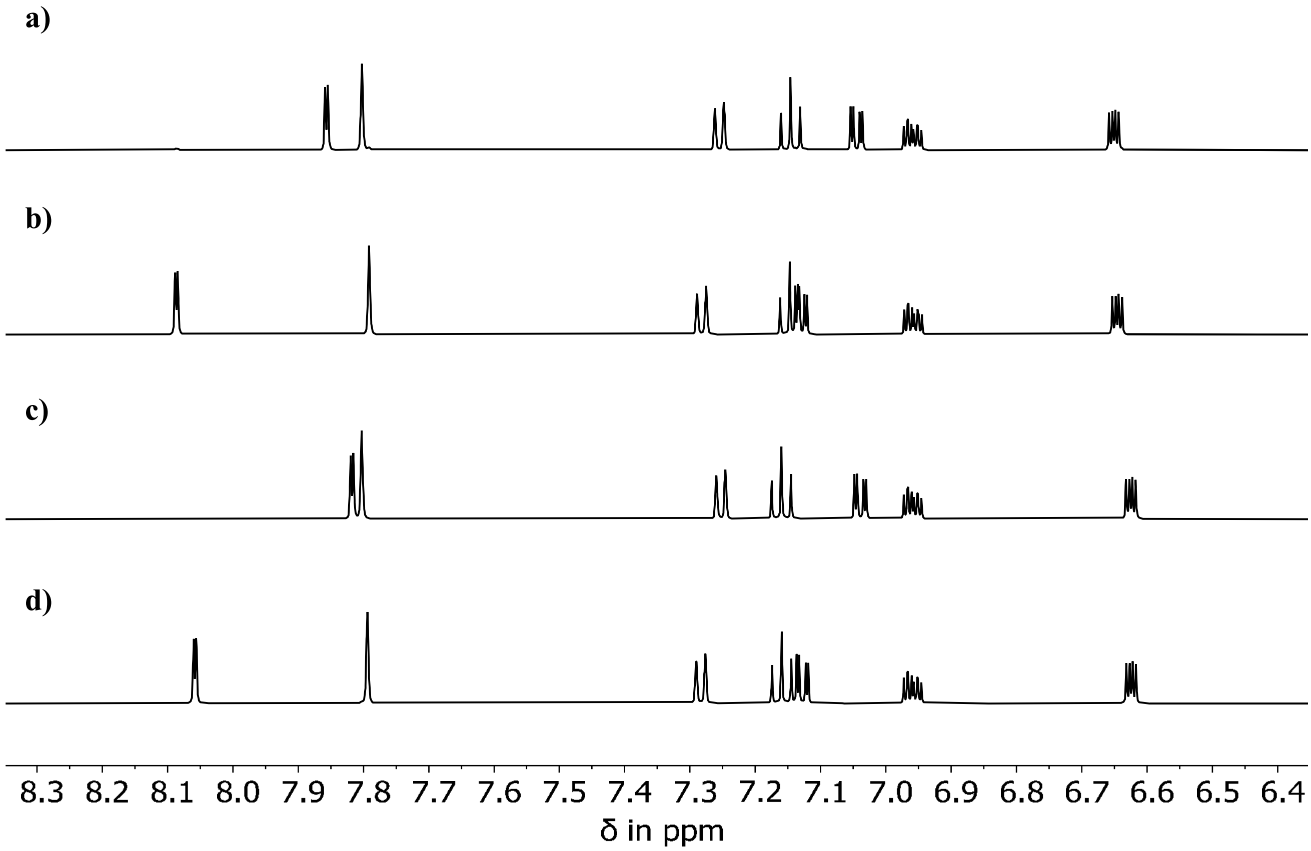

Supplementary Figure 29 Overview of ^{1}H NMR spectra of HTI 3. Aromatic region of ^{1}H NMR spectra (CD_2Cl_2, 400 MHz, 25 °C) of isomers a) **3**-(*E*)-(*S*)-(S_a), b) **3**-(*Z*)-(*S*)-(S_a), c) **3**-(*E)*-(*S*)-(R_a) and d) **3**-(*Z)*-(*S*)-(R_a) showing the most distinctly shifted signals.

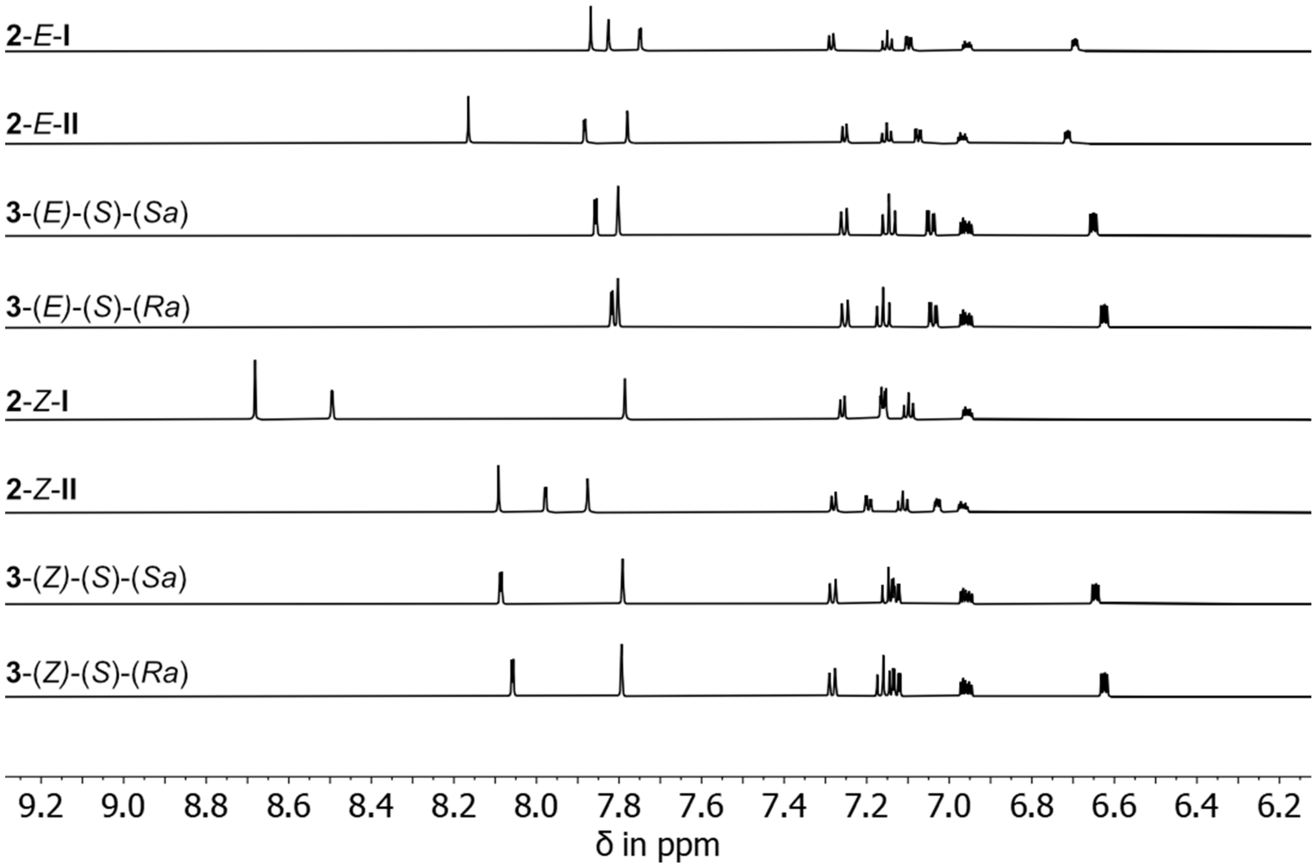

Supplementary Figure 30 Comparison of aromatic regions for HTIs 2 and 3. ^{1}H NMR spectra (CD_2Cl_2, 400 MHz, 25 °C) for macrocyclic isomers **2**-*E*-**I**, **2**-*E*-**II**, **2**-*Z*-**I** and **2**-*Z*-**II** with reference compound **3**-(*E*)-(*S*)-(S_a), **3**-(*E*)-(*S*)-(R_a), **3**-(*Z*)-(*S*)-(S_a) and **3**-(*Z*)-(*S*)-(R_a) showing the most distinctly shifted signals.

Isomer 3-(*E*)-(*S*)-(S_a)

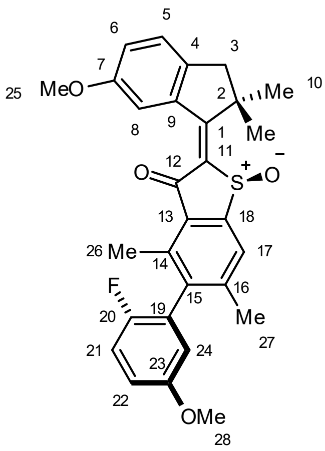

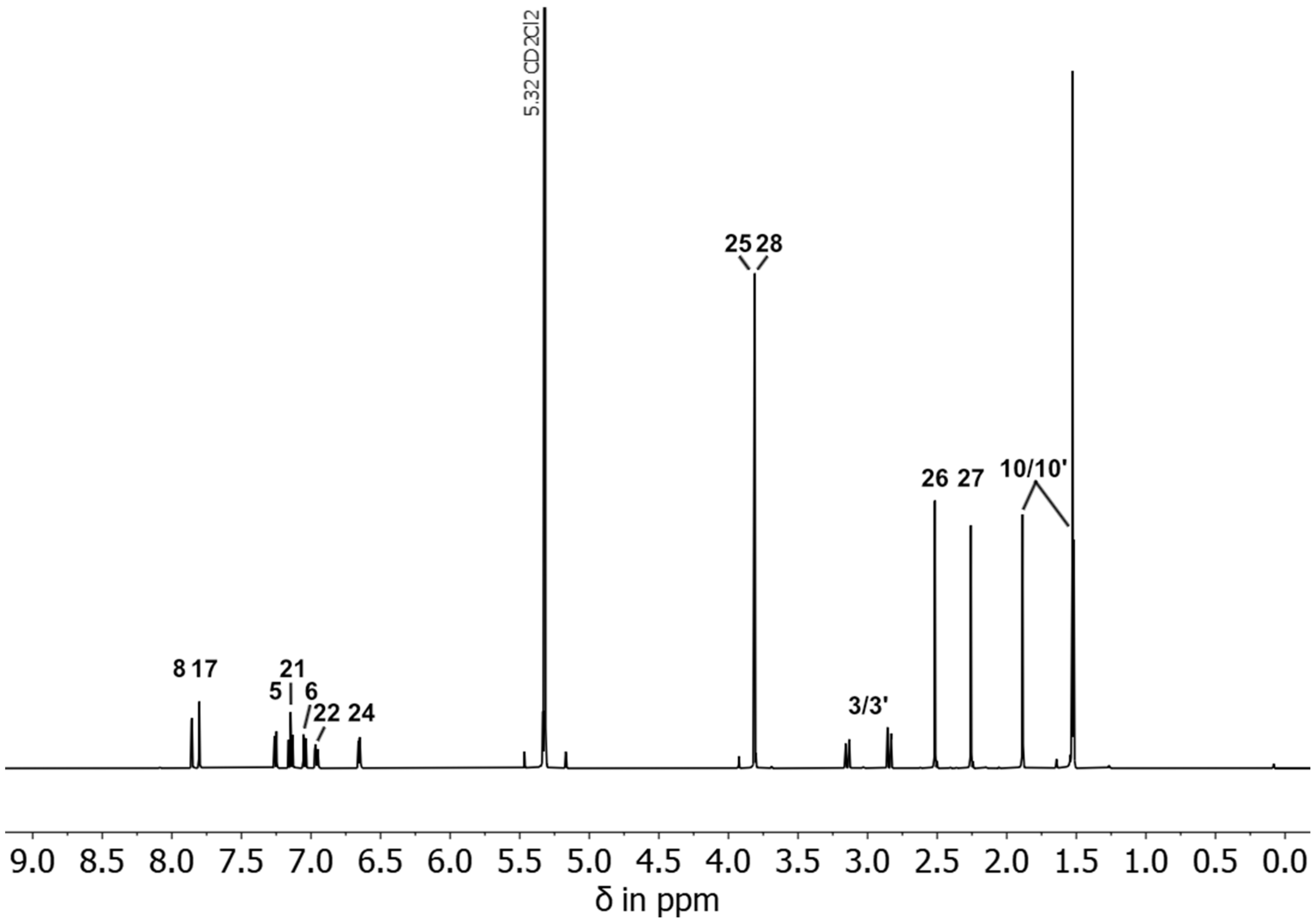

Supplementary Figure 31 **^{1}H NMR spectrum (CD_2Cl_2, 400 MHz, 25 °C) of racemic isomer 3-(*E*)-(*S*)-(S_a).** Arbitrary given numbers 1-28 were used for assignment of signals.

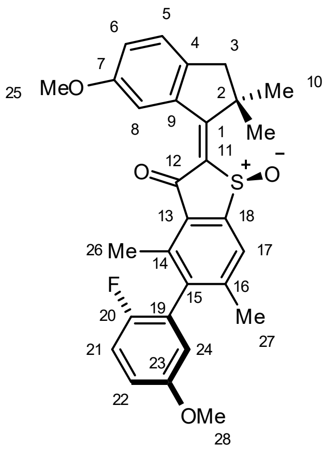

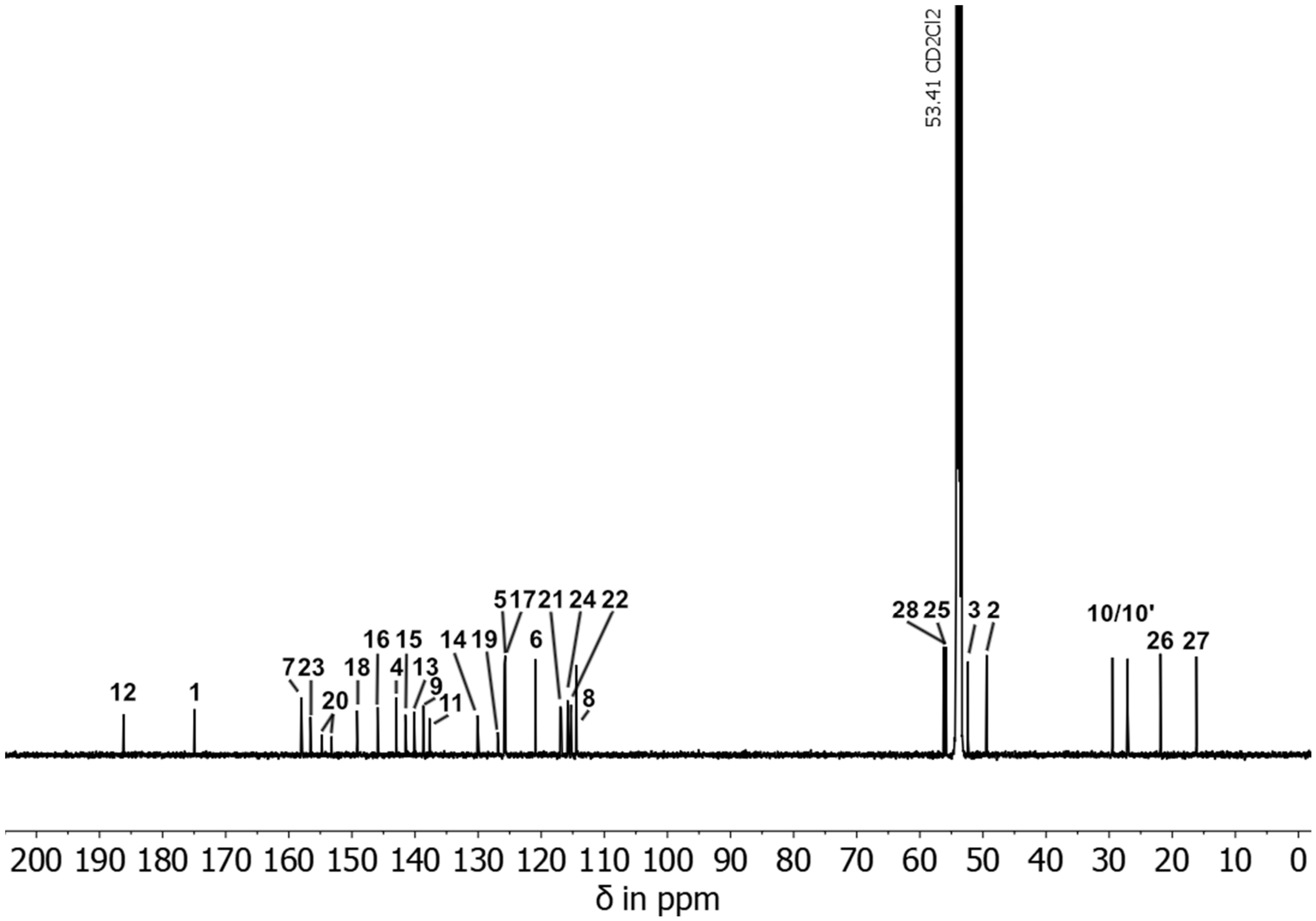

Supplementary Figure 32 **^{13}C NMR spectrum (CD_2Cl_2, 201 MHz, 25 °C) of racemic isomer 3-(*E*)-(*S*)-(S_a).** Arbitrary given numbers 1-28 were used for assignment of signals.

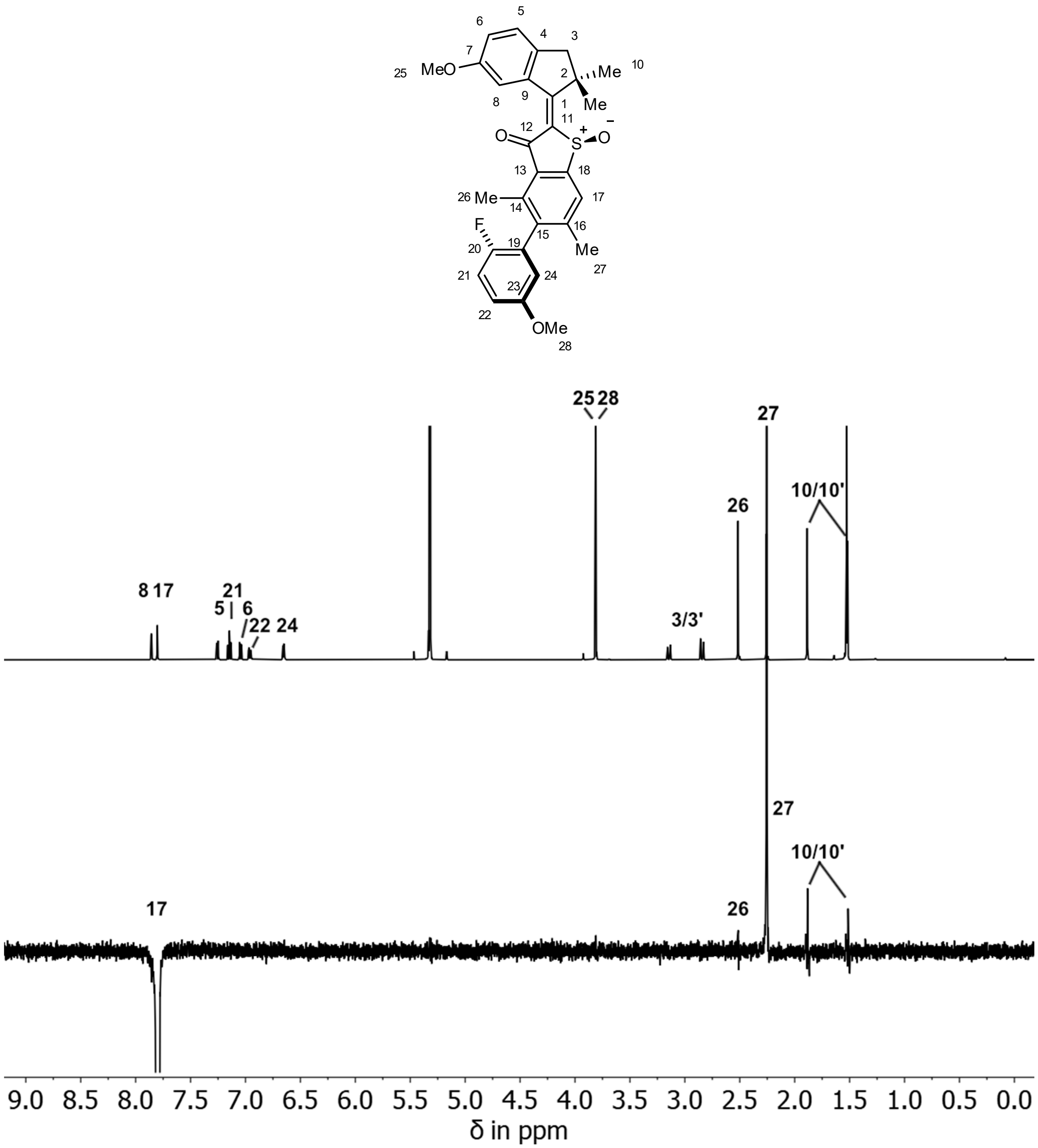

Supplementary Figure 33 NOE NMR spectrum (CD_2Cl_2, 600 MHz, 25 °C) of isomer 3-(*E*)-(*S*)-(*S*$_a$) evidencing *E*-configuration. Double bond configuration was assigned by coupling of aromatic proton H-C17 to methyl groups H_3-C10 and H_3-C10'.

Isomer 3-(*Z*)-(*S*)-(S_a)

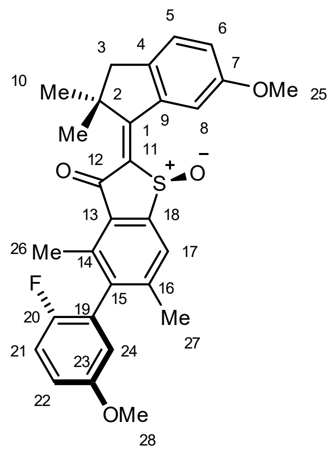

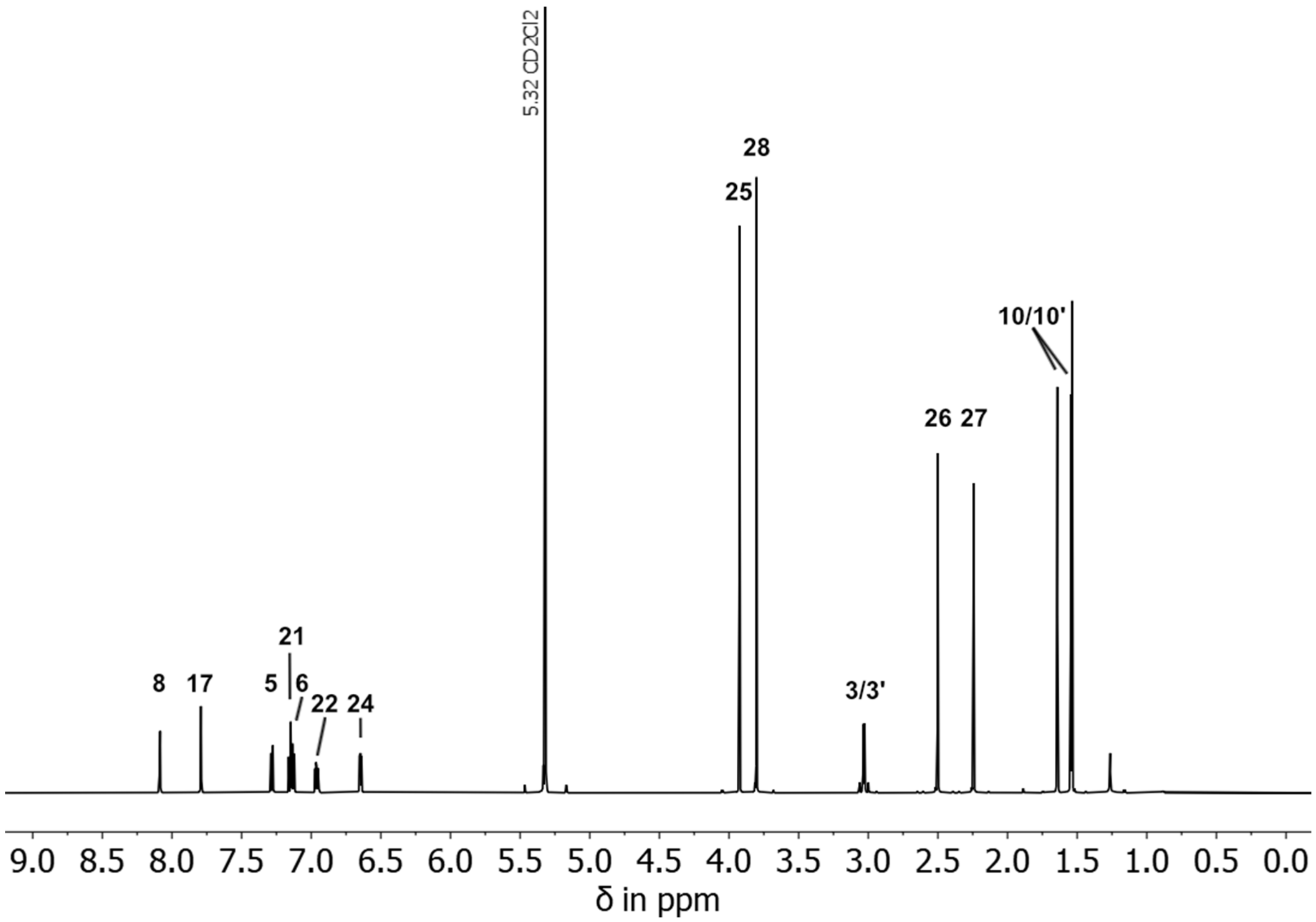

Supplementary Figure 34 **^{1}H NMR spectrum (CD_2Cl_2, 400 MHz, 25 °C) of racemic isomer 3-(*Z*)-(*S*)-(S_a).** Arbitrary given numbers 1-28 were used for assignment of signals.

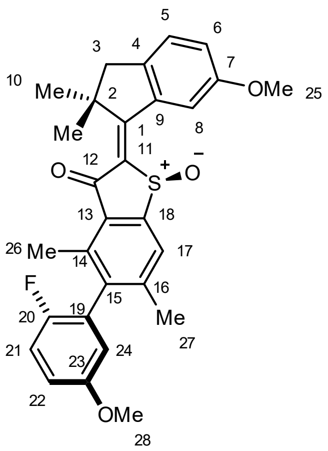

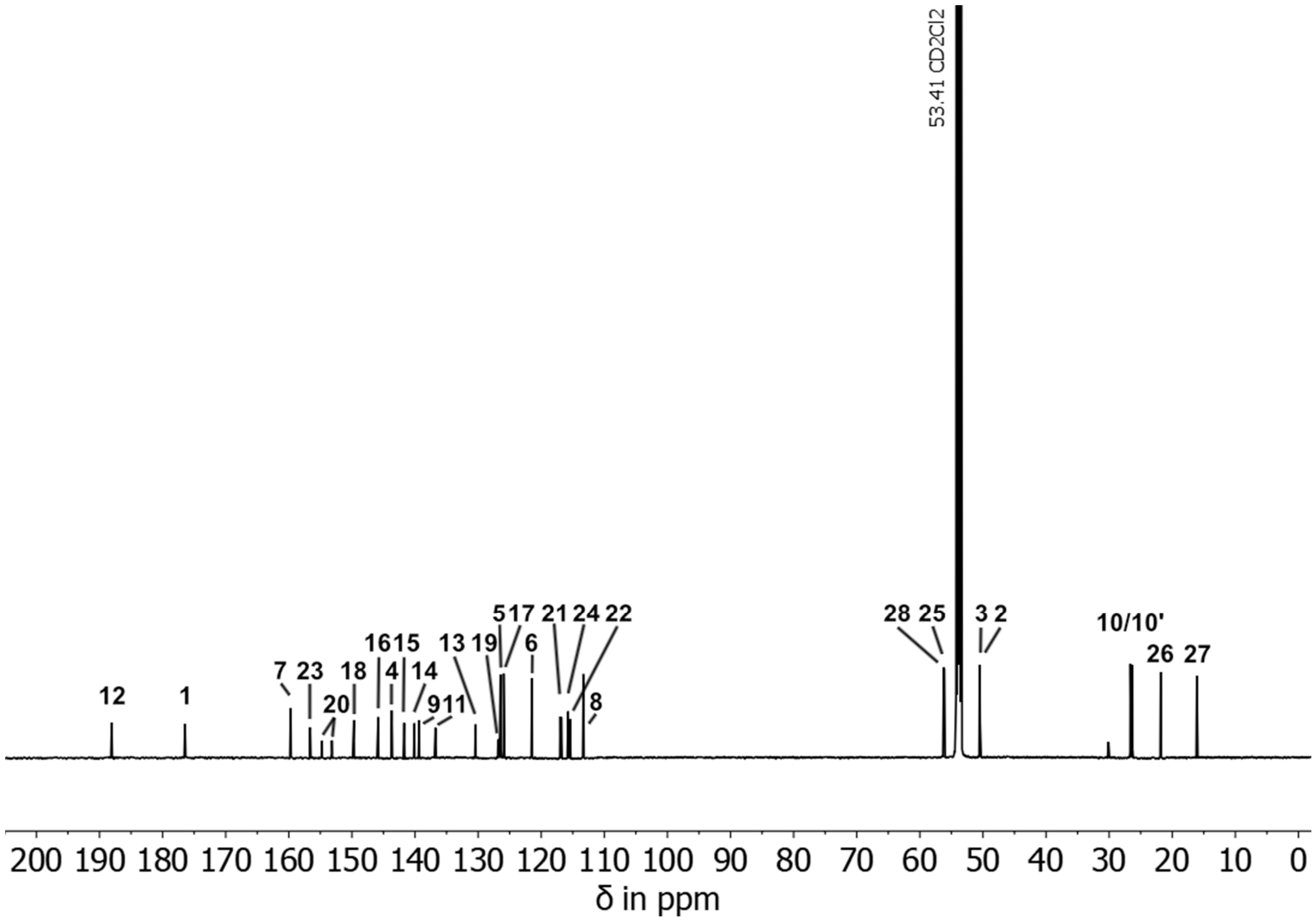

Supplementary Figure 35 **^{13}C NMR spectrum (CD_2Cl_2, 201 MHz, 25 °C) of racemic isomer 3-(*Z*)-(*S*)-(S_a).** Arbitrary given numbers 1-28 were used for assignment of signals.

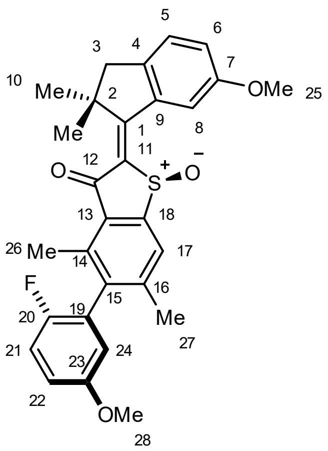

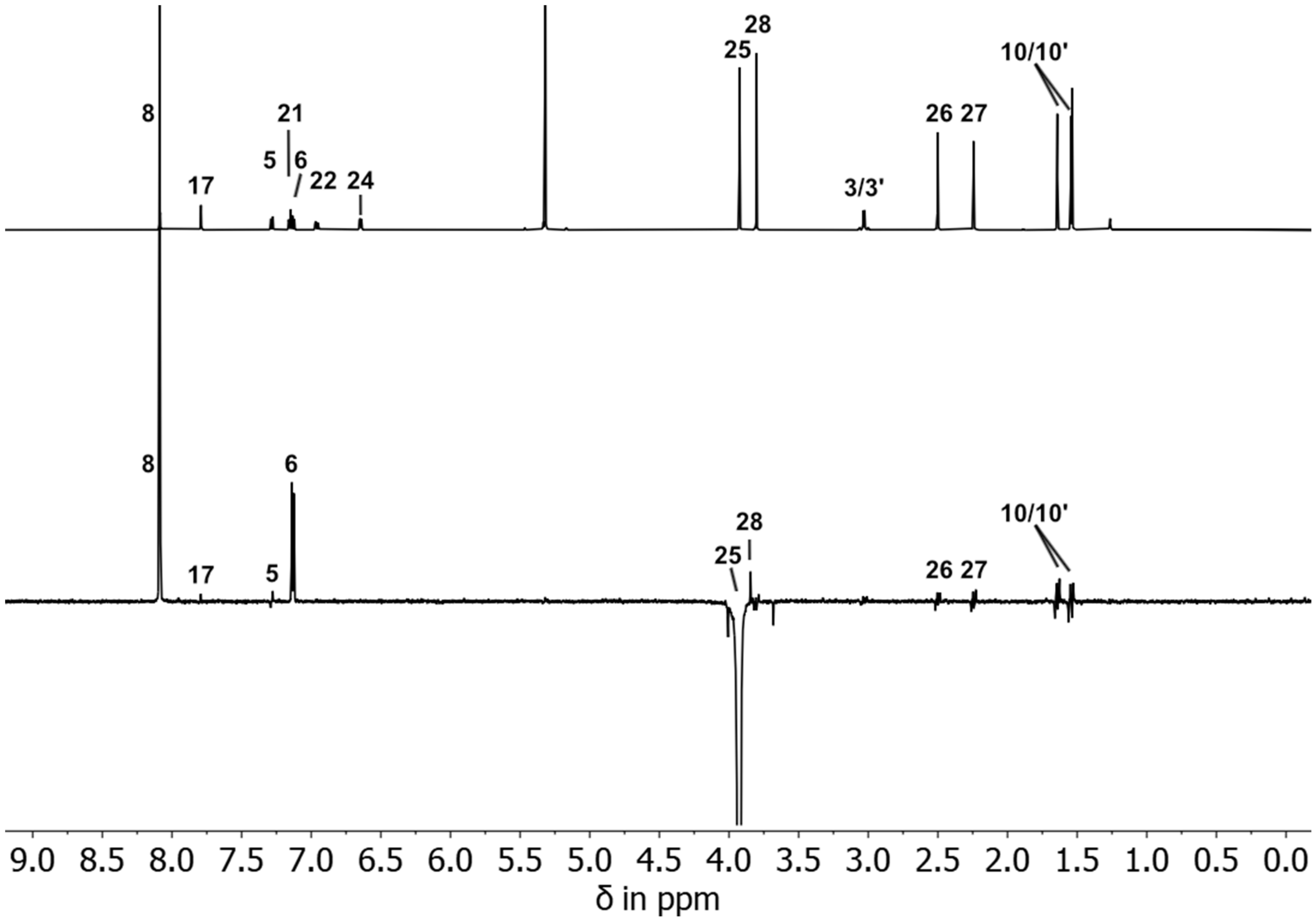

Supplementary Figure 36 NOE NMR spectrum (CD_2Cl_2, 600 MHz, 25 °C) of isomer 3-(*Z*)-(*S*)-(S_a) evidencing *Z*-configuration. Double bond configuration was assigned by coupling of methyl group H_3-C25 to aromatic proton H-C17.

Isomer 3-(*E*)-(*S*)-(*R*a)

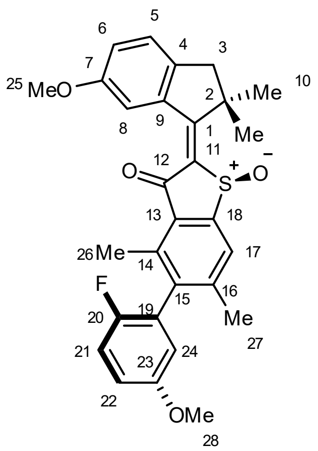

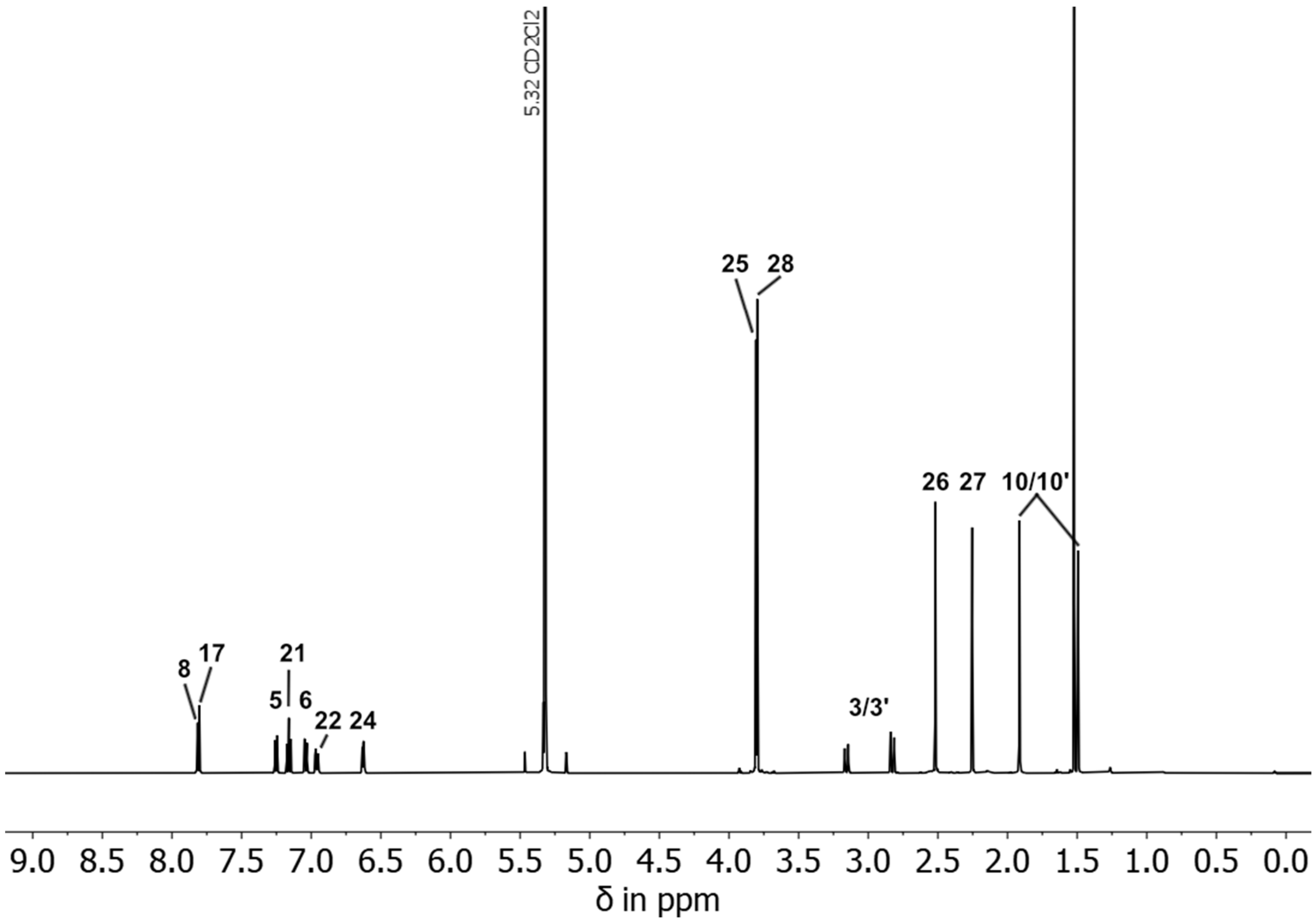

Supplementary Figure 37 ^{1}H NMR spectrum (CD_2Cl_2, 400 MHz, 25 °C) of racemic isomer 3-(*E*)-(*S*)-(*R*a). Arbitrary given numbers 1-28 were used for assignment of signals.

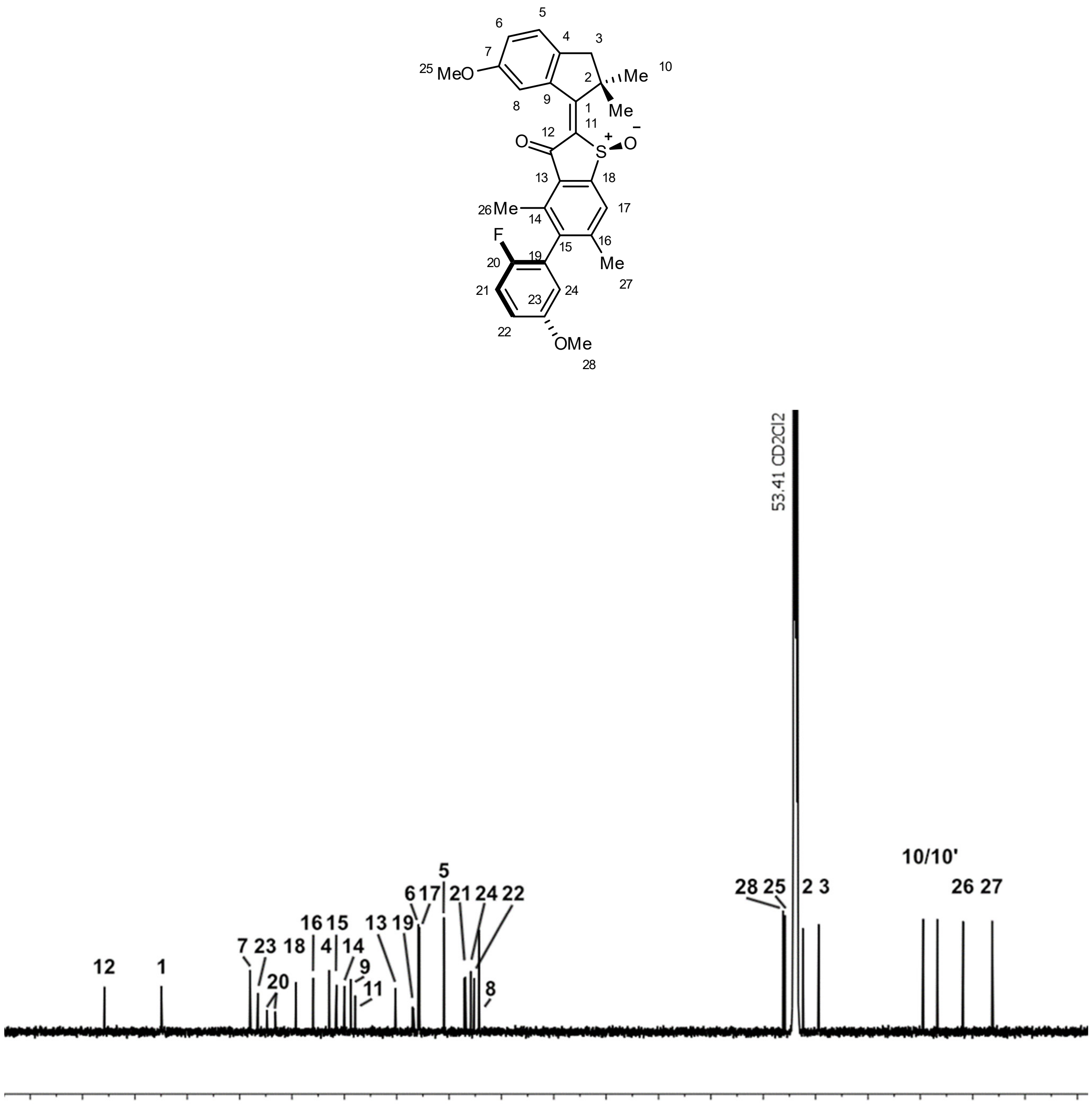

Supplementary Figure 38 **^{13}C NMR spectrum (CD_2Cl_2, 201 MHz, 25 °C) of racemic isomer 3-(*E*)-(*S*)-(*R*$_a$).** Arbitrary given numbers 1-28 were used for assignment of signals.

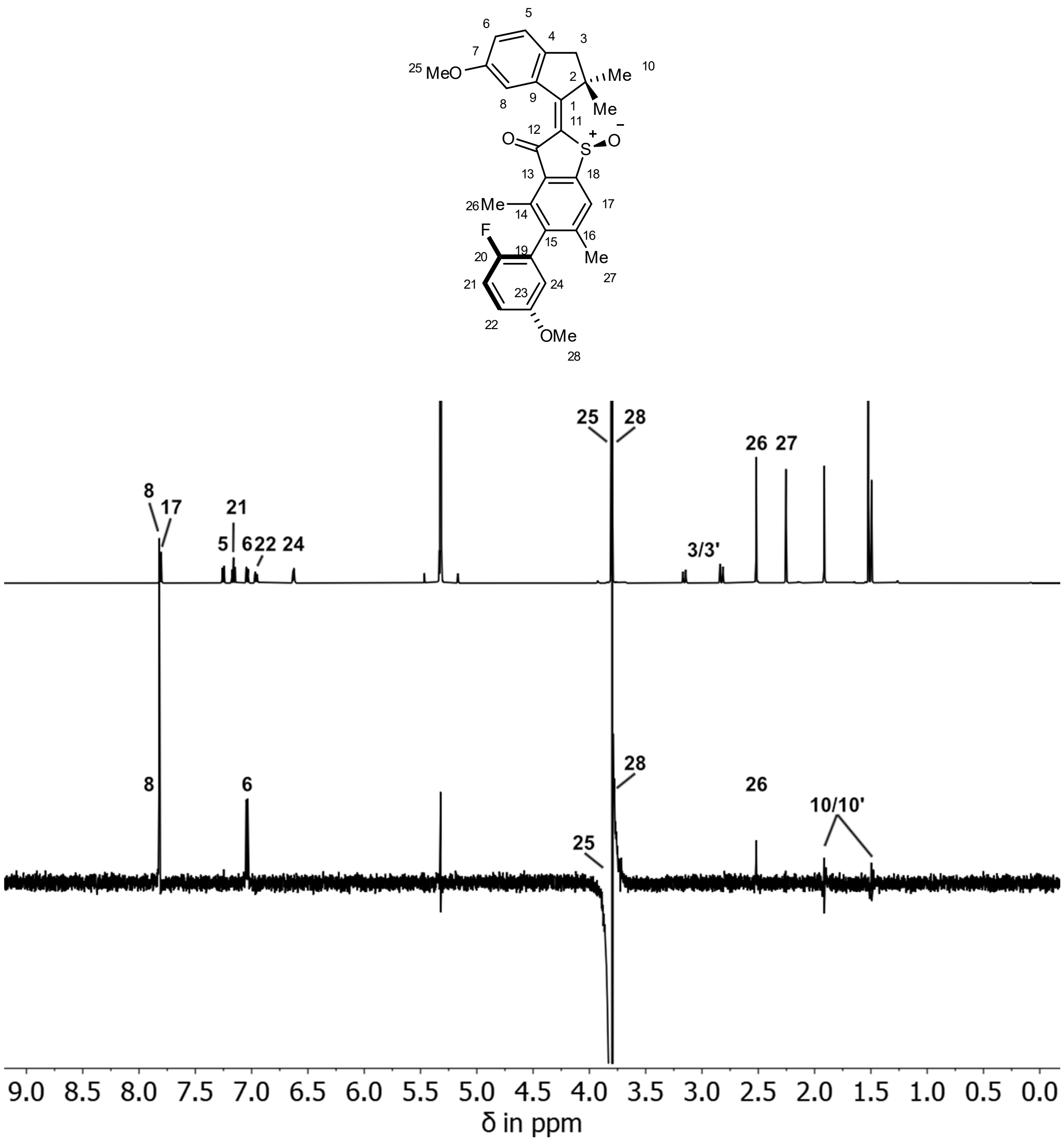

Supplementary Figure 39 NOE NMR spectrum (CD_2Cl_2, 600 MHz, 25 °C) of isomer 3-(*E*)-(*S*)-(*R*$_a$) evidencing *E*-configuration. Double bond configuration was assigned by coupling of methyl group H_3-C25 to aromatic methyl group H_3-C26 but not H_3-C27.

Isomer 3-(*Z*)-(*S*)-(*R*a)

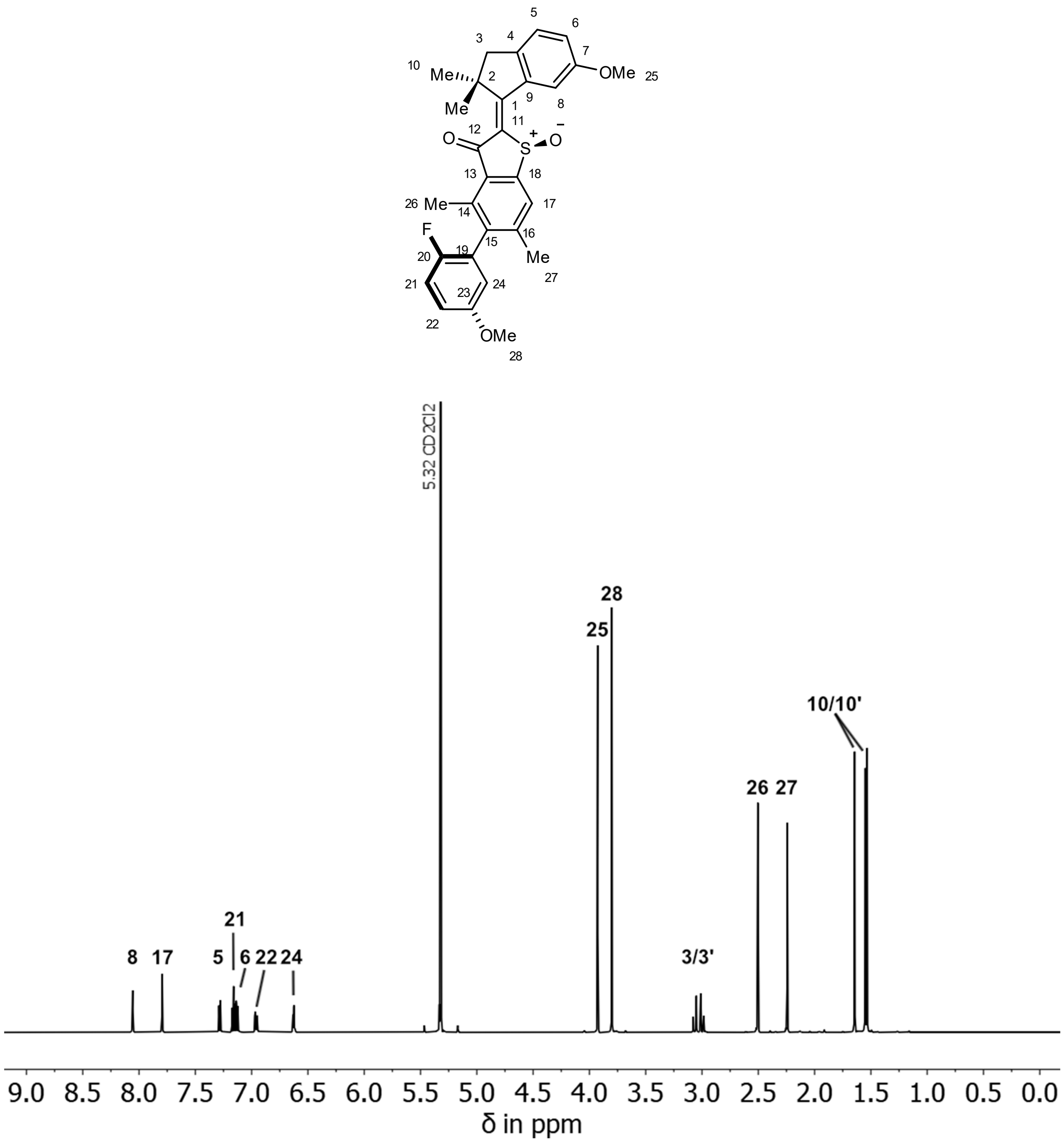

Supplementary Figure 40 ^{1}H NMR spectrum (CD_2Cl_2, 400 MHz, 25 °C) of racemic isomer 3-(*Z*)-(*S*)-(*R*a). Arbitrary given numbers 1-28 were used for assignment of signals.

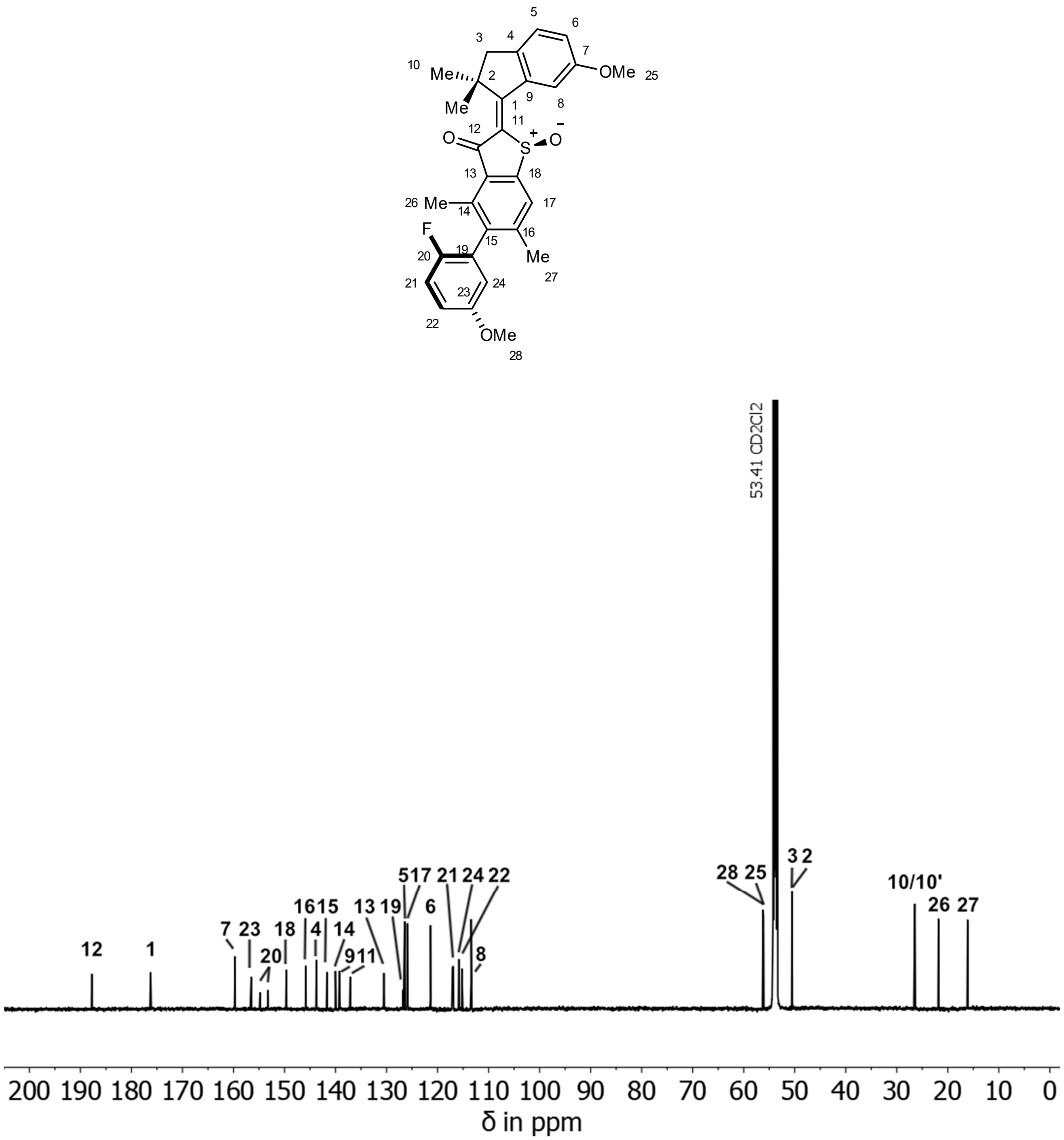

Supplementary Figure 41 **^{13}C NMR spectrum (CD_2Cl_2, 201 MHz, 25 °C) of racemic isomer 3-(*Z*)-(*S*)-(*R*$_a$).** Arbitrary given numbers 1-28 were used for assignment of signals.

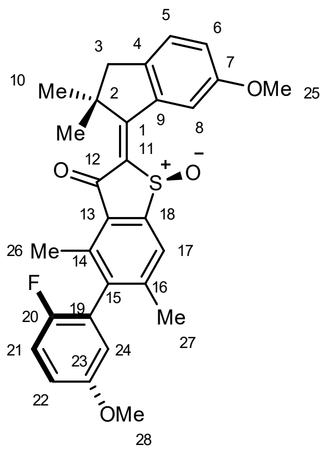

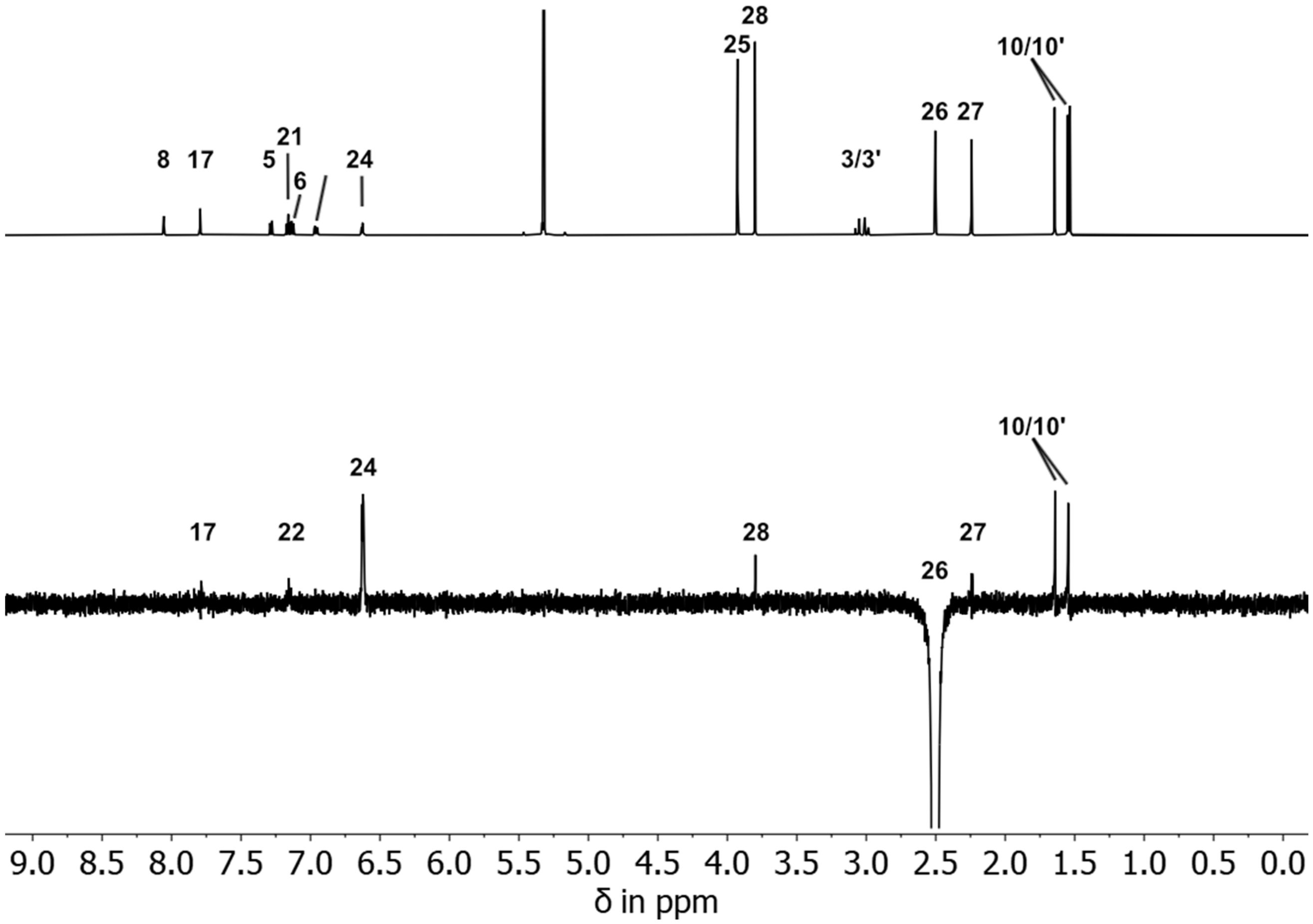

Supplementary Figure 42 NOE NMR spectrum (CD_2Cl_2, 600 MHz, 25 °C) of isomer 3-(*Z*)-(*S*)-(*R*$_a$) evidencing *Z*-configuration. Double bond configuration was assigned by coupling of aromatic methyl group H_3-C26 to methyl groups H_3-C10 and H_3-C10'.

Structures in the Crystalline State

Isomer 2-*Z*-I

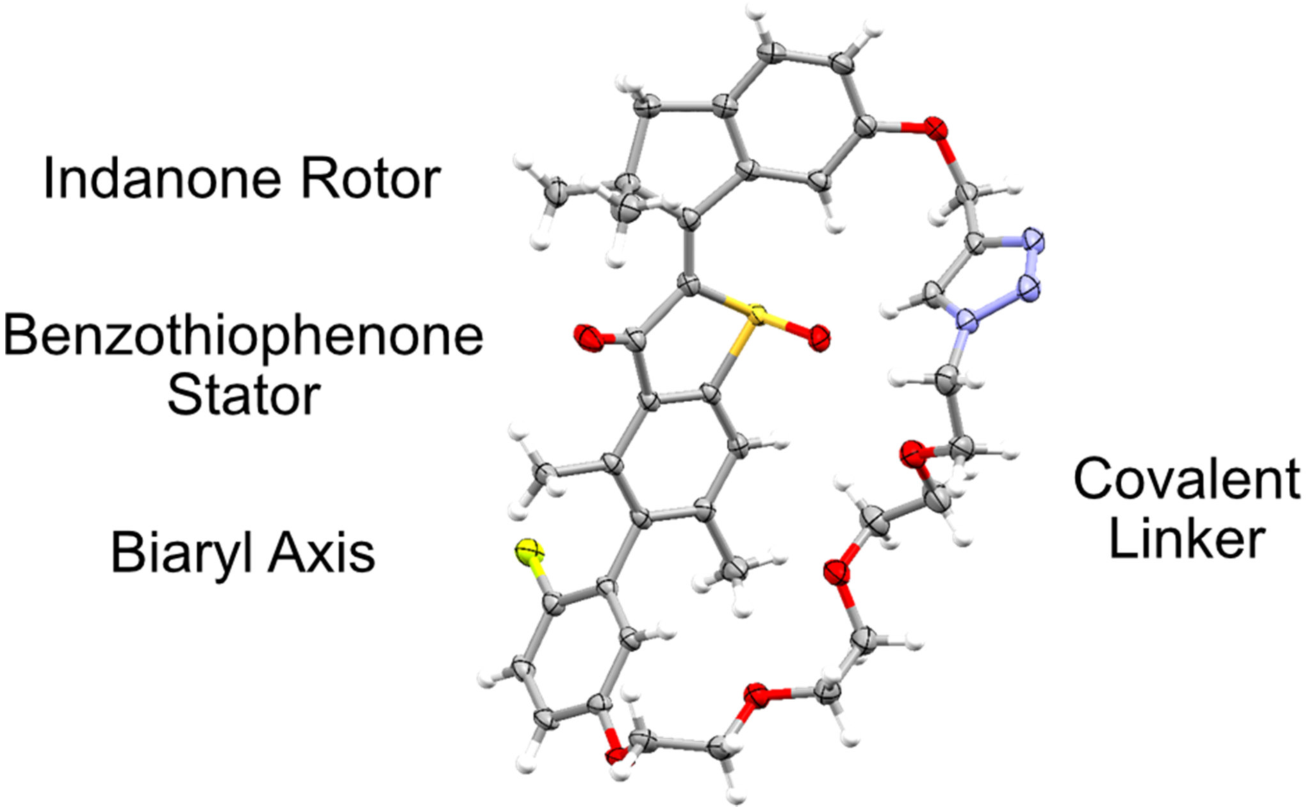

Supplementary Figure 43 Structure of racemic isomer 2-*Z*-I in the crystalline state. The crystal structure clearly allows the assignment of a *Z*-configured double bond of the motor unit, (*S*) configured sulfoxide, as well as (R_a)-configured biaryl axis, which is tilted towards the attachment point of the linker on the rotor moiety. Taken together all stereo-elements macrocyclic **2**-*Z*-**I** adopts (*Z*)-(*S*)-(*M*)-(S_a) configuration in the solid state (the enantiomeric (*Z*)-(*R*)-(*P*)-(R_a) configuration is not shown for clarity).

Isomer 2-*E*-II

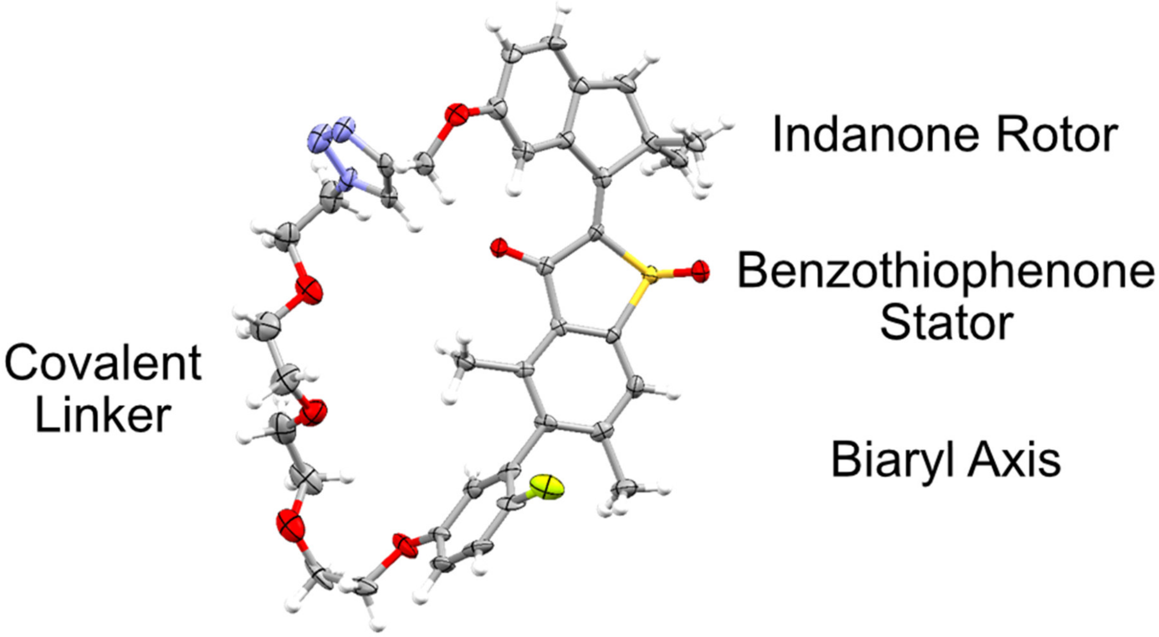

Supplementary Figure 44 Structure of racemic isomer 2-*E*-II in the crystalline state. The crystal structure clearly allowes the assignment of an *E*-configured double bond of the motor unit, (*S*) configured sulfoxide, as well as (R_a)-configured biaryl axis, which is tilted towards the attachment point of the linker on the rotor moiety. Taken together all stereo-elements macrocyclic **2**-*E*-**II** adopts (*E*)-(*S*)-(*P*)-(R_a) configuration in the solid state (the enantiomeric (*E*)-(*R*)-(*M*)-(S_a) configuration is not shown for clarity).

Isomer 2-*Z*-II

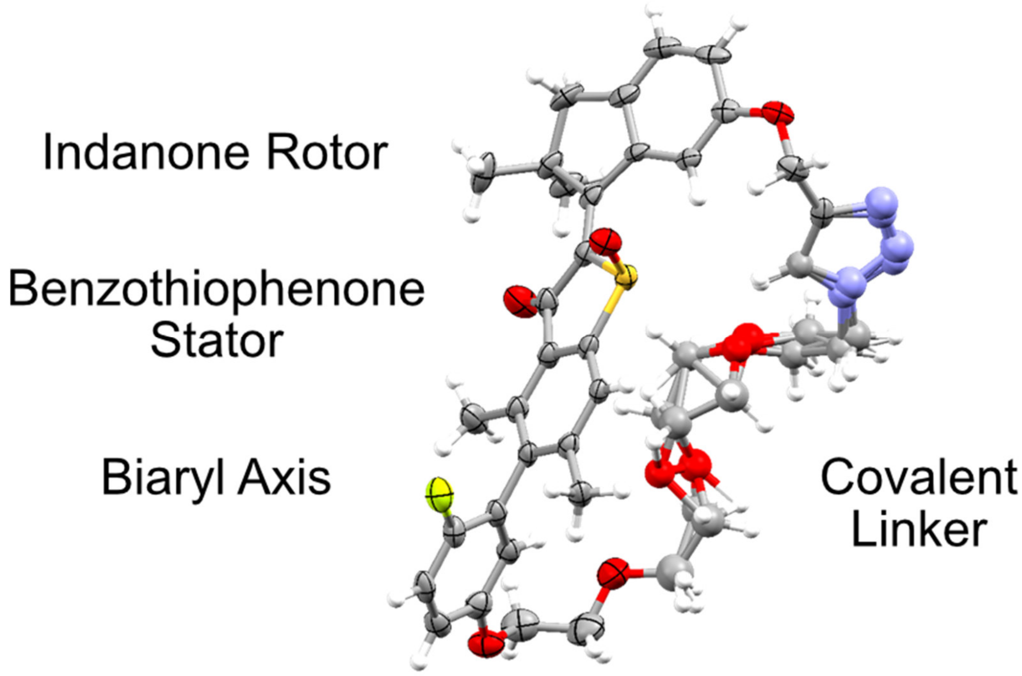

Supplementary Figure 45 Structure of racemic isomer 2-*Z*-II in the crystalline state. The crystal structure clearly allows the assignment of an *E*-configured double bond of the motor unit, (*S*) configured sulfoxide, as well as (R_a)-configured biaryl axis, which is tilted towards the attachment point of the linker on the rotor moiety. Taken together all stereo-elements macrocyclic **2**-*E*-**II** adopts (*Z*)-(*S*)-(*P*)-(R_a) configuration in the solid state (the enantiomeric (*Z*)-(*R*)-(*M*)-(S_a) configuration is not shown for clarity).

Isomer 3-*E*-(*R*)-(R_a)

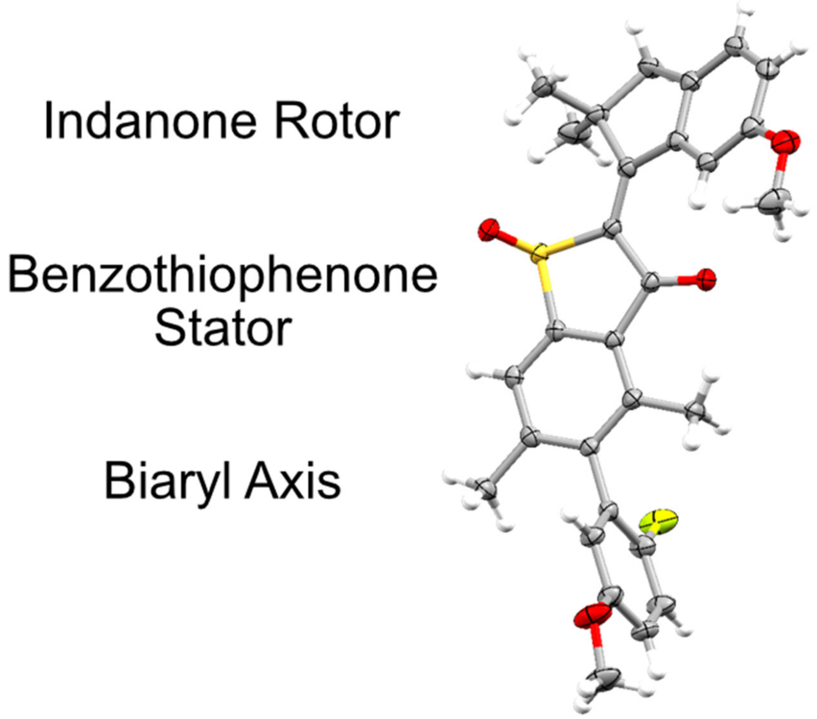

Supplementary Figure 46 Structure of enantiomerically pure isomer 3-*E*-(*R*)-(R_a) in the crystalline state. The crystal structure clearly allows the assignment of an *E*-configured double bond of the motor unit, (*R*) configured sulfoxide, as well as (R_a)-configured biaryl axis. Taken together all stereo-elements **3**-*E*-(*R*)-(R_a) adopts (*E*)-(*R*)-(*M*)-(R_a) configuration in the solid state.

Supplementary Note 4: Behavior at Elevated Temperatures

Thermal first-order isomerization processes were observed between **2**-*E*-**I** and **2**-*E*-**II**, **2**-*E*-**I** and **2**-*Z*-**I**, as well as **2**-*E*-**II** and **2**-*Z*-**II**, which proceed until a dynamic equilibrium is reached. This process is exemplarily described for the thermal isomerization between **2**-*E*-**I** and **2**-*Z*-**I** using Equation 1:

$$\ln\left[\frac{c(2\text{-}E\text{-I}_0) - c(2\text{-}E\text{-I}_{\text{eq}})}{c(2\text{-}E\text{-I}_t) - c(2\text{-}E\text{-I}_{\text{eq}})}\right] = [k(2\text{-}E\text{-I} \rightarrow 2\text{-}Z\text{-I}) + k(2\text{-}Z\text{-I} \rightarrow 2\text{-}E\text{-I})]\, t \qquad (1)$$

The observed decay is a combination of the rate constant for the forward process $k(2\text{-}E\text{-I} \rightarrow 2\text{-}Z\text{-I})$ and the rate constant for the backwards isomerization $k(2\text{-}Z\text{-I} \rightarrow 2\text{-}E\text{-I})$. This relationship can be fitted with a linear regression analysis using values for the initial concentration of 2-*E*-I $c(2\text{-}E\text{-I}_0)$, the concentration of 2-*E*-I at equilibrium $c(2\text{-}E\text{-I}_{eq})$ and the concentration of 2-*E*-I at the elapsed time *t* $c(2\text{-}E\text{-I}_t)$. The slope from the linear fit of the logarithmic plot *m* can then be used to determine the rate constant $k(2\text{-}E\text{-I} \rightarrow 2\text{-}Z\text{-I})$ using Equation 2 after rearrangement and taking the law of mass action shown in Equation 3 into account:

$$\frac{c(2\text{-}E\text{-I}_{\text{eq}})}{c(2\text{-}Z\text{-I}_{\text{eq}})} = \frac{k(2\text{-}Z\text{-I} \rightarrow 2\text{-}E\text{-I})}{k(2\text{-}E\text{-I} \rightarrow 2\text{-}Z\text{-I})} \qquad (2)$$

$$k(2\text{-}E\text{-I} \rightarrow 2\text{-}Z\text{-I}) = \frac{m}{1 + \frac{c(2\text{-}E\text{-I}_{\text{eq}})}{c(2\text{-}Z\text{-I}_{\text{eq}})}} \qquad (3)$$

With the rate constant $k(2\text{-}E\text{-I} \rightarrow 2\text{-}Z\text{-I})$ at hand, the Gibbs free energy of activation $\Delta G^{\ddagger}$ can be calculated for the thermal 2-*E*-I to 2-*Z*-I isomerization, using the Eyring Equation 4:

$$k(2\text{-}E\text{-I} \rightarrow 2\text{-}Z\text{-I}) = \frac{k_{\text{B}}T}{h} e^{\frac{-\Delta G^{\ddagger}}{RT}} \qquad (4)$$

Inserting numerical values for the ideal gas constant R (8.31446 J·K^{-1}·mol^{-1}), the Boltzmann constant k_B (1.38065 J·K^{-1}), temperature T (K) and Planck constant h (6.62607·10^{-34} J·s), Equation 4 can be rearranged to Equation 5:

$$\Delta^{\ddagger}G = 8.314 \cdot \mathrm{T} \cdot \left[23.760 + \ln\left(\frac{T}{k(2\text{-}E\text{-I} \rightarrow 2\text{-}Z\text{-I})}\right)\right] \qquad (5)$$

From the stationary equilibrium composition, the relative energy difference between the two isomers 2-*E*-I and 2-*Z*-I can then be determined through inserting the measured numerical values for the equilibrium constant K into Equation 6:

$$\Delta G = \ln(K) \cdot 8.314 \cdot T = \ln\left(\frac{c(2\text{-}E\text{-I}_{eq})}{c(2\text{-}Z\text{-I}_{eq})}\right) \cdot 8.314 \cdot T \qquad (6)$$

Isomer 2-*E*-I and 2-*E*-II

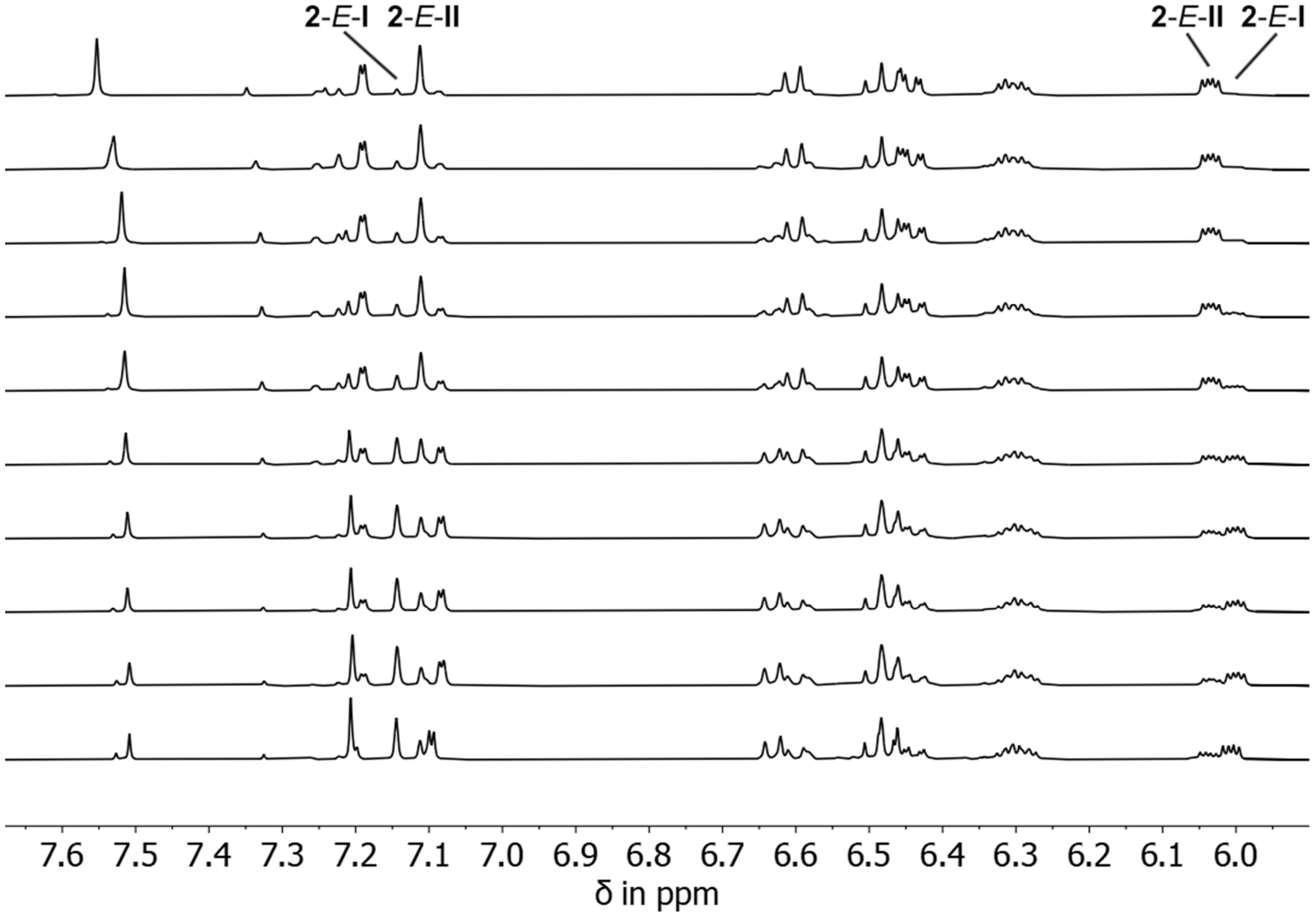

Supplementary Figure 47 ^{1}H NMR spectra recorded at 25 °C during thermal isomerization of isomer 2-*E*-II to 2-*E*-I at 80 °C in $(CDCl_2)_2$ solution. Starting from an isomer enriched solution containing 83% **2**-*E*-**II** and 17% **2**-*E*-**I** isomer a final solution containing 32% **2**-*E*-**II** and 68% **2**-*E*-**I** is obtained after heating for 88 hours. This translates into a Gibbs free energy difference of Δ*G* = 0.5 kcal mol^{-1} between **2**-*E*-**I** and **2**-*E*-**II**.

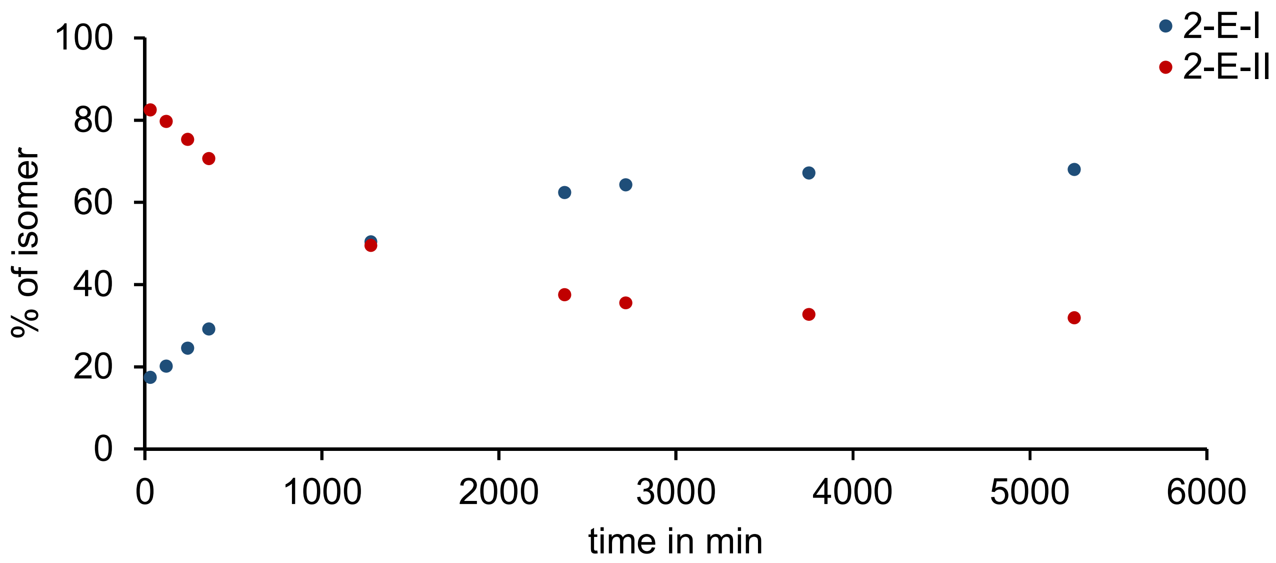

Supplementary Figure 48 Isomeric ratios during thermal isomerization. Changing isomeric ratios during thermal isomerization from **2**-*E*-**II** (red dots) to **2**-*E*-**I** (blue dots) at 80 °C in $(CDCl_2)_2$ solution.

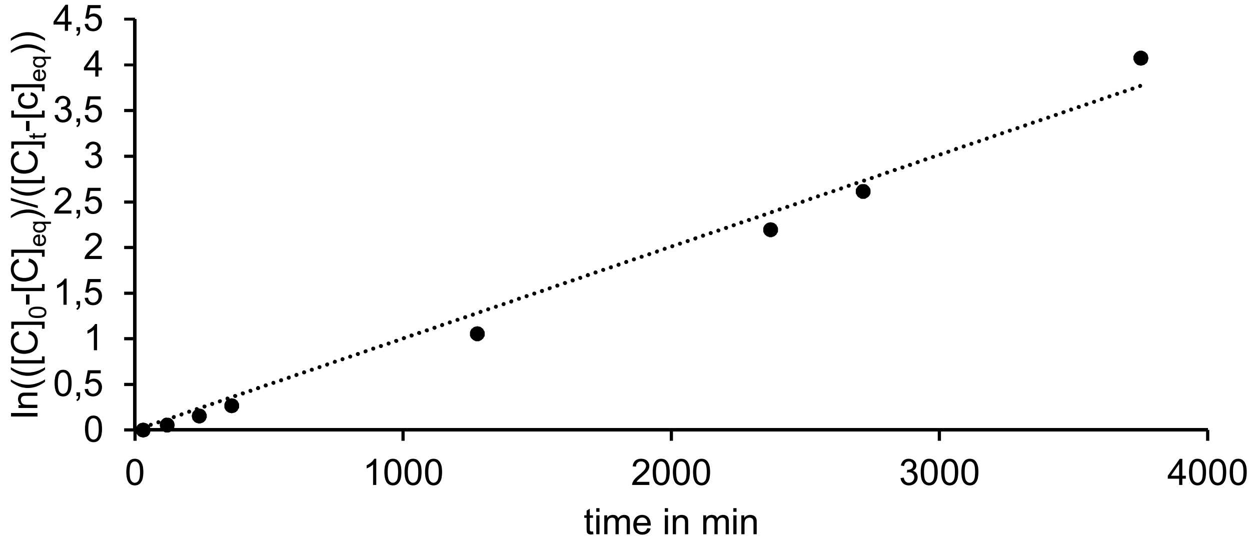

Supplementary Figure 49 Kinetic analysis of the thermal isomerization from 2-*E*-II to 2-*E*-I at 80 °C in $(CDCl_2)_2$ solution. Using a first order rate law and considering a dynamic equilibrium between isomer **2**-*E*-**II** and **2**-*E*-**I** provides a linear relationship. Fitting the data points with a linear regression analysis leads to a slope $m = 0.001007$, which translates into a Gibbs free energy of activation $\Delta G^{\ddagger} = 25.6$ kcal/mol.

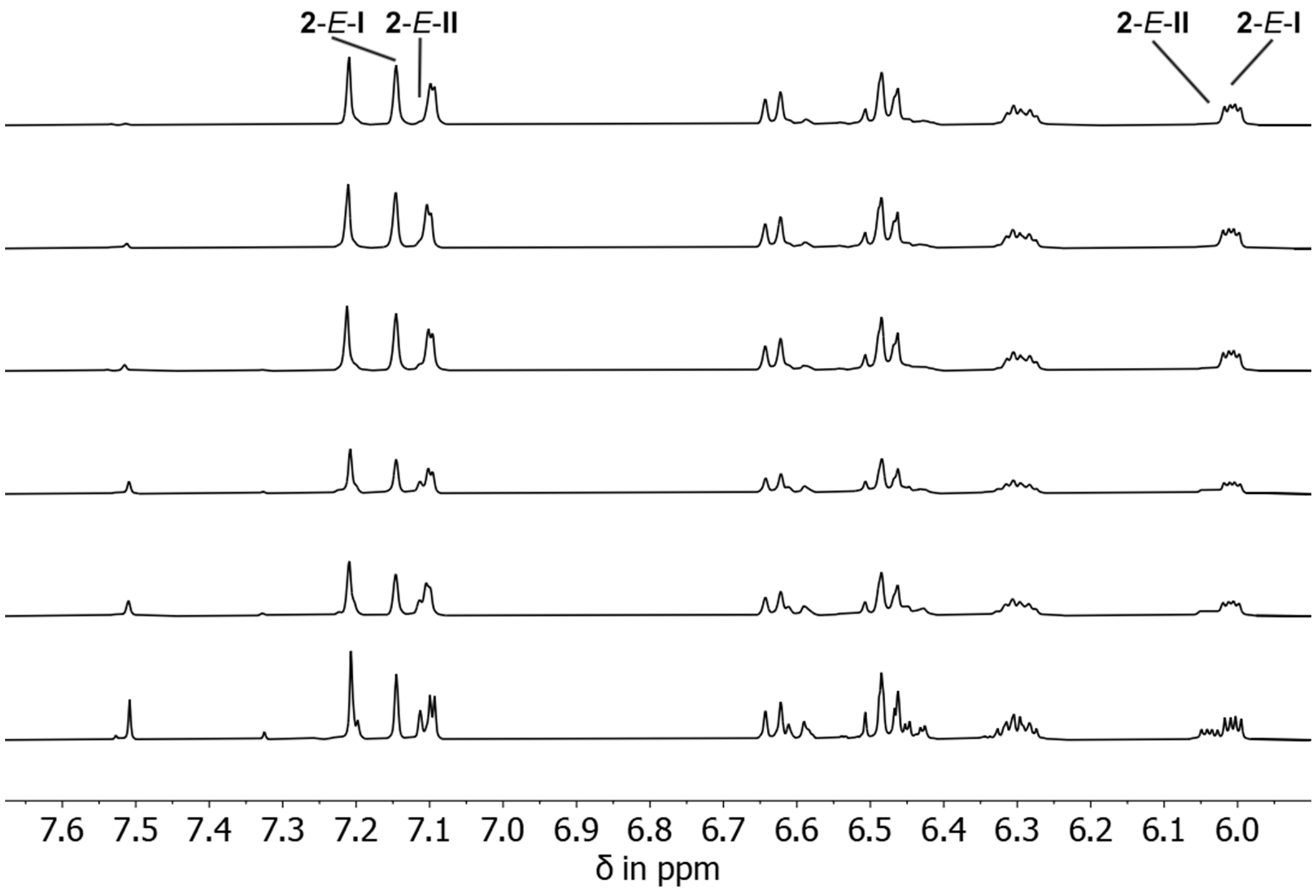

Supplementary Figure 50 ^{1}H NMR spectra recorded at 25 °C during thermal isomerization of isomer 2-*E*-I to 2-*E*-II at 80 °C in $(CDCl_2)_2$ solution. Starting from an isomer enriched solution containing 98% **2**-*E*-**I** and 2% **2**-*E*-**II** isomer a final solution containing 71% **2**-*E*-**I** and 29% **2**-*E*-**II** is obtained after heating for 82 hours. This translates into a Gibbs free energy difference of ΔG = 0.6 kcal mol^{-1} between **2**-*E*-**I** and **2**-*E*-**II**.

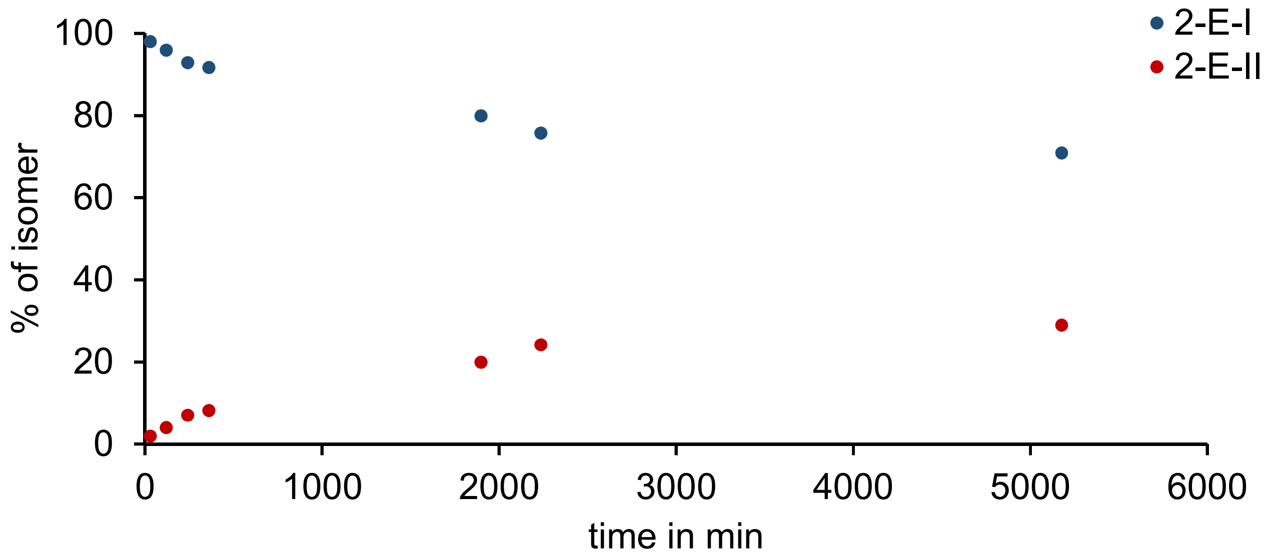

Supplementary Figure 51 Isomeric ratios during thermal isomerization. Changing isomeric ratios during thermal isomerization from **2**-*E*-**I** (blue dots) to **2**-*E*-**II** (red dots) at 80 °C in $(CDCl_2)_2$ solution.

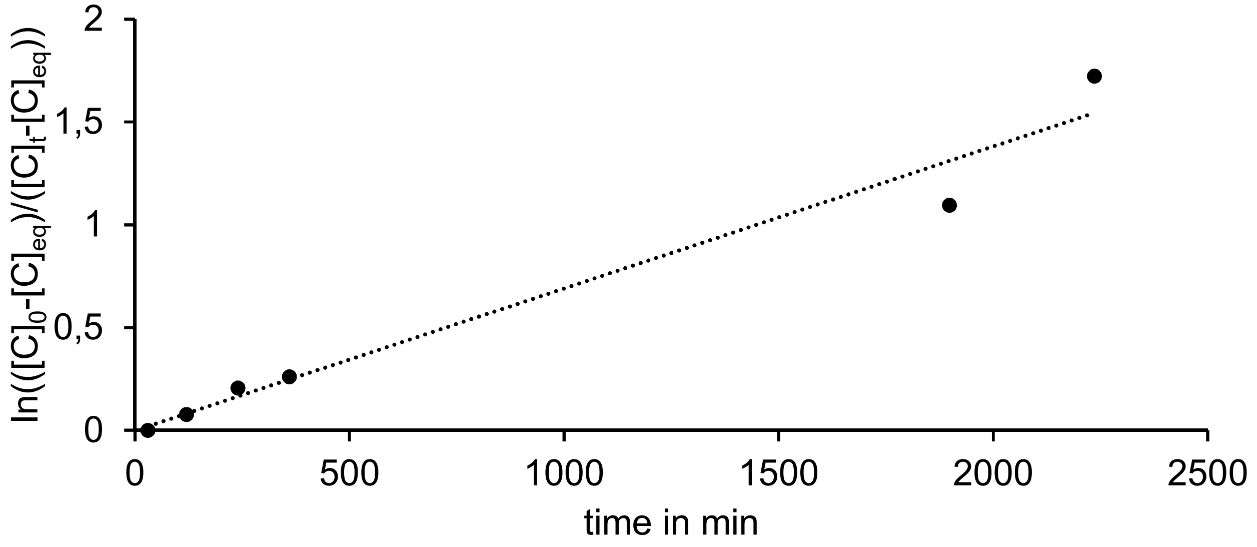

Supplementary Figure 52 Kinetic analysis of the thermal isomerization from 2-*E*-I to 2-*E*-II at 80 °C in $(CDCl_2)_2$ solution. Using a first order rate law and considering a dynamic equilibrium between isomer **2**-*E*-**I** and **2**-*E*-**II** provides a linear relationship. Fitting the data points with a linear regression analysis leads to a slope $m = 0.\ 000691$, which translates into a Gibbs free energy of activation $\Delta G^{\ddagger} = 25.9$ kcal mol^{-1}.

Isomer 2-*E*-I and 2-*Z*-I

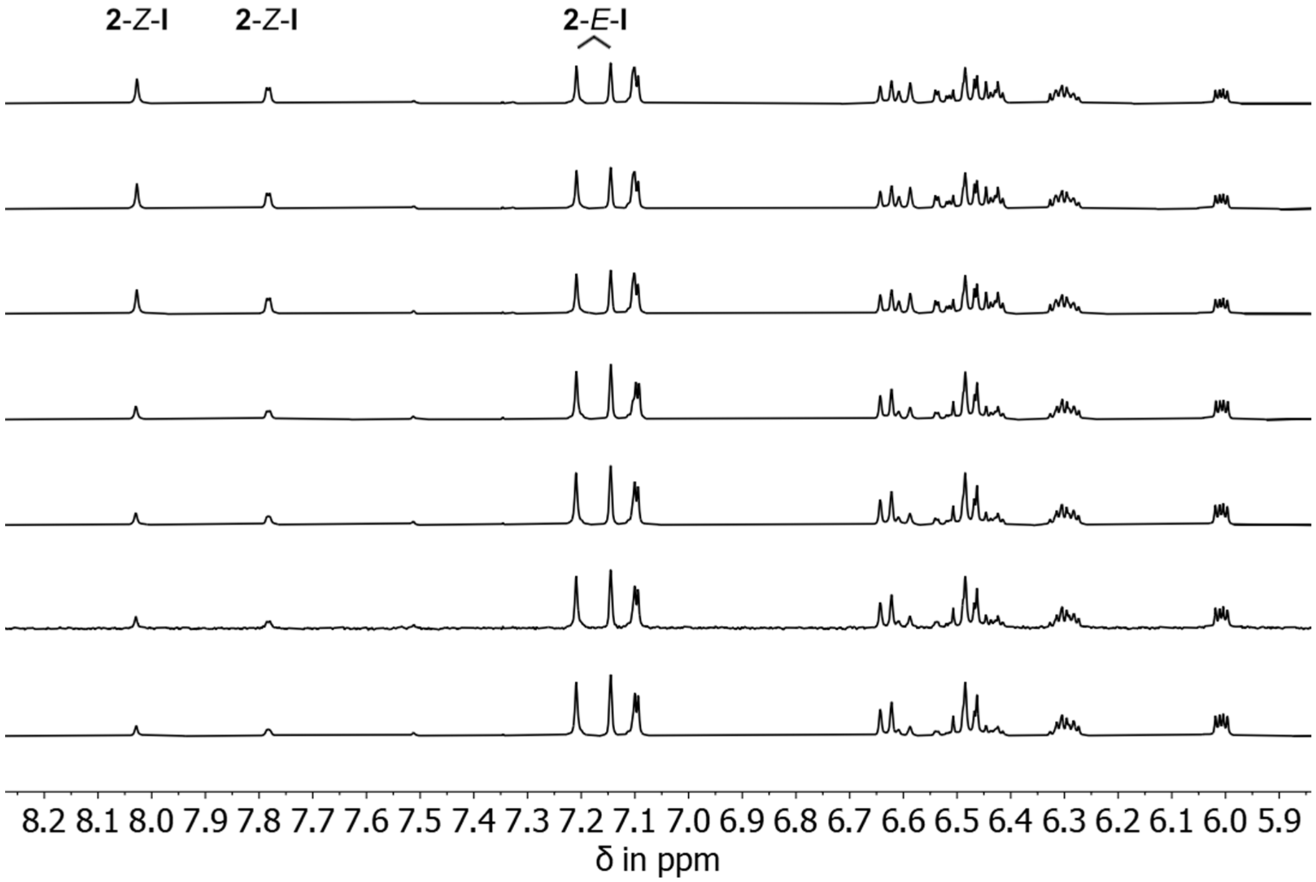

Supplementary Figure 53 **^{1}H NMR spectra recorded at 25 °C during thermal isomerization of isomer 2-*Z*-I to 2-*E*-I at 40 °C in $(CDCl_2)_2$ solution.** Starting from an isomer enriched solution containing 58% **2**-*E*-**I** and 42% **2**-*Z*-**I** isomer a final solution containing 84% **2**-*E*-**I** and 16% **2**-*Z*-**I** is obtained after heating for 71 hours. This translates into a Gibbs free energy difference of ΔG = 1.0 kcal/mol between **2**-*E*-**I** and **2**-*Z*-**I**.

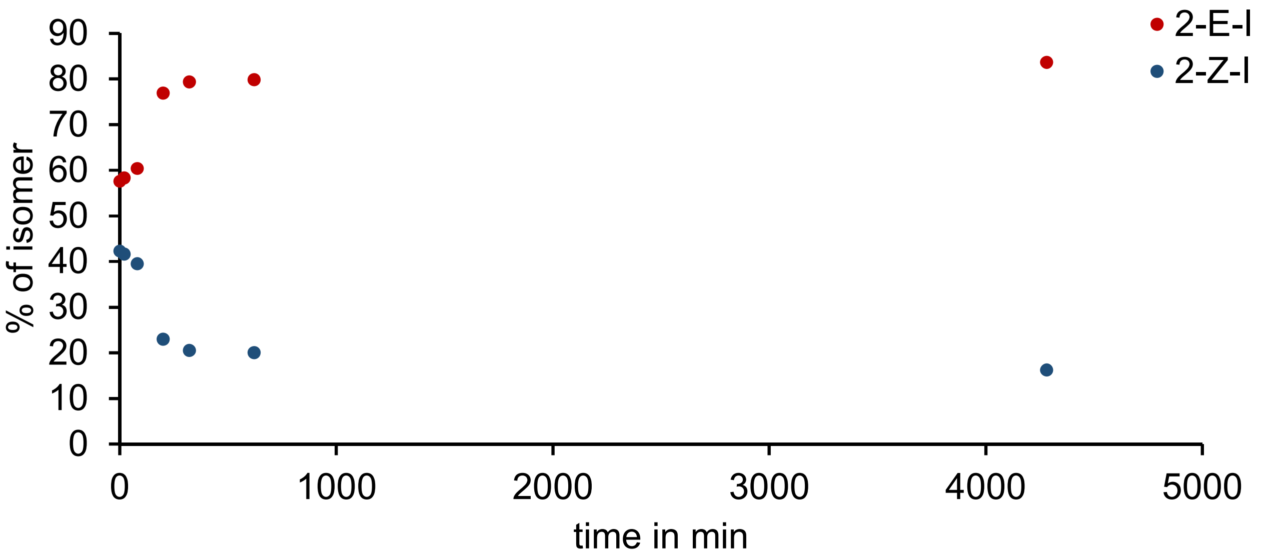

Supplementary Figure 54 Isomeric ratios during thermal isomerization. Changing isomeric ratios during thermal isomerization from **2**-*Z*-**I** (blue dots) to **2**-*E*-**I** (red dots) at 40 °C in $(CDCl_2)_2$ solution.

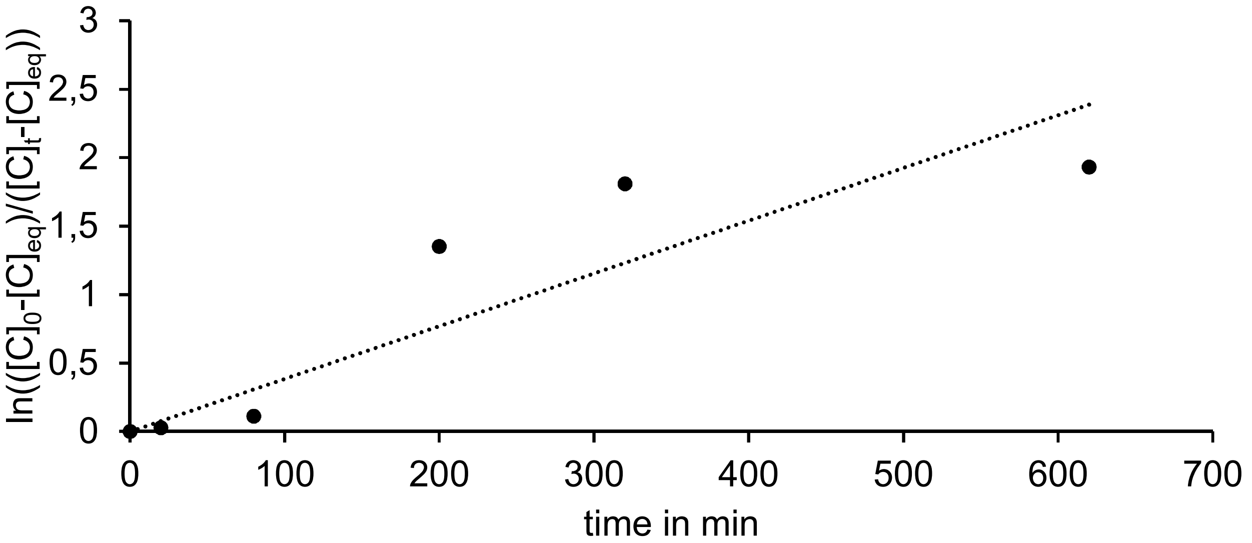

Supplementary Figure 55 Kinetic analysis of the thermal isomerization from 2-*E*-I to 2-*Z*-I at 40 °C in $(CDCl_2)_2$ solution. Using a first order rate law and considering a dynamic equilibrium between isomer **2**-*E*-**I** and **2**-*Z*-**I** provides a linear relationship. Fitting the data points with a linear regression analysis leads to a slope m = 0. 003854, which translates into a Gibbs free energy of activation $\Delta G^{\ddagger}$ = 21.8 kcal mol^{-1}.

Isomer 2-*E*-II and 2-*Z*-II

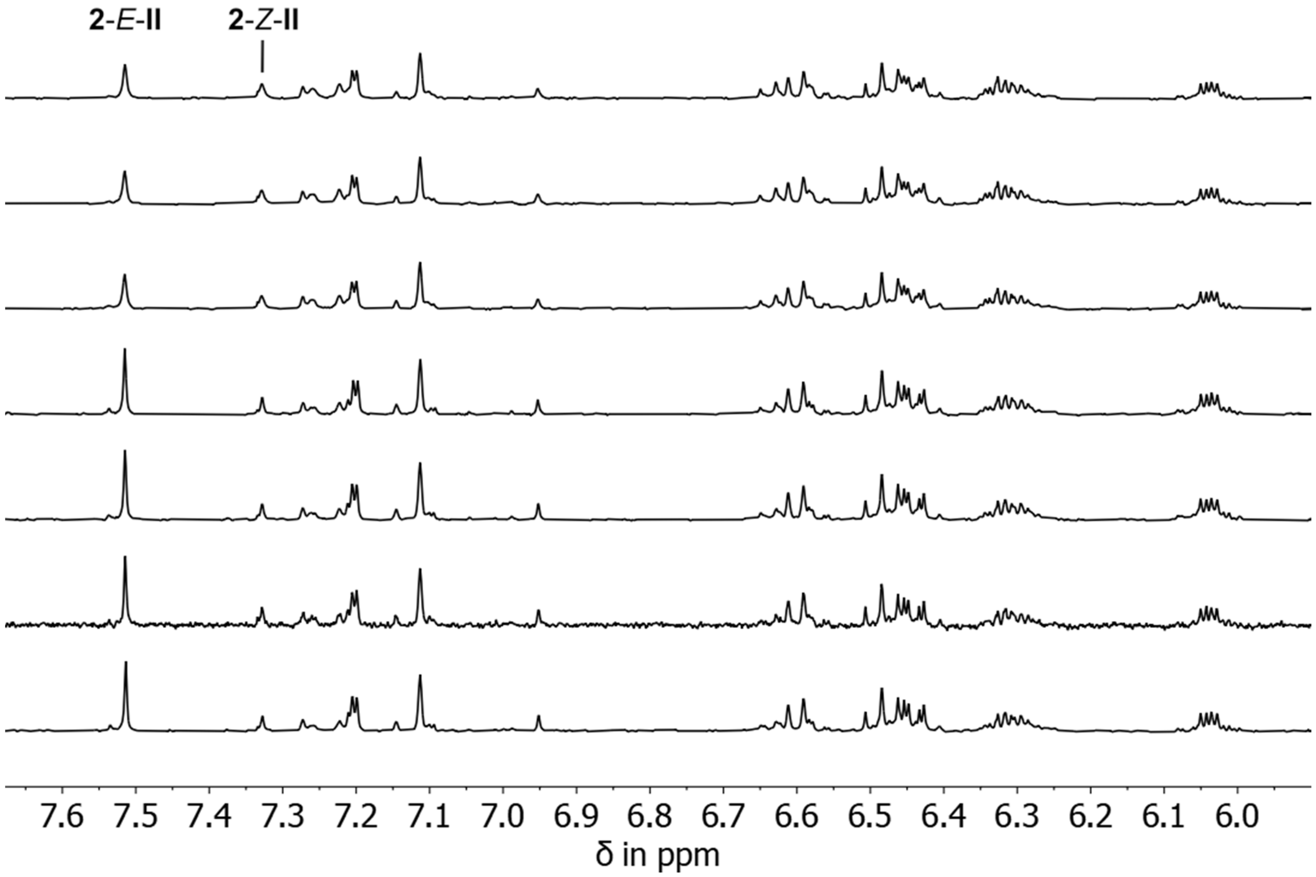

Supplementary Figure 56 **^{1}H NMR spectra recorded at 25 °C during thermal isomerization of isomer 2-*Z*-II to 2-*E*-II at 40 °C in $(CDCl_2)_2$ solution.** Starting from an isomer enriched solution containing 61% **2**-*E*-**II** and 39% **2**-*Z*-**II** isomer a final solution containing 80% **2**-*E*-**II** and 20% **2**-*Z*-**II** is obtained after heating for 71 hours. This translates into a Gibbs free energy difference of ΔG = 0.9 kcal kcal mol^{-1} between **2**-*E*-**II** and **2**-*Z*-**II**.

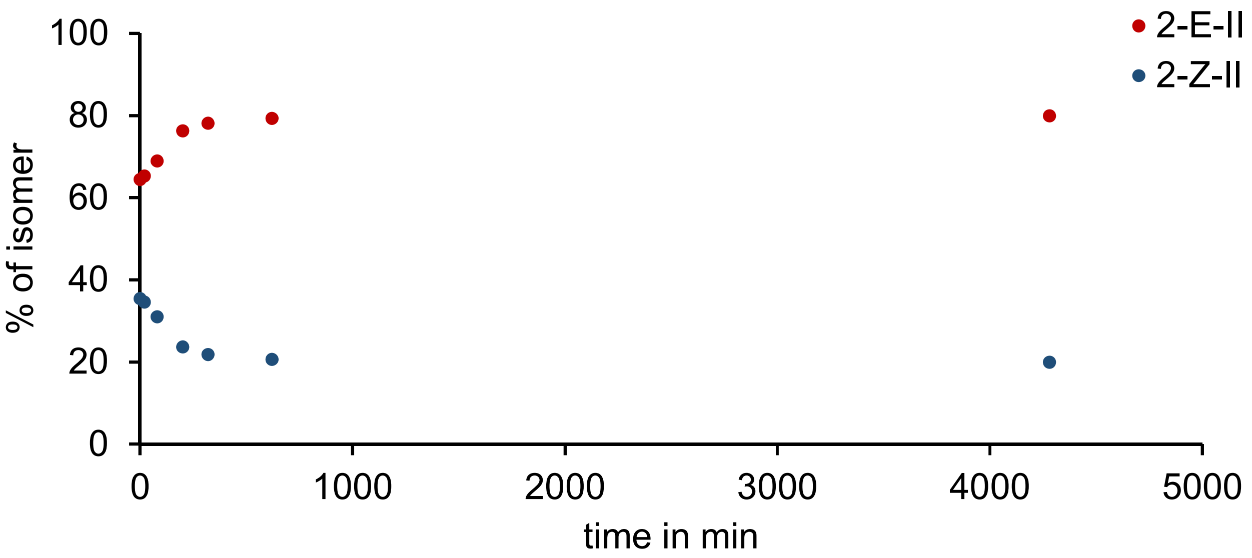

Supplementary Figure 57 Isomeric ratios during thermal isomerization. Changing isomeric ratios during thermal isomerization from **2**-*Z*-**II** (blue dots) to **2**-*E*-**II** (red dots) at 40 °C in $(CDCl_2)_2$ solution.

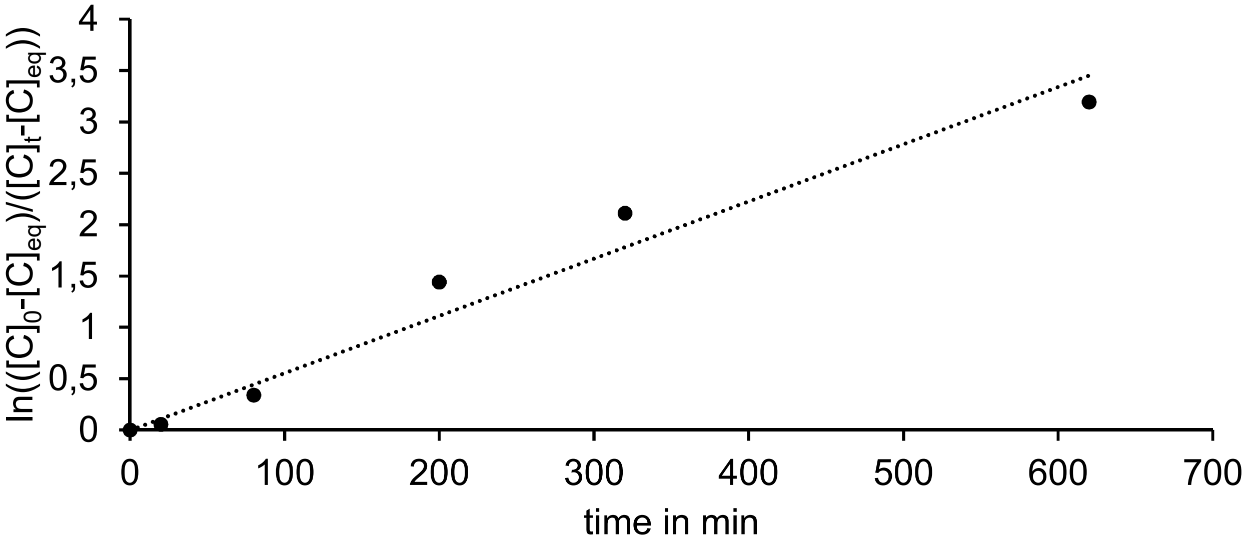

Supplementary Figure 58 Kinetic analysis of the thermal isomerization from 2-*E*-II to 2-*Z*-II at 40 °C in $(CDCl_2)_2$ solution. Using a first order rate law and considering a dynamic equilibrium between isomer **2**-*E*-**II** and **2**-*Z*-**II** provides a linear relationship. Fitting the data points with a linear regression analysis leads to a slope m = 0. 005570, which translates into a Gibbs free energy of activation $\Delta G^{‡}$ = 21.6 kcal mol^{-1}.

Supplementary Table 1 Overview of measured Gibbs energies of activation for macrocyclic HTI 2. Error margins were obtained from the 95% confidence interval of the slope of the linear regression analysis.

Isomer	$\Delta G^{\ddagger}$ (kcal mol^{-1})
**2**-*Z*-**I**	21.8 ± 0.3
**2**-*Z*-**II**	21.6 ± 0.1
**2**-*E*-**I**	25.6 ± 0.1
**2**-*E*-**II**	25.9 ± 0.1

Supplementary Table 2 Overview of measured ΔG values for the different isomers of macrocyclic HTI 2. The error margin was obtained from the lower and upper bound of the conservatively assumed 5% accuracy of ^{1}H NMR spectra integration.

Isomer	ΔG (kcal mol^{-1})
**2**-*E*-**I**	0
**2**-*E*-**II**	0.6 ± 0.2
**2**-*Z*-**I**	1.0 ± 0.3
**2**-*Z*-**II**	1.4 ± 0.3

Supplementary Note 5: UV-Vis Absorption Spectra

Isomers 2-*E*-I and 2-*Z*-I

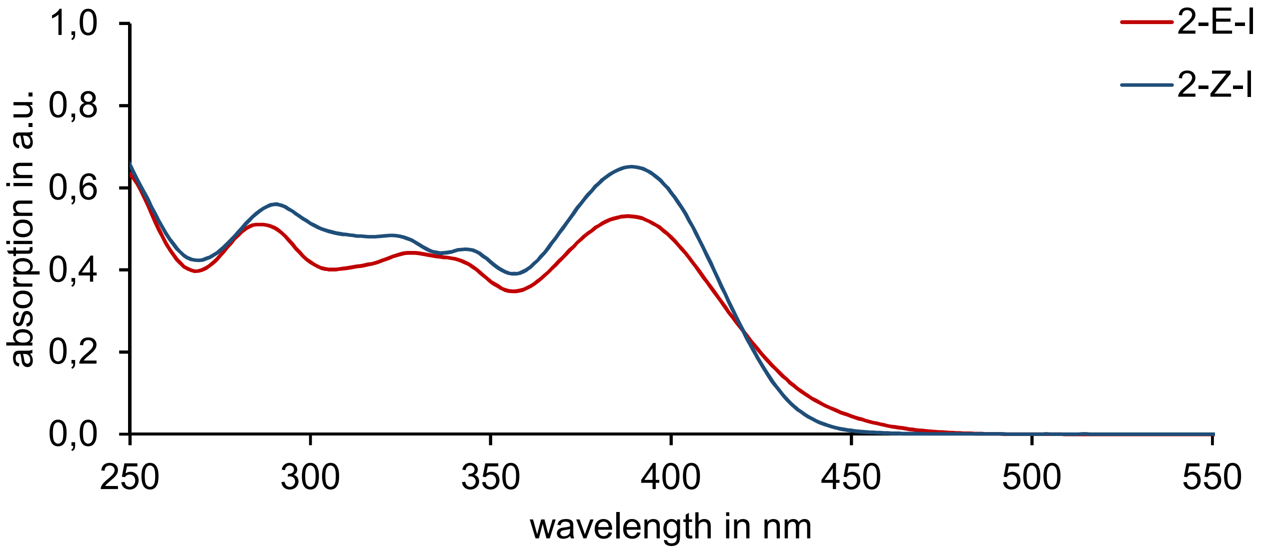

Supplementary Figure 59 Recorded UV-Vis absorption spectra spectra. UV-Vis spectra of isomer **2**-*E*-**I** (red line) scaled to fit the isosbestic point at 420 nm and **2**-*Z*-**I** (blue line) measured at 22 °C at a concentration of 42 µM in CH_2Cl_2.

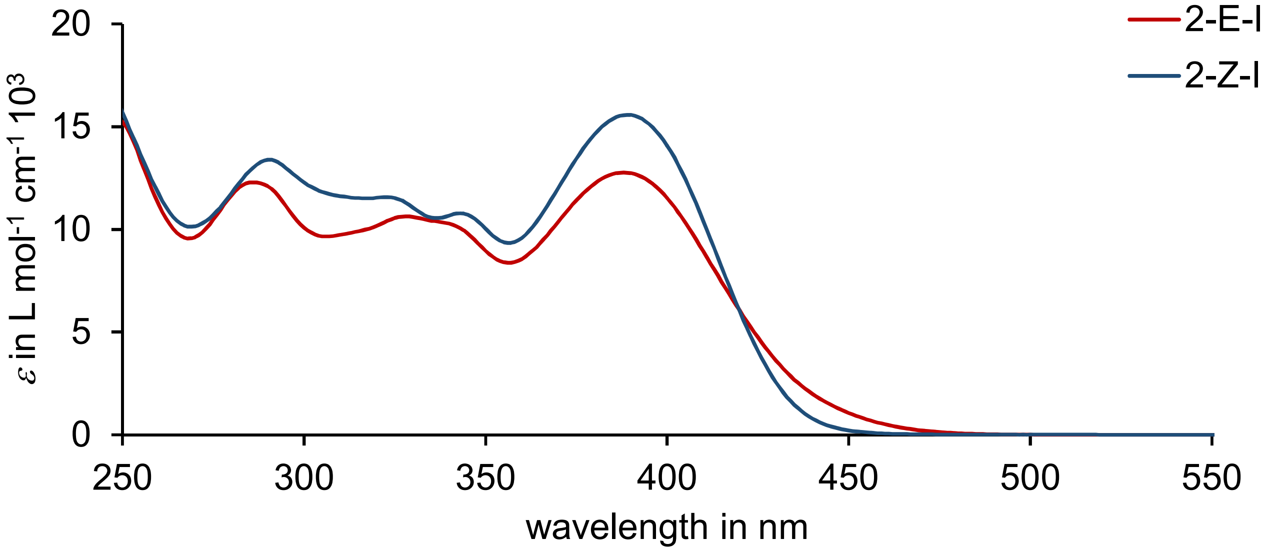

Supplementary Figure 60 Molar extinction coefficients. Obtained molar extinction coefficients *ε* of isomers **2**-*E*-**I** (red line) and **2**-*Z*-**I** (blue line) measured at 22 °C in CH_2Cl_2.

Isomers 2-*E*-II and 2-*Z*-II

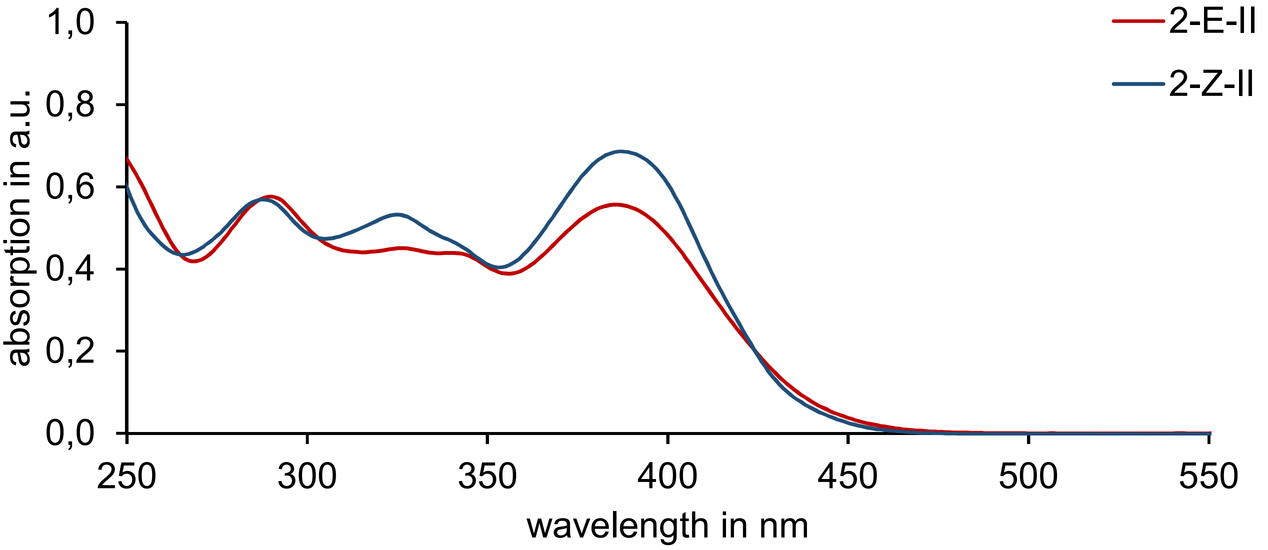

Supplementary Figure 61 Recorded UV-Vis absorption spectra spectra. UV-Vis spectra of isomer **2-*E*-II** (red line) scaled to fit the isosbestic point at 424 nm and **2-*Z*-II** (blue line) measured at 22 °C at a concentration of 47 μM in CH_2Cl_2.

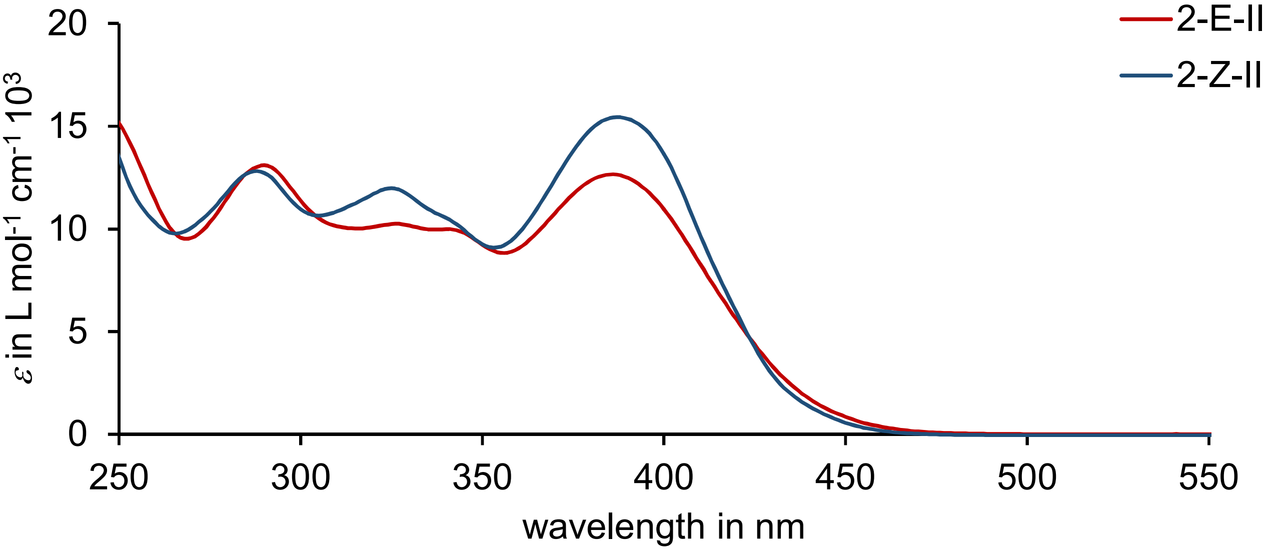

Supplementary Figure 62 Molar extinction coefficients. Obtained molar extinction coefficients ε of isomers **2-*E*-II** (red line) and **2-*Z*-II** (blue line) measured at 22 C in CH_2Cl_2.

Isomers 3-*E*-(*S*)-(S_a) and 3-*Z*-(*S*)-(S_a)

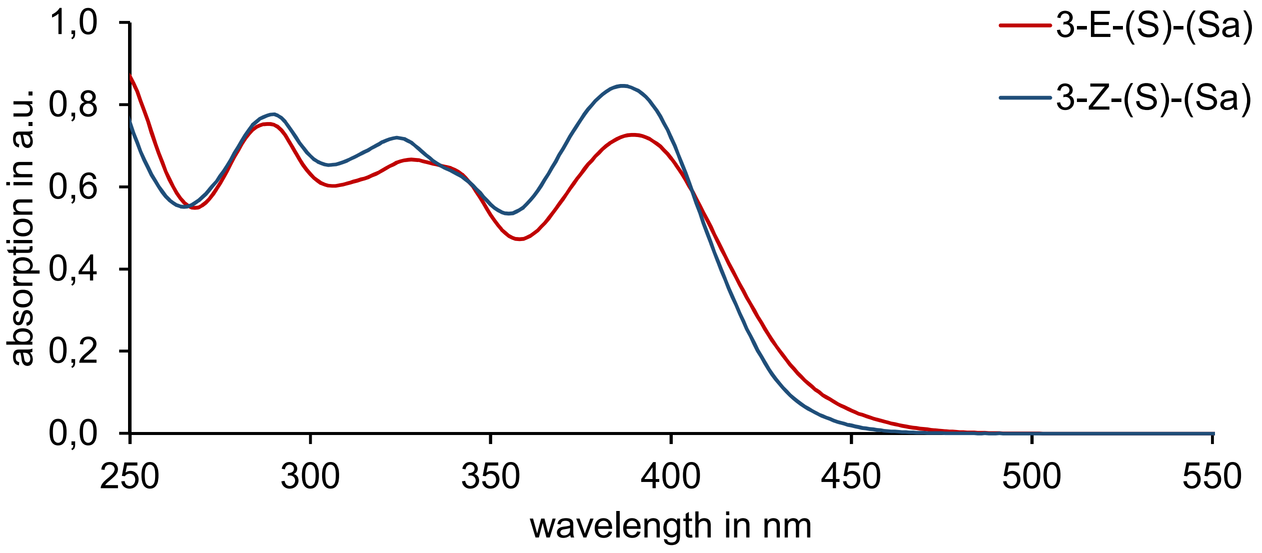

Supplementary Figure 63 Recorded UV-Vis absorption spectra spectra. UV-Vis spectra of isomer **3**-*E*-(*S*)-(S_a) (red line) scaled to fit the isosbestic point at 407 nm and **3**-*Z*-(*S*)-(S_a) (blue line) measured at 22 °C at a concentration of 42 μM in CH_2Cl_2.

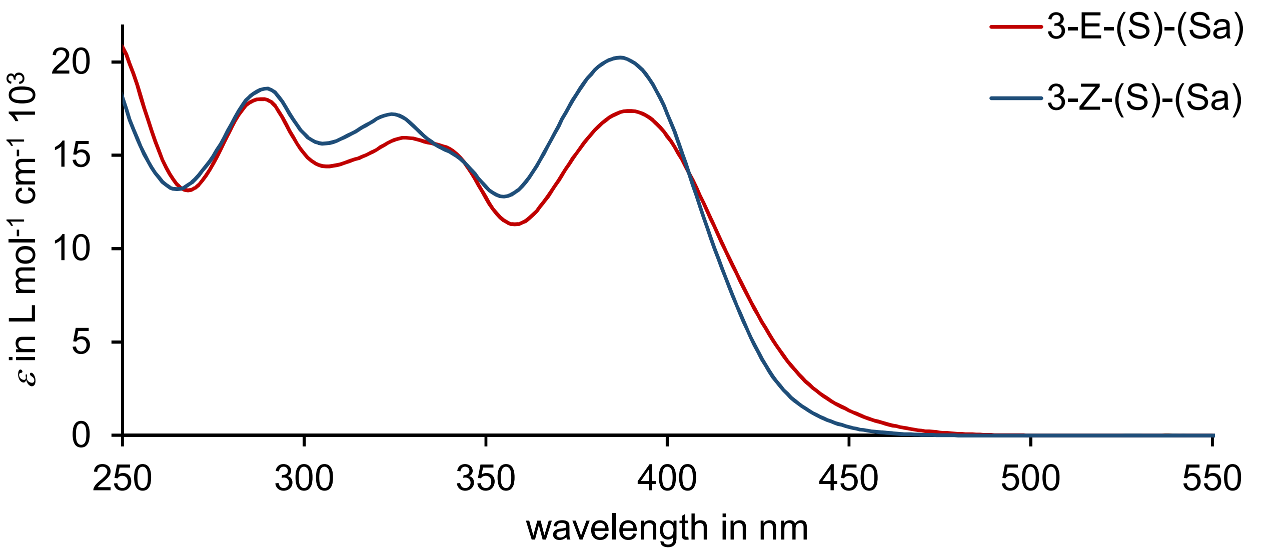

Supplementary Figure 64 Molar extinction coefficients. Obtained molar extinction coefficients *ε* of isomers **3**-*E*-(*S*)-(S_a) (red line) and **3**-*Z*-(*S*)-(S_a) (blue line) measured at 22 °C in CH_2Cl_2.

Isomers 3-*E*-(*S*)-(*R*$_a$) and 3-*Z*-(*S*)-(*R*$_a$)

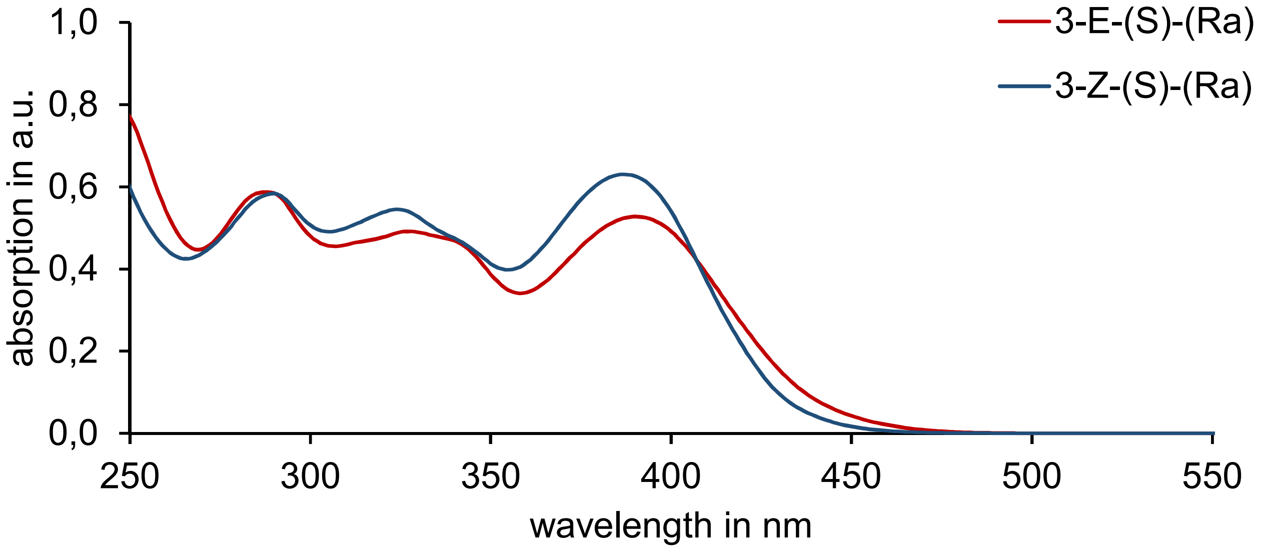

Supplementary Figure 65 Recorded UV-Vis absorption spectra spectra. UV-Vis spectra of isomer **3**-*E*-(*S*)-(*R*$_a$) (red line) scaled to fit the isosbestic point at 407 nm and **3**-*Z*-(*S*)-(*R*$_a$) (blue line) measured at 22 °C at a concentration of 40 µM in CH_2Cl_2.

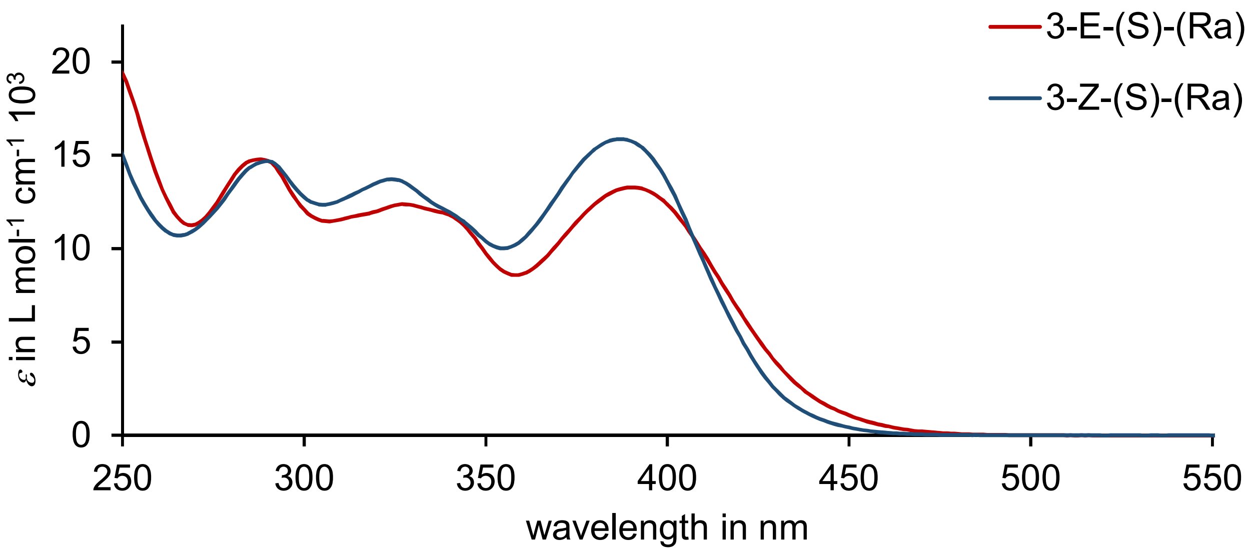

Supplementary Figure 66 Molar extinction coefficients. Obtained molar extinction coefficients *ε* of isomers **3**-*E*-(*S*)-(*R*$_a$) (red line) and **3**-*Z*-(*S*)-(*R*$_a$) (blue line) measured at 22 °C in CH_2Cl_2.

Isomers 4-*E*-(R_a/S_a) and 4-*Z*-(R_a/S_a)

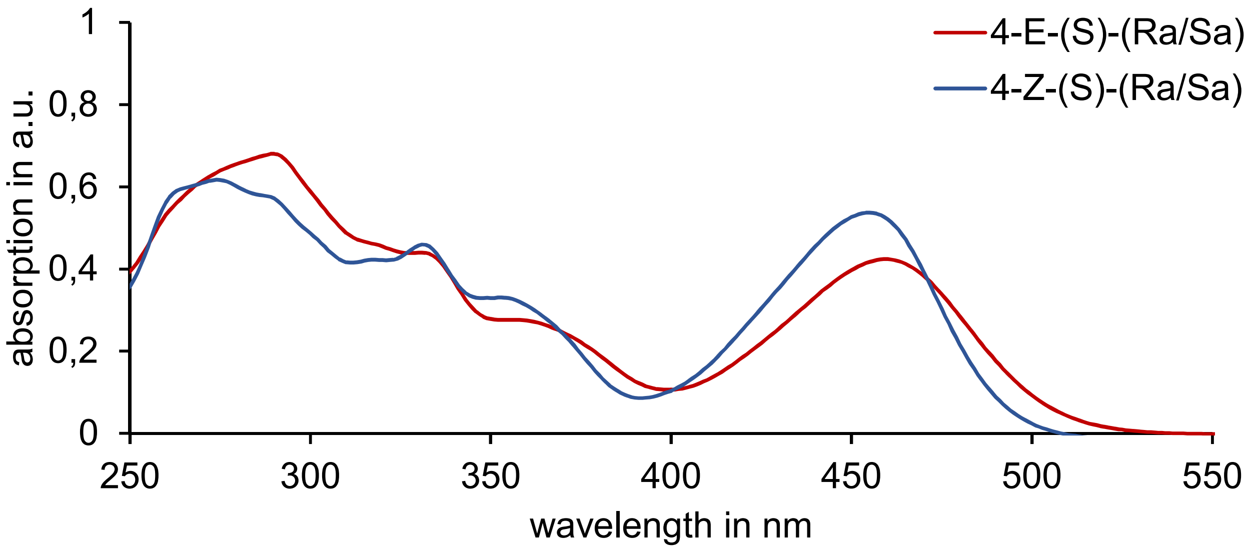

Supplementary Figure 67 Recorded UV-Vis absorption spectra spectra. UV-Vis spectra of isomer **4**-*E*-(R_a/S_a) (red line) scaled to fit the isosbestic point at 471 nm and **4**-*Z*-(R_a/S_a) (blue line) measured at 22 °C at a concentration of 31 µM in CH_2Cl_2.

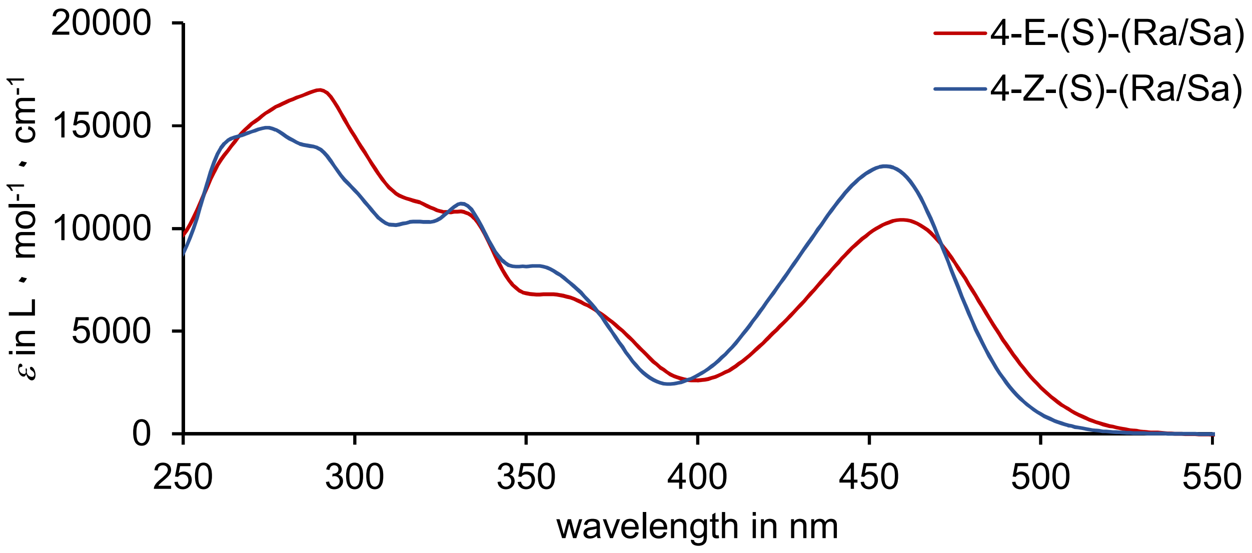

Supplementary Figure 68 Molar extinction coefficient. Obtained molar extinction coefficients ε of isomer **4**-*E*-(R_a/S_a) (red line) and **4**-*Z*-(R_a/S_a) (blue line) measured at 22 °C in CH_2Cl_2.

Isomers 5-*E*-(*R*a/*S*a) and 5-*Z*-(*R*a/*S*a)

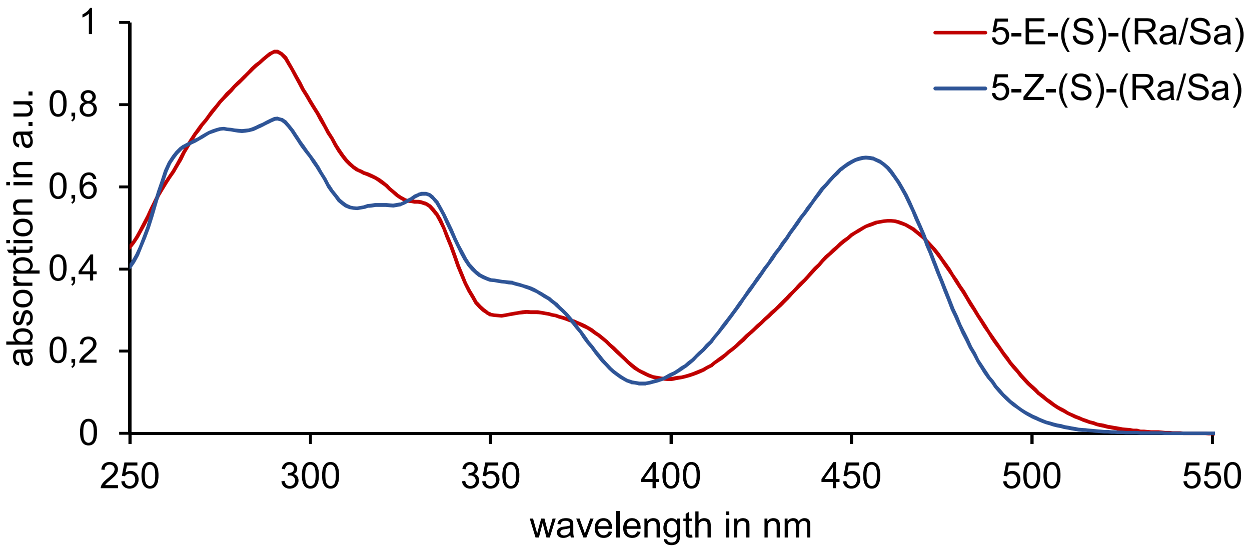

Supplementary Figure 69 Recorded UV-Vis absorption spectra spectra. UV-Vis spectra of isomer **5**-*E*-(R_a/S_a) (red line) scaled to fit the isosbestic point at 470 nm and **5**-*Z*-(R_a/S_a) (blue line) measured at 22 °C at a concentration of 35 µM in CH_2Cl_2.

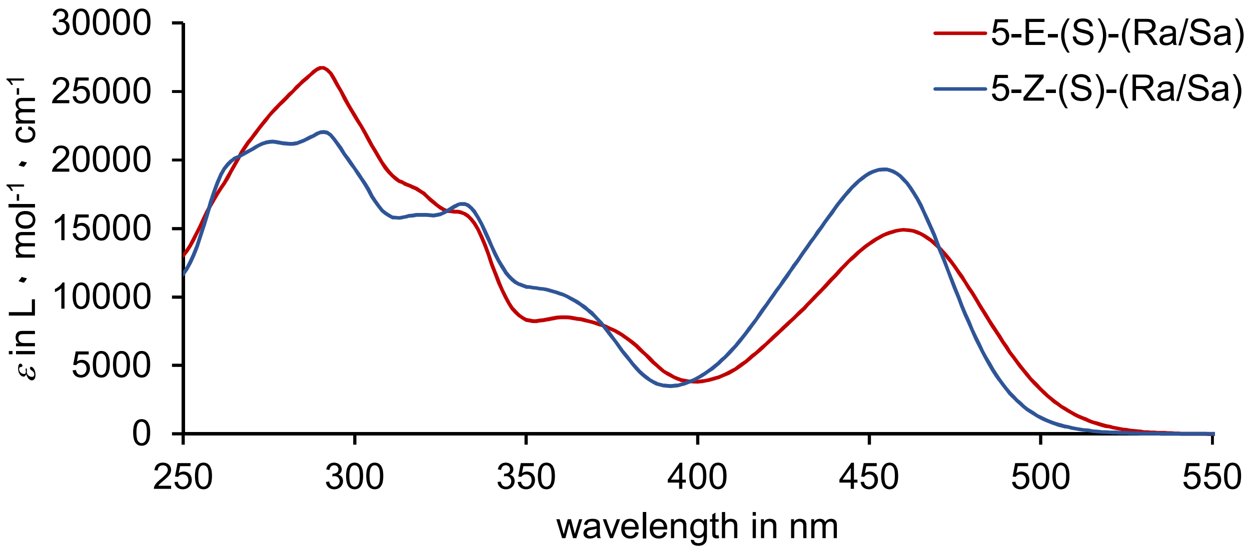

Supplementary Figure 70 Molar extinction coefficient. Obtained molar extinction coefficients ε of isomer **5**-*E*-(R_a/S_a) (red line) and **5**-*Z*-(R_a/S_a) (blue line) measured at 22 °C in CH_2Cl_2.

Supplementary Note 6: Quantum Yield Determination

All quantum yield measurements were carried out using an instrumental setup from the *Riedle* group.[1] The procedure involved filling a quartz cuvette of 1 cm path length with 2 mL of CH_2Cl_2 for which the illumination power P_{ill} was measured. The employed setup examines entire photoisomerization kinetics of a particular system until reaching the photostationary state to obtain the forwards and backwards quantum yields of a two-component system in one measurement. Therefore, pure and/or enriched macrocycles **2**-*E*-**I**, **2**-*E*-**II**, **3**-*E*-(*S*)-(R_a), **3**-*E*-(*S*)-(S_a) and **3**-*Z*-(*S*)-(R_a) motors, cyclic **4**-*Z*-(R_a)/(S_a), HTI **5**-*E*-(R_a)/(S_a) and **5**-*Z*-(R_a)/(S_a) were measured to deliver quantum yields for all isomers. A total volume of 2 mL of the corresponding CH_2Cl_2 solutions of each compound was filled into the cuvette and the absorption was adjusted to 0.6 – 1.7 a.u. at the particular irradiation wavelength. Whereas the solutions of macrocycle **2** and motor **3** were irradiated with a 400 nm LED, a 450 nm LED was used for the irradiation of cyclic **4** and HTI **5** in defined time intervals. After each irradiation step, the power of the solar cell detector was recorded and UV-Vis absorption spectrum was measured. The change in concentration, and hence the number of photoisomerized molecules was calculated from the predetermined molar extinction coefficients.

The quantum yield Φ of a photoisomerization step is calculated from the ratio of isomerized molecules *per* absorbed photons measured during the photoisomerization process as depicted in Equation 7:

$$\Phi = \frac{n\ (\text{photoisomerized molecules})}{n\ (\text{absorbed photons})} \tag{7}$$

The change in the number of photoisomerized molecules over a defined time interval can be calculated from the following Equations 8 and 9:

$$\begin{aligned}\frac{\mathrm{d}N_\mathrm{Z}(t)}{\mathrm{d}t} = &- \Phi_{\mathrm{Z}\rightarrow\mathrm{E}} \int \frac{c_\mathrm{Z}(t)\cdot\varepsilon_\mathrm{Z}(\lambda)}{c_\mathrm{Z}(t)\cdot\varepsilon_\mathrm{Z}(\lambda)+c_\mathrm{E}(t)\cdot\varepsilon_\mathrm{E}(\lambda)}\cdot\frac{P_\mathrm{ill}\cdot f(\lambda)\cdot\lambda}{h\cdot c}\cdot A(t,\lambda)\mathrm{d}\lambda \\ &+ \Phi_{\mathrm{E}\rightarrow\mathrm{Z}} \int \frac{c_\mathrm{E}(t)\cdot\varepsilon_\mathrm{Z}(\lambda)}{c_\mathrm{Z}(\mathrm{t})\cdot\varepsilon_\mathrm{Z}(\lambda)+c_\mathrm{E}(t)\cdot\varepsilon_\mathrm{E}(\lambda)}\cdot\frac{P_\mathrm{ill}\cdot f(\lambda)\cdot\lambda}{h\cdot c}\cdot A(t,\lambda)\mathrm{d}\lambda\end{aligned} \tag{8}$$

$$\frac{\mathrm{d}N_\mathrm{E}(t)}{\mathrm{d}t} = - \Phi_{\mathrm{E}\rightarrow\mathrm{Z}} \int \frac{c_\mathrm{E}(t)\cdot\varepsilon_\mathrm{E}(\lambda)}{c_\mathrm{Z}(t)\cdot\varepsilon_\mathrm{Z}(\lambda)+c_\mathrm{E}(t)\cdot\varepsilon_\mathrm{E}(\lambda)}\cdot\frac{P_\mathrm{ill}\cdot f(\lambda)\cdot\lambda}{h\cdot c}\cdot A(t,\lambda)\mathrm{d}\lambda \tag{9}$$

$$+\Phi_{Z\rightarrow E}\int\frac{c_Z(t)\cdot\varepsilon_Z(\lambda)}{c_Z(t)\cdot\varepsilon_Z(\lambda)+c_E(t)\cdot\varepsilon_E(\lambda)}\cdot\frac{P_{ill}\cdot f(\lambda)\cdot\lambda}{h\cdot c}\cdot A(t,\lambda)\mathrm{d}\lambda$$

Where N_Z is the number of molecules with *Z* configuration, N_E is the number of molecules with *E* configuration, $c_Z(t)$ stands for the concentration of the *Z* isomer at time *t*, $c_E(t)$ represents the concentration of the *E* isomer at time t, $\varepsilon_Z(\lambda)$ and $\varepsilon_E(\lambda)$ represent the molar extinction coefficients of the *Z* and *E* isomers at wavelength λ, respectively, P_{ill} stands for the illumination power, $f(\lambda)$ is the spectral distribution of the LED light, h the Planck's constant ($6.62607\cdot10^{-34}$ J·s), c the speed of light ($2.997925\cdot10^{8}$ $m\cdot s^{-1}$), and $A(t,\lambda)$ represents the absorbance at time *t* at wavelength λ.

The absorbance depends on the thickness of the cuvette d, the concentration c of the *Z* and *E* isomers and the corresponding extinction coefficients ε are shown in Equation 10:

$$A(t,\lambda)=1-10^{-d(c_Z(t)\cdot\varepsilon_Z(\lambda)+c_E(t)\cdot\varepsilon_E(\lambda))} \quad (10)$$

As stated above, both the forwards and backwards quantum yields $\Phi_{Z\rightarrow E}$ and $\Phi_{E\rightarrow Z}$, respectively, are calculated fitting the conversion between two species. This method therefore allows for extraction of quantum yields for both *Z* to *E* and *E* to *Z* photoisomerization processes from a single measurement.

Supplementary Table 3 Measured quantum yields Φ of macrocycle 2. Data was acquired at 25 °C in CH_2Cl_2 using a 400 nm LED for irradiation and corresponding error analysis. The data of each sample was considered normally distributed to calculate the confidence intervals, where mean quantum yields $\Phi_{aver.}$ are presented with a 95% confidence interval. [a]Values were obtained from the reverse reaction using experimental data starting from pure isomer of the corresponding opposed double bond configuration.

Isomer	Measurement #	Φ (%)	$\Phi_{aver.}$ (%)	Population Standard Deviation (σ)	Standard Error (σ_M)	Confidence Interval (CI)
**C 2-*E*-I**	1	3.38	3.45	0.06	0.06	3.45 ± 0.09
	2	3.51				
**D 2-Z-I**	1[a]	16.18	16.13	0.05	0.05	16.13 ± 0.08
	2[a]	16.07				
**A 2-*E*-II**	1	4.26	4.37	0.11	0.11	4.37 ± 0.15
	2	4.47				
**B 2-Z-II**	1[a]	9.06	9.26	0.20	0.20	9.26 ± 0.28
	2[a]	9.46				

Supplementary Table 4 Measured quantum yields Φ of non-macrocyclic 3. Data was acquired at 25 °C in CH_2Cl_2 using a 400 nm LED for irradiation and corresponding error analysis. The data of each sample was considered normally distributed to calculate the confidence intervals, where mean quantum yields $\Phi_{aver.}$ are presented with a 95% confidence interval. [a]Values were obtained from the reverse reaction using experimental data starting from pure isomer of the corresponding opposed double bond configuration. [b]Values were averaged including the corresponding triple measurements.

Isomer	Measurement #	Φ (%)	$\Phi_{aver.}$ (%)	Population Standard Deviation (σ)	Standard Error (σ_M)	Confidence Interval (CI)
**3**-*E*-(*S*)-(*S*$_a$)	1	4.15	3.99	0.16	0.16	3.99 ± 0.22
	2	3.83				
**3**-*Z*-(*S*)-(*S*$_a$)	1[a]	6.95	6.99	0.04	0.04	6.99 ± 0.06
	2[a]	7.03				
**3**-*E*-(*S*)-(*R*$_a$)	1	4.88	4.77	0.16	0.12	4.86 ± 0.19
	2	4.65				
	3[a]	5.05	4.86[b]			
**3**-*Z*-(*S*)-(*R*$_a$)	1[a]	7.89	7.82	0.06	0.04	7.81 ± 0.07
	2[a]	7.75				
	3	7.79	7.81[b]			

Supplementary Table 5 Measured quantum yields Φ of macrocycle 4 and HTI 5. Data was acquired at 25 °C in CH_2Cl_2 using a 450 nm LED for irradiation and corresponding error analysis. The data of each sample was considered normally distributed to calculate the confidence intervals, where mean quantum yields $\Phi_{aver.}$ are presented with a 95% confidence interval. [a]Values were obtained from the reverse reaction using experimental data starting from pure isomer of the corresponding opposed double bond configuration. [b]Values were averaged including the corresponding triple measurements.

Isomer	Measurement #	Φ (%)	$\Phi_{aver.}$ (%)	Population Standard Deviation (σ)	Standard Error (σ_M)	Confidence Interval (CI)
**4**-*E*-(R_a/S_a)	1	1.41	1.41	0.02	0.02	1.41 ± 0.02
	2	1.42				
**4**-*Z*-(R_a/S_a)	1[a]	4.26	4.37	0.10	0.10	4.37 ± 0.14
	2[a]	4.47				
**5**-*E*-(R_a/S_a)	1	3.64	3.74	0.07	0.05	3.73 ± 0.08
	2	3.83				
	3[a]	3.65	3.73[b]			
**5**-*Z*-(R_a/S_a)	1[a]	2.58	2.71	0.18	0.13	2.60 ± 0.20
	2[a]	2.83				
	3	2.45	2.60[b]			

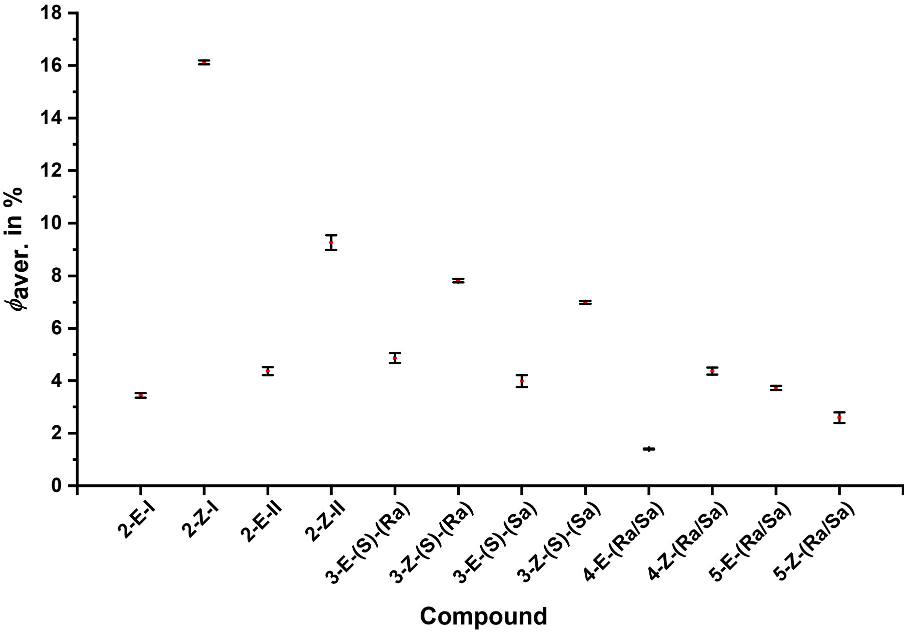

Supplementary Figure 71 Average values of quantum yields $\phi_{aver.}$ for all compounds. Error bars for each point represent 95% confidential levels, where significance level α is equal to 0.05. Each data point is represented by red dots.

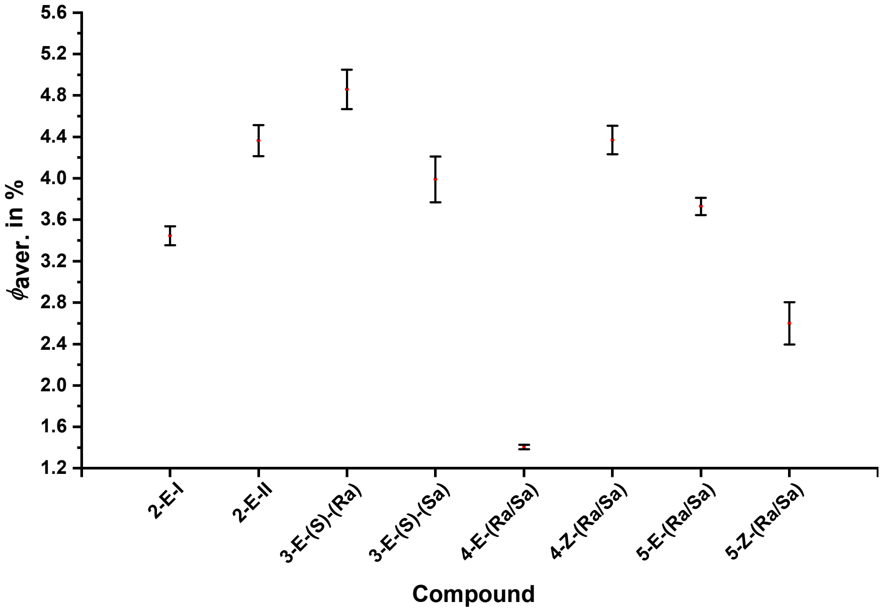

Supplementary Figure 72 Average values of quantum yields $\phi_{aver.}$ Values for macrocycles **2**-*E*-I, **2**-*E*-II, motors **3**-*E*-(*S*)-(R_a) and **3**-*E*-(*S*)-(S_a), cyclic **4**-*E*-(R_a)/(S_a) and **4**-*Z*-(R_a)/(S_a), as well as HTI **5**-*E*-(R_a)/(S_a) and **5**-*Z*-(R_a)/(S_a) are shown. Error bars for each point represent 95% confidential levels, where significance level α is equal to 0.05. Each data point is represented by red dots.

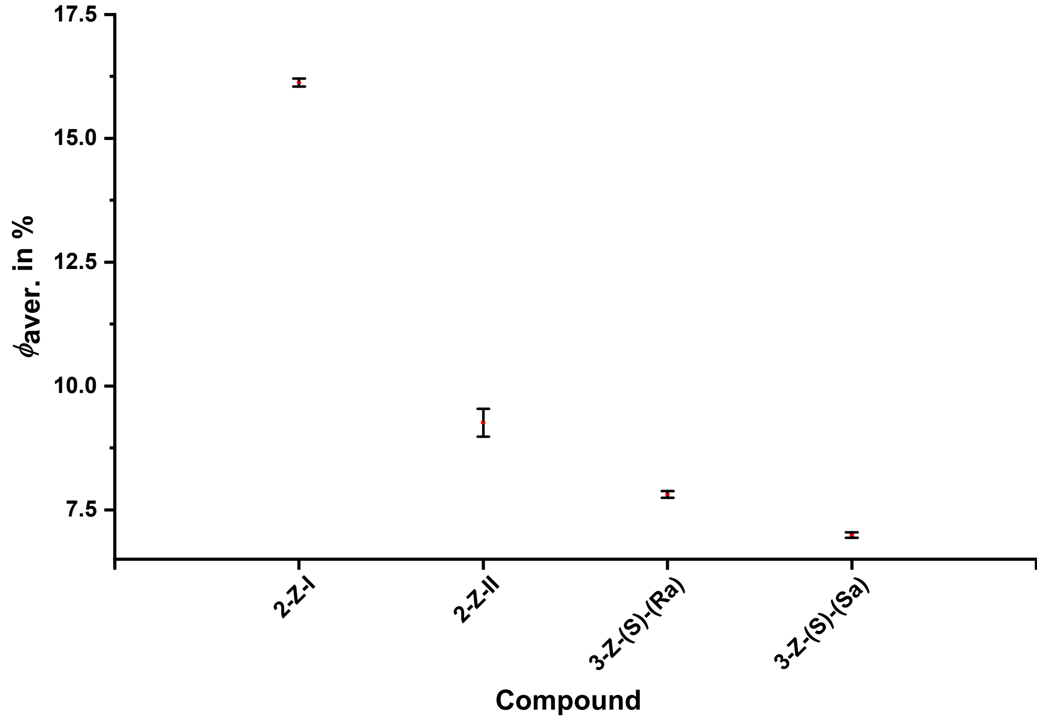

Supplementary Figure 73 Average values of quantum yields ϕ_{mean}. Values for macrocycles **2**-*Z*-**I**, **2**-*Z*-**II**, motors **3**-*Z*-(*S*)-(*R*$_a$) and **3**-*Z*-(*S*)-(*S*$_a$) are shown. Error bars for each point represent 95% confidential levels, where the significance level α is equal to 0.05. Each data point is represented by red dots.

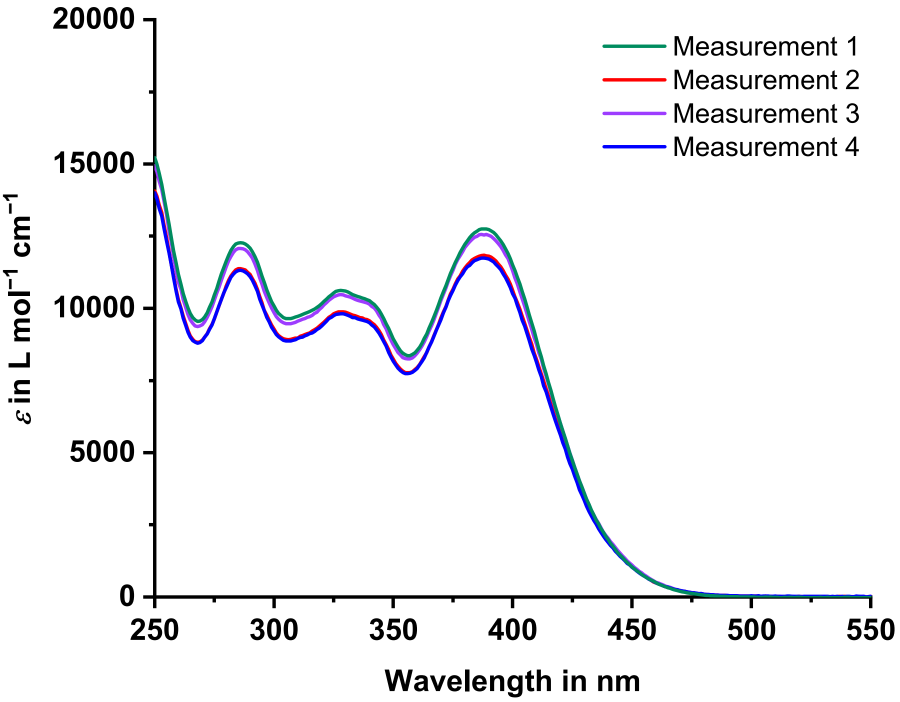

Supplementary Figure 74 Different extinction coefficient *ε* measurements of macrocycle 2-*E*-I recorded in CH_2Cl_2 at different concentrations. The concentrations for measurements 1 (green), 2 (red), 3 (purple) and 4 (blue) are 56.4 µM, 52.6 µM, 55.4 µM and 42 µM, respectively.

Supplementary Table 6 Impact of molar extinction coefficient measurements on quantum yield ϕ of macrocycle 1. Four different data sets obtained from repeated extinction coefficient ε measurements were employed for quantum yield determination. Each data series is considered normally distributed to calculate 95% confidence intervals.

Isomer	Measurement of ε (#)	Measurement of Φ (#)	Φ (%)	$\Phi_{aver.}$ (%)	Population Standard Deviation (σ)	Standard Error (σ_M)	Confidence Interval (CI)
**2**-*E*-**I**	1	1	3.38	4.04	0.39	0.22	4.04 ± 0.38
	2		4.37				
	3		4.17				
	4		4.22				
**2**-*E*-**I**	1	2	3.51	4.17	0.39	0.22	4.17 ± 0.38
	2		4.50				
	3		4.29				
	4		4.36				
**2**-*Z*-**I**	1	1	16.18	15.73	0.27	0.15	15.73 ± 0.26
	2		15.53				
	3		15.55				
	4		15.65				
**2**-*Z*-**I**	1	2	16.07	15.61	0.27	0.16	15.61 ± 0.27
	2		15.41				
	3		15.42				
	4		15.54				

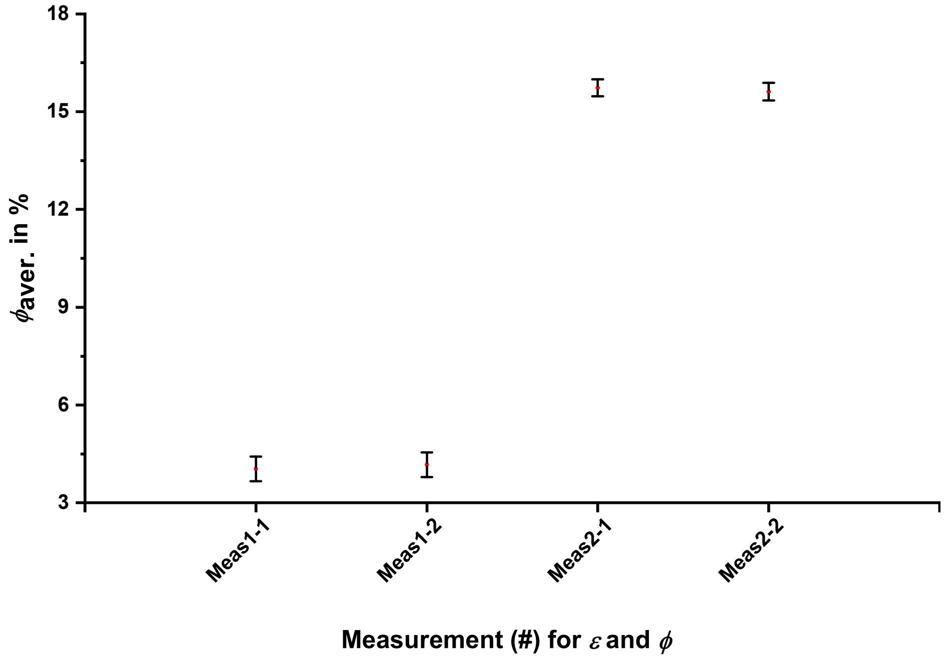

Supplementary Figure 75 Mean values of quantum yields $\phi_{\text{aver.}}$. Values for macrocycles **2**-*E*-**I** and **2**-*Z*-**I** determined by using four different extinction coefficient ε measurements are shown. Error bars for each point represent 95% confidential levels, where significance level α is equal to 0.05. Each data point is represented by red dots.

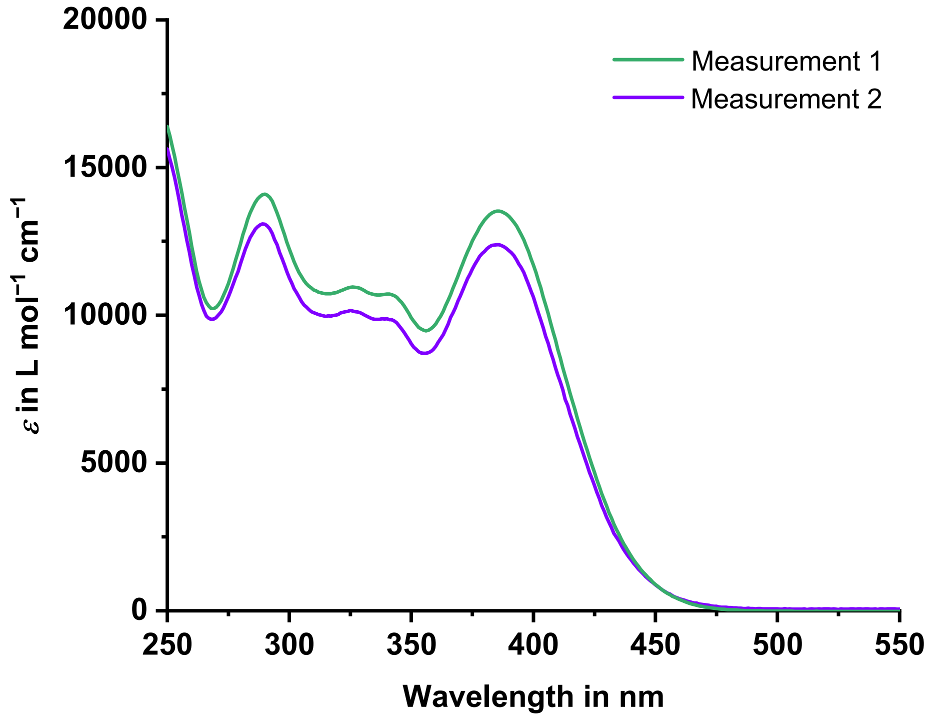

Supplementary Figure 76 Two different extinction coefficient ε measurements of macrocycle 2-*E*-II recorded in CH_2Cl_2. The concentrations for measurements 1 (green) and 2 (purple) are 47 µM and 55.3 µM, respectively.

Supplementary Table 7 Impact of molar extinction coefficient measurements on quantum yield ϕ of macrocycle 2. Two different data sets obtained from repeated extinction coefficient ε measurements were employed for quantum yield determination. Each data series is considered normally distributed to calculate 95% confidence intervals.

Isomer	Measurement of ε (#)	Measurement of Φ (#)	Φ (%)	$\Phi_{aver.}$ (%)	Population Standard Deviation (σ)	Standard Error (σ_M)	Confidence Interval (CI)
**2-*E*-II**	1<br>2	1<br>2	4.26<br>4.47	4.37	0.11	0.11	4.37 ± 0.15
**2-*E*-II**	1<br>2	1<br>2	4.69<br>5.84	5.27	0.57	0.57	5.27 ± 0.80
**2-*Z*-II**	1<br>2	1<br>2	9.06<br>9.46	9.26	0.20	0.20	9.26 ± 0.28
**2-*Z*-II**	1<br>2	1<br>2	8.92<br>10.17	9.55	0.63	0.63	9.55 ± 0.87

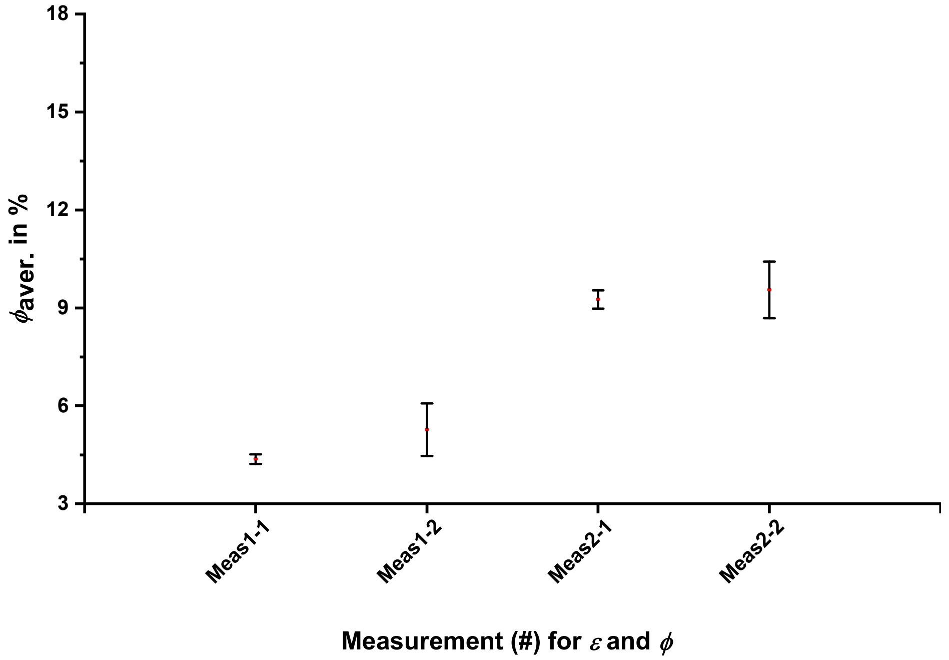

Supplementary Figure 77 Mean values of quantum yields $\phi_{\text{aver.}}$. Values for macrocycles **2**-*E*-**II** and **2**-*Z*-**II** determined by using four different extinction coefficient ε measurements are shown. Error bars for each point represent 95% confidential levels, where significance level α is equal to 0.05. Each data point is represented by red dots.

Quantum Yield of 2-*E*-I and 2-*Z*-I

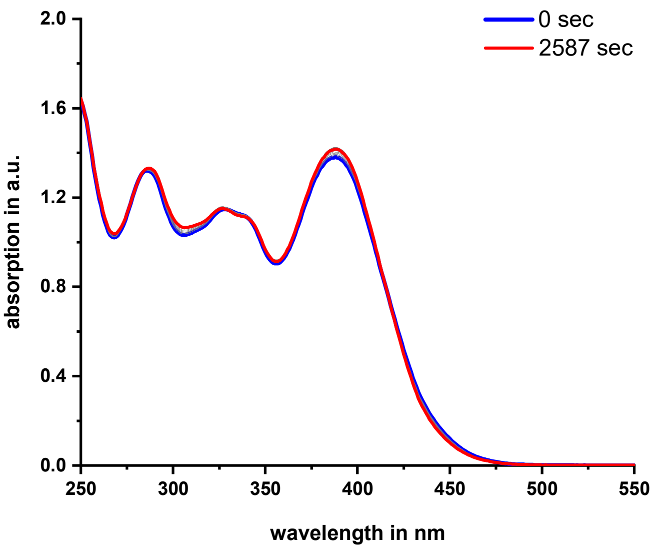

Supplementary Figure 78 Overview of spectra obtained during quantum yield measurement. Stacked UV-Vis absorption spectra measured in defined time intervals during irradiation with 400 nm light, starting from a CH_2Cl_2 solution of pure **2**-*E*-**I**. Only initial (blue) and final (red) spectra are highlighted for reasons of clarity, whereas grey lines stand for the spectra recorded in the interim.

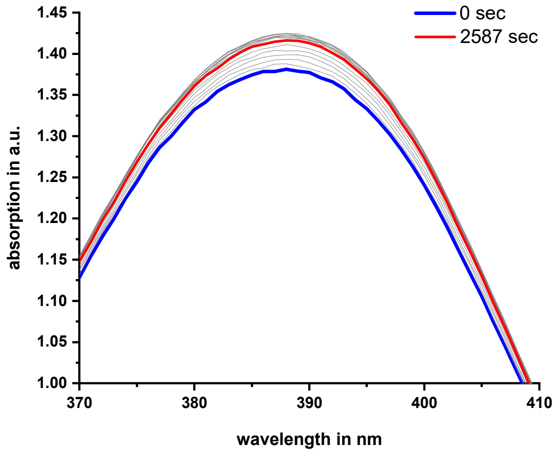

Supplementary Figure 79 Enlargement of spectra obtained during quantum yield measurement. Stacked UV-Vis absorption spectra measured in defined time intervals during irradiation with 400 nm light, starting from a CH_2Cl_2 solution of pure **2**-*E*-**I**. Only initial (blue) and final (red) spectra used for analysis are highligted for reasons of clarity, whereas grey lines stand for the spectra recorded in the interim.

Quantum Yield of 2-*E*-II and 2-*Z*-II

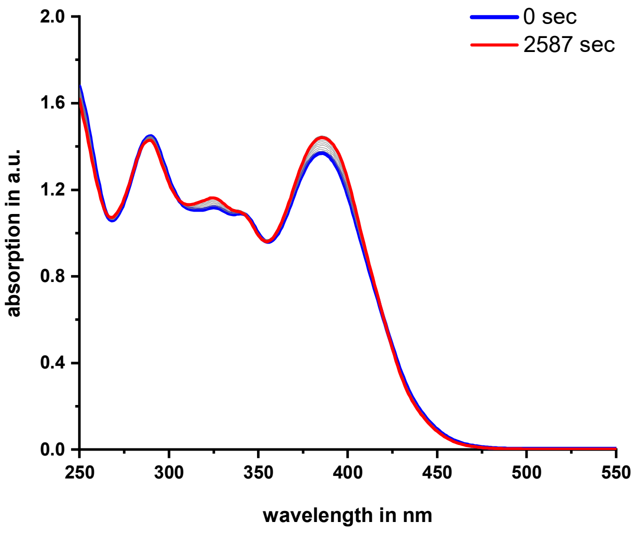

Supplementary Figure 80 Overview of spectra obtained during quantum yield measurement. Stacked UV-Vis absorption spectra measured in defined time intervals during irradiation with 400 nm light, starting from a CH_2Cl_2 solution of pure **2**-*E*-**II**. Only initial (blue) and final (red) spectra are highlighted for reasons of clarity. Grey lines depict the spectra recorded in the interim.

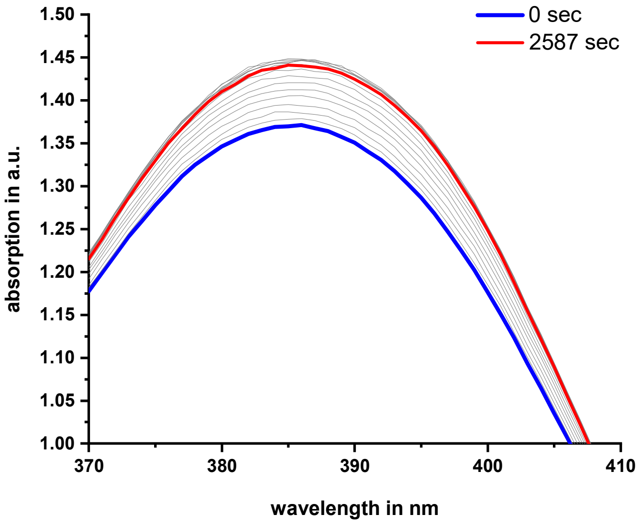

Supplementary Figure 81 Enlargement of spectra obtained during quantum yield measurement. Stacked UV-Vis absorption spectra measured in defined time intervals during irradiation with 400 nm light, starting from a CH_2Cl_2 solution of pure **2**-*E*-**II**. Only initial (blue) and final (red) spectra used for analysis are highligted for reasons of clarity. Grey lines depict the spectra recorded in the interim.

Quantum Yield of 3-*E*-(*S*)-(S_a) and 3-*Z*-(*S*)-(S_a)

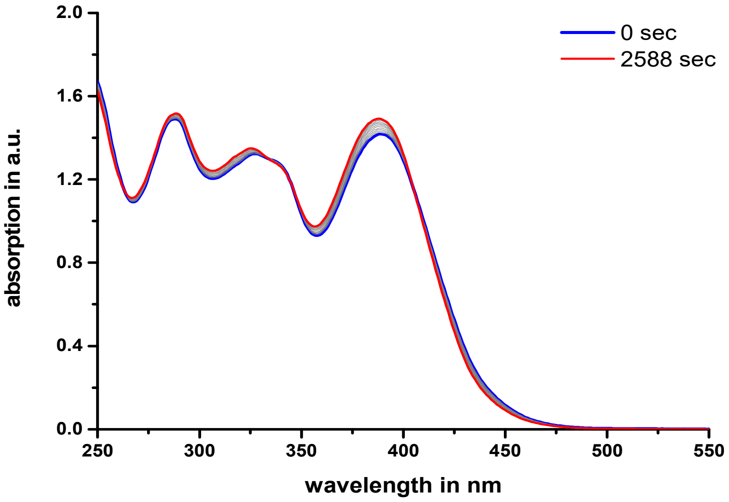

Supplementary Figure 82 Overview of spectra obtained during quantum yield measurement. Stacked UV-Vis absorption spectra measured in defined time intervals during irradiation with 400 nm light, starting from a CH_2Cl_2 solution of enriched **3**-*E*-(*S*)-(S_a). Only initial (blue) and final (red) spectra used for analysis are highlighted for reasons of clarity, whereas grey lines present the spectra recorded in the interim.

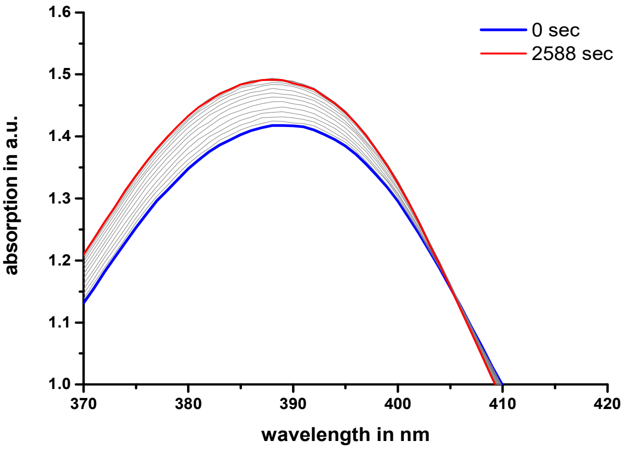

Supplementary Figure 83 Enlargement of spectra obtained during quantum yield measurement. Stacked UV-Vis absorption spectra measured in defined time intervals during irradiation with 400 nm light, starting from a CH_2Cl_2 solution of enriched **3**-*E*-(*S*)-(S_a). Only initial (blue) and final (red) spectra used for analysis are highlighted for reasons of clarity, whereas grey lines present the spectra recorded in the interim.

Quantum Yield of 3-*E*-(*S*)-(*R*a) and 3-*Z*-(*S*)-(*R*a)

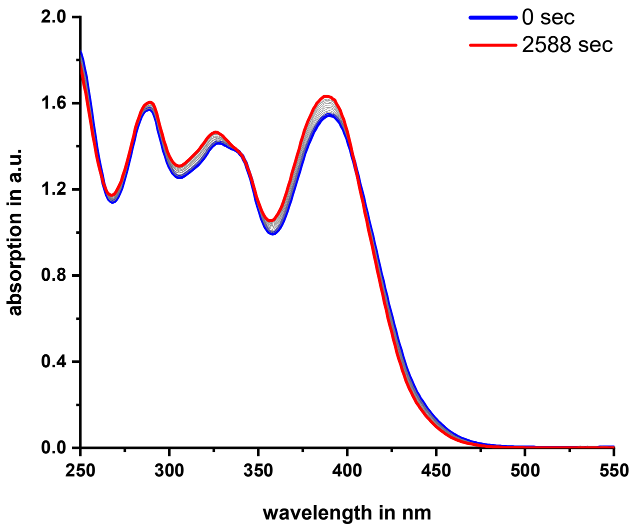

Supplementary Figure 84 Overview of spectra obtained during quantum yield measurement. Stacked UV-Vis absorption spectra measured in defined time intervals during irradiation with 400 nm light, starting from a CH_2Cl_2 solution of pure **3**-*E*-(*S*)-(*R*a). Only initial (blue) and final (red) spectra are highlighted for reasons of clarity. Grey lines illustrate the spectra recorded in the interim.

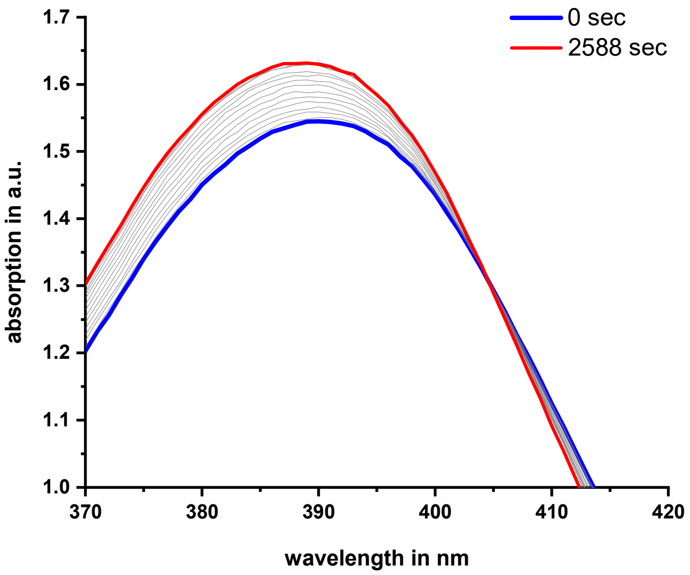

Supplementary Figure 85 Enlargement of spectra obtained during quantum yield measurement. Stacked UV-Vis absorption spectra measured in defined time intervals during irradiation with 400 nm light, starting from a CH_2Cl_2 solution of pure **3**-*E*-(*S*)-(*R*a). Only initial (blue) and final (red) spectra used for analysis are highlighted for reasons of clarity. Grey lines illustrate the spectra recorded in the interim.

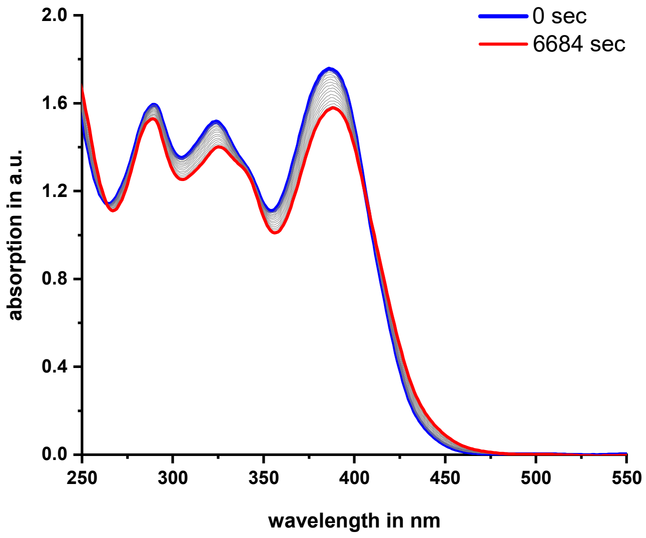

Supplementary Figure 86 Overview of spectra obtained during quantum yield measurement. Stacked UV-Vis absorption spectra measured in defined time intervals during irradiation with 400 nm light, starting from a CH_2Cl_2 solution of pure **3**-*Z*-(*S*)-(R_a). Only initial (blue) and final (red) spectra are highlighted for reasons of clarity, whereas time intervals different than initial and final measurements are presented as grey lines.

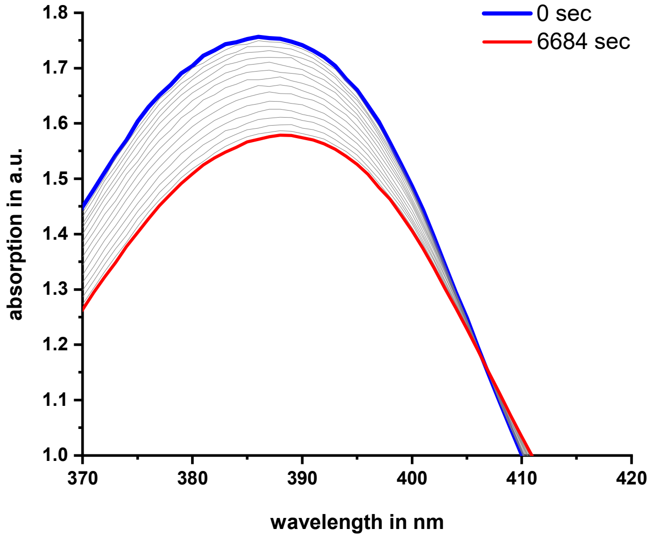

Supplementary Figure 87 Enlargement of spectra obtained during quantum yield measurement. Stacked UV-Vis absorption spectra measured in defined time intervals during irradiation with 400 nm light, starting from a CH_2Cl_2 solution of enriched **3**-*Z*-(*S*)-(R_a). Only initial (blue) and final (red) spectra used for analysis are highlighted for reasons of clarity, whereas time intervals different than initial and final measurements are presented as grey lines.

Quantum Yield of 4-*Z*-(R_a/S_a) and 4-*E*-(R_a/S_a)

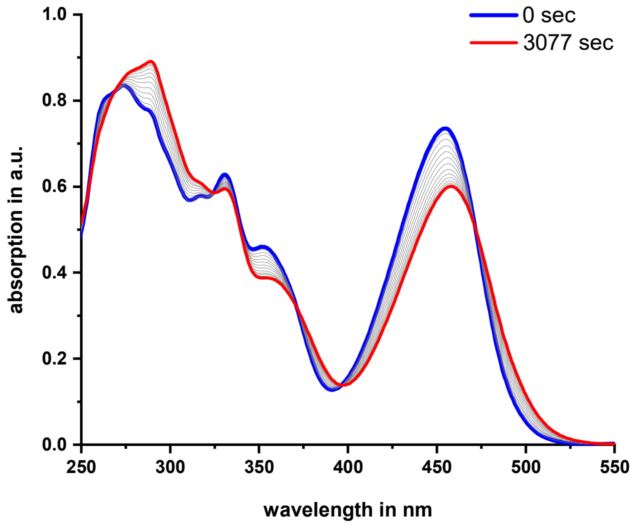

Supplementary Figure 88 Overview of spectra obtained during quantum yield measurement. Stacked UV-Vis absorption spectra measured in defined time intervals during irradiation with 450 nm light, starting from a CH_2Cl_2 solution of enriched **4**-*Z*-(R_a/S_a). Only initial (blue) and final (red) spectra are highlighted for reasons of clarity. Grey spectra correspond to measurements different than initial and final intervals.

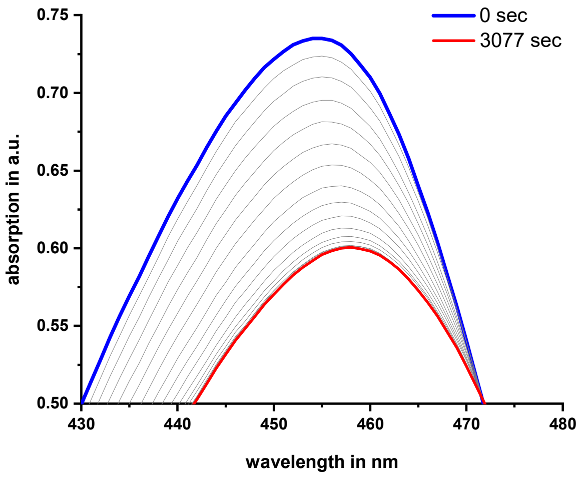

Supplementary Figure 89 Enlargement of spectra obtained during quantum yield measurement. Stacked UV-Vis absorption spectra measured in defined time intervals during irradiation with 450 nm light, starting from a CH_2Cl_2 solution of enriched **4**-*Z*-(R_a/S_a). Only initial (blue) and final (red) spectra used for analysis are highlighted for reasons of clarity. Grey spectra correspond to measurements different than initial and final intervals.

Quantum Yield of 5-*E*-(R_a/S_a) and 5-*Z*-(R_a/S_a)

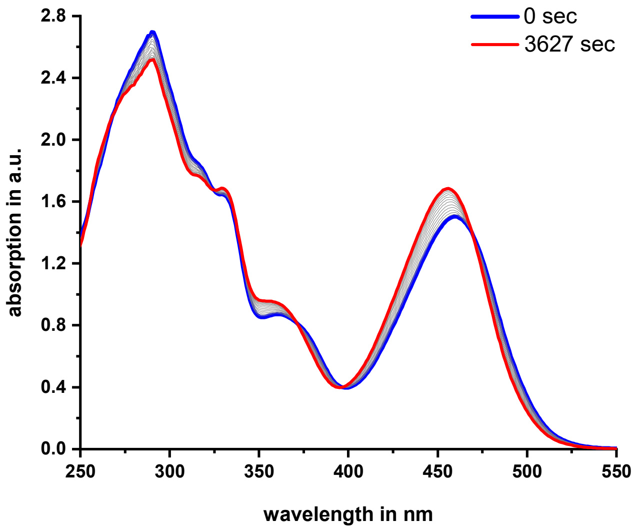

Supplementary Figure 90 Overview of spectra obtained during quantum yield measurement. Stacked UV-Vis absorption spectra measured in defined time intervals during irradiation with 450 nm light, starting from a CH_2Cl_2 solution of enriched **5**-*E*-(R_a/S_a). Only initial (blue) and final (red) spectra are highlighted for reasons of clarity, whereas grey lines indicate spectra recorded different than initial and final intervals.

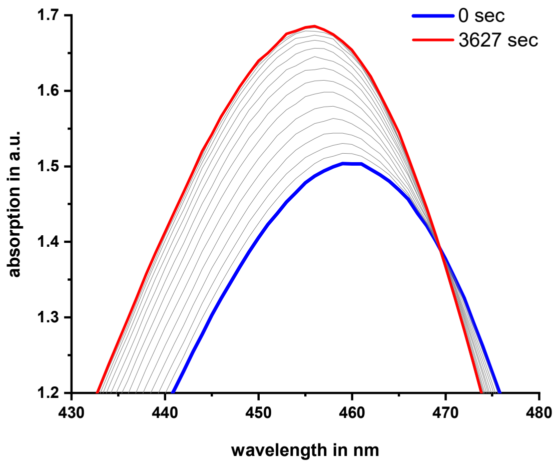

Supplementary Figure 91 Enlargement of spectra obtained during quantum yield measurement. Stacked UV-Vis absorption spectra measured in defined time intervals during irradiation with 450 nm light, starting from a CH_2Cl_2 solution of enriched **5**-*E*-(R_a/S_a). Only initial (blue) and final (red) spectra used for analysis are highlighted for reasons of clarity, whereas grey lines indicate spectra recorded different than initial and final intervals.

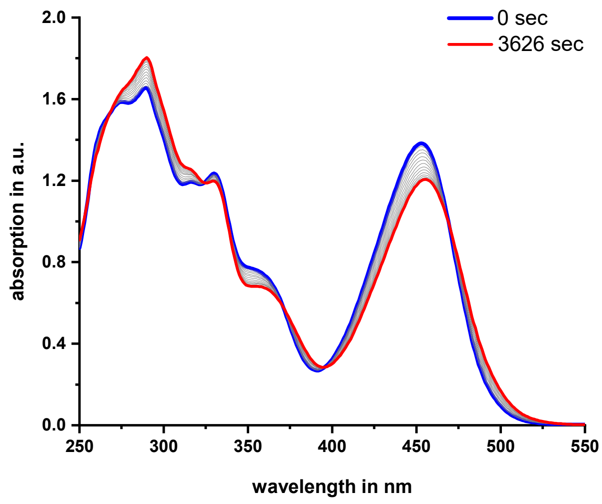

Supplementary Figure 92 Overview of spectra obtained during quantum yield measurement. Stacked UV-Vis absorption spectra measured in defined time intervals during irradiation with 450 nm light, starting from a CH_2Cl_2 solution of enriched **5**-*Z*-(R_a/S_a). Only initial (blue) and final (red) spectra are highlighted for reasons of clarity. Spectra recorded different than initial and final intervals are depicted in grey.

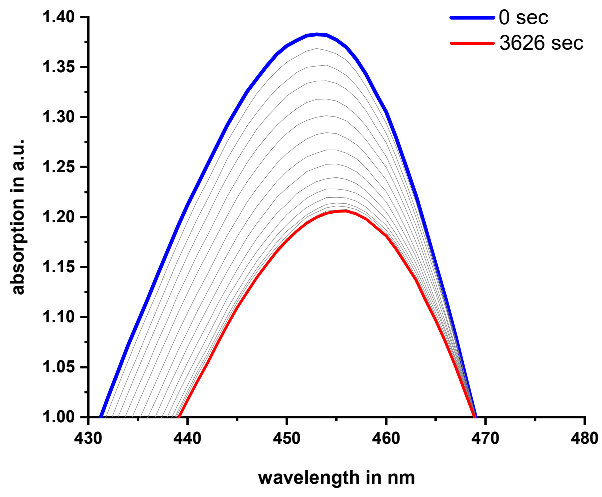

Supplementary Figure 93 Enlargement of spectra obtained during quantum yield measurement. Stacked UV-Vis absorption spectra measured in defined time intervals during irradiation with 450 nm light, starting from a CH_2Cl_2 solution of enriched **5**-*Z*-(R_a/S_a). Only initial (blue) and final (red) spectra used for analysis are highlighted for reasons of clarity. Spectra recorded different than initial and final intervals are depicted in grey.

Supplementary Note 7: Theoretical Description

All *ab initio* calculations were performed using the Gaussian16 Revision B.01 or C.01 program package.[3] Ground state and transition state geometries of compounds **1** and **3** were optimized at 25 °C on the B3LYP/6-311G(d,p) IEFPCM (CH_2Cl_2) level of theory. A subsequent frequency analysis of the ground state geometries revealed no imaginary frequencies, confirming that all structures were minima. Frequency analysis of the transition state geometries revealed only one imaginary frequency for each structure confirming only maxima were found.

Theoretical obtained Energies

Supplementary Table 8 Minimum- and transition state energies of molecular motor 1. Data were acquired on the B3LYP/6-311G(d,p) IEFPCM (CH_2Cl_2) level of theory.

Isomer	G_0 (Hartree)	ΔG (Hartree)	ΔG (kcal mol^{-1})
**1**-*Z*-(*S*)-(*P*)	-1282.2405	0	0
**1**-*Z*-(*S*)-(*M*)	-1282.2395	0.000941	0.590007
**1**-*E*-(*S*)-(*P*)	-1282.2397	0.000817	0.512259
**1**-*E*-(*S*)-(*M*)	-1282.2370	0.003496	2.191992
TS-**1**-*E*-(*S*)-(*M*/*P*)	-1282.2352	0.005271	3.304917
TS-**1**-*Z*-(*S*)-(*M*/*P*)	-1282.2365	0.003956	2.480412

Supplementary Table 9 Minimum- and transition state energies for molecular motors 3-(*S*)-(*S*a) and 3-(*S*)-(*R*a). Data were acquired on the B3LYP/6-311G(d,p) IEFPCM (CH_2Cl_2) level of theory.

Isomer	G_0 (Hartree)	ΔG (Hartree)	ΔG (kcal mol^{-1})
**3**-*Z*-(*S*)-(*P*)-(*S*a)	-1920.1997	0	0
**3**-*Z*-(*S*)-(*M*)-(*S*a)	-1920.1983	0.001405	0.880935
**3**-*E*-(*S*)-(*P*)-(*S*a)	-1920.1978	0.001918	1.202586
**3**-*E*-(*S*)-(*M*)-(*S*a)	-1920.1955	0.004228	2.650956
TS-**3**-*E*-(*S*)-(*M*/*P*)-(*S*a)	-1920.1934	0.006328	3.924393
TS-**3**-*Z*-(*S*)-(*M*/*P*)-(*S*a)	-1920.1944	0.005329	3.298020
**3**-*Z*-(*S*)-(*P*)-(*R*a)	-1920.1997	0	0
**3**-*Z*-(*S*)-(*M*)-(Ra)	-1920.1978	0.001902	1.192554
**3**-*E*-(*S*)-(*P*)-(*R*a)	-1920.1981	0.001544	0.968088
**3**-*E*-(*S*)-(*M*)-(Ra)	-1920.1958	0.003858	2.418966
TS-**3**-*E*-(*S*)-(*M*/*P*)-(*R*a)	-1920.1934	0.006307	3.954489
TS-**3**-*Z*-(*S*)-(*M*/*P*)-(*R*a)	-1920.1946	0.005060	3.172620

Ground State Geometries

Supplementary Table 10 Calculated xyz coordinates. Atomic coordinates of the *Z*-(*S*)-(*P*) and *Z*-(*S*)-(*M*) isomers of molecular motor **1**.

***Z*-(*S*)-(*P*)**				***Z*-(*S*)-(*M*)**			
C	1.023962	0.495110	-0.037825	C	1.030919	0.498237	0.050414
C	-0.315739	0.241684	-0.118417	C	-0.300572	0.213436	0.168189
C	2.106483	-0.492205	-0.153740	C	2.126398	-0.479771	-0.007828
C	3.323165	0.079864	0.255784	C	3.305835	0.147881	-0.447423
C	3.110003	1.480814	0.735866	C	3.057081	1.594114	-0.737088
C	1.685496	1.870038	0.226131	C	1.665447	1.904815	-0.098346
C	2.095375	-1.794771	-0.682565	C	2.160133	-1.832590	0.377410
C	3.283642	-2.507795	-0.758143	C	3.351747	-2.538271	0.260420
C	4.485962	-1.937908	-0.321836	C	4.510168	-1.913691	-0.214058
C	4.511945	-0.638925	0.179847	C	4.495162	-0.563283	-0.558368
C	0.980746	2.733150	1.284906	C	1.869211	2.513898	1.309321
C	1.81859	2.635533	-1.112322	C	0.905458	2.883243	-1.009264
C	-1.435968	1.188055	-0.333221	C	-1.431345	1.141958	0.427478
C	-2.739259	0.487700	-0.220904	C	-2.722787	0.493114	0.084516
C	-2.640592	-0.878569	0.006542	C	-2.60705	-0.845038	-0.268186
S	-0.939095	-1.486665	0.065848	S	-0.920232	-1.496706	-0.105762
C	-3.99377	1.090032	-0.329843	C	-3.972648	1.110390	0.091063
C	-5.128517	0.300940	-0.177778	C	-5.089480	0.360815	-0.268410
C	-5.014257	-1.073371	0.070075	C	-4.959230	-0.987768	-0.618686
C	-3.763205	-1.682508	0.160598	C	-3.709737	-1.611290	-0.616307
O	-1.344484	2.368771	-0.640696	O	-1.353606	2.270493	0.886718
O	-0.648632	-1.977193	1.477322	O	-0.915711	-2.312295	1.186360
H	3.135813	1.509714	1.830613	H	3.840472	2.246515	-0.342967
H	3.874862	2.174798	0.378603	H	3.016611	1.760392	-1.819067
H	1.182865	-2.24124	-1.052175	H	1.294721	-2.317274	0.809278
H	5.404989	-2.508951	-0.389197	H	5.431510	-2.479812	-0.292264
H	5.446672	-0.191953	0.499264	H	5.400159	-0.072921	-0.899305
H	0.039350	3.13755	0.922610	H	0.908386	2.671465	1.798045
H	1.633787	3.570631	1.548025	H	2.378224	3.477531	1.217458

H	0.793107	2.156734	2.194790	H	2.483608	1.862861	1.936828
H	2.341510	3.580021	-0.937275	H	1.556511	3.735904	-1.223706
H	2.394619	2.058207	-1.840522	H	0.649689	2.412787	-1.962776
H	0.836137	2.852932	-1.528606	H	-0.00236	3.260743	-0.547387
H	-3.674923	-2.746940	0.342025	H	-3.611263	-2.657962	-0.877985
H	3.280787	-3.509838	-1.170109	H	3.387001	-3.578482	0.562121
H	-4.062326	2.154337	-0.52024	H	-6.069382	0.822851	-0.277473
H	-6.111955	0.749716	-0.250716	H	-5.838784	-1.557773	-0.893797
H	-5.909209	-1.672914	0.187406	H	-4.055109	2.153694	0.371480

Supplementary Table 11 Calculated xyz coordinates. Atomic coordinates of *E*-(*S*)-(*P*) and *E*-(*S*)-(*M*) isomers of molecular motor **1**.

***E*-(*S*)-(*P*)**				***E*-(*S*)-(*M*)**			
C	-1.043697	0.342529	-0.118495	C	1.062149	0.356682	-0.11489
C	0.300441	0.100519	-0.133406	C	-0.276873	0.135144	-0.279247
C	-2.12628	-0.645306	0.039473	C	2.153412	-0.637171	-0.047096
C	-3.353176	-0.057408	-0.320637	C	3.3926	0.024065	0.052078
C	-3.142985	1.349706	-0.78423	C	3.224664	1.50536	-0.027705
C	-1.731135	1.725538	-0.240694	C	1.70033	1.751024	0.154898
C	-2.112761	-1.947415	0.566361	C	2.128676	-2.043451	0.017232
C	-3.309841	-2.640388	0.691968	C	3.317444	-2.743201	0.173679
C	-4.5203	-2.058313	0.297848	C	4.541944	-2.071611	0.260515
C	-4.5477	-0.759827	-0.205582	C	4.5822	-0.682347	0.200124
C	-1.073503	2.745235	-1.184795	C	1.42614	2.150611	1.625385
C	-1.881526	2.331332	1.179008	C	1.265	2.876733	-0.802099
C	1.003356	-1.198137	-0.326149	C	-1.004818	-1.134223	-0.568759
C	2.471647	-1.018031	-0.213911	C	-2.42322	-1.019778	-0.140207
C	2.87401	0.293368	-0.005253	C	-2.792032	0.259246	0.254277
S	1.51247	1.482006	-0.025082	S	-1.500892	1.496469	-0.049491
C	3.421162	-2.038764	-0.286247	C	-3.346265	-2.062713	-0.087911
C	4.762844	-1.714392	-0.11919	C	-4.631833	-1.792297	0.374273
C	5.15308	-0.388363	0.110539	C	-4.990671	-0.498323	0.768395
C	4.209242	0.63677	0.164387	C	-4.071383	0.551092	0.703855
O	0.488759	-2.269714	-0.60524	O	-0.549222	-2.140513	-1.085134
O	1.43497	2.145487	1.345566	O	-1.854827	2.129392	-1.391653
H	-3.143944	1.393401	-1.879315	H	3.815816	2.040964	0.719437
H	-3.911976	2.04083	-0.430731	H	3.551055	1.865942	-1.009389
H	-1.189266	-2.411908	0.871768	H	1.201701	-2.584314	-0.067753
H	-5.443525	-2.61775	0.399314	H	5.460162	-2.636064	0.378011
H	-5.487056	-0.298979	-0.490079	H	5.526386	-0.154222	0.273524
H	-0.197588	3.227308	-0.752556	H	0.359677	2.291763	1.802196
H	-1.798526	3.536823	-1.394814	H	1.937014	3.090969	1.849021
H	-0.78832	2.289072	-2.135863	H	1.794412	1.388654	2.317056
H	-2.411979	3.285194	1.109398	H	1.934155	3.730048	-0.658134

H	-2.455967	1.668274	1.83053	H	1.342315	2.557892	-1.844194
H	-0.902973	2.501666	1.628357	H	0.249131	3.228718	-0.635717
H	4.511447	1.663571	0.331924	H	-4.352617	1.555949	0.995788
H	-3.305457	-3.642571	1.104572	H	3.293616	-3.825502	0.226453
H	5.516065	-2.491924	-0.164092	H	-3.05262	-3.05834	-0.398565
H	6.202854	-0.154234	0.242464	H	-5.362944	-2.589792	0.431653
H	3.099591	-3.05896	-0.458132	H	-5.995381	-0.306242	1.125967

Supplementary Table 12 Calculated xyz coordinates. Atomic coordinates of *Z*-(*S*)-(*P*)-(S_a) and *Z*-(*S*)-(*M*)-(S_a) isomers of molecular motor **3**.

***Z*-(*S*)-(*P*)-(S_a)**				***Z*-(*S*)-(*M*)-(S_a)**			
C	-2.995984	1.070403	-0.067146	C	-2.974432	1.063865	-0.021609
C	-1.751289	0.515462	0.000865	C	-1.746931	0.483373	-0.154865
C	-4.272514	0.336608	-0.103979	C	-4.266133	0.357813	0.026196
C	-5.31314	1.208402	-0.470021	C	-5.264607	1.225355	0.506634
C	-4.77782	2.574228	-0.76603	C	-4.686925	2.565579	0.837704
C	-3.337439	2.578694	-0.158437	C	-3.275745	2.573215	0.165399
C	-4.558408	-0.983756	0.258024	C	-4.599751	-0.934127	-0.39573
C	-5.877734	-1.437908	0.216769	C	-5.923422	-1.369064	-0.281961
C	-6.910808	-0.570925	-0.173136	C	-6.910472	-0.51166	0.225702
C	-6.623136	0.752062	-0.508338	C	-6.576533	0.788623	0.608361
C	-2.40585	3.401936	-1.062059	C	-3.373029	3.240193	-1.227225
C	-3.393642	3.176184	1.267986	C	-2.297354	3.343402	1.067088
C	-0.460137	1.154389	0.36883	C	-0.429514	1.133292	-0.406986
C	0.674105	0.213405	0.176209	C	0.685697	0.214101	-0.055638
C	0.269539	-1.055326	-0.21968	C	0.247083	-1.066765	0.256134
S	-1.515123	-1.259634	-0.403507	S	-1.537373	-1.321825	0.058447
C	2.046147	0.485018	0.369116	C	2.062483	0.515945	-0.022921
C	2.958885	-0.557872	0.121318	C	2.949994	-0.523338	0.323111
C	2.526147	-1.843539	-0.285207	C	2.48323	-1.820261	0.642205
C	1.161419	-2.08475	-0.455876	C	1.111608	-2.0875	0.598034
O	-0.347823	2.274213	0.850357	O	-0.28234	2.24933	-0.881362
C	4.428286	-0.32329	0.294585	C	4.426375	-0.27271	0.336957
C	3.508453	-2.964566	-0.517737	C	3.437368	-2.921131	1.031056
C	2.514187	1.844608	0.823476	C	2.557213	1.907226	-0.33183
C	5.25839	-0.074788	-0.796789	C	5.15635	-0.199464	-0.848424
C	6.633866	0.134782	-0.633469	C	6.538736	0.026499	-0.841371
C	7.193288	0.092728	0.646792	C	7.208033	0.184752	0.375608
C	6.372438	-0.154968	1.747776	C	6.489303	0.110552	1.569333
C	5.019174	-0.356505	1.558271	C	5.127278	-0.115525	1.53314
F	4.230704	-0.588368	2.643053	F	4.444332	-0.195321	2.708851
O	7.334129	0.371058	-1.778076	O	7.133887	0.075782	-2.065781

C	8.740043	0.596697	-1.679461	C	8.541719	0.302387	-2.128096
O	-6.064043	-2.735326	0.588059	O	-6.159918	-2.640023	-0.713275
C	-7.388802	-3.267194	0.582752	C	-7.492197	-3.147681	-0.653324
O	-1.820338	-1.483676	-1.879474	O	-1.70862	-2.064549	-1.266667
H	-4.724658	2.736848	-1.848132	H	-5.304637	3.39639	0.486817
H	-5.393608	3.375862	-0.350408	H	-4.581038	2.680238	1.922124
H	-3.796614	-1.674539	0.589664	H	-3.884823	-1.602531	-0.856908
H	-7.93576	-0.914328	-0.204235	H	-7.938252	-0.83886	0.304465
H	-7.430946	1.417862	-0.790971	H	-7.35168	1.451193	0.977182
H	-1.428286	3.553679	-0.61258	H	-2.414136	3.186021	-1.741733
H	-2.859074	4.382837	-1.234292	H	-3.648897	4.291899	-1.108139
H	-2.27837	2.917507	-2.03393	H	-4.134299	2.756557	-1.845004
H	-3.691196	4.227039	1.208199	H	-2.743746	4.309892	1.319473
H	-4.12659	2.650421	1.885868	H	-2.119042	2.80566	2.002539
H	-2.417596	3.116359	1.747125	H	-1.344125	3.529811	0.580655
H	0.813989	-3.066305	-0.756752	H	0.742641	-3.080929	0.825777
H	4.149873	-3.11503	0.354525	H	3.910081	-2.701716	1.992613
H	2.985649	-3.898594	-0.725577	H	2.91415	-3.873467	1.120976
H	4.167189	-2.747979	-1.362786	H	4.23768	-3.033435	0.295788
H	2.016899	2.636806	0.264604	H	2.490138	2.11339	-1.403967
H	2.259487	2.004018	1.875258	H	1.939621	2.656609	0.162787
H	3.591581	1.949483	0.712274	H	3.592917	2.035925	-0.024207
H	4.845029	-0.03376	-1.797386	H	4.657021	-0.317191	-1.802552
H	8.251006	0.250251	0.803612	H	8.273547	0.362214	0.412776
H	6.78168	-0.189874	2.749791	H	6.984712	0.228774	2.525012
H	9.085002	0.760855	-2.698241	H	8.794018	0.303769	-3.186474
H	8.959717	1.481985	-1.07458	H	8.809151	1.268973	-1.689799
H	9.254064	-0.272748	-1.257703	H	9.095905	-0.495361	-1.6237
H	-7.292077	-4.300496	0.909496	H	-7.438965	-4.161354	-1.045143
H	-8.038607	-2.726229	1.277673	H	-7.862194	-3.176305	0.376363
H	-7.823522	-3.243942	-0.421227	H	-8.173389	-2.555069	-1.271951

Supplementary Table 13 Calculated xyz coordinates. Atomic coordinates of *Z*-(*S*)-(*P*)-(R_a) and *Z*-(*S*)-(*M*)-(R_a) isomers of molecular motor **3**.

***Z*-(*S*)-(*P*)-(R_a)**				***Z*-(*S*)-(*M*)-(R_a)**			
C	-2.998207	1.052254	-0.054589	C	2.931221	1.087716	0.12524
C	-1.745993	0.511104	-0.055386	C	1.741699	0.435643	0.273611
C	-4.264224	0.312438	0.085074	C	4.232649	0.459606	-0.158454
C	-5.343567	1.146029	-0.257115	C	5.15221	1.439092	-0.577442
C	-4.852205	2.483674	-0.714762	C	4.503551	2.786798	-0.627363
C	-3.364629	2.546051	-0.238385	C	3.166436	2.619679	0.16516
C	-4.500861	-0.973002	0.582548	C	4.645391	-0.866642	0.012532
C	-5.813255	-1.433568	0.70159	C	5.965075	-1.215071	-0.289761
C	-6.887334	-0.606784	0.336034	C	6.870773	-0.241865	-0.736692
C	-6.646284	0.683531	-0.136004	C	6.460974	1.086092	-0.868298
C	-2.524977	3.297552	-1.28334	C	3.3817	3.054995	1.634112
C	-3.303731	3.258165	1.134196	C	2.080785	3.480144	-0.50151
C	-0.435005	1.184309	0.141656	C	0.437445	0.974301	0.753181
C	0.688983	0.239063	-0.085494	C	-0.679746	0.074703	0.358456
C	0.26664	-1.058284	-0.345819	C	-0.236339	-1.110122	-0.215838
S	-1.524276	-1.290132	-0.335006	S	1.565785	-1.313985	-0.230925
C	2.069529	0.535469	-0.04575	C	-2.061729	0.310732	0.510806
C	2.969663	-0.519085	-0.290708	C	-2.94829	-0.677827	0.037275
C	2.516989	-1.829736	-0.581754	C	-2.476328	-1.88375	-0.532953
C	1.146601	-2.09357	-0.60207	C	-1.099582	-2.095859	-0.650979
O	-0.294154	2.337792	0.52664	O	0.306983	1.995265	1.411227
C	4.445983	-0.276074	-0.222214	C	-4.427481	-0.454305	0.108976
C	3.489322	-2.946367	-0.866829	C	-3.430814	-2.948103	-1.012108
C	2.552276	1.939343	0.223548	C	-2.560923	1.574506	1.166303
C	5.102458	-0.163937	1.002463	C	-5.073225	0.388926	-0.794135
C	6.484243	0.05433	1.072747	C	-6.457233	0.59663	-0.737848
C	7.228234	0.165027	-0.105483	C	-7.214658	-0.050318	0.242812
C	6.583556	0.051145	-1.337738	C	-6.580962	-0.899786	1.150413
C	5.220192	-0.166583	-1.377771	C	-5.21494	-1.088334	1.070552
F	4.610544	-0.286252	-2.589298	F	-4.616404	-1.929853	1.957666
O	7.003235	0.144553	2.329279	O	-6.964707	1.442917	-1.677362

C	8.4061	0.366869	2.47003	C	-8.368404	1.701766	-1.670959
O	-5.949676	-2.694886	1.198002	O	6.282525	-2.526152	-0.096084
C	-7.262929	-3.231455	1.356886	C	7.621465	-2.951865	-0.345939
O	-1.969111	-1.639167	-1.749377	O	1.887141	-2.265689	0.921167
H	-4.894673	2.552432	-1.807351	H	5.127142	3.578054	-0.203031
H	-5.438697	3.315794	-0.317093	H	4.286831	3.070000	-1.663332
H	-3.704259	-1.630405	0.90024	H	3.99898	-1.633123	0.41923
H	-7.907062	-0.954733	0.429012	H	7.89577	-0.503569	-0.961214
H	-7.484001	1.319696	-0.399109	H	7.175924	1.834653	-1.19138
H	-1.514697	3.492133	-0.933555	H	2.481202	2.872682	2.219663
H	-3.004381	4.257952	-1.495622	H	3.611748	4.123806	1.666687
H	-2.474089	2.735309	-2.219698	H	4.215415	2.513368	2.088668
H	-3.61773	4.299218	1.015787	H	2.470349	4.494541	-0.629264
H	-3.973444	2.781396	1.855011	H	1.828341	3.09423	-1.493179
H	-2.28882	3.242095	1.528524	H	1.174508	3.539823	0.094328
H	0.78633	-3.094884	-0.807594	H	-0.725402	-3.01717	-1.082154
H	4.241543	-3.03209	-0.078969	H	-4.175488	-2.537149	-1.698039
H	2.968956	-3.900524	-0.95411	H	-2.894376	-3.748504	-1.522432
H	4.022285	-2.764235	-1.804294	H	-3.973786	-3.387041	-0.170355
H	2.007549	2.658458	-0.388557	H	-2.046959	1.742631	2.112524
H	2.366667	2.221751	1.263464	H	-2.348207	2.447681	0.542399
H	3.617697	2.035928	0.026253	H	-3.633621	1.533625	1.341236
H	4.544641	-0.243891	1.927681	H	-4.504755	0.902284	-1.560225
H	8.295305	0.335689	-0.083428	H	-8.283844	0.091695	0.312781
H	7.137621	0.13203	-2.264663	H	-7.145487	-1.413511	1.918608
H	8.59288	0.406608	3.541194	H	-8.545108	2.392195	-2.493131
H	8.984701	-0.451689	2.030545	H	-8.943135	0.785207	-1.836663
H	8.706481	1.315174	2.0136	H	-8.682804	2.167491	-0.731769
H	-7.124829	-4.23248	1.760252	H	7.638757	-4.016223	-0.120752
H	-7.854738	-2.635962	2.058934	H	7.900237	-2.798746	-1.393202
H	-7.785478	-3.296159	0.397639	H	8.332989	-2.432114	0.303341

Supplementary Table 14 Calculated xyz coordinates. Atomic coordinates of *E*-(*S*)-(*P*)-(S_a) and *E*-(*S*)-(*M*)-(S_a) isomers of molecular motor **3**.

***E*-(*S*)-(*P*)-(S_a)**				***E*-(*S*)-(*M*)-(S_a)**			
C	3.273956	-0.787435	-0.076157	C	-3.279837	-0.789544	-0.249147
C	1.929069	-1.004299	-0.128877	C	-1.947065	-1.006611	-0.441745
C	3.978411	0.509882	-0.148908	C	-3.991252	0.503643	-0.139674
C	5.348109	0.282426	-0.385954	C	-5.375699	0.272053	-0.019013
C	5.620209	-1.182037	-0.533534	C	-5.697192	-1.182592	-0.130132
C	4.373396	-1.864376	0.107808	C	-4.329138	-1.908672	0.016855
C	3.517827	1.807511	0.0836	C	-3.505393	1.813457	-0.054575
C	4.419812	2.873038	0.049816	C	-4.393769	2.871349	0.144519
C	5.778367	2.645786	-0.216001	C	-5.77177	2.633464	0.253914
C	6.23759	1.345248	-0.428398	C	-6.25464	1.328784	0.171732
C	4.12848	-3.22443	-0.564909	C	-4.17532	-2.406244	1.474412
C	4.62953	-2.05886	1.624729	C	-4.291078	-3.094202	-0.965629
C	0.855472	-0.066302	-0.580577	C	-0.855247	-0.029119	-0.745128
C	-0.491254	-0.635384	-0.319918	C	0.456654	-0.563144	-0.289372
C	-0.446307	-1.929213	0.180245	C	0.387509	-1.899239	0.085635
S	1.209702	-2.641022	0.274511	S	-1.213885	-2.677959	-0.254573
C	-1.736639	0.004346	-0.509501	C	1.677451	0.134236	-0.195317
C	-2.893942	-0.698757	-0.125791	C	2.798349	-0.576862	0.281651
C	-2.824381	-2.015647	0.393397	C	2.706112	-1.935085	0.665978
C	-1.581168	-2.633202	0.537898	C	1.481031	-2.60021	0.552499
O	1.055693	0.998335	-1.1445	O	-1.005747	1.042183	-1.308161
C	-4.239307	-0.050221	-0.240147	C	4.130043	0.102155	0.371413
C	-4.072301	-2.767388	0.782287	C	3.904181	-2.68133	1.196201
C	-1.809584	1.389996	-1.101487	C	1.770421	1.591883	-0.572674
C	-4.664988	0.896842	0.690047	C	4.899294	0.334017	-0.76777
C	-5.925381	1.5002	0.592868	C	6.148811	0.962189	-0.689025
C	-6.780231	1.152258	-0.456953	C	6.641281	1.370322	0.553899
C	-6.367618	0.202584	-1.392325	C	5.882262	1.141449	1.702164
C	-5.120437	-0.378335	-1.270906	C	4.65466	0.517664	1.595821
F	-4.74011	-1.312252	-2.18635	F	3.932768	0.293312	2.728832
O	-6.220321	2.40836	1.564624	O	6.800176	1.12848	-1.873842

C	-7.488908	3.061074	1.521627	C	8.077964	1.764428	-1.863244
O	3.881358	4.103319	0.292778	O	-3.821514	4.107947	0.218758
C	4.742962	5.24006	0.28545	C	-4.665076	5.241442	0.415601
O	1.485487	-3.01592	1.726548	O	-1.066246	-3.34853	-1.617773
H	5.690266	-1.458552	-1.591918	H	-6.416106	-1.520103	0.621028
H	6.547144	-1.502472	-0.05148	H	-6.13534	-1.400819	-1.110403
H	2.480794	2.024184	0.281985	H	-2.459411	2.047022	-0.157696
H	6.480836	3.467514	-0.245769	H	-6.465912	3.449045	0.40399
H	7.292406	1.174658	-0.613048	H	-7.319678	1.146388	0.262834
H	3.428627	-3.852119	-0.014156	H	-3.206701	-2.883198	1.626586
H	5.077753	-3.766883	-0.599918	H	-4.955812	-3.139494	1.695119
H	3.769241	-3.109882	-1.590517	H	-4.270693	-1.581146	2.184901
H	5.439052	-2.781087	1.764592	H	-5.19397	-3.694376	-0.819534
H	4.925982	-1.118868	2.096638	H	-4.280107	-2.745838	-2.001144
H	3.731508	-2.432131	2.117139	H	-3.43709	-3.754139	-0.82596
H	-1.513839	-3.641987	0.928206	H	1.400971	-3.645979	0.826077
H	-4.686962	-2.978811	-0.097221	H	4.20834	-2.283512	2.168516
H	-3.820856	-3.716797	1.255774	H	3.676957	-3.740521	1.320155
H	-4.687671	-2.187392	1.474294	H	4.761654	-2.58694	0.525754
H	-1.233692	1.442415	-2.025417	H	0.903507	2.143059	-0.209927
H	-2.838091	1.681907	-1.301531	H	2.676665	2.045376	-0.176079
H	-1.367438	2.126824	-0.42474	H	1.778139	1.711004	-1.660266
H	-4.017083	1.183007	1.50974	H	4.535464	0.027871	-1.741123
H	-7.757511	1.602393	-0.559513	H	7.600805	1.859201	0.645947
H	-7.011899	-0.0853	-2.213768	H	6.242159	1.44716	2.676723
H	-7.505433	3.731132	2.378715	H	8.404552	1.788175	-2.900832
H	-7.605481	3.644009	0.602673	H	8.010117	2.787519	-1.480414
H	-8.309702	2.342054	1.606905	H	8.800138	1.196884	-1.268062
H	4.105428	6.095025	0.501733	H	-4.000616	6.102857	0.440354
H	5.516123	5.160492	1.05629	H	-5.377174	5.357385	-0.407557
H	5.214071	5.378351	-0.692882	H	-5.208239	5.175919	1.363743

Supplementary Table 15 Calculated xyz coordinates. Atomic coordinates of *E*-(*S*)-(*P*)-(R_a) and *E*-(*S*)-(*M*)-(R_a) isomers of molecular motor **3**.

***E*-(*S*)-(*P*)-(R_a)**				***E*-(*S*)-(*M*)-(R_a)**			
C	3.25589	-0.780771	-0.348827	C	-3.290087	-0.774849	0.023516
C	1.905155	-0.958329	-0.308208	C	-1.988078	-1.056464	-0.269044
C	3.996424	0.493023	-0.234228	C	-3.968781	0.540452	0.006872
C	5.327721	0.295939	-0.649367	C	-5.337183	0.36924	0.294738
C	5.531613	-1.113921	-1.109397	C	-5.687297	-1.076119	0.436311
C	4.328111	-1.890572	-0.492592	C	-4.320961	-1.809	0.563982
C	3.60634	1.724925	0.294838	C	-3.458314	1.833731	-0.152377
C	4.537751	2.761874	0.379800	C	-4.307399	2.934624	-0.029221
C	5.855217	2.571379	-0.063173	C	-5.670917	2.755372	0.246594
C	6.24579	1.332251	-0.572538	C	-6.177897	1.467332	0.408461
C	3.970934	-3.084164	-1.392476	C	-4.026349	-2.073239	2.060404
C	4.721407	-2.388526	0.922186	C	-4.398408	-3.136683	-0.212665
C	0.829576	0.070085	-0.459223	C	-0.921274	-0.166057	-0.825525
C	-0.506007	-0.511996	-0.171853	C	0.423936	-0.662515	-0.426628
C	-0.464091	-1.881684	0.049556	C	0.376406	-1.926795	0.149121
S	1.164174	-2.629184	-0.171566	S	-1.261755	-2.701923	0.090526
C	-1.73695	0.176881	-0.096361	C	1.657086	0.004927	-0.559403
C	-2.884498	-0.574165	0.221236	C	2.810271	-0.655082	-0.085643
C	-2.813989	-1.966724	0.47365	C	2.743954	-1.947507	0.483827
C	-1.585339	-2.621821	0.375164	C	1.503949	-2.58666	0.593537
O	1.013383	1.216549	-0.83791	O	-1.11654	0.812804	-1.526128
C	-4.224464	0.092634	0.275932	C	4.145669	0.016062	-0.183508
C	-4.044499	-2.756317	0.842421	C	3.984239	-2.661069	0.961737
C	-1.80513	1.667084	-0.321014	C	1.742522	1.372851	-1.187686
C	-4.909696	0.429494	-0.890475	C	4.738396	0.620207	0.9238
C	-6.16664	1.045956	-0.846722	C	5.991062	1.240487	0.832809
C	-6.753119	1.334824	0.388777	C	6.668317	1.256168	-0.389837
C	-6.079237	0.999719	1.563756	C	6.085056	0.655675	-1.506209
C	-4.841899	0.390213	1.491305	C	4.847971	0.052443	-1.388858
F	-4.204677	0.061012	2.648713	F	4.289349	-0.520271	-2.489156
O	-6.729857	1.322255	-2.056204	O	6.458726	1.796054	1.985844

C	-8.011495	1.950065	-2.080933	C	7.728677	2.447151	1.959574
O	4.069429	3.926909	0.914421	O	-3.713087	4.151633	-0.196187
C	4.965808	5.028984	1.043033	C	-4.516307	5.325727	-0.088581
O	1.562463	-3.299325	1.138489	O	-1.263708	-3.568595	-1.165533
H	5.489883	-1.174556	-2.203064	H	-6.335124	-1.2776	1.293461
H	6.488353	-1.539148	-0.796146	H	-6.219733	-1.427103	-0.454686
H	2.601925	1.912857	0.637972	H	-2.424615	2.019434	-0.389359
H	6.579905	3.371872	-0.002763	H	-6.334987	3.603982	0.338276
H	7.271154	1.185911	-0.893578	H	-7.230928	1.331017	0.628515
H	3.302666	-3.796451	-0.909638	H	-3.052845	-2.547013	2.190708
H	4.891769	-3.625801	-1.627665	H	-4.788863	-2.740254	2.471699
H	3.522019	-2.760412	-2.334663	H	-4.039092	-1.143809	2.635432
H	5.51531	-3.135819	0.834396	H	-5.294813	-3.674467	0.109971
H	5.093888	-1.56643	1.538267	H	-4.478473	-2.959871	-1.287749
H	3.862709	-2.839733	1.419407	H	-3.548553	-3.794967	-0.042802
H	-1.51946	-3.689441	0.549438	H	1.439785	-3.583161	1.015280
H	-4.851521	-2.597589	0.123101	H	4.423196	-2.160156	1.828760
H	-3.82246	-3.822977	0.884099	H	3.752561	-3.688191	1.245095
H	-4.420198	-2.449915	1.822722	H	4.752231	-2.682338	0.184567
H	-1.663952	1.907808	-1.378565	H	0.898785	1.995223	-0.894431
H	-2.764119	2.071288	-0.003786	H	2.670916	1.873486	-0.918166
H	-1.004801	2.175589	0.216397	H	1.70547	1.292192	-2.278636
H	-4.472371	0.216985	-1.858545	H	4.229509	0.623399	1.880230
H	-7.720759	1.811866	0.454463	H	7.636068	1.726771	-0.490683
H	-6.51262	1.211915	2.533231	H	6.588407	0.656981	-2.465024
H	-8.260019	2.072704	-3.133102	H	7.894941	2.811014	2.971430
H	-8.770075	1.325328	-1.599024	H	8.527928	1.75025	1.688535
H	-7.983303	2.931677	-1.597718	H	7.728133	3.293023	1.265080
H	4.383327	5.83268	1.489085	H	-3.840576	6.161861	-0.257175
H	5.807948	4.785026	1.698457	H	-5.306145	5.340409	-0.846186
H	5.342651	5.353464	0.067891	H	-4.961912	5.415462	0.907285

Supplementary Note 8: Crystal Structural Data

Supplementary Table 16 Crystal structural data. Data acquired for **2**-*Z*-**I** and **2**-*E*-**II**.

**Compound**	**2-*Z*-I** (CCDC 2154099, xv772)	**2-*E*-II** (CCDC 2154098, xv666)#
Net Formula	$C_{39}H_{42}Cl_2FN_3O_7S$	$C_{38}H_{40}FN_3O_7S$
M_r/g mol^{-1}	786.71	701.79
Crystal Size/mm	0.100 × 0.070 × 0.050	0.100 × 0.030 × 0.010
T/K	102.(2)	102.(2)
Radiation	MoKα	MoKα
Diffractometer	'Bruker D8 Venture TXS'	'Bruker D8 Venture TXS'
Crystal system	triclinic	triclinic
Space group	'P -1'	'P -1'
a/Å	11.2427(4)	10.2939(14)
b/Å	13.2813(5)	12.5509(16)
c/Å	15.0661(6)	14.2604(18)
α/°	104.7280(10)	94.002(4)
β/°	101.4800(10)	107.559(4)
γ/°	114.0950(10)	99.947(5)
V/Å^3	1865.67(12)	1715.5(4)
Z	2	2
Calc. Density/g cm^{-3}	1.4	1.359
μ/mm^{-1}	0.289	0.155
Absorption Correction	Multi-Scan	Multi-Scan
Transmission Factor Range	0.95–0.99	0.84–1.00
refls. measured	29982	5808
R_{int}	0.0327	0.1046
Mean $\sigma(I)/I$	0.0307	0.1062
θ Range	2.528–26.371	2.412–25.347
Observed refls.	6522	4311
x, y (weighting scheme)	0.0710, 2.2929	0, 4.9472
Hydrogen Refinement	constr	constr
Refls in Refinement	7608	5808
Parameters	492	456
Restraints	0	1
$R(F_{obs})$	0.0527	0.0854
$R_w(F^2)$	0.1512	0.1692
S	1.066	1.125
Shift/Error$_{max}$	0.001	0.001
Max Electron Density/e Å^{-3}	0.842	0.529
Min Electron Density/e Å^{-3}	−0.764	−0.485

#all reflexes obtained from the data-reduction have been used with the exception of two, which were blocked by the beamstop. The Completeness was determined using the command TWST 0. The Rint was taken from the TWINABS output.

Supplementary Table 17 Crystal structural data. Data acquired for **2**-*Z*-**II** and **4**-*Z*.

**Compound**	**2-*Z*-II (CCDC 2154100, xv804)**	**4-*Z* (CCDC 2154101, yv042)**
Net Formula	$C_{38}H_{40}FN_3O_7S$	$C_{39}H_{42}Cl_2FN_3O_6S$
M_r/g mol^{-1}	701.79	770.71
Crystal Size/mm	0.100 × 0.020 × 0.020	0.080 × 0.050 × 0.020
T/K	102.(2)	102.(2)
Radiation	MoKα	MoKα
Diffractometer	'Bruker D8 Venture TXS'	'Bruker D8 Venture TXS'
Crystal system	monoclinic	triclinic
Space group	'P 1 21/n 1'	'P -1'
a/Å	17.0034(8)	11.1182(10)
b/Å	9.0297(4)	13.0839(10)
c/Å	23.6766(13)	15.0986(12)
α/°	90	105.030(3)
β/°	109.863(2)	103.046(3)
γ/°	90	111.473(3)
V/Å^3	3418.9(3)	1844.1(3)
Z	4	2
Calc. Density/g cm^{-3}	1.363	1.388
μ/mm^{-1}	0.156	0.289
Absorption Correction	Multi-Scan	Multi-Scan
Transmission Factor Range	0.91–1.00	0.91–0.99
refls. measured	54144	20287
R_{int}	0.1179	0.0558
Mean $\sigma(I)/I$	0.0635	0.0643
θ Range	2.599–25.349	2.591–25.350
Observed refls.	3976	4540
x, y (weighting scheme)	0.1006, 16.7733	0.0710, 1.5772
Hydrogen Refinement	constr	constr
Refls in Refinement	6241	6678
Parameters	417	482
Restraints	120	0
$R(F_{obs})$	0.1016	0.0562
$R_w(F^2)$	0.2711	0.1573
S	1.029	1.032
Shift/Error$_{max}$	0.001	0.001
Max Electron Density/e Å^{-3}	1.382	0.495
Min Electron Density/e Å^{-3}	−1.421	−0.520

Supplementary Table 18 Crystal structural data. Data acquired for **3**-*E*-(*R*)-(*R*a).

**Compound**	**3-*E*-(*R*)-(*R*a) (CCDC 2163097, 22dub_brp01)**
Diffractometer	SuperNova, Atlas
Formula	$C_{29}H_{27}FO_4S$
$D_{calc.}$/ g cm^{-3}	1.305
m/mm^{-1}	1.491
Formula Weight	490.56
Colour	clear light yellow
Shape	block-shaped
Size/mm^3	0.25×0.18×0.14
T/K	154(2)
Crystal System	orthorhombic
Flack Parameter	–0.016(11)
Hooft Parameter	–0.015(10)
Space Group	$P2_12_12_1$
a/Å	12.7504(3)
b/Å	13.2036(3)
c/Å	14.8359(3)
a/°	90
b/°	90
g/°	90
V/Å^3	2497.64(10)
Z	4
Z'	1
Wavelength/Å	1.54184
Radiation type	Cu K_a
θ Range	4.483–72.257
Measured Refl's.	8119
Indep't Refl's	4745
Refl's I≥2 *s*(I)	4451
R_{int}	0.0302
Parameters	322
Restraints	0
Largest Peak	0.14
Deepest Hole	–0.253
GooF	1.036
wR_2 (all data)	0.085
wR_2	0.0821
R_1 (all data)	0.0362
R_1	0.033

Supplementary Note 9: NMR Spectra of Synthesized Compounds

a)

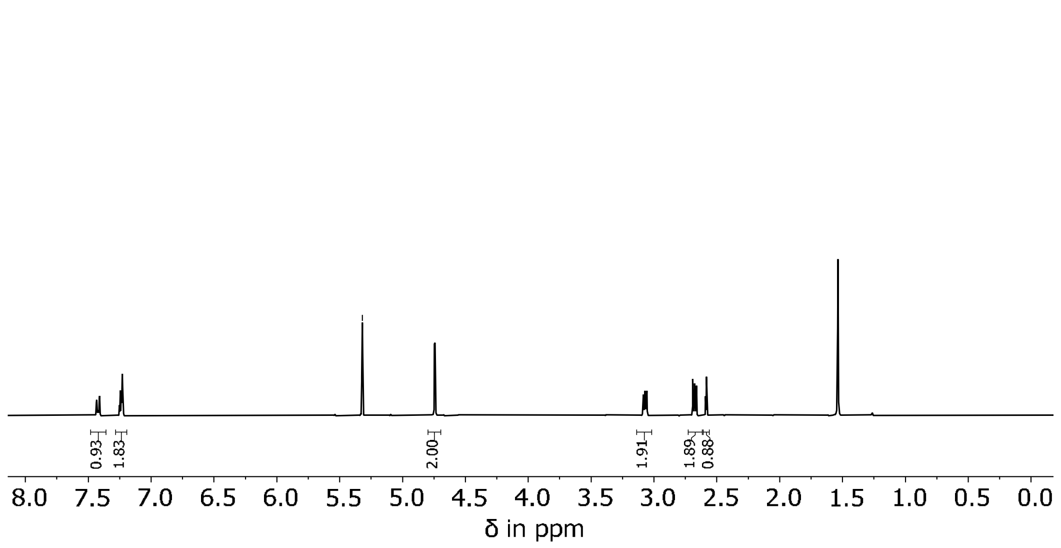

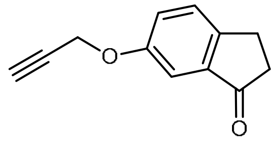

7

b)

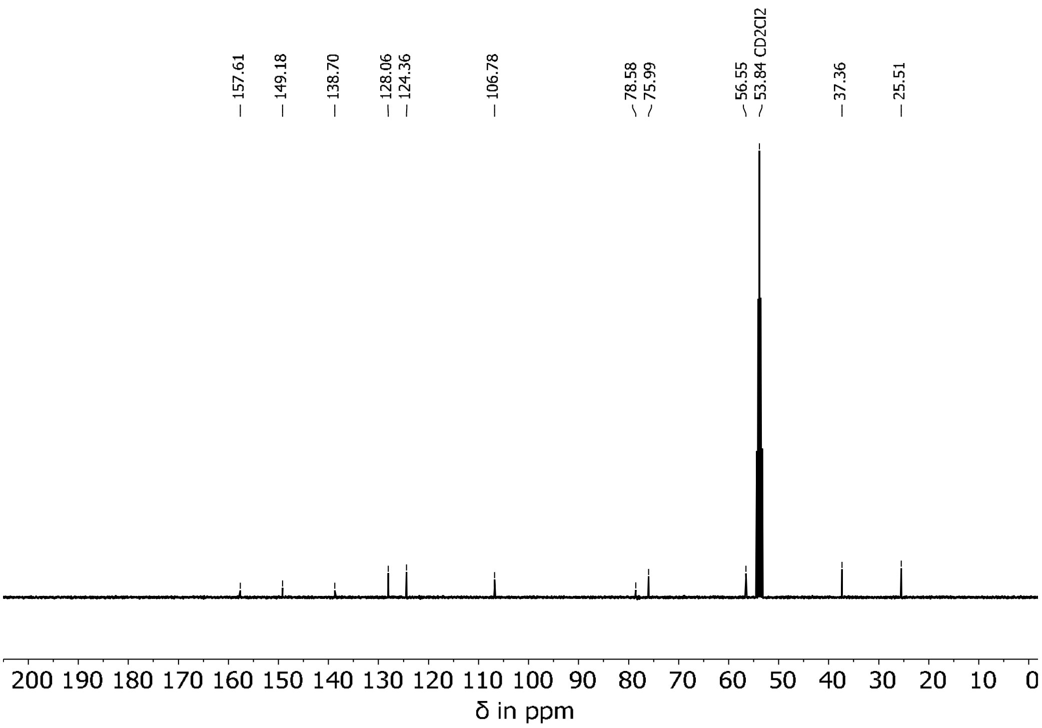

Supplementary Figure 94 NMR spectra of compound 7 in CD_2Cl_2 at 25 °C. a) ^{1}H NMR spectrum measured at 400 MHz and b) ^{13}C NMR spectrum measured at 101 MHz.

a)

b)

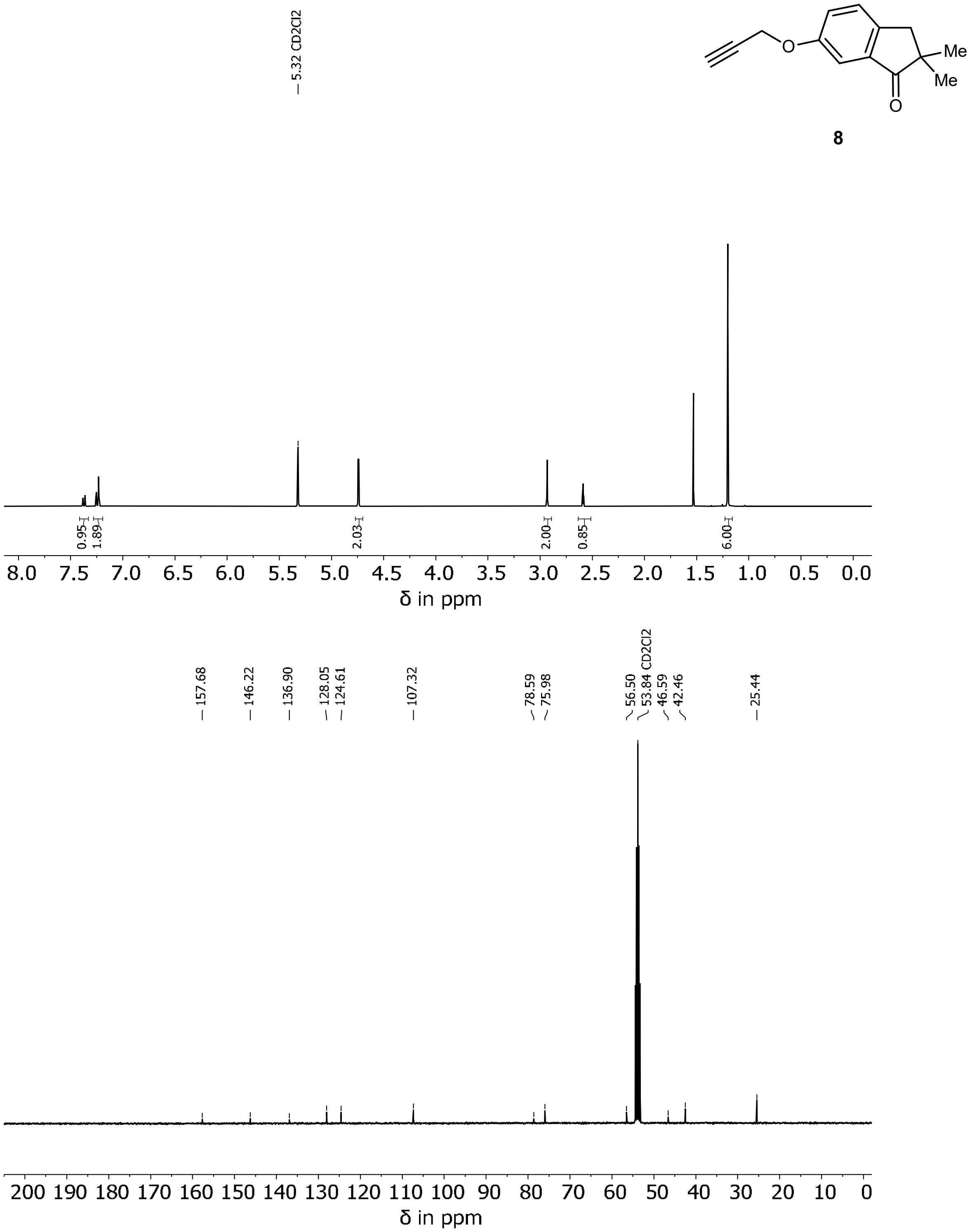

Supplementary Figure 95 NMR spectra of compound 8 in CD_2Cl_2 at 25 °C. a) ^1H NMR spectrum measured at 400 MHz and b) ^{13}C NMR spectrum measured at 101 MHz.

a)

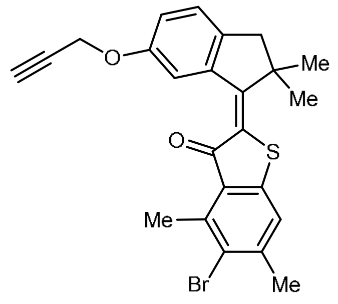

15

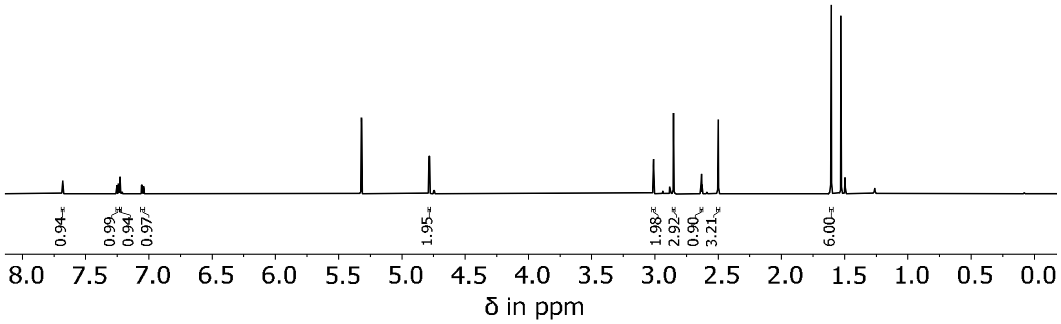

b)

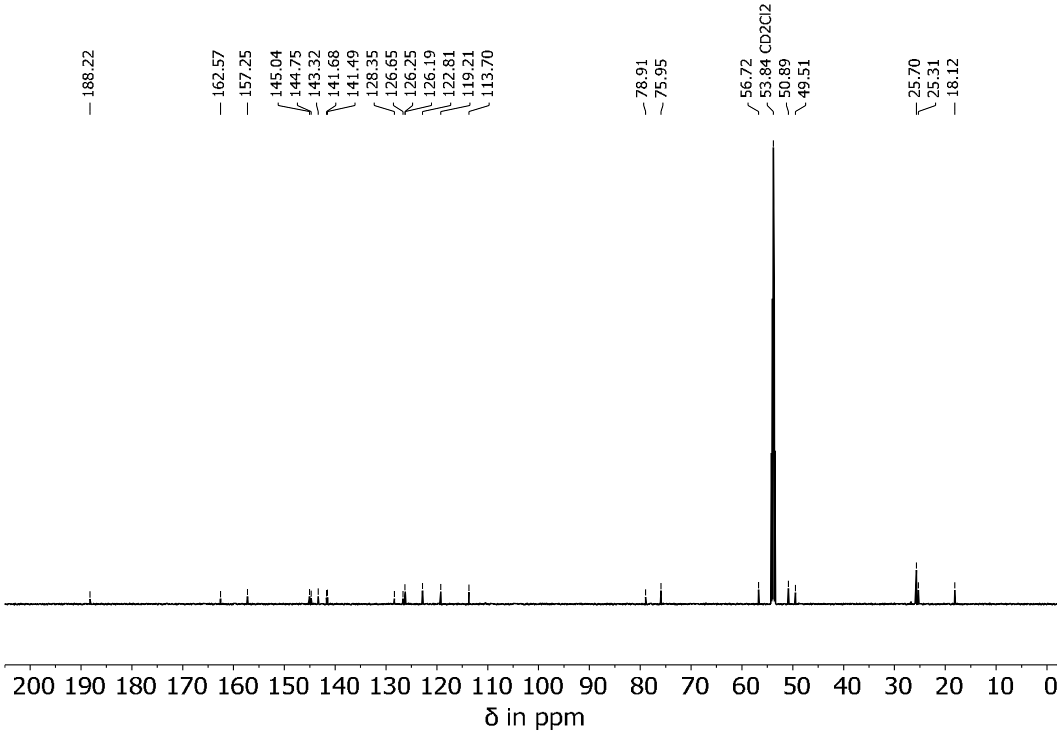

Supplementary Figure 96 NMR spectra of compound 15 in CD_2Cl_2 at 25 °C. a) ^{1}H NMR spectrum measured at 400 MHz and b) ^{13}C NMR spectrum measured at 101 MHz.

a)

b)

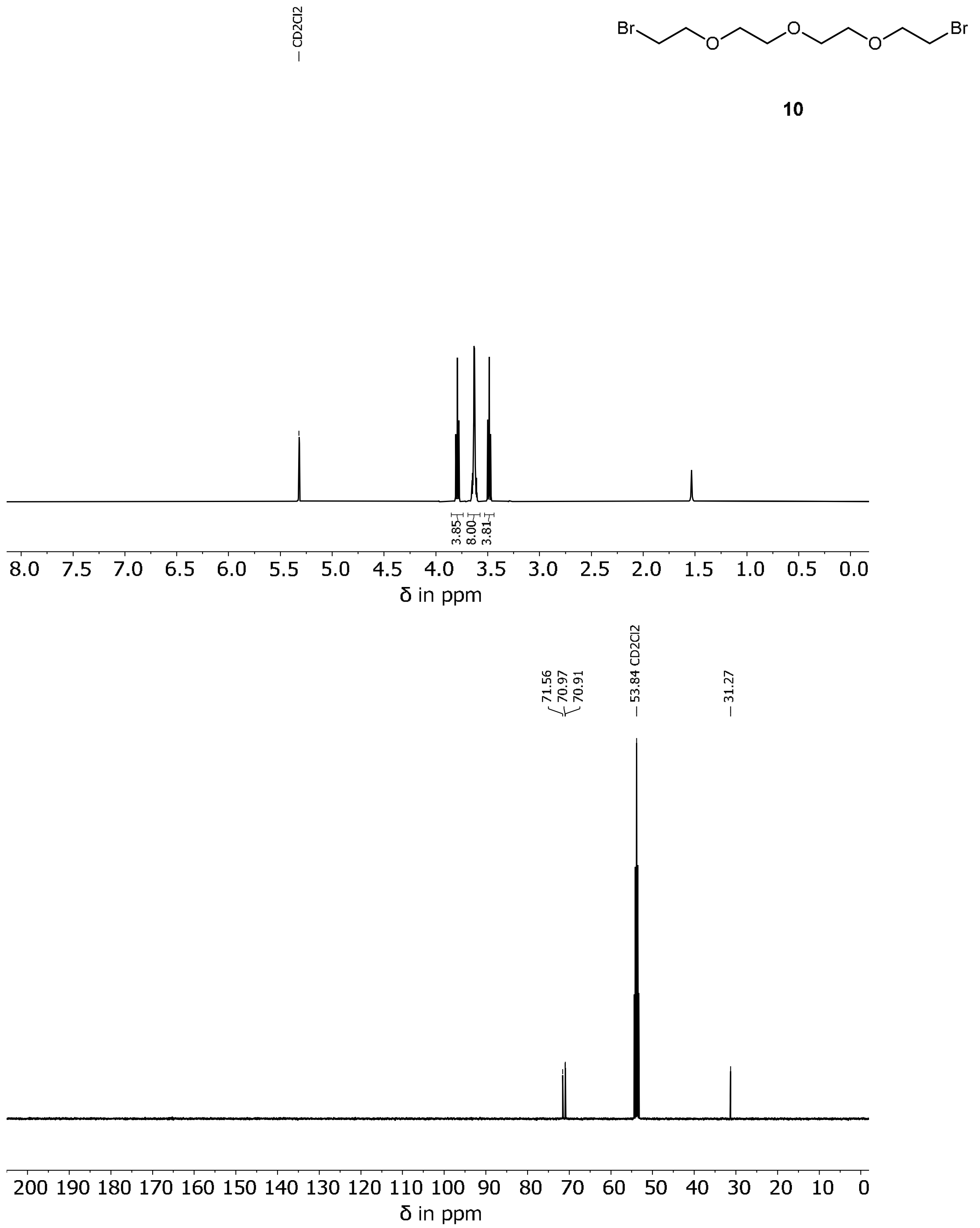

Supplementary Figure 97 NMR spectra of compound 10 in CD_2Cl_2 at 25 °C. a) ^{1}H NMR spectrum measured at 400 MHz and b) ^{13}C NMR spectrum measured at 101 MHz.

a)

b)

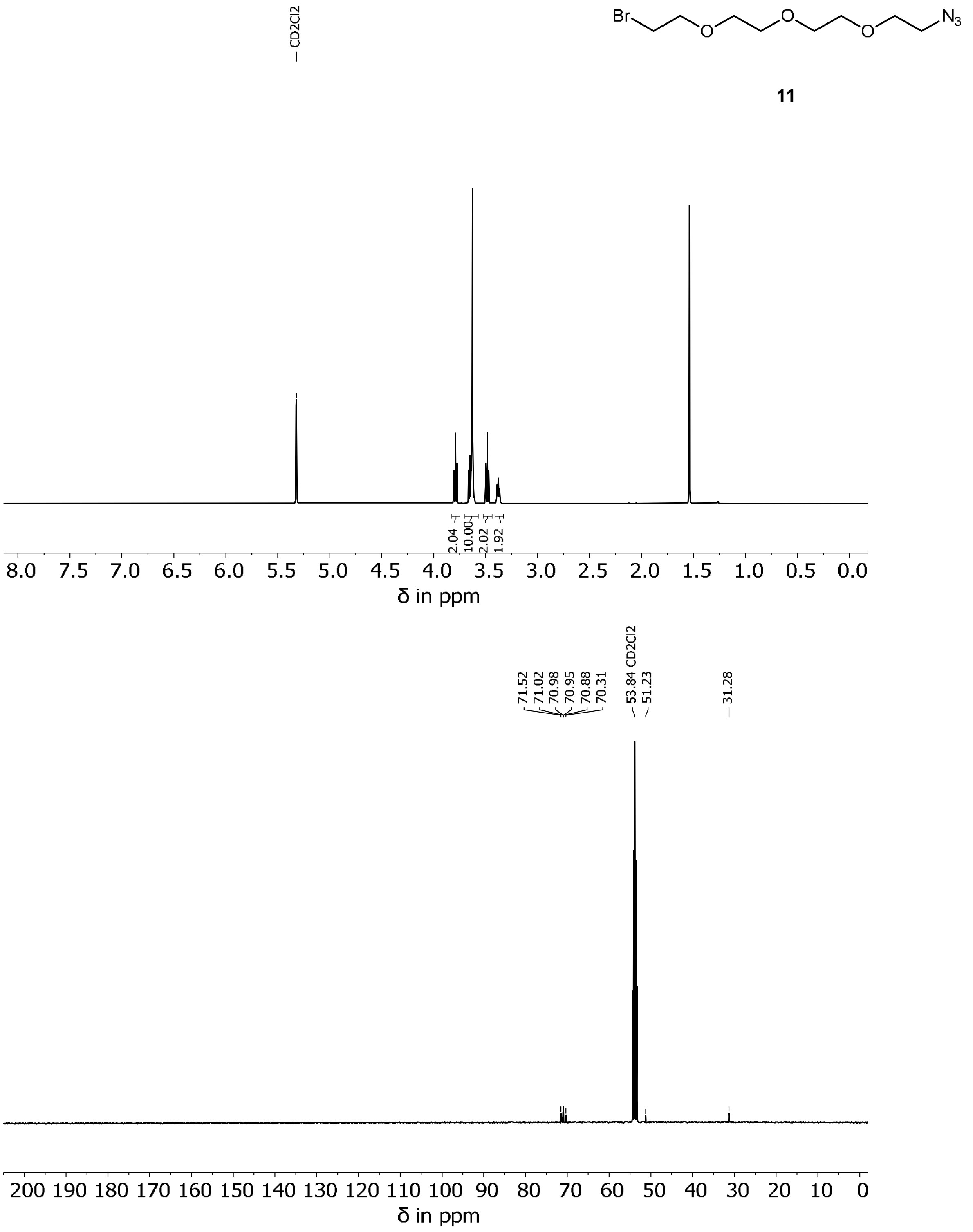

Supplementary Figure 98 NMR spectra of compound 11 in CD_2Cl_2 at 25 °C. a) ^{1}H NMR spectrum measured at 400 MHz and b) ^{13}C NMR spectrum measured at 101 MHz.

a)

b)

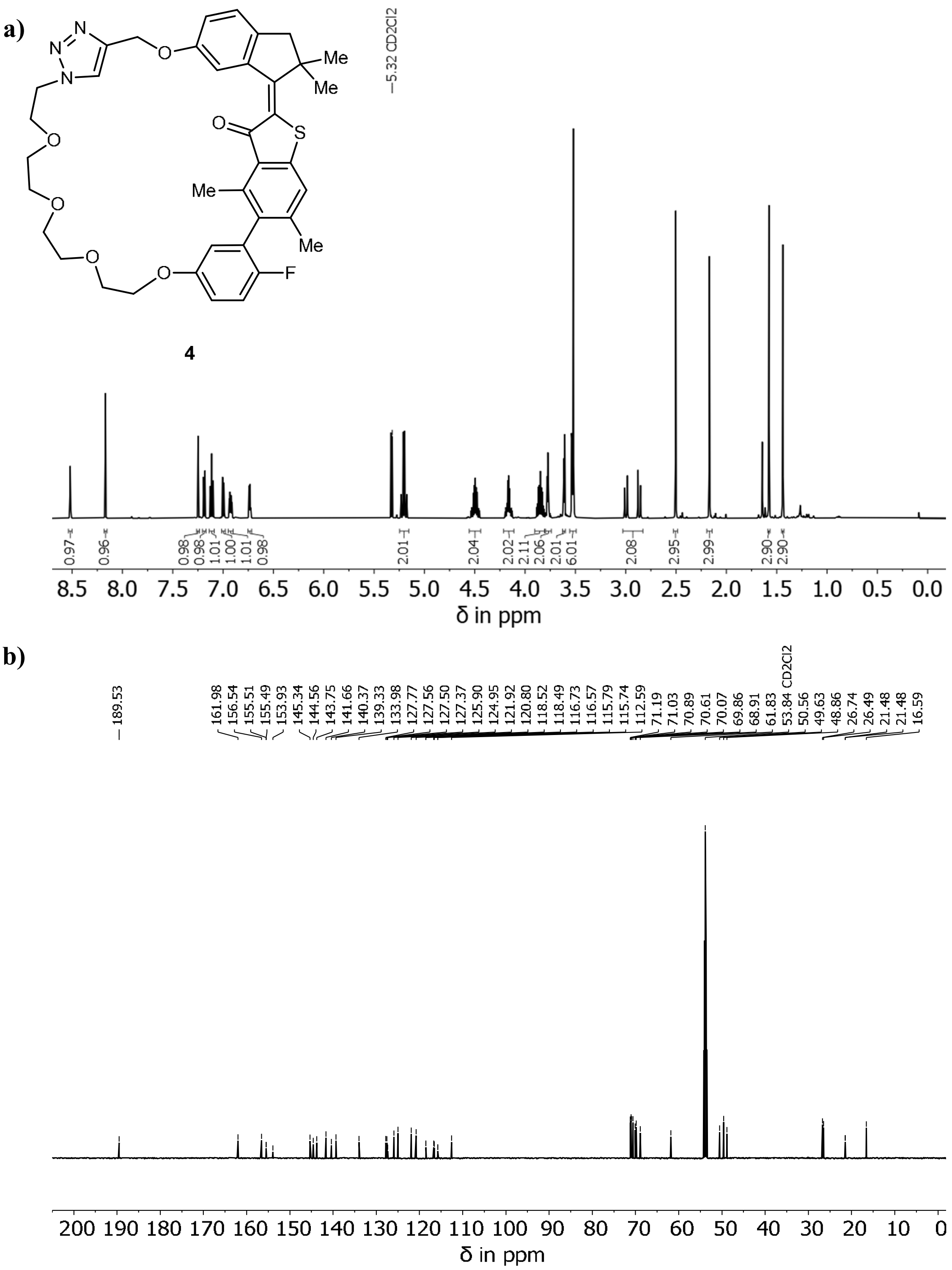

Supplementary Figure 99 NMR spectra of compound 4 in CD_2Cl_2 at 25 °C. a) ^{1}H NMR spectrum measured at 600 MHz and b) ^{13}C NMR spectrum measured at 151 MHz.

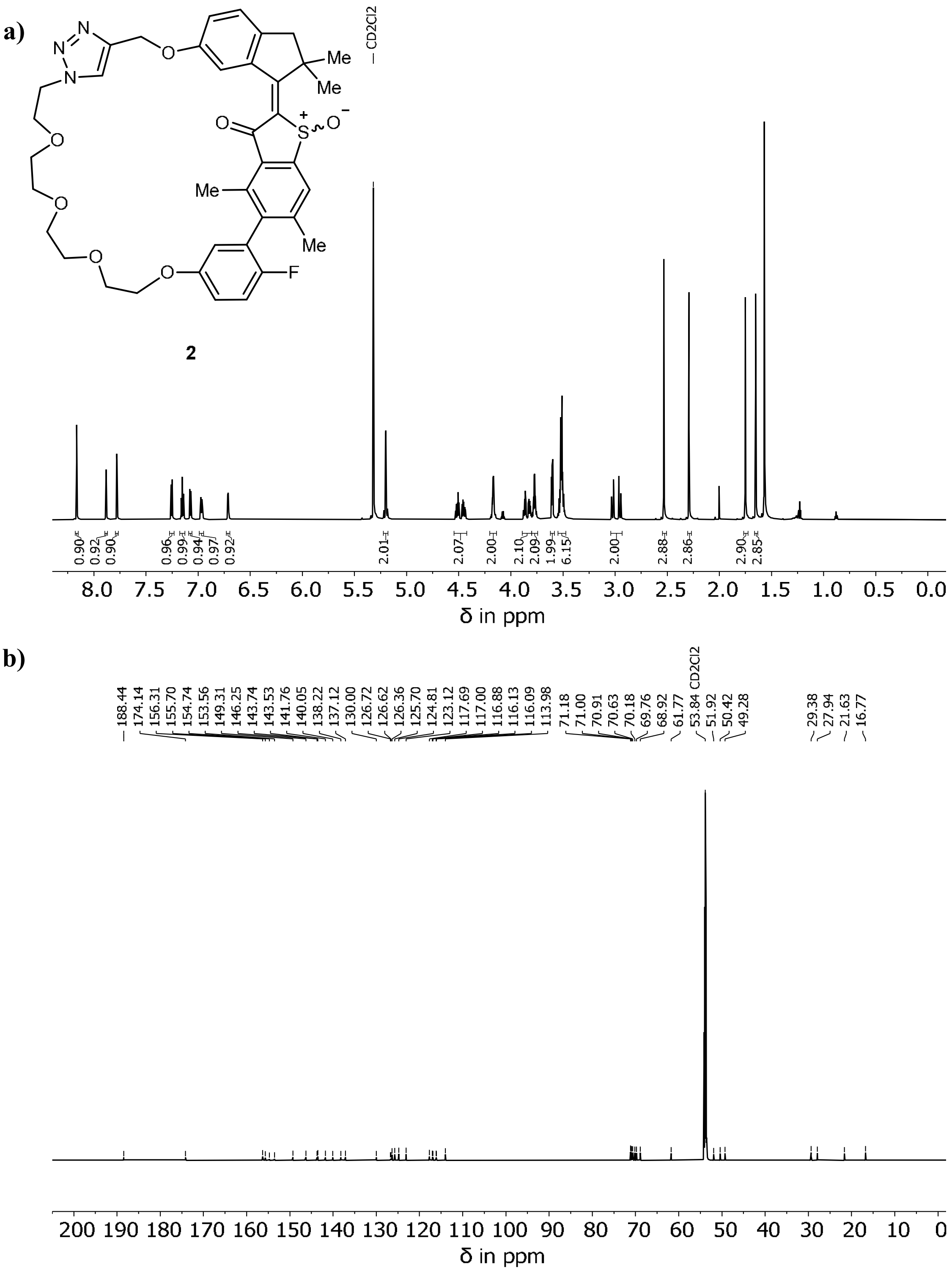

Supplementary Figure 100 NMR spectra of compound 2 in CD_2Cl_2 at 25 °C. a) ^{1}H NMR spectrum measured at 800 MHz and b) ^{13}C NMR spectrum measured at 201 MHz.

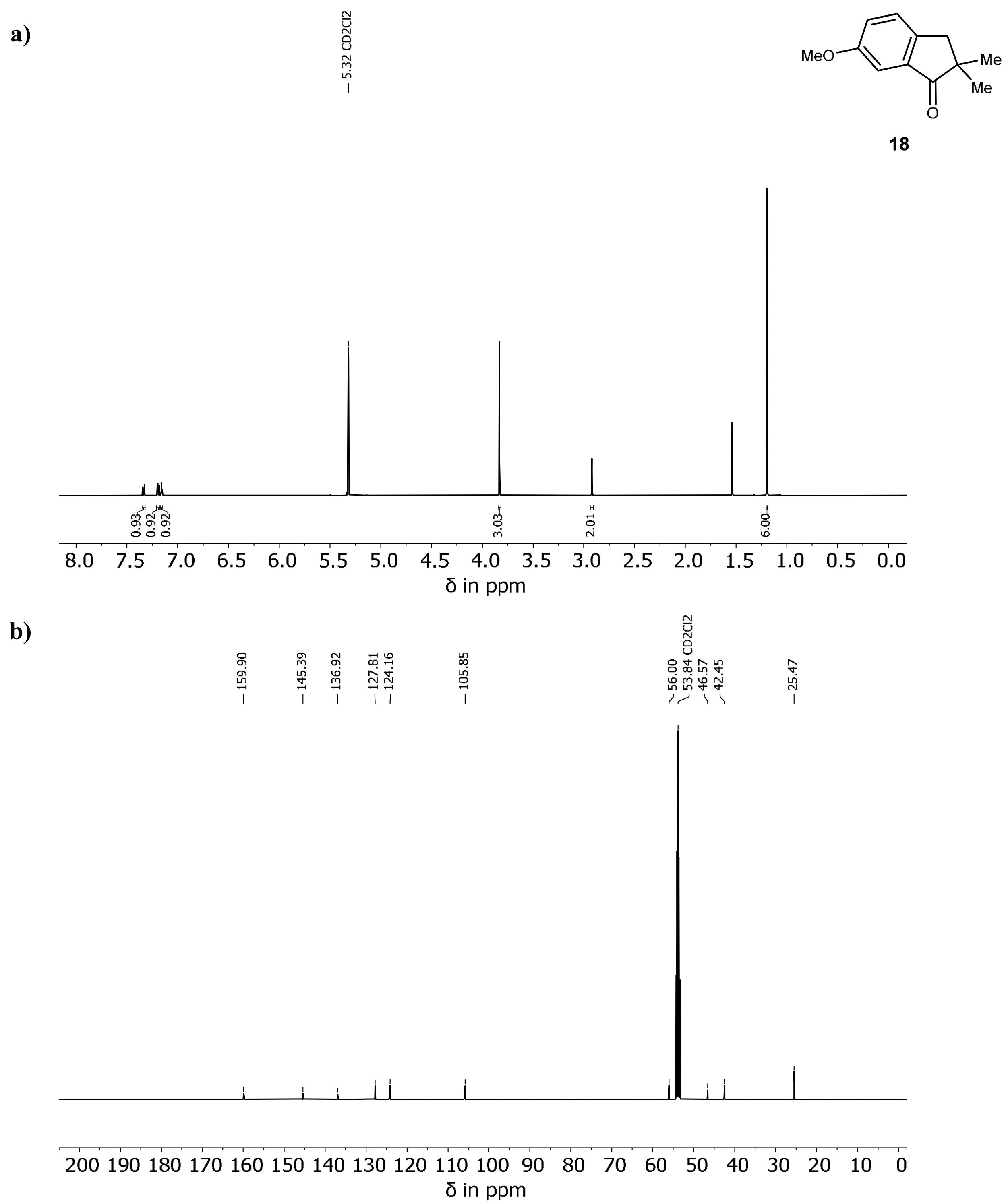

Supplementary Figure 101 NMR spectra of compound 18 in CD_2Cl_2 at 25 °C. a) ^{1}H NMR spectrum measured at 500 MHz and b) ^{13}C NMR spectrum measured at 126 MHz.

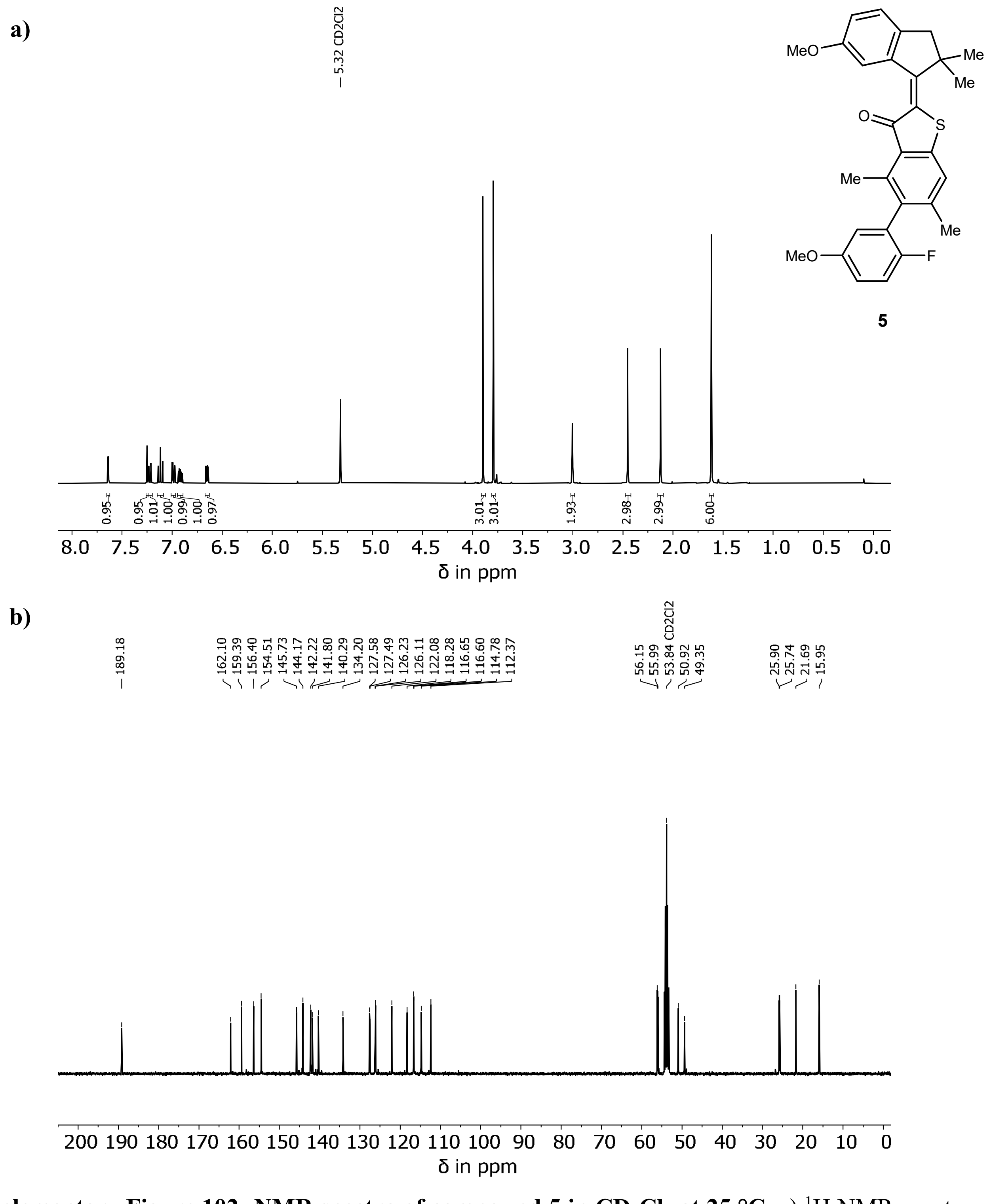

Supplementary Figure 102 NMR spectra of compound 5 in CD_2Cl_2 at 25 °C. a) ^{1}H NMR spectrum measured at 400 MHz and b) ^{13}C NMR spectrum measured at 126 MHz.

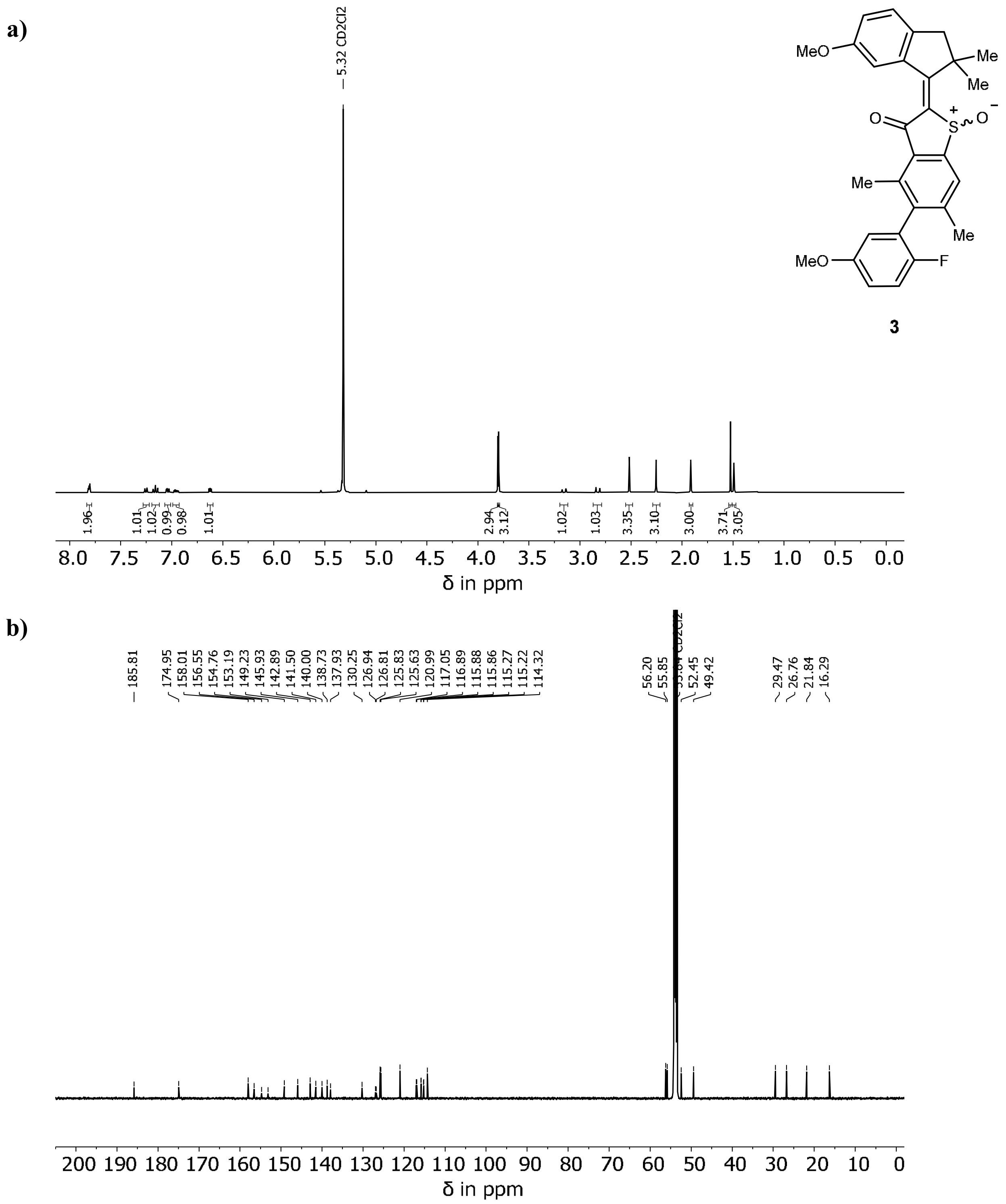

Supplementary Figure 103 NMR spectra of compound 3 in CD_2Cl_2 at 25 °C. a) ^{1}H NMR spectrum measured at 601 MHz and b) ^{13}C NMR spectrum measured at 151 MHz.

Supplementary References

[1] H. Volfova, Q. Hu, Riedle Eberhard, *EPA Newsletter* **2019**, 51.

[2] E. Uhl, P. Mayer, H. Dube, *Angew. Chem. Int. Ed.* **2020**, *59*, 5730-5737.

[3] M. J. Frisch, G. W. Trucks, H. B. Schlegel, G. E. Scuseria, M. A. Robb, J. R. Cheeseman, G. Scalmani, V. Barone, G. A. Petersson, H. Nakatsuji, X. Li, M. Caricato, A. V. Marenich, J. Bloino, B. G. Janesko, R. Gomperts, B. Mennucci, H. P. Hratchian, J. V. Ortiz, A. F. Izmaylov, J. L. Sonnenberg, Williams, F. Ding, F. Lipparini, F. Egidi, J. Goings, B. Peng, A. Petrone, T. Henderson, D. Ranasinghe, V. G. Zakrzewski, J. Gao, N. Rega, G. Zheng, W. Liang, M. Hada, M. Ehara, K. Toyota, R. Fukuda, J. Hasegawa, M. Ishida, T. Nakajima, Y. Honda, O. Kitao, H. Nakai, T. Vreven, K. Throssell, J. A. Montgomery Jr., J. E. Peralta, F. Ogliaro, M. J. Bearpark, J. J. Heyd, E. N. Brothers, K. N. Kudin, V. N. Staroverov, T. A. Keith, R. Kobayashi, J. Normand, K. Raghavachari, A. P. Rendell, J. C. Burant, S. S. Iyengar, J. Tomasi, M. Cossi, J. M. Millam, M. Klene, C. Adamo, R. Cammi, J. W. Ochterski, R. L. Martin, K. Morokuma, O. Farkas, J. B. Foresman, D. J. Fox, Wallingford, CT, **2016**.
