# Supplementary material for: A photochemical method to evidence directional molecular motions
Source: Nat Commun. 2023 Jul 31;14:4595. doi: 10.1038/s41467-023-40190-4 (PMC10390485; doi:10.1038/s41467-023-40190-4)
Supplement: Supplementary file 2 — Description of Additional Supplementary Files [file 41467_2023_40190_MOESM2_ESM.pdf]

Description of Additional Supplementary Files

File Name: Supplementary Movie 1
Description: Possible rotation directions of motor 1, 2 and 3 shown in structural representation (top) and modified Newman projection (bottom) with grey half circles shown for 180° motions in the sulfoxide half cycle (solid arrows) and dashed grey half circles shown for motions in the opposite 180° half cycle (dotted arrows). The indanone rotor and part of the benzothiophenone stator are colored purple and the biaryl axis part is sketched in dark red. Structural lability allows for different feasible rotation directions (Problem) in 1 (clockwise depicted by blue arrows and counterclockwise depicted by orange arrows). Macrocyclic setup 2 with locked atropisomers of the biaryl moiety restricts the motor motions to only one half-space with respect to the sulfoxide oxygen (Solution). This allows to measure quantum yields (QY) for the clockwise and counterclockwise motions on both sides. Comparing these quantum yields with the values measured for 3 delivers a prediction of the preferred rotation direction for an unrestricted fast molecular motor (Result)
