# Supplementary figures and images for: A photochemical method to evidence directional molecular motions
Source: Nat Commun. 2023 Jul 31;14:4595. doi: 10.1038/s41467-023-40190-4 (PMC10390485; doi:10.1038/s41467-023-40190-4)

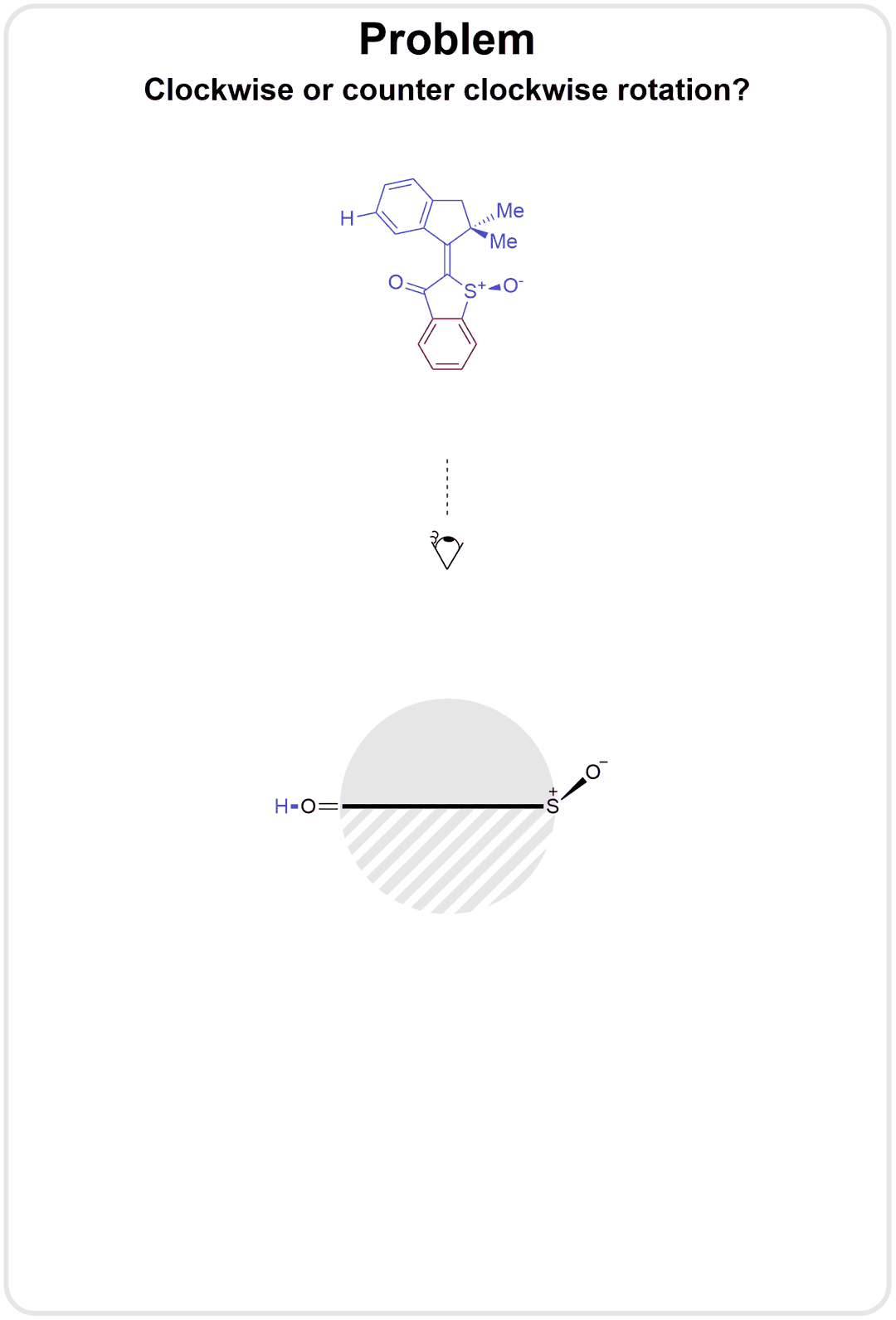

Supplement: Supplementary file 3 — Supplementary Movie 1 [file 41467_2023_40190_MOESM3_ESM.gif]
